# Gene expression profiles of skin from cyclin dependent kinases 5-knockdown mice

**DOI:** 10.5713/ab.23.0244

**Published:** 2023-11-02

**Authors:** Shanshan Yang, Dingxing Jiao, Tao Song, Ping Rui, Ruiwen Fan, Zengjun Ma

**Affiliations:** 1 College of Animal Science and Technology, Hebei Normal University of Science & Technology, Qinhuangdao 066600, China; 2 Hebei Key Laboratory of Veterinary Preventive Medicine, College of Animal Science and Technology, Hebei Normal University of Science & Technology, Qinhuangdao 066600, China; 3 College of Veterinary Medicine, Shanxi Agricultural University, Taigu, Shanxi 030801, China

**Keywords:** Cyclin Dependent Kinases 5 (CDK5), Gene Expression Profile, Melanogenesis, Pigmentation

## Abstract

**Objective:**

This study aimed to identify genes regulated by cyclin dependent kinases 5 (CDK5) that participate in hair pigmentation in mice.

**Methods:**

The mRNA expression profiles of skin samples from *CDK5*-knockdown mice were constructed using high-throughput RNA sequencing and compared with those of wild-type mice.

**Results:**

In total, 8,002 known genes were differentially expressed between *CDK5*-knockdown and wild-type mice. Of these, 3,658 were upregulated and 4,344 were downregulated in the skin of *CDK5*-knockdown mice. An additional 318 previously unknown genes were also differentially expressed, with 171 downregulated and 147 upregulated genes in the skin of *CDK5*-knockdown mice. Of the known genes expressed in mouse skin, 80 were associated with hair color, with 61 showing lower expression and 19 exhibiting higher expression in skin of *CDK5*-knockdown mice. Importantly, the expression of the tyrosinase-related protein 1 (*TYRP1*) and the calcium signaling pathway were also found to be regulated by CDK5, suggesting that pigmentation is regulated by CDK5 via the calcium signaling pathway and TYRP1.

**Conclusion:**

The transcriptome profiles obtained from the skin of *CDK5*-knockdown mice compared to wild-type mice provide a valuable resource to help understand the mechanism by which CDK5 regulates melanogenesis in mice and other animals.

## INTRODUCTION

Hair color is one of the most important factors affecting the economic value of fiber-producing animals. Hair color genes are also useful candidates for the traceability of farm animals [1]. Mouse models have been extensively used to investigate the functions of hair color-related genes. These models have received increasing attention, revealing that the fiber diameter, length, and color are determined by both genetics [2,3] and environment [4]. Hair and skin color depend on the pigments produced by melanocytes at the base of the epithelium [5]. Mammalian melanocytes produce two chemically distinct types of melanin, black/brown eumelanin and yellow/red-brown pheomelanin [6]. The quality and ratio of eumelanin to pheomelanin determine the final color of the hair and skin. Through extensive studies, the genetic basis for hair and skin color in rodents has been relatively well elucidated, with many genes involved in pigmentation being common to other species. For example, microphthalmia-associated transcription factor (MITF) plays an important role in skin color and melanoma [7] and the agouti signaling protein (ASIP) is a major regulator of mouse pigmentation [8].

Cyclin dependent kinases (CDKs) are proline-directed serine/threonine protein kinases that play important roles in cell cycle regulation [9]. However, cyclin-dependent kinase 5 (CDK5) is notable in that it does not appear to be directly involved in the cell cycle, is not activated after binding to cyclin, and does not require T-loop phosphorylation for activation [10]. An analysis of the transcriptome profile of alpaca skin with different hair colors revealed that *CDK5* is a candidate gene associated with alpaca fleece quality, coat color, and fiber growth and development [11]. Cyclin-dependent kinase 5 is localized in hair follicles, with higher expression levels in animals with brown than in those with white fleece colors. Our previous study investigating the genetic components of hair color in a mouse model showed that CDK5 plays a role in determining hair color in mice and a change in hair color from black to light brown was noted [11]. To further investigate the genes or pathways involved in pigmentation regulated by CDK5, *CDK5*-knockdown mice were generated, and skin mRNA profiles from *CDK5*-knockdown mice were constructed.

This study revealed that numerous mRNA transcripts are regulated by CDK5, providing insights into its potential functions. These data will contribute to a better understanding of how hair and skin pigmentation are determined in animals of economic importance and may lead to the design of more productive or desirable breeds.

## MATERIALS AND METHODS

### Ethics statement

The procedures for animal housing, care, and collection of skin samples were approved by the Animal Ethics Committee (2017[050]) at Shanxi Agriculture University (Taigu, Shanxi, China; Approval number: 2023008) and performed according to the Committee guidelines.

### Skin sampling and total RNA extraction

The *CDK5*-knockdown mouse models were prepared according to previously described instructions [12]. Three healthy wild-type and three *CDK5*-knockdown mice were randomly selected, and three pieces of skin (2×3 cm) from their backs were collected under local anesthesia and immediately stored in liquid nitrogen. Total RNA was extracted from the samples using the TRIzol reagent (Invitrogen, Carlsbad, CA, USA) according to the manufacturer’s instructions. The RNA integrity was evaluated by gel electrophoresis, and the concentration was measured by absorbance at optical density (OD_260/280_) using a NanoDrop spectrophotometer (Thermo Fisher Scientific, Waltham, MA, USA).

### Library generation and sequencing

After total RNA extraction and DNase I treatment, magnetic beads bound to oligo-dT were used to isolate the mRNAs. These were mixed with a fragmentation buffer to break the mRNAs into short fragments. cDNA was synthesized from the mRNA fragment templates. Short fragments were purified and resolved using the Qiagen EB buffer (Qiagen, Hilden, Germany) for end preparation and single-nucleotide adenine addition. These fragments were then ligated to adapters and suitably sized fragments were selected for polymerase chain reaction (PCR) amplification using agarose gel electrophoresis. During these quality control steps, an Agilent 2100 Bioanalyzer (Agilent Technologies, Santa Clara, CA, USA) and an ABI StepOnePlus Real-Time PCR System (Applied Biosystems, Foster City, CA, USA) were used for mRNA quantification and qualification. Finally, the library was sequenced using an Illumina HiSeq 2000 (Illumina, San Diego, CA, USA) to generate raw reads, which were filtered into clean reads by removing adaptors and low-quality reads, and then aligned to the reference sequences. These alignment data were used to calculate the distribution of reads on the reference genes and mapping ratios for further analyses, including gene ontology (GO) enrichment analysis, Kyoto encyclopedia of genes and genomes (KEGG) pathway enrichment analysis, and transcription factors identification.

### UniGene assembly and functional annotation

UniGene assembly was performed using the Trinity software (http://www.genomics.cn). BLASTX alignment (e-values <0.00001) between the unigenes and several protein databases (nr, Swiss-Prot, KEGG, and clusters of orthologous genes) was performed, with the best alignment results used to determine the sequence direction of the unigenes. GO functional annotation was based on the nr annotation, and Blast2GO (http://www.blast2go.com) was used to assign GO annotations. WEGO (http://wego.genomics.org.cn/cgibin/wego/index.pl) was used to perform the GO functional classifications of all unigenes.

### Identification of differentially expressed genes and pathway analysis

A rigorous algorithm was used to identify genes in the skin that are differentially expressed between *CDK5*-knockdown and wild-type mice. The algorithm consisted of a threshold false discovery rate value of ≤0.001 and a reads per kilobase of transcript per million mapped reads ratio of ≥2. Differentially expressed genes were mapped to each term in the GO database (http://www.geneontology.org/) and the number of genes assigned to each GO term was calculated. The calculated p-values were Bonferroni corrected and corrected p-values ≤0.05 were determined to be significant. GO terms that fulfilled this condition were defined as significantly enriched. The differentially expressed genes was also mapped to terms on the KEGG pathway database (http://www.genome.jp/kegg/pathway.html) to reveal the biological pathways that differed between *CDK5*-knockdown and wild-type mice.

### Validation of differential gene expression in the skins of *CDK5*-knockdown and wild-type mice

To validate the sequencing data, 10 genes were randomly selected from a list of differentially expressed genes using the quantitative real-time PCR (qRT-PCR). Total RNA from six samples used for RNA sequencing was used for this analysis. One microgram of DNase-treated RNA was converted to cDNA using the PrimeScript RT Reagent Kit (TaKaRa, Dalian, China). The cDNA was used for qRT-PCR quantification using mRNA-specific primers (Table 1). β-Actin was used as an endogenous control. The qRT-PCR was performed in triplicate using a Stratagene Mx3005P system (Stratagene, La Jolla, CA, USA). Each 10 μL PCR reaction volume included 5 μL of SYBR Premix Ex TaqTM II (TaKaRa, China), 0.2 μL of specific forward primer, 0.2 μL of reverse primer, 0.2 μL of ROX reference dye, 1 μL of 10-fold diluted cDNA, and 3.4 μL of water. The cycling parameters were 95°C for 30 s, followed by 40 cycles of 95°C for 5 s, 56°C or 58°C for 30 s, and 72°C for 15 s. Melting curve analysis was performed at each amplification step. The mRNA abundance of each target gene was quantified using the comparative threshold cycle (CT) method and normalized to β-actin [13]. Differences in the mRNA abundance of genes were determined using analysis of variance.

## RESULTS

### Sequencing statistics

**Figure 1:**
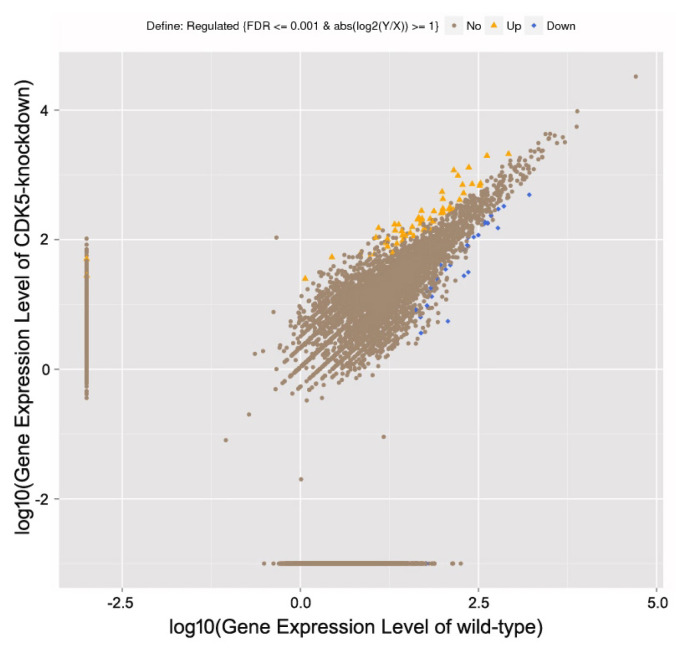
Statistically significant differentially expressed genes. Gene expression levels in the skin of *CDK5*-knockdown versus wild-type mice as revealed using high-throughput sequencing.

To identify the RNAs expressed in mouse skin, wild-type and CDK5-knockdown skin mRNA libraries were generated using high-throughput sequencing. A total of 98,801,386 and 96,503,448 raw reads were obtained from the wild-type and *CDK5*-knockdown mice, respectively. After filtering low-quality reads, 86,182,330 (87.23%) and 86,398,588 (89.53%) clean reads remained in the wild-type and *CDK5*-knockdown datasets, respectively. Using an algorithm based on a previously described method [14], 8,002 known genes were identified as differentially expressed between the skin of *CDK5*-knockdown and wild-type mice. Of these, 4,344 were downregulated (≤2-fold) and 3,658 were upregulated (≥2-fold) in *CDK5*-knockdown mice compared to wild-type (Figure 1; Supplementary Table S1).

### Gene ontology analysis of genes expressed in the skin of *CDK5*-knockdown versus wild-type mice

**Figure 2:**
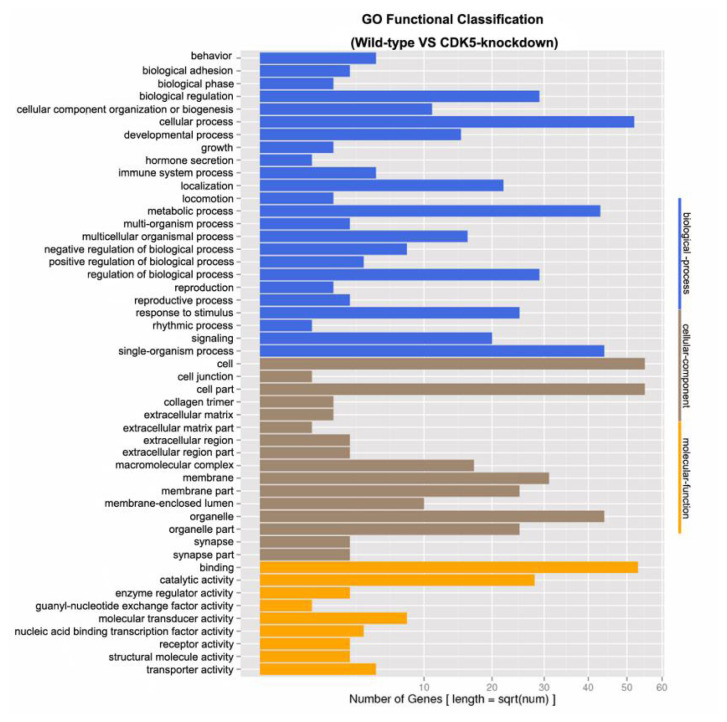
Gene ontology (GO) functional classification of differentially expressed unigenes. The GO functional classifications of unigenes that were differentially expressed between the skin of *CDK5*-knockdown and wild-type mice are shown. GO, gene ontology; *CDK5*, cyclin dependent kinases 5.

Genes differentially expressed between the skin of *CDK5*-knockdown and wild-type mice were grouped into 49 classes based on their putative functions. For subsequent GO analysis, the differentially expressed genes were characterized at the biological process (48.98%), cellular component (32.65%), and molecular function (18.37%) levels (Figure 2). A total of 318 unknown genes were also identified as differentially expressed, of which 171 were downregulated (≤2-fold) and 147 were upregulated (≥2-fold) in the skin of *CDK5*-knockdown mice compared to the skin of wild-type mice (Supplementary Table S2).

### Differential expression of known genes related to hair color formation

**Figure 3:**
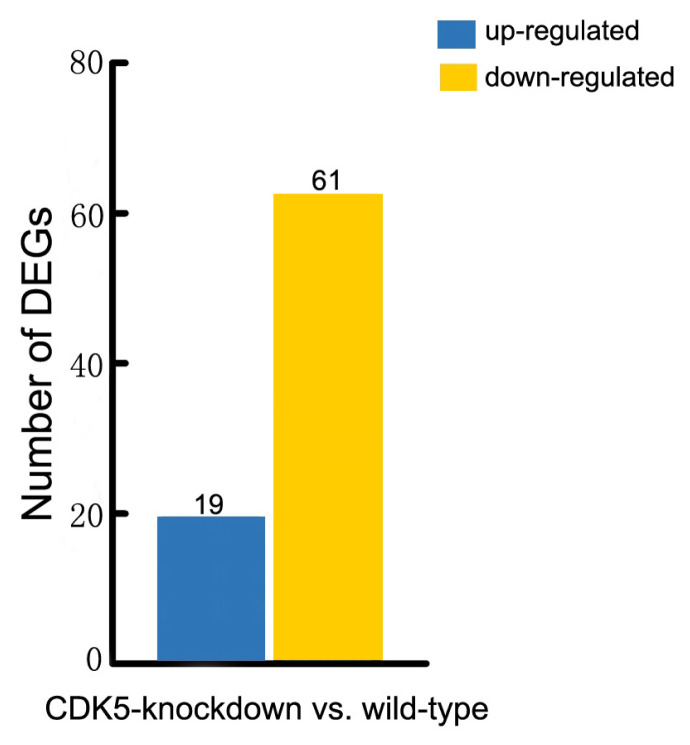
The number of statistically significant differentially expressed genes that affect hair color. Associated statistics of differentially expressed genes in the skin of wild-type versus *CDK5*-knockdown mice are shown. *CDK5*, cyclin dependent kinases 5.

More than 150 mutations affecting hair and skin pigmentation have been identified in mice. They are well dispersed throughout the mouse genome and are found at more than 50 distinct genetic loci [15]. Known hair color genes are typically classified into six functional groups: melanocyte development, melanosome components and precursors, melanosome construction and protein routing, melanosome transport, eumelanin and pheomelanin production, and other wider systemic effects [14]. In this study we found that 80 of these hair color genes are expressed in mouse skin. Our analysis revealed that 61 of these genes were expressed at lower levels in the skin of *CDK5*-knockdown mice than in wild-type mice, and 19 genes were expressed at higher levels (Figure 3; Supplementary Table S3). We also found that all the genes involved in encoding the components of melanosomes and their precursors were expressed at lower levels in the skin of *CDK5*-knockdown mice than in the skin of wild-type mice (Supplementary Table S4). Most genes involved in the production of eumelanin and pheomelanin also showed lower expression levels in the *CDK5*-knockdown mice. Among the hair color genes with lower expression in the skin of *CDK5*-knockdown mice, tyrosinase-related protein 1 (*TYRP1*) showed the largest decrease in expression between CDK5-knockdown mice and wild-type mice, followed by paired box3 (*PAX3*), dopachrome tautomerase (*DCT*), adenomatous polyposis coli (*Apc*), and lysosomal trafficking regulator (*Lyst*) genes (Table 2).

### Kyoto encyclopedia of genes and genomes pathway analysis

**Figure 4:**
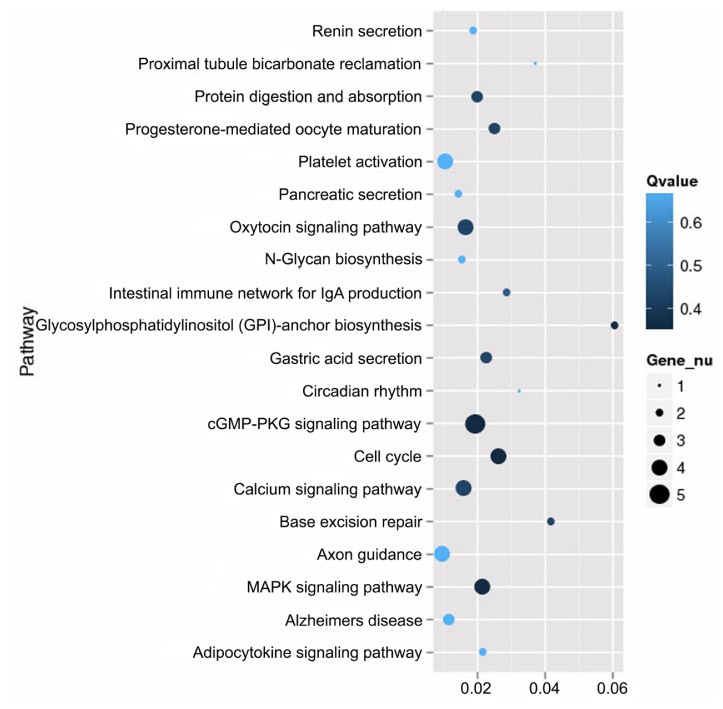
Pathway analysis comparing the skin of *CDK5*-knockdown mice to wild-type. The 20 most significant pathways between the skin of *CDK5*-knockdown mice relative to that of wild-type identified using Kyoto encyclopedia of genes and genomes (KEGG) pathway analysis. *CDK5*, cyclin dependent kinases 5.

**Figure 5:**
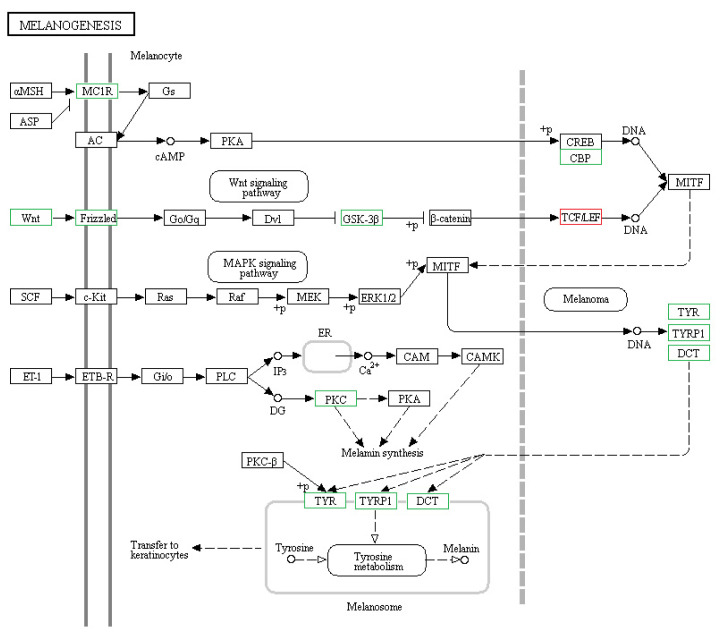
The expression of specific hair color genes. The expression of genes known to be involved in skin and hair pigmentation in mice, and their involvement in the melanogenesis pathway. Genes highlighted in red are upregulated in *CDK5*-knockdown mice versus wild-type, while those highlighted in green are downregulated. *CDK5*, cyclin dependent kinases 5.

Of the 8,004 genes that were differentially expressed between the skin of *CDK5*-knockdown mice and wild-type mice, 234 had specific KEGG annotations. The top 20 pathways that were most highly expressed in the skin of mice included the cGMP-PKG signaling pathway, calcium signaling pathway, and MAPK signaling pathway (Figure 4). The genes identified in the mouse skin transcriptome related to pigmentation and melanogenesis and their relative level of differential expression between the *CDK5*-knockdown and wild-type mice are shown in Table 3 and Figure 5.

### Validation of sequencing data by quantitative real-time polymerase chain reaction

**Figure 6:**
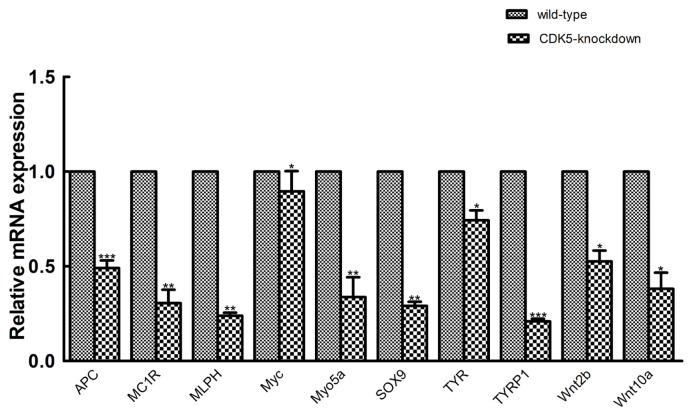
Quantitative real-time polymerase chain reaction (PCR) validation. Quantitative real-time PCR validation of 10 randomly selected genes that were determined to be differentially expressed between *CDK5*-knockdown and wild-type mice. The abundances of target genes were normalized to the abundance of a β-actin endogenous control. The bars in each panel represent the mean±standard error (n = 3). * p<0.05; ** p<0.01; *** p<0.001. *CDK5*, cyclin dependent kinases 5.

To validate the transcriptome sequencing results, we randomly selected 10 genes from a population of genes known to be involved in hair color for validation using qRT-PCR. Transcriptome sequencing analysis revealed that these genes were also differentially expressed between the *CDK5*-knockdown and wild-type mice. The results of qRT-PCR analysis showed that the mRNA expression of the selected genes was consistent with the transcriptome sequencing data (Figure 6). Among the differentially expressed genes, *TYRP1* showed the greatest difference in expression between *CDK5*-knockdown and wild-type mice, again supporting the sequencing data.

## DISCUSSION

Mammalian hair color exhibits a wide range of shades that are dictated by the production of melanin by melanocytes (melanogenesis). These are specialized cells that synthesize two primary melanins (eumelanin and pheomelanin) [16]. The quality of these melanins and the ratio of eumelanin to pheomelanin determine the final hair and skin colors [17]. During the development of skin and hair pigmentation, precisely coordinated mechanisms play a role in regulating the various processes that lead to the final color.

This study identified several genes known to be involved in hair color, such as *SOX9*. Sox (SRY type HMG box) proteins are transcription factors that belong to the HMG box superfamily of DNA-binding proteins and play a key role during development. SOX9 belongs to the SOX-E subgroup, which includes SOX8, SOX9, and SOX10. SOX9 directly binds and regulates the expression of MITF [18], which subsequently regulates the transcription of three major pigmentation enzyme genes, tyrosinase (*TYR*), tyrosine related protein-1 (*TYRP1*), and tyrosine related protein-2 (*TYRP2*; also known as *DCT*) [19]. When levels of MITF reach a certain threshold, repression is removed, allowing activation of TYRP2 transcription in the presence of β-catenin, leading to melanocyte maturation [20]. Ectopic SOX9 expression in the neural crest (NC) is sufficient to promote melanocytic differentiation, which suggests a role for SOX9 in melanocytic development [21]. In summary, SOX9 appears to be an important CDK5-regulated transcription factor that is involved in the differences in hair color observed in *CDK5*-knockdown mice. We also found some important transcription factors and signaling pathways in the *CDK5*-knockdown mice, such as PAX3 [11] and the MAPK signaling pathway.

Among the differentially expressed hair color genes identified, *TYRP1* showed the largest decrease in expression in the skins of *CDK5*-knockdown, compared to that of wild-type mice. *TYRP1*, a type I membrane-bound protein, is specifically expressed in melanocytes and involved in melanin production. TYRP1 is mostly expressed in the retinal pigment epithelium [22]. Melanocytes, which are derived from the NC, can be classified into two groups: cutaneous/classical and noncutaneous/nonclassical melanocytes. TYRP1 is also highly expressed in tumors derived from melanocytes such as in cutaneous and uveal melanomas. The three members of the tyrosinase family (TYR, TYRP2(DCT), and TYRP1) are regulated by the same transcription factor, MITF. In melanocytes, TYRP2 (DCT) catalyzes the rapid conversion of dopaquinone to 5,6-dihydroxyindole-2-carboxylic acid (DHICA), and then TYRP1 catalyzes the oxidation of DHICA to eumelanin [23]. TYRP1 also contributes to melanosomal structure and maturation [24]. Mutation of *TYRP1*, which leads to the mouse “light” phenotype (hairs pigmented only at their tips), has been associated with disrupted melanosomal structures. Therefore, it is likely that TYRP1 is an important multifunctional melanogenic protein that indirectly contributes to the regulation of melanin production in CDK5-knockdown mice.

Additionally, KEGG pathway analysis revealed that many differentially regulated pathways are involved in pigmentation or melanogenesis. These include the calcium, cGMP-PKG, and MAPK signaling pathways. In the electron transfer system, calcium plays an important role in both melanocyte function and survival [25]. An increase in free intracellular Ca^2+^ levels may be important for stimulating melanogenesis [26]. Previous studies have suggested that increasing Ca^2+^ level inhibits basal melanogenesis, as increasing the amount of Ca^2+^ in B16 [27] and human melanocytes [28] resulted in decreased tyrosinase activity, and hence a reduced basal melanin content.

In summary, our study revealed differentially expressed genes and pathways in the skin of *CDK5*-knockdown mice, indicating the physiological functions of CDK5 in melanogenesis. This finding that the CDK5 regulation of melanin production potentially occurs via the potential calcium signaling pathway and TYRP1 will contribute to the understanding of animal hair and skin color development.

## Figures and Tables

**Table 1:** Primers used for the quantitative real time polymerase chain reaction (qRT-PCR)

Genes	Primers (5′→3′)	Application
*APC*	F: TGAGGCACTGAAGATGGAGA	Real-time PCR
	R: AGGAGCGAAGGGACATTTTT	
*MC1R*	F:GTGAGTCTGGTGGAGAATGTG	Real-time PCR
	R:ATAGAAGATGGAGATGTAGCG	
*MLPH*	F: AGGAGGCGAGAGGAAGAAAG	Real-time PCR
	R: CTAGGCACTGTCGTCTGCTG	
*Myc*	F: CTGTGGAGAAGAGGCAAACC	Real-time PCR
	R: TTGGCAGCTGGATAGTCCTT	
*Myo5a*	F: GAGGCCAATGTTGAGGAAA	Real-time PCR
	R: CTTCTGCCTGGAACACCACT	
*SOX9*	F: AGGAAGCTGGCAGACCAGTA	Real-time PCR
	R: CGTTCTTCACCGACTTCCTC	
*TYR*	F: CACTCACAGGGATAGCAG	Real-time PCR
	R: TAGAGCGGTATGAAAGGA	
*TYRP1*	F: GTGCTTGGAGGTCCGTGTAT	Real-time PCR
	R: CAAAGACCGCATCAGTGAAA	
*Wnt2b*	F: CACCCGGACTGATCTTGTCT	Real-time PCR
	R: TGTTTCTGCACTCCTTGCAC	
*Wnt10a*	F: CGCTCTGGGTAAACTGAAGG	Real-time PCR
	R: GAAGTATGGCCGGGTGTTC	
*β* *-actin*	F:CTAAGGAGAAGGGCCAGTCC	Real-time PCR
	R:CTCAAGTTGGGGGACAAAAA	

F, forward primer; R, reverse primer; *APC*, adenomatosis polyposis coli; *MC1R*, Melanocortin 1 receptor; *MLPH*, melanophilin; *Myc*, myelocytomatosis oncogene; *Myo5a*, myosin 5a; *SOX9*, SRY-Box transcription factor 9; *TYR*, tyrosinase; *TYRP1*, tyrosinase-related protein 1.

**Table 2:** Highly downregulated coat color genes in *CDK5*-knockdown mice skin

Genes	log_2_Ratio (*CDK5*-knockdown/Wild-type)	Classification	Function
*TYRP1*	−13.68496773	Components of melanosomes and their precursors	Melanosomal enzyme/stabilizing factor
*PAX3*	−13.05494342	Melanocyte development	Transcription factor; neural tube development
*DCT*	−11.63026713	Components of melanosomes and their precursors	Melanosomal enzyme
*APC*	−10.15481811	Melanocyte development	Wnt pathway mediator; transcription factor
*Lyst*	−9.22881869	Melanosome construction / protein routing (HPS-related)	Organelle biogenesis and size

*CDK5*, cyclin dependent kinases 5; *TYRP1*, tyrosinase-related protein 1; *PAX3*, paired box 3; *DCT*, dopachrome tautomerase; *APC*, adenomatosis polyposis coli; *Lyst*, lysosomal trafficking regulator.

**Table 3:** Differentially expressed genes and their gene ontology terms related to pigmentation and melanogenesis in *CDK5*-knockdown versus wild-type mice skin

GO terms	Genes	Relative expression in CDK5-knockdown vs wild-type mice skin	Fold change
Melanocortin receptor binding	Melanocortin 2 receptor accessory protein 2 (*Mrap2*)	Down-regulation	12.65
	Melanocortin 1 receptor (*MC1R*)	Down-regulation	0.136
	Nonagouti (*a*)	Down-regulation	1.1
pigment cell differentiation	Tyrosinase-related protein 1 (*TYRP1*)	Down-regulation	13.68
	Melanophilin (*Mlph*)	Down-regulation	0.103
	SRY (sex determining region Y)-box 10 (*SOX10*)	Down-regulation	13.27
Tyrosine metabolic process	Tyrosinase-related protein 1 (*TYRP1*)	Down-regulation	13.68
Pigment granule organization	Dopachrome tautomerase (*TYRP2*;*DCT*)	Down-regulation	11.63
	Melanophilin (*Mlph*)	Down-regulation	0.103
	Lysosomal trafficking regulator (*Lyst*)	Down-regulation	9.23
	Vacuolar protein sorting 33B (yeast) (*Vps33b*)	Up-regulation	0.45
Melanin metabolic process	Dopachrome tautomerase (*TYRP2*; *DCT*)	Down-regulation	11.63
	Melanocortin 1 receptor (*MC1R*)	Down-regulation	0.13
	Premelanosome protein (*Pmel*)	Down-regulation	2.69
	Tyrosinase (*TYR*)	Down-regulation	0.76
Intracellular membrane-bounded organelle	Paired box 3 (*PAX3*)	Down-regulation	13.05

GO, gene ontology; *CDK5*, cyclin dependent kinases 5.

## Data Availability

The dataset generated in the current study is available from the corresponding authors upon request. The RNA-Seq data have been uploaded to the BioProject database under the accession numbers PRJNA1014499.

## SUPPLEMENTARY MATERIAL

**Supplementary Table S1**. Differentially expressed known genes in CDK5-knockdown versus wild-type mice skin

**Supplementary Table S2**. Differentially expressed unknown genes in CDK5-knockdown versus wild-type mice skin

**Supplementary Table S3**. Statistic of Differentially Expressed Genes in CDK5-knockdown wersus wild-type mice skin

**Supplementary Table S4**. Differentially expressed known hair color genes in CDK5-knockdown vs wild-type mice skin
