# Supplementary material for: Gene expression profiles of skin from cyclin dependent kinases 5-knockdown mice
Source: Anim Biosci. 2023 Nov 2;37(4):567–75. doi: 10.5713/ab.23.0244 (PMC10915219; doi:10.5713/ab.23.0244)
Supplement: Supplementary file 1 [file ab-23-0244-Supplementary-Table-S1.pdf]

Supplementary Table 1 Differentially expressed known genes in CDK5-knockdown versus wild-type mice skin

geneID	Wild-type-Expression	CDK5-knockdown-Expression	Wild-type-FPKM	CDK5-knockdown-FPKM	log2 Ratio(CDK5-knockdown/Wild-type)	Up-Down-Regulation(CDK5-knockdown/Wild-type)	P-value	FDR
68240	0	9	0.001	103.27	16.65606169	Up	0.001368742	0.036978254
319192	0	6.61	0.001	83.8	16.35466262	Up	0.01218244	0.160142312
17766	0	9	0.001	70.85	16.11248023	Up	0.001368742	0.03662155
66141	0	6	0.001	69.63	16.0874214	Up	0.01218244	0.157651762
11812	0	5	0.001	68.51	16.06402696	Up	0.0252468	0.245418952
259003	0	9.98	0.001	67.4	16.04046097	Up	0.001368742	0.036156515
12859	0	5	0.001	64.77	15.98303812	Up	0.0252468	0.243993755
68223	0	3	0.001	63.38	15.95174004	Up	0.1084294	0.512636953
621628	0	5	0.001	56.91	15.79639456	Up	0.0252468	0.246571154
13531	0	18.56	0.001	50.24	15.61654884	Up	1.94E-06	0.000139252
56312	0	4	0.001	47.11	15.52374571	Up	0.052321	0.340659656
66448	0	4	0.001	45.17	15.46307729	Up	0.052321	0.347179459
20287	0	3	0.001	44.81	15.45153311	Up	0.1084294	0.501245021
12858	0	4	0.001	42.35	15.37007435	Up	0.052321	0.355398401
223267	0	6	0.001	40.89	15.31946044	Up	0.01218244	0.156677099
76560	0	10	0.001	38.67	15.23892714	Up	0.000660466	0.020506484
18674	0	8	0.001	38.53	15.23369457	Up	0.00283656	0.061306534
54368	0	4	0.001	36.68	15.16270602	Up	0.052321	0.342266542
319159	0	2	0.001	36.62	15.16034417	Up	0.224708	0.690216046
27369	0	5	0.001	36.49	15.15521353	Up	0.0252468	0.249203586

69519	0	6	0.001	36.48	15.15481811	Up		0.01218244	0.157897326
383766	0	3	0.001	35.33	15.10860613	Up		0.1084294	0.500966706
14871	0	5	0.001	35.08	15.09836113	Up		0.0252468	0.24628209
67186	0	1.98	0.001	34.68	15.08181628	Up		0.46568	0.813376003
12257	0	4	0.001	34.05	15.05536718	Up		0.052321	0.346626625
319150	0	2.03	0.001	33.9	15.04899765	Up		0.224708	0.676726481
12238	0	4	0.001	33.65	15.03831888	Up		0.052321	0.360101771
73673	0	4	0.001	33.57	15.03488492	Up		0.052321	0.364015921
16833	0	6	0.001	33.48	15.03101191	Up		0.01218244	0.159637926
12957	0	3	0.001	33.24	15.02063276	Up		0.1084294	0.493297451
100191037	0	4	0.001	32.82	15.00228762	Up		0.052321	0.340926422
93725	0	2	0.001	32.6	14.99258434	Up		0.224708	0.69612631
319183	0	1.81	0.001	32.6	14.99258434	Up		0.46568	0.828860595
73316	0	6	0.001	31.95	14.96352831	Up		0.01218244	0.157406962
68953	0	4	0.001	31.91	14.96172099	Up		0.052321	0.354819104
14963	0	5	0.001	31.51	14.94352213	Up		0.0252468	0.245705992
68349	0	4	0.001	31.45	14.9407724	Up		0.052321	0.36250045
56743	0	7	0.001	31.1	14.92462696	Up		0.00587846	0.1021183
68468	0	4	0.001	30.77	14.90923682	Up		0.052321	0.352520681
26446	0	3	0.001	30.67	14.90454055	Up		0.1084294	0.507447153
67295	0	33.19	0.001	27.87	14.76642538	Up		3.48E-11	6.15E-09
17087	0	2	0.001	27.67	14.75603502	Up		0.224708	0.68591169
208990	0	2	0.001	27.67	14.75603502	Up		0.224708	0.6414392
67130	0	2	0.001	27.09	14.72547277	Up		0.224708	0.67943142
270150	0	4	0.001	27.08	14.72494012	Up		0.052321	0.363105122
78372	0	3	0.001	26.59	14.69859616	Up		0.1084294	0.504327019

70713	0	9	0.001	26.57	14.69751061	Up	0.001368742	0.036504174
30951	0	5	0.001	25.81	14.65564252	Up	0.0252468	0.243710699
58804	0	3	0.001	25.66	14.64723355	Up	0.1084294	0.510606133
408193	0	3	0.001	25.48	14.63707766	Up	0.1084294	0.509452873
66445	0	4	0.001	24.55	14.5834354	Up	0.052321	0.343617238
56640	0	4	0.001	23.67	14.53077209	Up	0.052321	0.348290433
66736	0	4	0.001	23.52	14.52160044	Up	0.052321	0.346075549
71790	0	6	0.001	23.2	14.50183718	Up	0.01218244	0.159889721
72315	0	4	0.001	22.89	14.48242984	Up	0.052321	0.366158992
71997	0	7	0.001	22.66	14.46786024	Up	0.00587846	0.104295662
12775	0	6	0.001	22.58	14.46275787	Up	0.01218244	0.161160705
11604	0	2	0.001	22.51	14.45827844	Up	0.224708	0.629772741
16012	0	4	0.001	22.41	14.45185503	Up	0.052321	0.344978638
11308	0	10	0.001	22.28	14.44346161	Up	0.000660466	0.020583287
101358	0	13	0.001	22.09	14.4311058	Up	7.42E-05	0.003355796
52717	0	4	0.001	21.99	14.42455998	Up	0.052321	0.356270901
436090	0	6	0.001	21.93	14.42061819	Up	0.01218244	0.160649894
665891	0	3	0.001	21.87	14.4166656	Up	0.1084294	0.510317329
228033	0	2	0.001	21.86	14.41600578	Up	0.224708	0.683654577
18301	0	3	0.001	21.56	14.39606956	Up	0.1084294	0.507732717
258457	0	3	0.001	21.28	14.37721053	Up	0.1084294	0.504891459
14317	0	8	0.001	21.26	14.37585398	Up	0.00283656	0.061147709
664968	0	3	0.001	21.01	14.35878854	Up	0.1084294	0.4971025
66910	0	3	0.001	20.91	14.35190544	Up	0.1084294	0.497925517
20646	0	6	0.001	20.91	14.35190544	Up	0.01218244	0.159386923
68736	0	4	0.001	20.86	14.34845154	Up	0.052321	0.34968919

69168	0	2	0.001	20.5	14.32333629	Up	0.224708	0.633828904
244551	0	2	0.001	20.48	14.32192809	Up	0.224708	0.672346375
94043	0	3	0.001	20.43	14.31840158	Up	0.1084294	0.489284727
242681	0	2	0.001	20.33	14.31132259	Up	0.224708	0.638155382
554292	0	4	0.001	20.12	14.29634268	Up	0.052321	0.340127376
171209	0	5	0.001	19.81	14.27394126	Up	0.0252468	0.246860896
73660	0	4	0.001	19.8	14.27321281	Up	0.052321	0.357147696
17152	0	9	0.001	19.75	14.26956503	Up	0.001368742	0.036042096
21917	0	9	0.001	19.69	14.26517549	Up	0.001368742	0.036387547
75586	0	2	0.001	19.66	14.2629757	Up	0.224708	0.647436035
69769	0	3	0.001	19.64	14.26150731	Up	0.1084294	0.497376537
56486	0	3	0.001	19.64	14.26150731	Up	0.1084294	0.476871584
23789	0	5	0.001	19.48	14.24970606	Up	0.0252468	0.247151321
80886	0	6	0.001	19.44	14.2467406	Up	0.01218244	0.15716292
94063	0	3	0.001	19.42	14.24525558	Up	0.1084294	0.496282199
18406	0	2	0.001	19.26	14.23332008	Up	0.224708	0.677462054
670482	0	2	0.001	19.18	14.2273151	Up	0.224708	0.645647537
66311	0	4	0.001	19.07	14.21901722	Up	0.052321	0.353953692
12391	0	3	0.001	18.77	14.19614103	Up	0.1084294	0.495736834
78284	0	4	0.001	18.72	14.19229281	Up	0.052321	0.3513826
26897	0	4	0.001	18.67	14.18843431	Up	0.052321	0.35081631
17089	0	4	0.001	18.56	14.17990909	Up	0.052321	0.3649313
75553	0	6	0.001	18.37	14.16506401	Up	0.01218244	0.160395701
53893	0	4	0.001	18.3	14.15955603	Up	0.052321	0.35744092
20972	0	2	0.001	18.18	14.15006458	Up	0.224708	0.643868894
380773	0	1	0.001	18.12	14.14529533	Up	0.46568	0.786626732

26434	0	8	0.001	18.1	14.14370208	Up	0.00283656	0.061626673
66174	0	3	0.001	17.71	14.11227659	Up	0.1084294	0.489815981
14373	0	2	0.001	17.53	14.09753838	Up	0.224708	0.669218063
12613	0	5	0.001	17.39	14.08597031	Up	0.0252468	0.244561843
101122	0	3	0.001	17.29	14.07765025	Up	0.1084294	0.4954646
238323	0	6	0.001	17.07	14.05917544	Up	0.01218244	0.160904894
11629	0	2	0.001	16.94	14.04814625	Up	0.224708	0.69200417
17235	0	2	0.001	16.67	14.02496648	Up	0.224708	0.685408823
14133	0	3	0.001	16.65	14.02323456	Up	0.1084294	0.51292839
57444	0	2	0.001	16.63	14.02150055	Up	0.224708	0.644756989
171201	0	2	0.001	16.57	14.01628598	Up	0.224708	0.678938006
109979	0	3	0.001	16.56	14.01541505	Up	0.1084294	0.517934005
232334	0	3	0.001	16.52	14.01192607	Up	0.1084294	0.504609081
20778	0	5	0.001	16.45	14.00579996	Up	0.0252468	0.255570101
244218	0	2	0.001	16.45	14.00579996	Up	0.224708	0.646317065
56040	0	1	0.001	16.41	14.00228762	Up	0.46568	0.844025981
66124	0	2	0.001	16.36	13.99788513	Up	0.224708	0.699250287
241850	0	4	0.001	16.35	13.99700302	Up	0.052321	0.358914296
98845	0	7	0.001	16.35	13.99700302	Up	0.00587846	0.102978243
100312484	0	2	0.001	16.34	13.99612036	Up	0.224708	0.650363571
14567	0	6	0.001	16.27	13.98992663	Up	0.01218244	0.158638628
234388	0	2	0.001	16.25	13.9881521	Up	0.224708	0.670658274
233005	0	5	0.001	16.23	13.98637538	Up	0.0252468	0.25341209
241070	0	4	0.001	16.14	13.97835296	Up	0.052321	0.350533849
26565	0	2	0.001	15.97	13.96307669	Up	0.224708	0.639027774
20909	0	3	0.001	15.96	13.96217303	Up	0.1084294	0.482481838

68036	0	9.97	0.001	15.93	13.95945865	Up	0.001368742	0.036271663
16008	0	2	0.001	15.85	13.95219522	Up	0.224708	0.652405886
109820	0	3	0.001	15.82	13.94946198	Up	0.1084294	0.477881905
547109	0	4	0.001	15.8	13.94763694	Up	0.052321	0.344432786
66498	0	4	0.001	15.62	13.93110683	Up	0.052321	0.35195072
101142	0	5	0.001	15.6	13.92925841	Up	0.0252468	0.253106774
259116	0	2	0.001	15.44	13.91438513	Up	0.224708	0.639683636
28200	0	2	0.001	15.35	13.90595104	Up	0.224708	0.691236698
70405	0	3	0.001	15.34	13.90501086	Up	0.1084294	0.509165371
330096	0	8	0.001	15.29	13.90030079	Up	0.00283656	0.061466187
108832	0	3	0.001	15.1	13.88226093	Up	0.1084294	0.486383309
81489	0	6	0.001	15.08	13.88034881	Up	0.01218244	0.16141733
16980	0	7	0.001	15.03	13.87555739	Up	0.00587846	0.103413669
448850	0	2	0.001	15.01	13.87363636	Up	0.224708	0.667545615
407790	0	2	0.001	14.96	13.86882255	Up	0.224708	0.673072451
110842	0	3	0.001	14.93	13.86592655	Up	0.1084294	0.496555332
18675	0	13.01	0.001	14.84	13.85720347	Up	7.42E-05	0.003374134
72303	0	3.97	0.001	14.82	13.85525783	Up	0.1084294	0.492489649
57751	0	3	0.001	14.62	13.83565569	Up	0.1084294	0.487961621
71838	0	4	0.001	14.6	13.83368075	Up	0.052321	0.364625662
105886298	0	2	0.001	14.58	13.8317031	Up	0.224708	0.689706849
14356	0	2	0.001	14.55	13.82873153	Up	0.224708	0.639246245
57277	0	1	0.001	14.51	13.8247599	Up	0.46568	0.781865069
11746	0	4	0.001	14.45	13.81878187	Up	0.052321	0.345800668
66119	0	2	0.001	14.36	13.80976813	Up	0.224708	0.678691567
319822	0	7	0.001	14.29	13.8027183	Up	0.00587846	0.104073757

278672	0	4	0.001	14.27	13.80069771	Up	0.052321	0.35861865
58864	0	2	0.001	14.24	13.79766153	Up	0.224708	0.631474255
74246	0	3	0.001	14.21	13.79461893	Up	0.1084294	0.50716191
22144	0	3	0.001	14.18	13.79156991	Up	0.1084294	0.491149177
72074	0	4	0.001	14.13	13.78647385	Up	0.052321	0.365237451
225896	0	2	0.001	14.05	13.77828251	Up	0.224708	0.647884708
53422	0	3	0.001	13.96	13.76901132	Up	0.1084294	0.485597975
16985	0	4	0.001	13.92	13.76487159	Up	0.052321	0.339332066
214254	0	5	0.001	13.9	13.76279726	Up	0.0252468	0.252498345
17252	0	3	0.001	13.86	13.75863964	Up	0.1084294	0.485075827
56437	0	3	0.001	13.84	13.75655632	Up	0.1084294	0.48533676
56399	0	7.28	0.001	13.75	13.747144	Up	0.00587846	0.103632766
24109	0	5	0.001	13.51	13.72174005	Up	0.0252468	0.255881392
78753	0	5	0.001	13.34	13.70347105	Up	0.0252468	0.250093599
225655	0	4	0.001	13.34	13.70347105	Up	0.052321	0.356854952
230777	0	4	0.001	13.29	13.69805348	Up	0.052321	0.35223547
104349	0	3.74	0.001	13.25	13.69370474	Up	0.1084294	0.5006887
16524	0	6	0.001	13.19	13.68715694	Up	0.01218244	0.159136708
22178	0	5	0.001	13.17	13.68496773	Up	0.0252468	0.252195225
327942	0	3	0.001	13.15	13.68277518	Up	0.1084294	0.502641246
69547	0	5	0.001	13.08	13.67507492	Up	0.0252468	0.254640755
14714	0	1	0.001	13.05	13.67176219	Up	0.46568	0.830281397
12931	0	3	0.001	13.04	13.67065625	Up	0.1084294	0.47737621
69923	0	4	0.001	12.99	13.66511381	Up	0.052321	0.36159721
17748	0	1	0.001	12.98	13.66400276	Up	0.46568	0.839818656
83409	0	1	0.001	12.91	13.65620138	Up	0.46568	0.837097274

71091	0	3	0.001	12.9	13.65508345	Up	0.1084294	0.495192666
22163	0	2	0.001	12.7	13.63254088	Up	0.224708	0.649234468
259002	0	2.02	0.001	12.7	13.63254088	Up	0.224708	0.644979396
21809	0	6	0.001	12.67	13.6291289	Up	0.01218244	0.158143656
71020	0	2	0.001	12.65	13.62684976	Up	0.224708	0.642100023
235169	0	4	0.001	12.62	13.62342429	Up	0.052321	0.346902822
107328	0	2	0.001	12.56	13.61654884	Up	0.224708	0.670417809
225432	0	11	0.001	12.55	13.61539974	Up	0.000318698	0.011284622
56320	0	5	0.001	12.38	13.59572369	Up	0.0252468	0.243146554
18160	0	7	0.001	12.35	13.59222342	Up	0.00587846	0.103852793
101883	0	2	0.001	12.3	13.5863707	Up	0.224708	0.647211931
13528	0	4	0.001	12.26	13.58167136	Up	0.052321	0.347734058
67268	0	3	0.001	12.23	13.57813678	Up	0.1084294	0.512345847
280667	0	4.89	0.001	12.2	13.57459353	Up	0.052321	0.345252213
109978	0	4	0.001	12.11	13.56391124	Up	0.052321	0.352806354
98999	0	10	0.001	12.02	13.55314928	Up	0.000660466	0.020738632
667034	0	2	0.001	11.98	13.54834029	Up	0.224708	0.641219228
665992	0	1	0.001	11.96	13.54592977	Up	0.46568	0.854825343
67264	0	1	0.001	11.74	13.51914479	Up	0.46568	0.765341355
72522	0	4	0.001	11.73	13.51791539	Up	0.052321	0.35921043
67883	0	2	0.001	11.59	13.50059295	Up	0.224708	0.657452626
213765	0	6	0.001	11.54	13.4943556	Up	0.01218244	0.158887278
56370	0	2	0.001	11.53	13.49310489	Up	0.224708	0.652861476
76238	0	2	0.001	11.43	13.48053778	Up	0.224708	0.681660688
12585	0	4	0.001	11.42	13.47927503	Up	0.052321	0.351666431
407786	0	4	0.001	11.35	13.47040468	Up	0.052321	0.362802534

69912	0	2	0.001	11.34	13.46913302	Up		0.224708	0.652178329
94065	0	1	0.001	11.33	13.46786024	Up		0.46568	0.806771451
225362	0	3	0.001	11.32	13.46658634	Up		0.1084294	0.489019532
625068	0	4	0.001	11.31	13.46531131	Up		0.052321	0.34940854
252868	0	1.98	0.001	11.23	13.45507031	Up		0.46568	0.823750697
70335	0	3	0.001	11.23	13.45507031	Up		0.1084294	0.490615028
218397	0	8	0.001	11.23	13.45507031	Up		0.00283656	0.060676133
101351	0	5	0.001	11.21	13.45249866	Up		0.0252468	0.248908321
66790	0	2	0.001	11.16	13.44604941	Up		0.224708	0.661172301
94245	0	2	0.001	11.15	13.44475609	Up		0.224708	0.665170853
13797	0	4	0.001	11.1	13.43827206	Up		0.052321	0.357734627
114332	0	4	0.001	11.06	13.43306377	Up		0.052321	0.348848591
15500	0	4	0.001	10.99	13.42390377	Up		0.052321	0.348012023
20877	0	3	0.001	10.96	13.41996018	Up		0.1084294	0.498751264
19245	0	3.99	0.001	10.9	13.41204051	Up		0.1084294	0.515566307
14525	0	5	0.001	10.89	13.41071633	Up		0.0252468	0.245132582
207781	0	10	0.001	10.88	13.40939094	Up		0.000660466	0.020660668
107734	0	1	0.001	10.85	13.40540742	Up		0.46568	0.838909565
232933	0	3	0.001	10.8	13.39874369	Up		0.1084294	0.480426537
26938	0	3	0.001	10.72	13.38801729	Up		0.1084294	0.499303286
19088	0	5	0.001	10.7	13.38532318	Up		0.0252468	0.252802194
18857	0	2	0.001	10.7	13.38532318	Up		0.224708	0.637068234
53321	0	8	0.001	10.68	13.38262403	Up		0.00283656	0.060989705
19331	0	2	0.001	10.65	13.37856581	Up		0.224708	0.637502648
15417	0	4	0.001	10.63	13.37585398	Up		0.052321	0.354241693
246779	0	1	0.001	10.62	13.37449615	Up		0.46568	0.766400965

216705	0	5	0.001	10.57	13.36768776	Up		0.0252468	0.251591165
59008	0	4	0.001	10.56	13.36632221	Up		0.052321	0.341461209
66128	0	1	0.001	10.54	13.36358725	Up		0.46568	0.802593886
14466	0	3	0.001	10.49	13.35672706	Up		0.1084294	0.477628924
14359	0	3	0.001	10.44	13.34983409	Up		0.1084294	0.514977761
21366	0	9	0.001	10.43	13.34845154	Up		0.001368742	0.036858583
67468	0	4	0.001	10.36	13.33873638	Up		0.052321	0.341729232
73834	0	2	0.001	10.35	13.33734315	Up		0.224708	0.684154873
15270	0	2	0.001	10.32	13.33315535	Up		0.224708	0.664934306
15356	0	2	0.001	10.3	13.33035672	Up		0.224708	0.693030122
16005	0	3	0.001	10.28	13.32755264	Up		0.1084294	0.513512258
83383	0	3	0.001	10.27	13.32614856	Up		0.1084294	0.499027122
14705	0	2	0.001	10.26	13.32474311	Up		0.224708	0.687930562
18717	0	6	0.001	10.19	13.31486643	Up		0.01218244	0.158390755
140859	0	4	0.001	10.17	13.31203206	Up		0.052321	0.354530164
21646	0	2	0.001	10.17	13.31203206	Up		0.224708	0.656529237
20704	0	1.91	0.001	10.16	13.31061278	Up		0.46568	0.827267993
270076	0	3	0.001	10.13	13.30634655	Up		0.1084294	0.48955021
192197	0	5	0.001	10.08	13.29920802	Up		0.0252468	0.24484688
16977	0	2	0.001	10	13.28771238	Up		0.224708	0.632114695
100039257	0	1.98	0.001	9.99	13.28626896	Up		0.46568	0.802261549
67874	0	2	0.001	9.98	13.2848241	Up		0.224708	0.629348794
73683	0	3	0.001	9.93	13.277578	Up		0.1084294	0.4845548
320664	0	5	0.001	9.9	13.27321281	Up		0.0252468	0.24744243
17907	0	1	0.001	9.88	13.27029533	Up		0.46568	0.84072972
26432	0	5	0.001	9.82	13.26150731	Up		0.0252468	0.254332473

71877	0	3	0.001	9.82	13.26150731	Up	0.1084294	0.485859471
170786	0	2	0.001	9.82	13.26150731	Up	0.224708	0.637937655
53860	0	4	0.001	9.77	13.25414285	Up	0.052321	0.342535831
216443	0	4	0.001	9.76	13.25266543	Up	0.052321	0.367085195
68090	0	2	0.001	9.76	13.25266543	Up	0.224708	0.675992505
26940	0	2	0.001	9.74	13.24970606	Up	0.224708	0.684655902
270160	0	4	0.001	9.69	13.24228095	Up	0.052321	0.34997029
76799	0	1	0.001	9.66	13.23780747	Up	0.46568	0.78297096
330959	0	1	0.001	9.54	13.21977355	Up	0.46568	0.831528601
19746	0	2	0.001	9.52	13.21674586	Up	0.224708	0.628925418
67463	0	4	0.001	9.49	13.21219237	Up	0.052321	0.350251843
22320	0	1	0.001	9.48	13.21067134	Up	0.46568	0.855202666
75573	0	1	0.001	9.44	13.20457114	Up	0.46568	0.818703419
66440	0	1	0.001	9.38	13.19537221	Up	0.46568	0.834213839
14168	0	3	0.001	9.33	13.18766137	Up	0.1084294	0.508018602
67320	0	1.08	0.001	9.32	13.18611424	Up	0.46568	0.762930356
56629	0	3	0.001	9.29	13.18146288	Up	0.1084294	0.479150843
243385	0	4	0.001	9.28	13.17990909	Up	0.052321	0.362198869
107993	0	2	0.001	9.27	13.17835362	Up	0.224708	0.699511885
16513	0	7	0.001	9.26	13.17679648	Up	0.00587846	0.103195497
66695	0	3	0.001	9.23	13.17211493	Up	0.1084294	0.5161562
50849	0	4	0.001	9.22	13.17055104	Up	0.052321	0.361297129
76938	0	2	0.001	9.19	13.16584915	Up	0.224708	0.671621864
80287	0	3	0.001	9.18	13.16427844	Up	0.1084294	0.514683992
26394	0	2	0.001	9.13	13.15639914	Up	0.224708	0.640999406
216445	0	3	0.001	9.13	13.15639914	Up	0.1084294	0.480170855

414077	0	1	0.001	9.12	13.15481811	Up	0.46568	0.836555112
227613	0	2	0.001	9.09	13.15006458	Up	0.224708	0.688690706
13363	0	3	0.001	9.09	13.15006458	Up	0.1084294	0.487171187
217707	0	2	0.001	9.08	13.14847658	Up	0.224708	0.649685639
19214	0	4	0.001	9.02	13.13891172	Up	0.052321	0.342805544
12075	0	3	0.001	8.97	13.13089227	Up	0.1084294	0.506308102
74281	0	2	0.001	8.97	13.13089227	Up	0.224708	0.644534736
13537	0	2	0.001	8.92	13.12282799	Up	0.224708	0.653774569
20597	0	3	0.001	8.91	13.12120972	Up	0.1084294	0.479660307
330483	0	2	0.001	8.89	13.1179677	Up	0.224708	0.65195093
67432	0	2	0.001	8.89	13.1179677	Up	0.224708	0.635984785
269120	0	2	0.001	8.88	13.11634396	Up	0.224708	0.666593678
233893	0	3	0.001	8.76	13.09671515	Up	0.1084294	0.480682492
57390	0	1	0.001	8.73	13.09176594	Up	0.46568	0.855013963
66264	0	1	0.001	8.67	13.08181628	Up	0.46568	0.781234532
320825	0	3	0.001	8.67	13.08181628	Up	0.1084294	0.484034891
83768	0	2	0.001	8.63	13.07514484	Up	0.224708	0.679184623
208285	0	2	0.001	8.58	13.06676193	Up	0.224708	0.668739366
547176	0	4	0.001	8.54	13.06002035	Up	0.052321	0.367394971
68035	0	2	0.001	8.54	13.06002035	Up	0.224708	0.682905503
18505	0	4	0.001	8.51	13.05494342	Up	0.052321	0.353379092
71703	0	4	0.001	8.49	13.05154884	Up	0.052321	0.359804166
381677	0	3	0.001	8.47	13.04814625	Up	0.1084294	0.50859134
68236	0	1	0.001	8.42	13.03960452	Up	0.46568	0.796326198
17441	0	2	0.001	8.42	13.03960452	Up	0.224708	0.651042921
17117	0	3	0.001	8.4	13.03617361	Up	0.1084294	0.48743438

114679	0	1	0.001	8.34	13.02583167	Up	0.46568	0.820784427
20345	0	3	0.001	8.3	13.01889562	Up	0.1084294	0.484294706
320162	0	3	0.001	8.29	13.01715639	Up	0.1084294	0.503201917
66042	0	2	0.001	8.29	13.01715639	Up	0.224708	0.672104697
107503	0	2	0.001	8.28	13.01541505	Up	0.224708	0.698988885
18718	0	4	0.001	8.28	13.01541505	Up	0.052321	0.360399868
70387	0	4	0.001	8.28	13.01541505	Up	0.052321	0.339596756
242037	0	2	0.001	8.25	13.0101784	Up	0.224708	0.63620118
216011	0	3	0.001	8.2	13.00140819	Up	0.1084294	0.509740699
57357	0	2	0.001	8.2	13.00140819	Up	0.224708	0.667784024
17120	0	3	0.001	8.19	12.99964774	Up	0.1084294	0.511474511
76775	0	4	0.001	8.16	12.99435344	Up	0.052321	0.365544115
18770	0	3	0.001	8.12	12.98726401	Up	0.1084294	0.486645651
23945	0	4	0.001	8.11	12.9854862	Up	0.052321	0.341193606
73327	0	1	0.001	8.1	12.98370619	Up	0.46568	0.829392825
234395	0	4	0.001	8.09	12.98192399	Up	0.052321	0.353666158
22378	0	2	0.001	8.08	12.98013958	Up	0.224708	0.654919533
14569	0	3	0.001	8.06	12.97656412	Up	0.1084294	0.494378651
18256	0	2	0.001	8.06	12.97656412	Up	0.224708	0.642541329
237222	0	5	0.001	8.05	12.97477307	Up	0.0252468	0.248026709
213539	0	2	0.001	7.99	12.96397979	Up	0.224708	0.633399481
20259	0	3	0.001	7.98	12.96217303	Up	0.1084294	0.483516097
57896	0	2	0.001	7.97	12.96036401	Up	0.224708	0.660705042
116972	0	1	0.001	7.95	12.95673915	Up	0.46568	0.780605012
67515	0	2	0.001	7.92	12.95128471	Up	0.224708	0.678445308
541610	0	3	0.001	7.92	12.95128471	Up	0.1084294	0.494107907

15424	0	3	0.001	7.91	12.94946198	Up	0.1084294	0.498200462
227095	0	2	0.001	7.89	12.94580958	Up	0.224708	0.628713944
258989	0	1	0.001	7.85	12.93847694	Up	0.46568	0.835292796
258436	0	1	0.001	7.85	12.93847694	Up	0.46568	0.795835547
71780	0	2	0.001	7.84	12.93663794	Up	0.224708	0.659307217
11690	0	1	0.001	7.84	12.93663794	Up	0.46568	0.762479984
70604	0	2	0.001	7.83	12.93479659	Up	0.224708	0.695608359
18019	0	3	0.001	7.82	12.93295289	Up	0.1084294	0.479915445
19141	0	2	0.001	7.73	12.9162527	Up	0.224708	0.643425763
436440	0	1	0.001	7.69	12.90876788	Up	0.46568	0.769903294
14699	0	1	0.001	7.67	12.90501086	Up	0.46568	0.844577873
74035	0	4	0.001	7.64	12.89935692	Up	0.052321	0.365851295
21761	0	2	0.001	7.64	12.89935692	Up	0.224708	0.637285367
330301	0	3	0.001	7.63	12.89746734	Up	0.1084294	0.491952583
67556	0	8	0.001	7.55	12.88226093	Up	0.00283656	0.060832515
15212	0	2	0.001	7.54	12.88034881	Up	0.224708	0.675748199
404335	0	1	0.001	7.52	12.87651695	Up	0.46568	0.811672241
93840	0	6	0.001	7.51	12.87459719	Up	0.01218244	0.156435314
66593	0	2	0.001	7.47	12.86689253	Up	0.224708	0.673314825
217695	0	4	0.001	7.42	12.85720347	Up	0.052321	0.353092491
66087	0	2	0.001	7.4	12.85330956	Up	0.224708	0.65172369
69131	0	5	0.001	7.39	12.85135865	Up	0.0252468	0.250690481
16679	0	2	0.001	7.39	12.85135865	Up	0.224708	0.673800097
17761	0	4	0.001	7.37	12.8474489	Up	0.052321	0.343346247
96979	0	2	0.001	7.35	12.84352853	Up	0.224708	0.667307376
21915	0	1	0.001	7.33	12.83959748	Up	0.46568	0.798294866

106583	0	5	0.001	7.32	12.83762793	Up	0.0252468	0.247734225
74596	0	4	0.001	7.28	12.82972274	Up	0.052321	0.343888658
56088	0	1	0.001	7.25	12.82376528	Up	0.46568	0.823225681
280668	0	3.11	0.001	7.19	12.81177606	Up	0.1084294	0.50376384
209047	0	1	0.001	7.19	12.81177606	Up	0.46568	0.767919794
12447	0	2	0.001	7.16	12.80574387	Up	0.224708	0.661406179
19170	0	1	0.001	7.11	12.79563384	Up	0.46568	0.792742079
74748	0	2	0.001	7.09	12.79156991	Up	0.224708	0.674042995
26465	0	2	0.001	7.08	12.78953364	Up	0.224708	0.674529317
234729	0	3	0.001	7.07	12.7874945	Up	0.1084294	0.516747444
20947	0	4	0.001	7.07	12.7874945	Up	0.052321	0.366467206
259300	0	3	0.001	7.06	12.78545247	Up	0.1084294	0.513220158
72691	0	2	0.001	7.06	12.78545247	Up	0.224708	0.647660294
68092	0	2	0.001	7.04	12.78135971	Up	0.224708	0.666118727
71177	0	3	0.001	7.03	12.77930897	Up	0.1084294	0.48119522
272347	0	3	0.001	7	12.77313921	Up	0.1084294	0.50830481
327957	0	1	0.001	6.98	12.76901132	Up	0.46568	0.823925852
26931	0	4	0.001	6.96	12.76487159	Up	0.052321	0.355108516
232987	0	1	0.001	6.96	12.76487159	Up	0.46568	0.768072008
272551	0	1	0.001	6.93	12.75863964	Up	0.46568	0.782812784
22349	0	3	0.001	6.92	12.75655632	Up	0.1084294	0.508878194
53619	0	2	0.001	6.89	12.75028827	Up	0.224708	0.672830251
319207	0	3	0.001	6.89	12.75028827	Up	0.1084294	0.493567307
24064	0	2	0.001	6.88	12.74819285	Up	0.224708	0.664697927
19201	0	3	0.001	6.87	12.74609438	Up	0.1084294	0.506024138
73024	0	1	0.001	6.86	12.74399286	Up	0.46568	0.811502258

71991	0	2	0.001	6.86	12.74399286	Up	0.224708	0.65469022
22630	0	2	0.001	6.85	12.74188827	Up	0.224708	0.64276221
66357	0	1	0.001	6.8	12.73131903	Up	0.46568	0.781549673
78894	0	3	0.001	6.79	12.72919586	Up	0.1084294	0.496828765
76524	0	2	0.001	6.77	12.72494012	Up	0.224708	0.644312635
22654	0	2	0.001	6.76	12.72280753	Up	0.224708	0.632328464
18000	0	3	0.001	6.76	12.72280753	Up	0.1084294	0.47940544
210148	0	2	0.001	6.71	12.71209705	Up	0.224708	0.695867238
14748	0	2	0.001	6.7	12.70994538	Up	0.224708	0.642983242
12757	0	1	0.001	6.62	12.6926155	Up	0.46568	0.797145295
67059	0	2	0.001	6.6	12.68825031	Up	0.224708	0.659074821
19698	0	2	0.001	6.59	12.68606275	Up	0.224708	0.682407032
208606	0	2	0.001	6.59	12.68606275	Up	0.224708	0.663518548
235497	0	2	0.001	6.59	12.68606275	Up	0.224708	0.661640222
66983	0	3	0.001	6.58	12.68387187	Up	0.1084294	0.511184724
71137	0	3	0.001	6.58	12.68387187	Up	0.1084294	0.49985653
19725	0	3	0.001	6.58	12.68387187	Up	0.1084294	0.483257117
109778	0	1.25	0.001	6.56	12.6794801	Up	0.46568	0.844761997
68992	0	1	0.001	6.56	12.6794801	Up	0.46568	0.838183708
23950	0	1	0.001	6.56	12.6794801	Up	0.46568	0.795019138
50817	0	3	0.001	6.56	12.6794801	Up	0.1084294	0.478135155
29815	0	3	0.001	6.5	12.666224	Up	0.1084294	0.517636855
66213	0	2	0.001	6.5	12.666224	Up	0.224708	0.658842589
594844	0	1	0.001	6.5	12.666224	Up	0.46568	0.770975583
69940	0	3	0.001	6.47	12.65955	Up	0.1084294	0.514390557
13480	0	2	0.001	6.47	12.65955	Up	0.224708	0.668022604

15018	0	1	0.001	6.47	12.65955	Up	0.46568	0.815601617
69714	0	1	0.001	6.46	12.65731845	Up	0.46568	0.770056296
229512	0	4	0.001	6.44	12.65284497	Up	0.052321	0.364320536
66329	0	1	0.001	6.42	12.64835758	Up	0.46568	0.778097044
21985	0	2	0.001	6.37	12.63707766	Up	0.224708	0.688944461
240672	0	1	0.001	6.35	12.63254088	Up	0.46568	0.83010353
56205	0	2	0.001	6.35	12.63254088	Up	0.224708	0.63425891
76499	0	2	0.001	6.34	12.63026713	Up	0.224708	0.657915295
20624	0	3	0.001	6.34	12.63026713	Up	0.1084294	0.477123764
114584	0	1	0.001	6.33	12.62798978	Up	0.46568	0.843474811
208677	0	2	0.001	6.33	12.62798978	Up	0.224708	0.674772742
330188	0	2	0.001	6.32	12.62570884	Up	0.224708	0.698205851
66225	0	1	0.001	6.31	12.62342429	Up	0.46568	0.803259386
320204	0	1	0.001	6.3	12.62113611	Up	0.46568	0.802095483
207259	0	4	0.001	6.24	12.60733031	Up	0.052321	0.340393308
63857	0	2	0.001	6.22	12.60269887	Up	0.224708	0.698727679
109857	0	1	0.001	6.22	12.60269887	Up	0.46568	0.768224282
72640	0	5	0.001	6.19	12.59572369	Up	0.0252468	0.254024937
72157	0	2	0.001	6.19	12.59572369	Up	0.224708	0.655608439
11488	0	4	0.001	6.17	12.59105477	Up	0.052321	0.36069846
67121	0	4	0.001	6.16	12.58871464	Up	0.052321	0.344160507
70807	0	2	0.001	6.15	12.5863707	Up	0.224708	0.697945229
22658	0	2	0.001	6.15	12.5863707	Up	0.224708	0.657683879
13875	0	3	0.001	6.15	12.5863707	Up	0.1084294	0.481451994
53895	0	1	0.001	6.07	12.5674808	Up	0.46568	0.809637125
77767	0	3	0.001	6.07	12.5674808	Up	0.1084294	0.48822567

100303744	0	3	0.001	6.07	12.5674808	Up	0.1084294	0.486121249
226245	0	2	0.001	6.05	12.56271943	Up	0.224708	0.691748157
66161	0	1	0.001	6.03	12.55794229	Up	0.46568	0.787586033
56310	0	1	0.001	6	12.55074679	Up	0.46568	0.804426672
230673	0	3	0.001	6	12.55074679	Up	0.1084294	0.482223964
14219	0	2	0.001	5.97	12.54351522	Up	0.224708	0.663989797
20725	0	1	0.001	5.95	12.53867395	Up	0.46568	0.846053118
13209	0	5	0.001	5.95	12.53867395	Up	0.0252468	0.242585015
14126	0	2	0.001	5.94	12.53624722	Up	0.224708	0.685157665
30925	0	2	0.001	5.91	12.52894242	Up	0.224708	0.680915975
13006	0	4	0.001	5.9	12.52649924	Up	0.052321	0.347456537
76787	0	4	0.001	5.87	12.51914479	Up	0.052321	0.358028817
69942	0	1	0.001	5.87	12.51914479	Up	0.46568	0.813034679
105180375	0	1	0.001	5.85	12.51422091	Up	0.46568	0.821828903
269713	0	4	0.001	5.84	12.51175265	Up	0.052321	0.349128341
73614	0	1	0.001	5.84	12.51175265	Up	0.46568	0.790639314
21419	0	5	0.001	5.83	12.50928017	Up	0.0252468	0.250989991
224613	0	2	0.001	5.75	12.48934624	Up	0.224708	0.663047967
19184	0	1	0.001	5.73	12.48431942	Up	0.46568	0.789511671
71653	0	3	0.001	5.71	12.47927503	Up	0.1084294	0.516451653
19038	0	1	0.001	5.69	12.47421294	Up	0.46568	0.826562133
76808	0	1	0.001	5.66	12.46658634	Up	0.46568	0.820263184
228361	0	4	0.001	5.66	12.46658634	Up	0.052321	0.355979592
70285	0	1	0.001	5.65	12.46403515	Up	0.46568	0.843658454
74914	0	1	0.001	5.64	12.46147945	Up	0.46568	0.783604303

110606	0	2	0.001	5.63	12.45891921	Up	0.224708	0.674286069
386655	0	1	0.001	5.63	12.45891921	Up	0.46568	0.816805076
11475	0	2	0.001	5.61	12.45378506	Up	0.224708	0.695349672
12297	0	2	0.001	5.61	12.45378506	Up	0.224708	0.693801584
20656	0	2.99	0.001	5.6	12.45121111	Up	0.224708	0.636417722
74347	0	2	0.001	5.6	12.45121111	Up	0.224708	0.635768537
264064	0	2	0.001	5.57	12.44346161	Up	0.224708	0.687677554
171281	0	2	0.001	5.56	12.44086917	Up	0.224708	0.630409733
107932	0	5	0.001	5.55	12.43827206	Up	0.0252468	0.253718143
225579	0	2	0.001	5.55	12.43827206	Up	0.224708	0.676971495
271944	0	1	0.001	5.55	12.43827206	Up	0.46568	0.82515402
233204	0	2	0.001	5.54	12.43567026	Up	0.224708	0.688437138
225518	0	2	0.001	5.54	12.43567026	Up	0.224708	0.680668099
58207	0	2	0.001	5.54	12.43567026	Up	0.224708	0.653546057
21846	0	3	0.001	5.54	12.43567026	Up	0.1084294	0.484815173
269614	0	2	0.001	5.53	12.43306377	Up	0.224708	0.687424731
22195	0	2	0.001	5.52	12.43045255	Up	0.224708	0.670898912
20182	0	2	0.001	5.5	12.4252159	Up	0.224708	0.686919643
74781	0	3	0.001	5.5	12.4252159	Up	0.1084294	0.501802579
20810	0	1	0.001	5.5	12.4252159	Up	0.46568	0.797966079
170639	0	2	0.001	5.49	12.42259043	Up	0.224708	0.665407569
69225	0	1	0.001	5.49	12.42259043	Up	0.46568	0.785669765
55927	0	1	0.001	5.47	12.41732512	Up	0.46568	0.809975602
73720	0	3	0.001	5.45	12.41204051	Up	0.1084294	0.478896517
17920	0	6	0.001	5.44	12.40939094	Up	0.01218244	0.161674774
14827	0	2	0.001	5.42	12.40407714	Up	0.224708	0.682158069

70450	0	3	0.001	5.42	12.40407714	Up	0.1084294	0.500411002
17082	0	4	0.001	5.39	12.39606956	Up	0.052321	0.359507053
170742	0	1	0.001	5.38	12.39339046	Up	0.46568	0.822352139
230752	0	1	0.001	5.36	12.38801729	Up	0.46568	0.784715124
219132	0	2	0.001	5.34	12.38262403	Up	0.224708	0.671380707
240892	0	3	0.001	5.33	12.37991982	Up	0.1084294	0.496009366
381582	0	1	0.001	5.3	12.37177664	Up	0.46568	0.818012092
14349	0	1	0.001	5.29	12.36905201	Up	0.46568	0.853695369
74776	0	1	0.001	5.29	12.36905201	Up	0.46568	0.848275674
21897	0	2	0.001	5.29	12.36905201	Up	0.224708	0.679925552
20423	0	2	0.001	5.28	12.36632221	Up	0.224708	0.629560696
76251	0	2	0.001	5.27	12.36358725	Up	0.224708	0.682656177
217995	0	5	0.001	5.27	12.36358725	Up	0.0252468	0.250391684
433619	0	2	0.001	5.26	12.36084708	Up	0.224708	0.628291421
22368	0	2	0.001	5.24	12.3553511	Up	0.224708	0.630197259
22044	0	1	0.001	5.21	12.34706766	Up	0.46568	0.838365054
230126	0	2	0.001	5.21	12.34706766	Up	0.224708	0.645870559
22375	0	2	0.001	5.21	12.34706766	Up	0.224708	0.637720078
18563	0	3	0.001	5.21	12.34706766	Up	0.1084294	0.47864246
19724	0	3	0.001	5.15	12.33035672	Up	0.1084294	0.502921426
68145	0	3	0.001	5.13	12.32474311	Up	0.1084294	0.511764627
65963	0	1	0.001	5.13	12.32474311	Up	0.46568	0.83045934
20404	0	2	0.001	5.11	12.31910758	Up	0.224708	0.686415297
98952	0	3	0.001	5.1	12.31628153	Up	0.1084294	0.500133613
72238	0	4	0.001	5.09	12.31344994	Up	0.052321	0.363408215
67529	0	2	0.001	5.09	12.31344994	Up	0.224708	0.636634412

21427	0	1	0.001	5.08	12.31061278	Up	0.46568	0.846422735
66818	0	2	0.001	5.07	12.30777003	Up	0.224708	0.69405912
67488	0	2	0.001	5.07	12.30777003	Up	0.224708	0.6777076
13139	0	2	0.001	5.07	12.30777003	Up	0.224708	0.660938589
213417	0	3	0.001	5.04	12.29920802	Up	0.1084294	0.494649692
235086	0	2	0.001	5.04	12.29920802	Up	0.224708	0.630835111
233887	0	1.86	0.001	5.03	12.29634268	Up	0.46568	0.84091217
228491	0	3	0.001	5.03	12.29634268	Up	0.1084294	0.482739988
231510	0	2	0.001	5	12.28771238	Up	0.224708	0.655378643
17215	0	2	0.001	4.99	12.2848241	Up	0.224708	0.676236987
56442	0	2	0.001	4.98	12.28193003	Up	0.224708	0.660238442
22114	0	1	0.001	4.97	12.27903014	Up	0.46568	0.767159628
52118	0	2	0.001	4.96	12.27612441	Up	0.224708	0.696904684
66827	0	1	0.001	4.96	12.27612441	Up	0.46568	0.771897068
269513	0	2	0.001	4.95	12.27321281	Up	0.224708	0.643647252
209027	0	2	0.001	4.94	12.27029533	Up	0.224708	0.697424568
54645	0	2	0.001	4.92	12.2644426	Up	0.224708	0.6423206
14269	0	3	0.001	4.89	12.25561875	Up	0.1084294	0.490082041
11652	0	2	0.001	4.87	12.24970606	Up	0.224708	0.658610521
76425	0	3	0.001	4.87	12.24970606	Up	0.1084294	0.487697858
243867	0	2	0.001	4.86	12.2467406	Up	0.224708	0.640340845
66365	0	1	0.001	4.81	12.23182118	Up	0.46568	0.845499297
18744	0	2	0.001	4.79	12.22580994	Up	0.224708	0.677953324
12169	0	2	0.001	4.79	12.22580994	Up	0.224708	0.635336483
27055	0	2	0.001	4.78	12.2227949	Up	0.224708	0.683404703
52653	0	1	0.001	4.78	12.2227949	Up	0.46568	0.805095217

108934	0	3	0.001	4.78	12.2227949	Up	0.1084294	0.478388673
226122	0	1	0.001	4.77	12.21977355	Up	0.46568	0.807780546
13175	0	4	0.001	4.75	12.2137118	Up	0.052321	0.358323491
231386	0	2	0.001	4.74	12.21067134	Up	0.224708	0.688183757
57312	0	1	0.001	4.74	12.21067134	Up	0.46568	0.8159451
15201	0	2	0.001	4.73	12.20762447	Up	0.224708	0.649009118
16911	0	1	0.001	4.7	12.19844504	Up	0.46568	0.832242973
56223	0	1.29	0.001	4.69	12.19537221	Up	0.46568	0.836735755
76681	0	1	0.001	4.69	12.19537221	Up	0.46568	0.808623389
224893	0	1.34	0.001	4.69	12.19537221	Up	0.46568	0.803925992
69024	0	1	0.001	4.68	12.19229281	Up	0.46568	0.846237886
331401	0	5	0.001	4.68	12.19229281	Up	0.0252468	0.249796222
16564	0	4	0.001	4.67	12.18920683	Up	0.052321	0.339861859
225049	0	3	0.001	4.66	12.18611424	Up	0.1084294	0.481709043
94112	0	2	0.001	4.63	12.17679648	Up	0.224708	0.685660164
13043	0	2	0.001	4.62	12.17367714	Up	0.224708	0.693287085
217143	0	6	0.001	4.61	12.17055104	Up	0.01218244	0.16193304
66355	0	1	0.001	4.59	12.16427844	Up	0.46568	0.785192154
22228	0	1	0.001	4.59	12.16427844	Up	0.46568	0.773283432
69888	0	1.03	0.001	4.58	12.16113188	Up	0.46568	0.77066891
338369	0	1	0.001	4.57	12.15797845	Up	0.46568	0.812352889
230700	0	3	0.001	4.57	12.15797845	Up	0.1084294	0.49034839
228869	0	2	0.001	4.56	12.15481811	Up	0.224708	0.656068515
56066	0	1	0.001	4.55	12.15165083	Up	0.46568	0.812693641
57439	0	2	0.001	4.54	12.14847658	Up	0.224708	0.686163401
242642	0	1	0.001	4.54	12.14847658	Up	0.46568	0.800108049

14415	0	1	0.001	4.53	12.14529533	Up	0.46568	0.793066574
74467	0	2	0.001	4.53	12.14529533	Up	0.224708	0.63062235
20163	0	1	0.001	4.52	12.14210706	Up	0.46568	0.850509939
217674	0	1	0.001	4.5	12.13570929	Up	0.46568	0.798788555
242409	0	3	0.001	4.49	12.13249973	Up	0.1084294	0.482998414
100041585	0	2	0.001	4.49	12.13249973	Up	0.224708	0.629984929
19014	0	4	0.001	4.48	12.12928302	Up	0.052321	0.351099227
56356	0	1	0.001	4.48	12.12928302	Up	0.46568	0.781392071
67525	0	2	0.001	4.47	12.12605912	Up	0.224708	0.699773678
233826	0	2	0.001	4.42	12.10983065	Up	0.224708	0.692260373
212541	0	2	0.001	4.42	12.10983065	Up	0.224708	0.63859128
242705	0	3	0.001	4.41	12.10656294	Up	0.1084294	0.497650876
12905	0	1	0.001	4.4	12.10328781	Up	0.46568	0.778409658
56438	0	1	0.001	4.39	12.10000522	Up	0.46568	0.822526699
30960	0	1	0.001	4.38	12.09671515	Up	0.46568	0.843107763
68278	0	1	0.001	4.38	12.09671515	Up	0.46568	0.795508783
224902	0	2	0.001	4.36	12.09011242	Up	0.224708	0.668261354
83704	0	2	0.001	4.34	12.08347933	Up	0.224708	0.631261063
171580	0	2	0.001	4.3	12.07012094	Up	0.224708	0.696385575
52202	0	2.06	0.001	4.3	12.07012094	Up	0.224708	0.641879598
20342	0	1	0.001	4.28	12.06339508	Up	0.46568	0.765039147
20020	0	4	0.001	4.24	12.04984855	Up	0.052321	0.36189779
74551	0	2	0.001	4.23	12.04644195	Up	0.224708	0.663754089
12402	0	3	0.001	4.22	12.04302728	Up	0.1084294	0.505174153
16665	0	1	0.001	4.22	12.04302728	Up	0.46568	0.776071156
237339	0	2	0.001	4.21	12.03960452	Up	0.224708	0.648109278

11666	0	2	0.001	4.2	12.03617361	Up	0.224708	0.692516766
70397	0	1	0.001	4.2	12.03617361	Up	0.46568	0.787266006
20148	0	1	0.001	4.19	12.03273453	Up	0.46568	0.834932833
13805	0	2	0.001	4.19	12.03273453	Up	0.224708	0.655149008
75305	0	1	0.001	4.18	12.02928723	Up	0.46568	0.839272965
406218	0	2	0.001	4.18	12.02928723	Up	0.224708	0.681164032
20846	0	3	0.001	4.17	12.02583167	Up	0.1084294	0.510028851
237253	0	2	0.001	4.17	12.02583167	Up	0.224708	0.662108806
98766	0	2	0.001	4.16	12.02236781	Up	0.224708	0.669697446
18667	0	4	0.001	4.15	12.01889562	Up	0.052321	0.344705496
260302	0	2	0.001	4.14	12.01541505	Up	0.224708	0.692773349
13142	0	1	0.001	4.14	12.01541505	Up	0.46568	0.785351293
67888	0	1	0.001	4.11	12.00492268	Up	0.46568	0.833675404
101685	0	3	0.001	4.1	12.00140819	Up	0.1084294	0.498475711
72949	0	2	0.001	4.09	11.99788513	Up	0.224708	0.666356118
226153	0	2	0.001	4.09	11.99788513	Up	0.224708	0.635120675
235386	0	2.91	0.001	4.08	11.99435344	Up	0.224708	0.658146874
74178	0	2	0.001	4.08	11.99435344	Up	0.224708	0.635552436
244864	0	1	0.001	4.06	11.98726401	Up	0.46568	0.826914913
100502698	0	3	0.001	4.04	11.98013958	Up	0.1084294	0.514097457
20469	0	2	0.001	4.04	11.98013958	Up	0.224708	0.656759841
226856	0	4	0.001	4.04	11.98013958	Up	0.052321	0.343075682
74414	0	1	0.001	4.04	11.98013958	Up	0.46568	0.764133954
545812	0	1	0.001	4.01	11.96938652	Up	0.46568	0.765795115
102122	0	1	0.001	4	11.96578428	Up	0.46568	0.817494363
52639	0	1	0.001	3.99	11.96217303	Up	0.46568	0.838546479

245684	0	3	0.001	3.99	11.96217303	Up	0.1084294	0.491684489
74464	0	3	0.001	3.98	11.95855272	Up	0.1084294	0.512055072
117586	0	1	0.001	3.98	11.95855272	Up	0.46568	0.818184814
71998	0	1	0.001	3.97	11.95492329	Up	0.46568	0.776849094
19357	0	2	0.001	3.95	11.94763694	Up	0.224708	0.665644453
23961	0	1	0.001	3.95	11.94763694	Up	0.46568	0.792904293
12051	0	1	0.001	3.95	11.94763694	Up	0.46568	0.763531681
68942	0	1	0.001	3.94	11.94397991	Up	0.46568	0.846792675
56361	0	1	0.001	3.94	11.94397991	Up	0.46568	0.789833526
237459	0	2	0.001	3.94	11.94397991	Up	0.224708	0.634474132
77041	0	2	0.001	3.93	11.9403136	Up	0.224708	0.698466667
14854	0	1	0.001	3.93	11.9403136	Up	0.46568	0.774984656
71908	0	1	0.001	3.92	11.93663794	Up	0.46568	0.826385856
78246	0	1	0.001	3.92	11.93663794	Up	0.46568	0.801431909
230103	0	2	0.001	3.92	11.93663794	Up	0.224708	0.631901071
66875	0	2	0.001	3.91	11.93295289	Up	0.224708	0.697684801
241289	0	3	0.001	3.91	11.93295289	Up	0.1084294	0.515271866
16449	0	3	0.001	3.91	11.93295289	Up	0.1084294	0.493837459
80708	0	1	0.001	3.9	11.92925841	Up	0.46568	0.850883461
68939	0	1	0.001	3.9	11.92925841	Up	0.46568	0.796653635
319476	0	4	0.001	3.9	11.92925841	Up	0.052321	0.341997676
17977	0	4	0.001	3.89	11.92555444	Up	0.052321	0.363711814
72046	0	3	0.001	3.88	11.92184094	Up	0.1084294	0.502361379
140492	0	2	0.001	3.87	11.91811785	Up	0.224708	0.628502611
269152	0	4	0.001	3.85	11.91064273	Up	0.052321	0.36677594
70152	0	1	0.001	3.85	11.91064273	Up	0.46568	0.848833139

212442	0	1	0.001	3.84	11.9068906	Up		0.46568	0.802926498
18641	0	2	0.001	3.84	11.9068906	Up		0.224708	0.652633601
11520	0	1	0.001	3.84	11.9068906	Up		0.46568	0.77950579
18291	0	1	0.001	3.83	11.90312868	Up		0.46568	0.810144947
22221	0	2	0.001	3.83	11.90312868	Up		0.224708	0.645201956
107513	0	5	0.001	3.83	11.90312868	Up		0.0252468	0.24286546
71059	0	1	0.001	3.82	11.89935692	Up		0.46568	0.833496081
83410	0	2	0.001	3.81	11.89557528	Up		0.224708	0.671863194
68671	0	1	0.001	3.8	11.8917837	Up		0.46568	0.768833984
50887	0	1	0.001	3.79	11.88798213	Up		0.46568	0.847162938
209448	0	1	0.001	3.78	11.88417052	Up		0.46568	0.78790632
12296	0	2	0.001	3.76	11.87651695	Up		0.224708	0.646540549
110460	0	1.08	0.001	3.75	11.87267488	Up		0.46568	0.82165464
66586	0	1	0.001	3.74	11.86882255	Up		0.46568	0.854636806
665622	0	1.1	0.001	3.74	11.86882255	Up		0.46568	0.842374626
17698	0	2	0.001	3.74	11.86882255	Up		0.224708	0.662578054
665596	0	1.1	0.001	3.74	11.86882255	Up		0.46568	0.802427683
329207	0	2	0.001	3.73	11.86495992	Up		0.224708	0.650589864
72313	0	6	0.001	3.72	11.86108691	Up		0.01218244	0.156919633
72431	0	1	0.001	3.71	11.85720347	Up		0.46568	0.84586843
16777	0	3	0.001	3.71	11.85720347	Up		0.1084294	0.501523645
110816	0	2	0.001	3.71	11.85720347	Up		0.224708	0.650816313
12399	0	2	0.001	3.69	11.8494051	Up		0.224708	0.659772501
329178	0	7	0.001	3.69	11.8494051	Up		0.00587846	0.102761903
68051	0	1	0.001	3.68	11.84549005	Up		0.46568	0.84995027
13435	0	5	0.001	3.68	11.84549005	Up		0.0252468	0.251290219

279766	0	1	0.001	3.68	11.84549005	Up	0.46568	0.823575617
242891	0	1	0.001	3.68	11.84549005	Up	0.46568	0.782180719
268567	0	2	0.001	3.67	11.84156435	Up	0.224708	0.680172888
14423	0	2	0.001	3.67	11.84156435	Up	0.224708	0.632756436
22409	0	1	0.001	3.66	11.83762793	Up	0.46568	0.85163149
16773	0	5	0.001	3.66	11.83762793	Up	0.0252468	0.248319885
64296	0	1	0.001	3.65	11.83368075	Up	0.46568	0.828683336
18693	0	1	0.001	3.65	11.83368075	Up	0.46568	0.791446748
78816	0	2	0.001	3.64	11.82972274	Up	0.224708	0.672588226
18186	0	3	0.001	3.62	11.82177398	Up	0.1084294	0.506876987
20684	0	1	0.001	3.62	11.82177398	Up	0.46568	0.816632936
74053	0	2	0.001	3.62	11.82177398	Up	0.224708	0.654003242
23859	0	3	0.001	3.62	11.82177398	Up	0.1084294	0.490881957
19302	0	1	0.001	3.62	11.82177398	Up	0.46568	0.792255833
333193	0	1.31	0.001	3.62	11.82177398	Up	0.46568	0.764586283
16172	0	2	0.001	3.61	11.81778312	Up	0.224708	0.657221535
98710	0	1	0.001	3.61	11.81778312	Up	0.46568	0.774365164
19328	0	1	0.001	3.6	11.81378119	Up	0.46568	0.851257311
21677	0	1	0.001	3.6	11.81378119	Up	0.46568	0.825505599
66720	0	1	0.001	3.6	11.81378119	Up	0.46568	0.789190077
24061	0	2	0.001	3.6	11.81378119	Up	0.224708	0.640560215
22333	0	1	0.001	3.55	11.79360331	Up	0.46568	0.803092908
16404	0	2	0.001	3.55	11.79360331	Up	0.224708	0.648783924
213121	0	2	0.001	3.54	11.78953364	Up	0.224708	0.684405296
71750	0	2	0.001	3.54	11.78953364	Up	0.224708	0.656298795
22021	0	1	0.001	3.52	11.78135971	Up	0.46568	0.817666866

71791	0	1	0.001	3.51	11.77725532	Up	0.46568	0.768986561
53867	0	3	0.001	3.5	11.77313921	Up	0.1084294	0.510895265
66930	0	1	0.001	3.49	11.76901132	Up	0.46568	0.829748026
74468	0	1	0.001	3.49	11.76901132	Up	0.46568	0.819569222
78919	0	1	0.001	3.48	11.76487159	Up	0.46568	0.831885633
57230	0	1	0.001	3.47	11.76071995	Up	0.46568	0.806435646
226413	0	3	0.001	3.47	11.76071995	Up	0.1084294	0.488490004
20583	0	1	0.001	3.47	11.76071995	Up	0.46568	0.785510497
234825	0	1	0.001	3.47	11.76071995	Up	0.46568	0.765190221
71521	0	2	0.001	3.46	11.75655632	Up	0.224708	0.641659323
19243	0	2	0.001	3.45	11.75238065	Up	0.224708	0.681909288
18119	0	1	0.001	3.45	11.75238065	Up	0.46568	0.777316606
103284	0	1	0.001	3.44	11.74819285	Up	0.46568	0.852380836
72297	0	1	0.001	3.44	11.74819285	Up	0.46568	0.846607664
108900	0	1	0.001	3.44	11.74819285	Up	0.46568	0.81218262
14281	0	1	0.001	3.43	11.74399286	Up	0.46568	0.841825609
14919	0	4	0.001	3.43	11.74399286	Up	0.052321	0.360997546
56315	0	1	0.001	3.43	11.74399286	Up	0.46568	0.826033528
217410	0	2	0.001	3.42	11.73978061	Up	0.224708	0.659539777
51960	0	1	0.001	3.42	11.73978061	Up	0.46568	0.781077057
328370	0	1.13	0.001	3.41	11.73555602	Up	0.46568	0.841094699
56249	0	1	0.001	3.41	11.73555602	Up	0.46568	0.822701333
76479	0	1	0.001	3.4	11.73131903	Up	0.46568	0.849019124
30948	0	1	0.001	3.39	11.72706956	Up	0.46568	0.770362481
433375	0	1	0.001	3.38	11.72280753	Up	0.46568	0.821132291
26556	0	1	0.001	3.38	11.72280753	Up	0.46568	0.77856606

14815	0	3	0.001	3.38	11.72280753	Up	0.1084294	0.483775355
219148	0	2	0.001	3.37	11.71853288	Up	0.224708	0.677216685
20649	0	1	0.001	3.36	11.71424552	Up	0.46568	0.763080599
30945	0	2	0.001	3.35	11.70994538	Up	0.224708	0.694832876
232879	0	1	0.001	3.34	11.70563239	Up	0.46568	0.780919645
69863	0	4.07	0.001	3.32	11.69696753	Up	0.052321	0.356562687
16523	0	1	0.001	3.3	11.68825031	Up	0.46568	0.847904438
233060	0	1	0.001	3.3	11.68825031	Up	0.46568	0.797637563
102442	0	4	0.001	3.3	11.68825031	Up	0.052321	0.346350868
20500	0	1	0.001	3.3	11.68825031	Up	0.46568	0.763983297
56353	0	2	0.001	3.28	11.6794801	Up	0.224708	0.689452532
78558	0	1	0.001	3.27	11.67507492	Up	0.46568	0.81904952
230145	0	1	0.001	3.26	11.67065625	Up	0.46568	0.804092816
15944	0	1.01	0.001	3.26	11.67065625	Up	0.46568	0.8015977
27403	0	3	0.001	3.24	11.6617781	Up	0.1084294	0.515861085
228536	0	2	0.001	3.24	11.6617781	Up	0.224708	0.661874431
27399	0	2	0.001	3.24	11.6617781	Up	0.224708	0.660471659
28035	0	1	0.001	3.23	11.65731845	Up	0.46568	0.833316834
69675	0	3	0.001	3.23	11.65731845	Up	0.1084294	0.499579755
68760	0	2	0.001	3.23	11.65731845	Up	0.224708	0.664461716
15111	0	1	0.001	3.23	11.65731845	Up	0.46568	0.800603983
15251	0	2	0.001	3.23	11.65731845	Up	0.224708	0.648334004
319924	0	3	0.001	3.22	11.65284497	Up	0.1084294	0.504045272
229543	0	2	0.001	3.21	11.64835758	Up	0.224708	0.68141227
210530	0	1	0.001	3.21	11.64835758	Up	0.46568	0.801266187
17113	0	1	0.001	3.2	11.64385619	Up	0.46568	0.779976506

21337	0	1	0.001	3.2	11.64385619	Up	0.46568	0.771589661
67943	0	2	0.001	3.19	11.63934071	Up	0.224708	0.691492333
192289	0	1	0.001	3.19	11.63934071	Up	0.46568	0.762780173
100169	0	2	0.001	3.18	11.63481105	Up	0.224708	0.679678396
13190	0	1	0.001	3.17	11.63026713	Up	0.46568	0.842924359
74761	0	1	0.001	3.17	11.63026713	Up	0.46568	0.765946487
14595	0	2	0.001	3.16	11.62570884	Up	0.224708	0.649459975
320790	0	5	0.001	3.15	11.62113611	Up	0.0252468	0.255259566
171543	0	2	0.001	3.15	11.62113611	Up	0.224708	0.686667377
70652	0	1	0.001	3.15	11.62113611	Up	0.46568	0.788226867
75552	0	1	0.001	3.13	11.61194694	Up	0.46568	0.794041656
229317	0	1	0.001	3.11	11.60269887	Up	0.46568	0.806100121
230674	0	2	0.001	3.1	11.5980525	Up	0.224708	0.673557373
22041	0	1	0.001	3.1	11.5980525	Up	0.46568	0.806603514
70454	0	1	0.001	3.1	11.5980525	Up	0.46568	0.777940831
70839	0	1	0.001	3.09	11.59339112	Up	0.46568	0.785988495
108737	0	2	0.001	3.08	11.58871464	Up	0.224708	0.690725995
353499	0	1	0.001	3.08	11.58871464	Up	0.46568	0.784238672
71810	0	1	0.001	3.07	11.58402294	Up	0.46568	0.839454783
19153	0	2	0.001	3.07	11.58402294	Up	0.224708	0.684906692
81799	0	1	0.001	3.07	11.58402294	Up	0.46568	0.821480449
14585	0	2	0.001	3.07	11.58402294	Up	0.224708	0.667069307
11287	0	2	0.001	3.06	11.57931594	Up	0.224708	0.678199227
100201	0	2	0.001	3.06	11.57931594	Up	0.224708	0.668978629
17309	0	2	0.001	3.06	11.57931594	Up	0.224708	0.649911459
272411	0	1	0.001	3.05	11.57459353	Up	0.46568	0.841459996

74094	0	1	0.001	3.05	11.57459353	Up	0.46568	0.765643802
320590	0	1	0.001	3.03	11.56510208	Up	0.46568	0.808792169
16400	0	2	0.001	3.03	11.56510208	Up	0.224708	0.632542378
21339	0	1	0.001	3.02	11.56033283	Up	0.46568	0.783287504
98970	0	2	0.001	3.01	11.55554777	Up	0.224708	0.669457668
109552	0	1	0.001	3.01	11.55554777	Up	0.46568	0.814230569
67972	0	3	0.001	3.01	11.55554777	Up	0.1084294	0.488754625
66658	0	1	0.001	3.01	11.55554777	Up	0.46568	0.782022862
12983	0	2	0.001	3	11.55074679	Up	0.224708	0.697164529
57278	0	1	0.001	3	11.55074679	Up	0.46568	0.8256815
18552	0	3	0.001	2.98	11.54109662	Up	0.1084294	0.513804691
242274	0	3	0.001	2.98	11.54109662	Up	0.1084294	0.49492103
54721	0	2	0.001	2.97	11.53624722	Up	0.224708	0.629137035
338359	0	1	0.001	2.96	11.53138146	Up	0.46568	0.84920519
56707	0	2.62	0.001	2.96	11.53138146	Up	0.224708	0.687172094
18436	0	1	0.001	2.96	11.53138146	Up	0.46568	0.771743334
108707	0	1	0.001	2.95	11.52649924	Up	0.46568	0.833137665
666168	0	1	0.001	2.95	11.52649924	Up	0.46568	0.812864124
171188	0	1	0.001	2.95	11.52649924	Up	0.46568	0.794693043
70809	0	1	0.001	2.94	11.52160044	Up	0.46568	0.820610606
235636	0	1	0.001	2.93	11.51668495	Up	0.46568	0.835653069
78911	0	1	0.001	2.93	11.51668495	Up	0.46568	0.805429906
544922	0	1	0.001	2.92	11.51175265	Up	0.46568	0.849391337
13347	0	1	0.001	2.92	11.51175265	Up	0.46568	0.794367216
18074	0	2	0.001	2.91	11.50680344	Up	0.224708	0.663283174
67015	0	1	0.001	2.91	11.50680344	Up	0.46568	0.779035641

29875	0	3	0.001	2.9	11.50183718	Up	0.1084294	0.481966366
13392	0	1	0.001	2.89	11.49685378	Up	0.46568	0.806939459
26410	0	1	0.001	2.88	11.4918531	Up	0.46568	0.787425987
381921	0	2	0.001	2.87	11.48683502	Up	0.224708	0.695091178
14050	0	2	0.001	2.87	11.48683502	Up	0.224708	0.671139723
12452	0	1	0.001	2.87	11.48683502	Up	0.46568	0.793716362
19276	0	1.88	0.001	2.87	11.48683502	Up	0.46568	0.777004869
72160	0	1	0.001	2.86	11.48179943	Up	0.46568	0.841642763
26927	0	1	0.001	2.86	11.48179943	Up	0.46568	0.839091226
18988	0	1	0.001	2.86	11.48179943	Up	0.46568	0.836013653
71665	0	1	0.001	2.86	11.48179943	Up	0.46568	0.791608433
16516	0	2	0.001	2.85	11.4767462	Up	0.224708	0.651496609
21336	0	2	0.001	2.84	11.47167521	Up	0.224708	0.63361412
223649	0	1	0.001	2.84	11.47167521	Up	0.46568	0.771129011
70291	0	1	0.001	2.84	11.47167521	Up	0.46568	0.770822216
53324	0	1	0.001	2.83	11.46658634	Up	0.46568	0.827621375
234129	0	1	0.001	2.83	11.46658634	Up	0.46568	0.777628593
73225	0	1	0.001	2.82	11.46147945	Up	0.46568	0.85350733
78772	0	1	0.001	2.82	11.46147945	Up	0.46568	0.848647236
108083	0	2	0.001	2.82	11.46147945	Up	0.224708	0.670177515
241846	0	1	0.001	2.82	11.46147945	Up	0.46568	0.800273292
13798	0	1	0.001	2.82	11.46147945	Up	0.46568	0.79599903
64297	0	1	0.001	2.82	11.46147945	Up	0.46568	0.775294774
77065	0	2	0.001	2.81	11.45635442	Up	0.224708	0.694316847
110253	0	1	0.001	2.81	11.45635442	Up	0.46568	0.837459105
214150	0	3	0.001	2.81	11.45635442	Up	0.1084294	0.505457164

72354	0	1	0.001	2.81	11.45635442	Up		0.46568	0.784556242
67149	0	1	0.001	2.8	11.45121111	Up		0.46568	0.841277308
242773	0	1	0.001	2.8	11.45121111	Up		0.46568	0.814744172
19885	0	1	0.001	2.8	11.45121111	Up		0.46568	0.807443901
56541	0	1	0.001	2.79	11.44604941	Up		0.46568	0.852005998
20610	0	1	0.001	2.79	11.44604941	Up		0.46568	0.827091415
15395	0	1	0.001	2.79	11.44604941	Up		0.46568	0.823400612
68614	0	1	0.001	2.79	11.44604941	Up		0.46568	0.814915516
232156	0	2	0.001	2.78	11.44086917	Up		0.224708	0.680420403
228889	0	1	0.001	2.78	11.44086917	Up		0.46568	0.799612728
11499	0	1	0.001	2.78	11.44086917	Up		0.46568	0.776226619
218503	0	2	0.001	2.78	11.44086917	Up		0.224708	0.636851249
243277	0	2	0.001	2.77	11.43567026	Up		0.224708	0.651269686
329738	0	1	0.001	2.77	11.43567026	Up		0.46568	0.76776764
29810	0	1	0.001	2.76	11.43045255	Up		0.46568	0.815773322
13723	0	1	0.001	2.75	11.4252159	Up		0.46568	0.814401698
208104	0	1.01	0.001	2.75	11.4252159	Up		0.46568	0.77482969
72421	0	1	0.001	2.74	11.41996018	Up		0.46568	0.851070345
67956	0	1	0.001	2.74	11.41996018	Up		0.46568	0.849763877
18602	0	1	0.001	2.73	11.41468524	Up		0.46568	0.81611695
11749	0	1	0.001	2.73	11.41468524	Up		0.46568	0.783762799
67683	0	1	0.001	2.72	11.40939094	Up		0.46568	0.789672566
22685	0	1	0.001	2.72	11.40939094	Up		0.46568	0.788868746
320360	0	2	0.001	2.71	11.40407714	Up		0.224708	0.631687591
71760	0	1	0.001	2.7	11.39874369	Up		0.46568	0.768376617
98685	0	1	0.001	2.69	11.39339046	Up		0.46568	0.846977766

240066	0	2	0.001	2.69	11.39339046	Up	0.224708	0.690981252
216363	0	1	0.001	2.68	11.38801729	Up	0.46568	0.772820758
27060	0	1	0.001	2.67	11.38262403	Up	0.46568	0.780762297
73658	0	1	0.001	2.66	11.37721053	Up	0.46568	0.816460868
192654	0	1	0.001	2.65	11.37177664	Up	0.46568	0.832958573
67978	0	1	0.001	2.63	11.36084708	Up	0.46568	0.847533526
15139	0	2	0.001	2.63	11.36084708	Up	0.224708	0.668500275
54130	0	1	0.001	2.63	11.36084708	Up	0.46568	0.798130439
11982	0	2	0.001	2.62	11.3553511	Up	0.224708	0.639902556
244721	0	1	0.001	2.61	11.34983409	Up	0.46568	0.845130486
244183	0	1	0.001	2.6	11.34429591	Up	0.46568	0.850696659
99151	0	0.98	0.001	2.6	11.34429591	Up	0.96507	0.97043474
226154	0	1	0.001	2.6	11.34429591	Up	0.46568	0.774055789
22785	0	2	0.001	2.6	11.34429591	Up	0.224708	0.632970639
239038	0	1	0.001	2.59	11.33873638	Up	0.46568	0.842741035
211232	0	1	0.001	2.59	11.33873638	Up	0.46568	0.782496624
14404	0	1	0.001	2.58	11.33315535	Up	0.46568	0.788547676
66641	0	1	0.001	2.57	11.32755264	Up	0.46568	0.822876042
270084	0	1	0.001	2.57	11.32755264	Up	0.46568	0.804927977
56338	0	1	0.001	2.57	11.32755264	Up	0.46568	0.803425934
21822	0	1	0.001	2.56	11.32192809	Up	0.46568	0.842008535
218165	0	1	0.001	2.56	11.32192809	Up	0.46568	0.83727815
19058	0	1	0.001	2.54	11.31061278	Up	0.46568	0.8313502
229320	0	1	0.001	2.53	11.30492167	Up	0.46568	0.840547349
433926	0	1	0.001	2.53	11.30492167	Up	0.46568	0.835112776
108907	0	1	0.001	2.52	11.29920802	Up	0.46568	0.854448353

67466	0	1	0.001	2.52	11.29920802	Up	0.46568	0.851818703
20588	0	2	0.001	2.52	11.29920802	Up	0.224708	0.640121625
100073351	0	1	0.001	2.51	11.29347165	Up	0.46568	0.830993626
330401	0	2	0.001	2.5	11.28771238	Up	0.224708	0.658378615
102162	0	1	0.001	2.49	11.28193003	Up	0.46568	0.77343778
75956	0	3	0.001	2.49	11.28193003	Up	0.1084294	0.480938719
14151	0	1	0.001	2.48	11.27612441	Up	0.46568	0.844393829
105847	0	1	0.001	2.48	11.27612441	Up	0.46568	0.82410108
73813	0	1	0.001	2.48	11.27612441	Up	0.46568	0.807107536
12511	0	1	0.001	2.47	11.27029533	Up	0.46568	0.840000711
237353	0	2	0.001	2.47	11.27029533	Up	0.224708	0.631048015
56622	0	1	0.001	2.47	11.27029533	Up	0.46568	0.767311541
234593	0	1	0.001	2.46	11.2644426	Up	0.46568	0.829215339
16976	0	1	0.001	2.46	11.2644426	Up	0.46568	0.808961019
13543	0	1	0.001	2.45	11.25856603	Up	0.46568	0.822003241
56490	0	1	0.001	2.45	11.25856603	Up	0.46568	0.806267849
242838	0	1	0.001	2.44	11.25266543	Up	0.46568	0.828329047
26377	0	1	0.001	2.42	11.24079133	Up	0.46568	0.813205305
73914	0	1	0.001	2.42	11.24079133	Up	0.46568	0.777784681
22687	0	1	0.001	2.41	11.23481743	Up	0.46568	0.803592551
170728	0	1	0.001	2.41	11.23481743	Up	0.46568	0.796162581
68058	0	1	0.001	2.41	11.23481743	Up	0.46568	0.767615547
432770	0	1	0.001	2.41	11.23481743	Up	0.46568	0.767007775
23879	0	1	0.001	2.4	11.22881869	Up	0.46568	0.817149574
21886	0	1	0.001	2.4	11.22881869	Up	0.46568	0.775139684
105835	0	1	0.001	2.39	11.2227949	Up	0.46568	0.819395915

11512	0	2	0.001	2.37	11.21067134	Up	0.224708	0.664225672
16656	0	3	0.001	2.37	11.21067134	Up	0.1084294	0.492758622
231287	0	2	0.001	2.37	11.21067134	Up	0.224708	0.6346895
78514	0	1	0.001	2.37	11.21067134	Up	0.46568	0.76609792
93685	0	2	0.001	2.36	11.20457114	Up	0.224708	0.64542467
56515	0	1	0.001	2.36	11.20457114	Up	0.46568	0.772050863
327900	0	1	0.001	2.35	11.19844504	Up	0.46568	0.79128513
264895	0	1	0.001	2.34	11.19229281	Up	0.46568	0.798459361
94246	0	2	0.001	2.34	11.19229281	Up	0.224708	0.633184987
52552	0	1	0.001	2.33	11.18611424	Up	0.46568	0.840182845
67074	0	3	0.001	2.31	11.17367714	Up	0.1084294	0.505740492
381628	0	1	0.001	2.31	11.17367714	Up	0.46568	0.816288873
77980	0	2	0.001	2.31	11.17367714	Up	0.224708	0.654461067
20745	0	1	0.001	2.3	11.16741815	Up	0.46568	0.773901194
219134	0	1	0.001	2.27	11.14847658	Up	0.46568	0.830637359
74385	0	1	0.001	2.27	11.14847658	Up	0.46568	0.792417849
12340	0	1	0.001	2.26	11.14210706	Up	0.46568	0.800934948
75953	0	1	0.001	2.25	11.13570929	Up	0.46568	0.778722524
192140	0	1	0.001	2.24	11.12928302	Up	0.46568	0.834393471
622645	0	1	0.001	2.24	11.12928302	Up	0.46568	0.77560514
107338	0	2	0.001	2.23	11.12282799	Up	0.224708	0.662343347
17857	0	1	0.001	2.23	11.12282799	Up	0.46568	0.770209358
232664	0	1	0.001	2.23	11.12282799	Up	0.46568	0.765492548
235050	0	1	0.001	2.22	11.11634396	Up	0.46568	0.803759237
75320	0	2	0.001	2.22	11.11634396	Up	0.224708	0.644090688
14461	0	1	0.001	2.21	11.10983065	Up	0.46568	0.795672131

17436	0	1	0.001	2.21	11.10983065	Up	0.46568	0.78710609
98314	0	1	0.001	2.21	11.10983065	Up	0.46568	0.777472568
57815	0	1	0.001	2.21	11.10983065	Up	0.46568	0.766552578
320111	0	1	0.001	2.19	11.09671515	Up	0.46568	0.815258422
22324	0	1.24	0.001	2.19	11.09671515	Up	0.46568	0.799942874
71145	0	1	0.001	2.18	11.09011242	Up	0.46568	0.797801787
270118	0	2	0.001	2.18	11.09011242	Up	0.224708	0.640779735
52440	0	1	0.001	2.17	11.08347933	Up	0.46568	0.809298931
57080	0	1	0.001	2.16	11.0768156	Up	0.46568	0.843291247
20350	0	1	0.001	2.16	11.0768156	Up	0.46568	0.82779818
76884	0	2	0.001	2.16	11.0768156	Up	0.224708	0.643204427
16918	0	1	0.001	2.16	11.0768156	Up	0.46568	0.7712825
11641	0	2	0.001	2.15	11.07012094	Up	0.224708	0.662812927
102632	0	1	0.001	2.15	11.07012094	Up	0.46568	0.800438604
320878	0	2	0.001	2.14	11.06339508	Up	0.224708	0.656990607
14191	0	1	0.001	2.14	11.06339508	Up	0.46568	0.790800669
106869	0	1	0.001	2.13	11.05663772	Up	0.46568	0.836374548
240047	0	1	0.001	2.13	11.05663772	Up	0.46568	0.782654672
74182	0	1	0.001	2.12	11.04984855	Up	0.46568	0.845683824
19244	0	1	0.001	2.12	11.04984855	Up	0.46568	0.79911802
70551	0	1	0.001	2.12	11.04984855	Up	0.46568	0.782338639
215493	0	1	0.001	2.12	11.04984855	Up	0.46568	0.762630049
105670	0	1	0.001	2.11	11.04302728	Up	0.46568	0.818357609
108797	0	1	0.001	2.1	11.03617361	Up	0.46568	0.797309317
83379	0	1	0.001	2.09	11.02928723	Up	0.46568	0.847348192
332131	0	1	0.001	2.09	11.02928723	Up	0.46568	0.819222681

286942	0	1	0.001	2.09	11.02928723	Up	0.46568	0.817321932
67470	0	1	0.001	2.09	11.02928723	Up	0.46568	0.810653406
94249	0	1	0.001	2.09	11.02928723	Up	0.46568	0.790478025
224703	0	1	0.001	2.08	11.02236781	Up	0.46568	0.852193376
67784	0	2	0.001	2.07	11.01541505	Up	0.224708	0.693544239
100504663	0	1	0.001	2.07	11.01541505	Up	0.46568	0.82992574
20873	0	1	0.001	2.06	11.00842862	Up	0.46568	0.854259982
15950	0	1.01	0.001	2.06	11.00842862	Up	0.46568	0.853131502
170760	0	1	0.001	2.06	11.00842862	Up	0.46568	0.833854805
219189	0	2	0.001	2.06	11.00842862	Up	0.224708	0.646764188
72805	0	1	0.001	2.05	11.00140819	Up	0.46568	0.819916056
54396	0	1	0.001	2.03	10.98726401	Up	0.46568	0.82797506
18755	0	1	0.001	2.02	10.98013958	Up	0.46568	0.834752969
74393	0	1	0.001	2.02	10.98013958	Up	0.46568	0.779662632
52840	0	1	0.001	2.01	10.97297979	Up	0.46568	0.775449926
16150	0	1	0.001	2	10.96578428	Up	0.46568	0.840365057
71772	0	1	0.001	2	10.96578428	Up	0.46568	0.836194061
75753	0	1	0.001	2	10.96578428	Up	0.46568	0.818530477
108682	0	1	0.001	2	10.96578428	Up	0.46568	0.793228921
56543	0	2	0.001	1.99	10.95855272	Up	0.224708	0.666831408
104001	0	1	0.001	1.98	10.95128471	Up	0.46568	0.836916475
68493	0	1	0.001	1.98	10.95128471	Up	0.46568	0.790155644
20465	0	1	0.001	1.96	10.93663794	Up	0.46568	0.822177653
14345	0	1	0.001	1.96	10.93663794	Up	0.46568	0.796489883
384763	0	1	0.001	1.96	10.93663794	Up	0.46568	0.783445871
246198	0	2	0.001	1.96	10.93663794	Up	0.224708	0.638373256

76484	0	2	0.001	1.95	10.92925841	Up	0.224708	0.675504071
20473	0	1	0.001	1.95	10.92925841	Up	0.46568	0.764737178
433022	0	2	0.001	1.94	10.92184094	Up	0.224708	0.689961354
56811	0	1	0.001	1.94	10.92184094	Up	0.46568	0.809467992
19303	0	1	0.001	1.93	10.91438513	Up	0.46568	0.83963668
19730	0	1	0.001	1.92	10.9068906	Up	0.46568	0.834573181
21975	0	1	0.001	1.92	10.9068906	Up	0.46568	0.764284671
13051	0	1	0.001	1.91	10.89935692	Up	0.46568	0.79257993
57778	0	1	0.001	1.9	10.8917837	Up	0.46568	0.830815455
12153	0	1	0.001	1.9	10.8917837	Up	0.46568	0.772666656
104174	0	1	0.001	1.89	10.88417052	Up	0.46568	0.791932001
434215	0	1	0.001	1.88	10.87651695	Up	0.46568	0.855391453
408067	0	0.99	0.001	1.88	10.87651695	Up	0.96507	0.970786686
22717	0	1	0.001	1.88	10.87651695	Up	0.46568	0.766249413
22025	0	1	0.001	1.87	10.86882255	Up	0.46568	0.829037929
98956	0	1	0.001	1.86	10.86108691	Up	0.46568	0.838727983
227682	0	1	0.001	1.86	10.86108691	Up	0.46568	0.774519944
216987	0	1	0.001	1.85	10.85330956	Up	0.46568	0.835833322
12549	0	2	0.001	1.85	10.85330956	Up	0.224708	0.654232074
407812	0	1	0.001	1.84	10.84549005	Up	0.46568	0.821306333
14164	0	1	0.001	1.84	10.84549005	Up	0.46568	0.810992733
14376	0	1	0.001	1.84	10.84549005	Up	0.46568	0.807275683
78088	0	1	0.001	1.84	10.84549005	Up	0.46568	0.801100533
58869	0	1	0.001	1.84	10.84549005	Up	0.46568	0.769291896
170759	0	1	0.001	1.83	10.83762793	Up	0.46568	0.837640138
217344	0	1	0.001	1.83	10.83762793	Up	0.46568	0.784079984

72925	0	1	0.001	1.82	10.82972274	Up	0.46568	0.814059513
327987	0	3	0.001	1.81	10.82177398	Up	0.1084294	0.517340044
381924	0	1	0.001	1.8	10.81378119	Up	0.46568	0.784397425
75345	0	1	0.001	1.8	10.81378119	Up	0.46568	0.769444654
26569	0	1	0.001	1.79	10.80574387	Up	0.46568	0.799447757
67345	0	1	0.001	1.78	10.79766153	Up	0.46568	0.815429983
24059	0	1	0.001	1.78	10.79766153	Up	0.46568	0.814572899
16646	0	1	0.001	1.78	10.79766153	Up	0.46568	0.773129146
16560	0	2	0.001	1.75	10.77313921	Up	0.224708	0.676481645
237256	0	1	0.001	1.75	10.77313921	Up	0.46568	0.826738485
110784	0	1	0.001	1.75	10.77313921	Up	0.46568	0.772974921
18033	0	1	0.001	1.74	10.76487159	Up	0.46568	0.825329772
108148	0	1	0.001	1.74	10.76487159	Up	0.46568	0.789029379
269233	0	1	0.001	1.73	10.75655632	Up	0.46568	0.823050824
26361	0	1	0.001	1.73	10.75655632	Up	0.46568	0.817839443
19211	0	2	0.001	1.73	10.75655632	Up	0.224708	0.655838396
264134	0	1	0.001	1.72	10.74819285	Up	0.46568	0.851444359
18143	0	1	0.001	1.72	10.74819285	Up	0.46568	0.828506154
109331	0	1	0.001	1.72	10.74819285	Up	0.46568	0.809129939
72119	0	1	0.001	1.72	10.74819285	Up	0.46568	0.805932463
17978	0	2	0.001	1.72	10.74819285	Up	0.224708	0.648558886
272382	0	0.66	0.001	1.71	10.73978061	Up	0.96507	0.971491346
72333	0	1	0.001	1.7	10.73131903	Up	0.46568	0.791123577
50772	0	1	0.001	1.7	10.73131903	Up	0.46568	0.786147957
20512	0	1	0.001	1.7	10.73131903	Up	0.46568	0.785033079
110891	0	1	0.001	1.69	10.72280753	Up	0.46568	0.849577566

433931	0	1	0.001	1.69	10.72280753	Up	0.46568	0.796981341
72749	0	1	0.001	1.69	10.72280753	Up	0.46568	0.769139198
225283	0	1	0.001	1.67	10.70563239	Up	0.46568	0.784874069
230648	0	1	0.001	1.66	10.69696753	Up	0.46568	0.79096209
11600	0	1	0.001	1.66	10.69696753	Up	0.46568	0.773746661
212483	0	1	0.001	1.65	10.68825031	Up	0.46568	0.807948974
11754	0	1	0.001	1.63	10.67065625	Up	0.46568	0.852756004
16835	0	1	0.001	1.63	10.67065625	Up	0.46568	0.800769432
20501	0	1	0.001	1.62	10.6617781	Up	0.46568	0.812523229
107035	0	1	0.001	1.62	10.6617781	Up	0.46568	0.774210446
140500	0	1	0.001	1.61	10.65284497	Up	0.46568	0.7831292
108062	0	1	0.001	1.61	10.65284497	Up	0.46568	0.779192294
224938	0	1	0.001	1.59	10.63481105	Up	0.46568	0.807612188
71371	0	1	0.001	1.59	10.63481105	Up	0.46568	0.793878976
106585	0	2	0.001	1.59	10.63481105	Up	0.224708	0.646093735
382985	0	1	0.001	1.58	10.62570884	Up	0.46568	0.810823034
66756	0	1	0.001	1.58	10.62570884	Up	0.46568	0.799777767
74164	0	1	0.001	1.57	10.61654884	Up	0.46568	0.818876433
320127	0	1	0.001	1.56	10.60733031	Up	0.46568	0.829570387
98258	0	1	0.001	1.56	10.60733031	Up	0.46568	0.820958322
69834	0	1	0.001	1.56	10.60733031	Up	0.46568	0.809806328
257632	0	1	0.001	1.55	10.5980525	Up	0.46568	0.853883491
622486	0	0.5	0.001	1.55	10.5980525	Up	0.96507	0.971726461
622474	0	0.5	0.001	1.55	10.5980525	Up	0.96507	0.971256346
84505	0	1	0.001	1.54	10.58871464	Up	0.46568	0.8503233
105504	0	1	0.001	1.54	10.58871464	Up	0.46568	0.848461414

22704	0	1	0.001	1.54	10.58871464	Up	0.46568	0.844946201
16364	0	1	0.001	1.53	10.57931594	Up	0.46568	0.813717614
68166	0	1	0.001	1.5	10.55074679	Up	0.46568	0.775915755
72507	0	1	0.001	1.49	10.54109662	Up	0.46568	0.805764874
104263	0	1	0.001	1.48	10.53138146	Up	0.46568	0.799282855
70428	0	1	0.001	1.47	10.52160044	Up	0.46568	0.845314852
213402	0	1	0.001	1.47	10.52160044	Up	0.46568	0.832779557
22762	0	1	0.001	1.44	10.4918531	Up	0.46568	0.832421757
16480	0	1	0.001	1.43	10.48179943	Up	0.46568	0.843842178
83436	0	1	0.001	1.43	10.48179943	Up	0.46568	0.83782125
77976	0	1	0.001	1.42	10.47167521	Up	0.46568	0.842557791
237868	0	1	0.001	1.42	10.47167521	Up	0.46568	0.842191541
217684	0	1	0.001	1.42	10.47167521	Up	0.46568	0.826209655
11835	0	2	0.001	1.42	10.47167521	Up	0.224708	0.65308951
16504	0	1	0.001	1.41	10.46147945	Up	0.46568	0.812012422
58235	0	1	0.001	1.41	10.46147945	Up	0.46568	0.795345501
67276	0	1	0.001	1.41	10.46147945	Up	0.46568	0.794530096
14236	0	1	0.001	1.4	10.45121111	Up	0.46568	0.824451762
78798	0	1	0.001	1.4	10.45121111	Up	0.46568	0.813888528
212073	0	1	0.001	1.4	10.45121111	Up	0.46568	0.776382144
228911	0	1	0.001	1.39	10.44086917	Up	0.46568	0.820436858
68732	0	1	0.001	1.39	10.44086917	Up	0.46568	0.798953254
271842	0	1	0.001	1.39	10.44086917	Up	0.46568	0.77934901
636931	0	1	0.001	1.38	10.43045255	Up	0.46568	0.788387239
75729	0	1	0.001	1.38	10.43045255	Up	0.46568	0.77143605
246710	0	1	0.001	1.37	10.41996018	Up	0.46568	0.810314362

79235	0	1	0.001	1.34	10.38801729	Up	0.46568	0.764888133
60599	0	1	0.001	1.33	10.37721053	Up	0.46568	0.847718941
217893	0	1	0.001	1.33	10.37721053	Up	0.46568	0.831171875
625018	0	1	0.001	1.33	10.37721053	Up	0.46568	0.824802742
20112	0	1	0.001	1.32	10.36632221	Up	0.46568	0.797473406
69718	0	1	0.001	1.32	10.36632221	Up	0.46568	0.794856057
16568	0	1	0.001	1.32	10.36632221	Up	0.46568	0.776693381
319880	0	1	0.001	1.31	10.3553511	Up	0.46568	0.763832699
226026	0	1	0.001	1.29	10.33315535	Up	0.46568	0.827444647
229900	0	1	0.001	1.28	10.32192809	Up	0.46568	0.824276384
243362	0	1	0.001	1.28	10.32192809	Up	0.46568	0.77825332
382056	0	1	0.001	1.26	10.29920802	Up	0.46568	0.789994552
320558	0	1	0.001	1.26	10.29920802	Up	0.46568	0.789350841
78334	0	1	0.001	1.26	10.29920802	Up	0.46568	0.788708178
227929	0	1	0.001	1.25	10.28771238	Up	0.46568	0.775760416
27273	0	0.6	0.001	1.25	10.28771238	Up	0.96507	0.970083048
241226	0	1	0.001	1.24	10.27612441	Up	0.46568	0.786307484
106633	0	1	0.001	1.23	10.2644426	Up	0.46568	0.787746144
12801	0	1	0.001	1.23	10.2644426	Up	0.46568	0.766855983
213499	0	1	0.001	1.2	10.22881869	Up	0.46568	0.804760806
331623	0	1	0.001	1.18	10.20457114	Up	0.46568	0.76368216
18008	0	1	0.001	1.16	10.17990909	Up	0.46568	0.848090015
19877	0	1	0.001	1.16	10.17990909	Up	0.46568	0.796817454
27404	0	1	0.001	1.16	10.17990909	Up	0.46568	0.793553815
320376	0	1	0.001	1.16	10.17990909	Up	0.46568	0.790316802
78455	0	1	0.001	1.15	10.16741815	Up	0.46568	0.811842296

12669	0	1	0.001	1.15	10.16741815	Up		0.46568	0.764435447
11789	0	2	0.001	1.14	10.15481811	Up		0.224708	0.690470926
66526	0	1	0.001	1.14	10.15481811	Up		0.46568	0.780133537
71960	0	1	0.001	1.12	10.12928302	Up		0.46568	0.77359219
75089	0	1	0.001	1.11	10.11634396	Up		0.46568	0.834034283
327814	0	1	0.001	1.11	10.11634396	Up		0.46568	0.769750354
433586	0	1	0.001	1.1	10.10328781	Up		0.46568	0.813546773
17880	0	1	0.001	1.08	10.0768156	Up		0.46568	0.791770184
320632	0	1	0.001	1.06	10.04984855	Up		0.46568	0.853319375
227541	0	1	0.001	1.06	10.04984855	Up		0.46568	0.779819537
77018	0	1	0.001	1.06	10.04984855	Up		0.46568	0.774674786
432572	0	1	0.001	1.06	10.04984855	Up		0.46568	0.772204719
627626	0	1	0.001	1.04	10.02236781	Up		0.46568	0.852568378
16764	0	1	0.001	0.99	9.951284715	Up		0.46568	0.808286041
77011	0	1	0.001	0.99	9.951284715	Up		0.46568	0.783921359
16798	0	1	0.001	0.99	9.951284715	Up		0.46568	0.767463514
100710	0	1	0.001	0.98	9.936637939	Up		0.46568	0.805262527
225115	0	1	0.001	0.96	9.906890596	Up		0.46568	0.772358637
19820	0	1	0.001	0.96	9.906890596	Up		0.46568	0.768681468
213109	0	1	0.001	0.94	9.876516947	Up		0.46568	0.820089583
67958	0	1	0.001	0.94	9.876516947	Up		0.46568	0.819742602
19268	0	1	0.001	0.93	9.861086906	Up		0.46568	0.805597356
15463	0	1	0.001	0.92	9.845490051	Up		0.46568	0.816977289
66860	0	1	0.001	0.92	9.845490051	Up		0.46568	0.804259709
54650	0	1	0.001	0.91	9.829722735	Up		0.46568	0.786467075
71228	0	1	0.001	0.91	9.829722735	Up		0.46568	0.780290632

244650	0	1	0.001	0.91	9.829722735	Up	0.46568	0.7632309
11519	0	1	0.001	0.89	9.797661526	Up	0.46568	0.832064265
121021	0	1	0.001	0.89	9.797661526	Up	0.46568	0.811332345
192188	0	1	0.001	0.88	9.781359714	Up	0.46568	0.794204403
208431	0	1	0.001	0.87	9.764871591	Up	0.46568	0.795182286
224014	0	1	0.001	0.86	9.74819285	Up	0.46568	0.844209865
19255	0	1	0.001	0.85	9.731319031	Up	0.46568	0.802760157
72194	0	1	0.001	0.83	9.696967526	Up	0.46568	0.780447791
269116	0	1	0.001	0.76	9.569855608	Up	0.46568	0.825857477
269610	0	1	0.001	0.76	9.569855608	Up	0.46568	0.815086933
347722	0	1	0.001	0.76	9.569855608	Up	0.46568	0.808117472
320772	0	1.01	0.001	0.73	9.511752654	Up	0.46568	0.763381261
14104	0	1	0.001	0.72	9.491853096	Up	0.46568	0.798623924
21676	0	1	0.001	0.72	9.491853096	Up	0.46568	0.772512616
229003	0	1	0.001	0.7	9.451211112	Up	0.46568	0.831707079
13176	0	1	0.001	0.69	9.430452552	Up	0.46568	0.832600619
209683	0	1	0.001	0.67	9.388017285	Up	0.46568	0.788066561
70549	0	1.11	0.001	0.65	9.344295908	Up	0.46568	0.854071695
271564	0	1	0.001	0.64	9.321928095	Up	0.46568	0.793391335
65969	0	1	0.001	0.63	9.299208018	Up	0.46568	0.786946239
20273	0	1	0.001	0.63	9.299208018	Up	0.46568	0.778879051
17101	0	1	0.001	0.6	9.22881869	Up	0.46568	0.801929487
77771	0	0.77	0.001	0.54	9.076815597	Up	0.96507	0.970669342
14725	0	1	0.001	0.46	8.845490051	Up	0.46568	0.824978344
64113	0	0.23	0.001	0.45	8.813781191	Up	0.96507	0.971373832
11426	0	1	0.001	0.41	8.6794801	Up	0.46568	0.786786453

100503043	0	0.46	0.001	0.36	8.491853096	Up	0.96507	0.970552027
319161	0.02	5	0.46	107.21	7.864589903	Up	0.0252468	0.245993704
229791	1	19.82	1.17	24.92	4.412723633	Up	1.06E-05	0.00064571
52850	1.24	22.45	2.77	53.61	4.274544254	Up	1.36E-06	1.00E-04
20541	1	18	0.42	7.64	4.185111405	Up	2.10E-05	0.001136162
217721	1	15	1.95	31.37	4.0078395	Up	0.000160331	0.006322824
230654	1	13	2.13	29.71	3.80202327	Up	0.00061179	0.019731413
547347	1	13	4.15	57.86	3.801383082	Up	0.00061179	0.01965523
13498	1	13	1.51	21.05	3.801199779	Up	0.00061179	0.019808189
67620	1	13	1.96	27.3	3.799975392	Up	0.00061179	0.019579633
68235	1	8	1.26	17.17	3.768394401	Up	0.01604696	0.189399651
72472	1	12.46	2.92	38.9	3.735729881	Up	0.00118829	0.032959204
13171	1.3	15.75	2.65	34.38	3.69750528	Up	0.000160331	0.006352933
241324	1	12	1.04	13.42	3.689729238	Up	0.00118829	0.032849705
14816	13	146	12.51	150.68	3.590334244	Up	5.48E-32	4.15E-29
71971	1	11	2.47	29.12	3.559427409	Up	0.00229768	0.054315328
12773	2	20.83	4.82	53.83	3.481305374	Up	3.33E-05	0.001701078
216963	1	10	1.82	19.56	3.425896015	Up	0.00441992	0.087567034
54188	1	10	4.17	44.7	3.422155543	Up	0.00441992	0.087776025
228960	1	10	1.81	19.37	3.419762352	Up	0.00441992	0.087986015
11832	2.04	18.7	5.43	53.42	3.298355873	Up	0.000119792	0.004910275
20661	1	9	0.97	9.39	3.275068505	Up	0.0084515	0.134207885
240675	1	9	1.72	16.62	3.272439912	Up	0.0084515	0.133191158
18024	1	9	2.69	25.99	3.272278556	Up	0.0084515	0.133697588
76192	1	9	3.43	33.13	3.271857722	Up	0.0084515	0.132438666
64659	1	9	3.43	33.08	3.269678753	Up	0.0084515	0.133443893

68720	1	9	10.85	104.61	3.269253822	Up	0.0084515	0.132939379
19943	1	9	14.4	138.78	3.268658955	Up	0.0084515	0.13268855
20630	1	9	8.98	86.54	3.268579771	Up	0.0084515	0.13395225
238564	9.75	84.93	11.54	107.68	3.222035186	Up	7.88E-18	2.52E-15
209318	1	8	3.38	31.17	3.205063003	Up	0.01604696	0.188331106
18247	2	17	7.28	66.32	3.187433651	Up	0.000225704	0.008498113
14180	2	16.9	3.23	29.23	3.177843759	Up	0.000423248	0.014797675
16592	1	8	7.11	62.22	3.12945693	Up	0.01604696	0.190209052
110119	1	8	3.85	33.04	3.101283336	Up	0.01604696	0.189938484
72630	3	24	6.84	58.64	3.099816873	Up	1.24E-05	0.000726967
70240	1	8	6.91	59.23	3.09957047	Up	0.01604696	0.188065851
100504112	1	8	6.54	56.03	3.098836952	Up	0.01604696	0.187537576
213603	1	8	2.6	22.27	3.09851803	Up	0.01604696	0.190480391
73713	1	8	1.01	8.65	3.09834484	Up	0.01604696	0.190752506
66449	1	8	12.85	110.03	3.098056668	Up	0.01604696	0.18913138
72948	1	8	1.31	11.21	3.097147561	Up	0.01604696	0.191025399
67017	1	8	1.86	15.91	3.096559309	Up	0.01604696	0.187801342
16362	2	16	6.34	54.16	3.094672993	Up	0.000423248	0.01473576
52882	18.39	140.31	21.05	172.17	3.031941642	Up	9.28E-27	5.94E-24
230073	2	15	2.7	21.73	3.008656862	Up	0.000789544	0.023297148
75763	1.1	8.1	0.94	7.47	2.990375581	Up	0.01604696	0.189668685
11564	1	7	1.8	13.53	2.910093028	Up	0.0302136	0.268597613
26896	1	7	0.97	7.29	2.909862162	Up	0.0302136	0.272085894
22247	1	7	2.08	15.63	2.909662345	Up	0.0302136	0.270330501
75083	1	7	3.87	29.06	2.908629231	Up	0.0302136	0.269172768
26412	2	14	2.87	21.55	2.908565227	Up	0.001464302	0.037722777

54218	1	7	4.24	31.83	2.908250987	Up	0.0302136	0.270040135
16565	1	7	0.73	5.48	2.908207524	Up	0.0302136	0.271205357
20621	1	7	2.3	17.26	2.907726698	Up	0.0302136	0.270913109
19186	1	7	7.05	52.88	2.907027014	Up	0.0302136	0.269750392
68554	1	7	5.07	38.01	2.906321373	Up	0.0302136	0.271498235
14911	1	7	3.2	23.99	2.906289347	Up	0.0302136	0.269461271
70925	1	7	1.92	14.39	2.905888376	Up	0.0302136	0.268310956
242700	1	7	1.67	12.51	2.905161782	Up	0.0302136	0.268884883
72667	0.15	1.01	0.23	1.72	2.902702799	Up	0.46568	0.801763559
13800	3.29	22.99	1.86	13.52	2.861720625	Up	4.27E-05	0.002088884
18823	12.16	80.04	23.96	169.74	2.824626769	Up	8.24E-15	2.08E-12
57261	1	4	1.12	7.9	2.818353921	Up	0.1876922	0.613668682
71939	0.82	5.32	2.68	18.61	2.79577315	Up	0.0252468	0.248613755
29808	2.25	14.37	1.12	7.7	2.781359714	Up	0.001464302	0.037606349
66487	3	19	17.12	116.23	2.763227882	Up	0.000263188	0.009563264
100040048	0.25	1	2.45	16.29	2.73313295	Up	0.46568	0.835472893
100861978	0.25	1	2.45	16.29	2.73313295	Up	0.46568	0.770515665
18187	3.2	19.91	3.19	21.2	2.732435936	Up	0.000263188	0.009521684
71742	9	54.46	21.39	138.74	2.697375402	Up	6.74E-10	9.85E-08
14154	1	6	0.97	6.27	2.692408791	Up	0.0563104	0.355507465
72668	1	6	1.52	9.8	2.688710426	Up	0.0563104	0.360152835
56218	1	6	2.16	13.92	2.688055994	Up	0.0563104	0.354700105
14055	1	6	1.58	10.18	2.687741098	Up	0.0563104	0.354163899
70823	1	6	2.45	15.78	2.687243551	Up	0.0563104	0.35987622
26424	1	6	1.84	11.85	2.687109388	Up	0.0563104	0.358773996
216850	1	6	1	6.44	2.687060688	Up	0.0563104	0.355237937

56856	1	6	2.16	13.91	2.687019202	Up	0.0563104	0.354968817
24105	1	6	2.99	19.25	2.686641056	Up	0.0563104	0.354431799
11429	1	6	2.41	15.51	2.686093635	Up	0.0563104	0.359600029
22601	1	6	1.61	10.36	2.68589141	Up	0.0563104	0.357133261
20431	2	12	6.33	40.73	2.68581441	Up	0.00493554	0.095286841
229675	1	6	0.99	6.37	2.685792942	Up	0.0563104	0.356318508
333715	1	6	6.95	44.71	2.685512663	Up	0.0563104	0.356589679
56309	3	18	13.73	88.3	2.685081812	Up	0.00047754	0.016087491
225898	2	12	4.2	27.01	2.685032407	Up	0.00493554	0.094846717
67608	1	6	1.51	9.71	2.684922746	Up	0.0563104	0.357951748
66949	1	6	2.3	14.79	2.684916286	Up	0.0563104	0.356861263
67808	3	18	11.57	74.39	2.684719833	Up	0.00047754	0.016218818
20492	3	18	11.18	71.88	2.684670221	Up	0.00047754	0.016152888
60409	1	6	5.72	36.77	2.684442124	Up	0.0563104	0.355777402
11983	1	6	4.49	28.86	2.68428395	Up	0.0563104	0.357405674
66379	1	6	9.41	60.47	2.683952951	Up	0.0563104	0.358225412
67025	1	6	11.88	76.34	2.68390435	Up	0.0563104	0.360429876
244349	1	6	0.73	4.69	2.683619554	Up	0.0563104	0.359324263
671535	1	6	2.03	13.04	2.683392237	Up	0.0563104	0.35604775
98582	1	6	3	19.27	2.683322166	Up	0.0563104	0.358499494
56349	2	12	3.45	22.09	2.678725152	Up	0.00493554	0.095066269
17996	1	6	0.3	1.9	2.662965013	Up	0.0563104	0.359048918
75302	4.33	25	3.23	20.04	2.633276439	Up	2.68E-05	0.001401843
12476	1	5	3.54	21.8	2.62250687	Up	0.1036324	0.502227839
319236	9	51.55	16.66	101.58	2.608156073	Up	3.82E-09	4.67E-07
22631	1	6	1.86	11.33	2.606773335	Up	0.0563104	0.357678503

66205	3	17	15.31	92.95	2.601980582	Up	0.000861184	0.024968335
16956	98.58	546.28	162.84	967.49	2.570791623	Up	1.65E-83	3.44E-80
58243	2	11	7.08	41.74	2.559609335	Up	0.00895402	0.136458609
226548	2	11	4.66	27.47	2.55945505	Up	0.00895402	0.136209142
70481	2	11	5.71	33.64	2.558615055	Up	0.00895402	0.135712933
18975	2	11	2.91	17.14	2.558276051	Up	0.00895402	0.135960585
23881	2	11	3.16	18.45	2.545624353	Up	0.00895402	0.136708992
56406	1	3	0.97	5.52	2.508611615	Up	0.332862	0.687622816
77032	1.07	5.7	4.52	25.71	2.507934932	Up	0.1036324	0.504578818
18703	133.04	697.01	231.29	1299.21	2.489859846	Up	5.21E-102	1.45E-98
75209	61.97	323.56	97.44	545.45	2.484860916	Up	1.66E-48	1.97E-45
50911	12	62	50.17	277.87	2.46951324	Up	2.68E-10	4.06E-08
668923	10.05	51.59	9.35	51.44	2.459852373	Up	1.25E-08	1.37E-06
17876	7	35	16.05	87.75	2.450825828	Up	3.12E-06	0.000211337
60533	5.96	30.09	10.93	59.12	2.435352868	Up	5.03E-06	0.000324758
244923	1	5	1.06	5.71	2.429426481	Up	0.1036324	0.506654054
277939	1	5	0.85	4.57	2.426659419	Up	0.1036324	0.507847586
56857	1	5	1.52	8.17	2.426264755	Up	0.1036324	0.509949852
20682	1	5	1.61	8.65	2.425639444	Up	0.1036324	0.507250118
107368	2	10	1.88	10.1	2.425550726	Up	0.0160922	0.185976661
69608	1	5	1.72	9.24	2.425484287	Up	0.1036324	0.500188631
225358	1	5	1.99	10.69	2.425421517	Up	0.1036324	0.503106885
320435	1	5	2.32	12.46	2.425107358	Up	0.1036324	0.500478932
218454	2	10	3.07	16.48	2.424405682	Up	0.0160922	0.187277197
12807	1	5	1.85	9.93	2.424268447	Up	0.1036324	0.508146848
67111	1	5	2.78	14.92	2.424090748	Up	0.1036324	0.509648464

68815	1	5	2.62	14.06	2.423957878	Up	0.1036324	0.502520513
73991	1	5	2.38	12.77	2.423725046	Up	0.1036324	0.501351861
384724	1	5	4.49	24.08	2.423048042	Up	0.1036324	0.504283743
110379	1	5	5.24	28.1	2.422931414	Up	0.1036324	0.508746431
77407	1	5	3.09	16.57	2.422894859	Up	0.1036324	0.508446463
73420	2	10	4.94	26.49	2.422864897	Up	0.0160922	0.185204974
104479	2	10	3.92	21.02	2.42283711	Up	0.0160922	0.187802519
78689	1	5	2.54	13.62	2.422826301	Up	0.1036324	0.509046753
66921	5	25	11.17	59.89	2.422685946	Up	8.93E-05	0.003931897
76123	5	25	9.54	51.15	2.422673069	Up	8.93E-05	0.003911203
15404	1	5	3.46	18.55	2.422575244	Up	0.1036324	0.506356548
225600	1	5	1.58	8.47	2.422437411	Up	0.1036324	0.501060546
213819	1	5	1.78	9.54	2.422112025	Up	0.1036324	0.501935507
93730	1	5	1.95	10.45	2.421956913	Up	0.1036324	0.504874239
56401	2	10	4.43	23.74	2.421941331	Up	0.0160922	0.187015637
17463	4	20	16.79	89.97	2.421841793	Up	0.000488618	0.016263162
12348	1	5	4.69	25.13	2.421750843	Up	0.1036324	0.50605939
11777	2	10	8.94	47.9	2.421678919	Up	0.0160922	0.186754806
12810	1	5	2.71	14.52	2.421676697	Up	0.1036324	0.503989013
67785	1	5	0.95	5.09	2.421666238	Up	0.1036324	0.510856161
666731	2	10	6.79	36.38	2.421662063	Up	0.0160922	0.186494702
98053	2	10	7.88	42.21	2.421317295	Up	0.0160922	0.186235322
74007	1	5	1.15	6.16	2.42129649	Up	0.1036324	0.511158981
67067	1	5	12.48	66.84	2.421093799	Up	0.1036324	0.50076957
399510	1	5	1.49	7.98	2.421076416	Up	0.1036324	0.502813528
13877	1	5	8.55	45.79	2.42103624	Up	0.1036324	0.511462159

194908	1	5	3.83	20.51	2.420911195	Up	0.1036324	0.505170006
68520	1	5	5.21	27.9	2.420909844	Up	0.1036324	0.503694626
20335	1	5	9.24	49.48	2.420880744	Up	0.1036324	0.510553701
16765	1	5	6.42	34.37	2.420504649	Up	0.1036324	0.505762581
17178	2	10	27.16	145.39	2.420372659	Up	0.0160922	0.185718719
14561	1	5	3.74	20.02	2.420331799	Up	0.1036324	0.509347431
20910	1	5	1.73	9.26	2.420240156	Up	0.1036324	0.507548676
80289	1	5	2.14	11.45	2.419664897	Up	0.1036324	0.510251598
69134	1	5	19.98	106.89	2.419498401	Up	0.1036324	0.50546612
76577	1	5	1.55	8.29	2.419103886	Up	0.1036324	0.503400584
227699	1	5	1.18	6.31	2.418853146	Up	0.1036324	0.501643514
240888	2	10	1.96	10.48	2.418713157	Up	0.0160922	0.18753949
228357	1	1.99	0.83	4.41	2.409595414	Up	0.947628	0.970846169
53872	1	5	1.09	5.78	2.406741358	Up	0.1036324	0.506951911
13682	6	20	12.63	64.82	2.359584382	Up	0.0033776	0.069912959
14531	8	37.86	26.55	134.67	2.342644736	Up	3.10E-06	0.000211577
192734	3	14	4.36	21.8	2.321928095	Up	0.0048381	0.094060351
22696	0.99	4.54	1.9	9.34	2.297423131	Up	0.052321	0.348569288
52822	2.35	10.69	4.41	21.56	2.289506617	Up	0.0160922	0.185461491
12892	3.5	15.93	7.36	35.95	2.288214099	Up	0.00274312	0.062194827
216766	2	9	2.18	10.54	2.273474827	Up	0.0286094	0.260743502
242915	2	9	3.49	16.85	2.27144965	Up	0.0286094	0.263631027
67809	2	9	5.98	28.87	2.271353718	Up	0.0286094	0.261603096
223648	2	9	5.85	28.24	2.271231559	Up	0.0286094	0.262468376
19377	2	9	1.87	9.02	2.270089163	Up	0.0286094	0.260173571
245347	2	9	14.69	70.85	2.269933457	Up	0.0286094	0.261029405

108037	2	9	5.87	28.31	2.269879341	Up	0.0286094	0.260458225
210094	2	9	5.24	25.27	2.269786947	Up	0.0286094	0.262758077
67615	2	9	4.05	19.53	2.269698136	Up	0.0286094	0.263048417
66816	2	8.99	3.93	18.95	2.269596631	Up	0.0502266	0.363422207
109624	2	9	3.32	16	2.268816758	Up	0.0286094	0.2633394
68813	2	9	1.29	6.21	2.267222203	Up	0.0286094	0.261890888
21869	16.16	70.25	45.86	218.24	2.250607289	Up	2.67E-10	4.11E-08
317653	78.07	344.48	415.9	1966.78	2.241527	Up	2.59E-45	2.39E-42
74455	1	4	2.45	11.57	2.23953521	Up	0.1876922	0.618529424
57782	1.1	4.85	2.19	10.31	2.235041558	Up	0.1876922	0.620988786
26965	3	13	6.34	29.47	2.216692315	Up	0.0084561	0.132261669
60527	3	13	6.15	28.58	2.216347601	Up	0.0084561	0.131766307
319317	14.7	63.68	16.72	77.66	2.21559686	Up	1.40E-09	1.94E-07
14314	3	13	5.57	25.86	2.214973042	Up	0.0084561	0.132013524
241113	18.7	80.18	44.29	203.61	2.200755517	Up	1.03E-11	1.91E-09
319740	1.91	8.17	2.32	10.63	2.195944886	Up	0.01604696	0.188863867
108015	2.14	9.13	4.12	18.81	2.190783606	Up	0.0286094	0.261315936
18377	4	17	12.11	55.17	2.187685115	Up	0.00258758	0.059643361
99982	4	17	8.86	40.36	2.187547571	Up	0.00258758	0.059809037
215690	1.01	4.19	0.52	2.34	2.169925001	Up	0.1876922	0.621483007
18103	1	4	6.69	29.84	2.15716942	Up	0.1876922	0.607935693
17025	1	4	1.47	6.44	2.131244533	Up	0.1876922	0.616575916
69368	1	4.02	1.46	6.3	2.10938346	Up	0.1876922	0.611506185
22354	1	4	1.35	5.81	2.105578756	Up	0.1876922	0.611027698
14248	1	4	1.64	7.05	2.103927443	Up	0.1876922	0.609358875
278679	0.5	2	1.51	6.49	2.103669929	Up	0.224708	0.665881506

666348	0.5	2	1.51	6.49	2.103669929	Up	0.224708	0.639464866
20239	1	4	1.48	6.36	2.10342959	Up	0.1876922	0.62049535
212124	1	4	0.81	3.48	2.103093493	Up	0.1876922	0.616332595
22628	1	4	1.86	7.99	2.102892882	Up	0.1876922	0.619510828
74019	1	4	1.9	8.16	2.102569734	Up	0.1876922	0.612225322
28185	1	4	1.77	7.6	2.102250058	Up	0.1876922	0.606990593
223666	2	8	2.97	12.75	2.101962411	Up	0.0502266	0.360911519
76793	1	4	2.87	12.32	2.101879614	Up	0.1876922	0.615846528
22619	1	4	1.95	8.37	2.101753499	Up	0.1876922	0.610788735
66614	1	4	4.14	17.77	2.101741009	Up	0.1876922	0.609121215
53378	2	8	5.27	22.62	2.101724062	Up	0.0502266	0.364372745
240614	1	4	1.37	5.88	2.101640262	Up	0.1876922	0.61779541
17758	1	4	1.37	5.88	2.101640262	Up	0.1876922	0.610549959
71522	1	4	3.67	15.75	2.10149986	Up	0.1876922	0.615603782
319352	2	8	6.5	27.89	2.101236311	Up	0.0502266	0.361223456
234404	1	4	2.41	10.34	2.101131134	Up	0.1876922	0.611985422
68294	1	4	3.55	15.23	2.101025012	Up	0.1876922	0.614876691
320864	2	8	5.17	22.18	2.10102318	Up	0.0502266	0.362476616
13602	1	4	2.14	9.18	2.100883357	Up	0.1876922	0.60959672
66226	1	4	5.11	21.92	2.100852602	Up	0.1876922	0.605813342
14619	1	4	2.8	12.01	2.100737419	Up	0.1876922	0.606519144
211586	5	20	4.84	20.76	2.100727491	Up	0.001379634	0.036214304
27389	1	4	3.88	16.64	2.100526876	Up	0.1876922	0.614634709
244091	2	8	4.51	19.34	2.100388456	Up	0.0502266	0.365009204
445007	1	4	3.09	13.25	2.100313616	Up	0.1876922	0.621235798
228966	1	4	2.05	8.79	2.100239256	Up	0.1876922	0.615361228

66086	1	4	4.73	20.28	2.100145564	Up	0.1876922	0.621730413
114886	1	4	2.89	12.39	2.10003479	Up	0.1876922	0.606754777
100206	1	4	4.98	21.35	2.100018422	Up	0.1876922	0.608646452
319734	1	4	1.15	4.93	2.099953785	Up	0.1876922	0.618774483
11465	1	4	3.52	15.09	2.099945472	Up	0.1876922	0.616089466
72018	1	4	2.37	10.16	2.099941438	Up	0.1876922	0.613909904
20289	1	4	6.03	25.85	2.099934373	Up	0.1876922	0.617063136
67959	1	4	3.66	15.69	2.099929799	Up	0.1876922	0.613186807
20018	1	4	5.97	25.59	2.099777311	Up	0.1876922	0.620248926
231503	2	8	4.33	18.56	2.09975778	Up	0.0502266	0.36060012
71711	2	8	6.74	28.89	2.099749707	Up	0.0502266	0.364690697
56846	1	4	3.74	16.03	2.09966425	Up	0.1876922	0.615118864
11911	1	4	3.88	16.63	2.099659611	Up	0.1876922	0.61681943
74008	1	4	4.34	18.6	2.099535674	Up	0.1876922	0.617307034
102098	2	8	2.52	10.8	2.099535674	Up	0.0502266	0.362162512
67456	1	4	1.75	7.5	2.099535674	Up	0.1876922	0.608883741
76933	1	4	10.54	45.17	2.099490047	Up	0.1876922	0.621978015
320351	2	8	11.35	48.64	2.099450931	Up	0.0502266	0.363106463
14538	2	8	3.33	14.27	2.099391252	Up	0.0502266	0.361848951
78658	2	8	2.42	10.37	2.099336942	Up	0.0502266	0.361535933
58859	1	4	4.35	18.64	2.099314554	Up	0.1876922	0.614151316
20016	1	4	5.23	22.41	2.099259797	Up	0.1876922	0.620002698
68292	1	4	1.58	6.77	2.099231275	Up	0.1876922	0.611745709
74600	1	4	7.69	32.95	2.099222962	Up	0.1876922	0.619019737
19899	1	4	9.87	42.29	2.099194571	Up	0.1876922	0.614392917
106504	1	4	2.04	8.74	2.099064128	Up	0.1876922	0.618039888

225027	1	4	2.99	12.81	2.099053086	Up		0.1876922	0.607226593
14632	2	8	3.66	15.68	2.099010006	Up		0.0502266	0.363738502
72397	2	8	4.05	17.35	2.09894185	Up		0.0502266	0.362791266
381580	1	4	3.24	13.88	2.09894185	Up		0.1876922	0.612946152
233064	1	4	1.41	6.04	2.098853387	Up		0.1876922	0.61342765
96957	1	4	2.33	9.98	2.098709861	Up		0.1876922	0.608172428
75099	1	4	2.26	9.68	2.098684275	Up		0.1876922	0.607462776
80838	1	4	9.36	40.09	2.098661983	Up		0.1876922	0.622225815
77519	1	4	1.06	4.54	2.098628033	Up		0.1876922	0.62074197
56150	1	4	3.93	16.83	2.098433959	Up		0.1876922	0.617551125
17341	1	4	1.92	8.22	2.098032083	Up		0.1876922	0.609834751
16425	1	4	2.14	9.16	2.097736802	Up		0.1876922	0.619756665
105782	1	4	1.22	5.22	2.097168659	Up		0.1876922	0.611266848
109154	1	4	1.12	4.79	2.096526924	Up		0.1876922	0.610072967
266645	2.02	8.05	5.9	25.23	2.096353347	Up		0.0502266	0.364055347
328949	31.96	127.41	27.13	115.93	2.095292998	Up		1.00E-16	2.68E-14
72750	1	4	1.21	5.17	2.095157233	Up		0.1876922	0.606048427
67454	1	3.99	2.07	8.83	2.09278267	Up		0.332862	0.68677032
21418	1	4	1.93	8.23	2.092291583	Up		0.1876922	0.608409348
16664	24	90	100.27	424.87	2.083131451	Up		1.98E-11	3.58E-09
73680	4.39	17.3	13.27	56.07	2.079060699	Up		0.00258758	0.0594786
56508	1.85	7.28	3.19	13.42	2.072756342	Up		0.0302136	0.270621491
74018	16.55	64.85	37.3	156.73	2.07103382	Up		6.13E-09	6.99E-07
13047	1	1	0.51	2.14	2.069041644	Up		0.947628	0.981480282
22754	17	46	19.61	82.16	2.066846646	Up		7.40E-05	0.003384251
69232	1	4	2.07	8.55	2.046293652	Up		0.1876922	0.619265185

229279	33	95	51.02	210.23	2.042833754	Up	2.52E-09	3.23E-07
54632	3.55	13.48	6.96	28.31	2.024152538	Up	0.0084561	0.131520015
117150	4	15	7.94	31.94	2.0081534	Up	0.0075449	0.121433487
232414	5	17	10.43	41.54	1.993762058	Up	0.00651834	0.110242088
50779	1.9	7	2.26	8.96	1.98717596	Up	0.0302136	0.26802491
14755	5	19	10.48	41.44	1.983385286	Up	0.00233514	0.055044476
225339	3	11	4.19	16.48	1.975694093	Up	0.025028	0.256159887
72508	3	11	3.71	14.59	1.975488791	Up	0.025028	0.255845194
13665	3	11	6.38	25.09	1.975484142	Up	0.025028	0.255218123
16467	3	11	5.46	21.47	1.975349335	Up	0.025028	0.255531274
66646	6	22	15.04	59.12	1.974841702	Up	0.001231978	0.033832637
27276	3	11	11.38	44.73	1.974742201	Up	0.025028	0.254594117
53612	6	22	31.58	124.11	1.974536287	Up	0.001231978	0.033721345
66530	3	11	11.86	46.6	1.974225945	Up	0.025028	0.254283258
12972	3	11	7.89	31	1.97417101	Up	0.025028	0.254905738
20602	10	34	7.65	29.79	1.961296471	Up	9.81E-05	0.004184772
52563	31	112	50.14	194.24	1.953806425	Up	1.89E-13	4.49E-11
142687	1	4	3.73	14.37	1.945812526	Up	0.1876922	0.61246541
240752	26.9	96.36	22.59	86.76	1.941345782	Up	6.04E-12	1.20E-09
170947	15.03	53.85	29.65	113.87	1.941283697	Up	6.26E-07	5.06E-05
22598	1.19	4.24	1.98	7.53	1.927149435	Up	0.1876922	0.606283694
75560	2	7	1.24	4.67	1.913082429	Up	0.0868802	0.454101849
213056	2	7	1.54	5.79	1.910632997	Up	0.0868802	0.455245683
252972	2	7	2.86	10.75	1.910249608	Up	0.0868802	0.454959185
319262	2	7	3.16	11.87	1.909323471	Up	0.0868802	0.457261318
55943	2	7	3.24	12.17	1.90926345	Up	0.0868802	0.453247739

18587	2	7	4.74	17.8	1.908918277	Up	0.0868802	0.453816789
23856	4	14	3.14	11.79	1.908727254	Up	0.01269428	0.162007828
19647	2	7	2.15	8.07	1.908232014	Up	0.0868802	0.456395293
15413	2	7	7.07	26.52	1.907298655	Up	0.0868802	0.455532542
67846	2	7	5.14	19.28	1.907264787	Up	0.0868802	0.454387269
66734	2	7	12.92	48.45	1.906890596	Up	0.0868802	0.454673047
71766	2	7	3.87	14.51	1.906642048	Up	0.0868802	0.456683603
19359	2	7	3.5	13.12	1.906340893	Up	0.0868802	0.456107346
13486	2	7	4.36	16.34	1.906007943	Up	0.0868802	0.455819763
269800	2	7	4.28	16.04	1.90599144	Up	0.0868802	0.452963749
75723	2	7	1.49	5.58	1.904952791	Up	0.0868802	0.456972278
26365	1.78	7.22	3.8	14.07	1.888551005	Up	0.0302136	0.271791747
434768	33.36	115.09	189.09	699.21	1.88665275	Up	2.46E-13	5.68E-11
56470	2	5	7.71	28.43	1.882611334	Up	0.245598	0.66894303
320595	1	2	1.05	3.85	1.874469118	Up	0.573542	0.723535928
72047	5	17	8.3	30.27	1.866705434	Up	0.00651834	0.109573954
54160	45	153	74.95	273.22	1.866062711	Up	7.12E-17	2.12E-14
17775	5	17	15.83	57.7	1.865910063	Up	0.00651834	0.110466613
76498	13	44	34.88	126.56	1.85934956	Up	9.28E-06	0.000567541
26356	1	4	3.6	13.04	1.856875058	Up	0.1876922	0.612705687
241322	33.14	110.79	48.83	175.02	1.841680109	Up	2.68E-12	5.86E-10
71752	3	10	4.4	15.74	1.838860112	Up	0.0422656	0.323841674
217198	3	10	6.69	23.92	1.838139274	Up	0.0422656	0.325942593
331474	3	10	4.63	16.55	1.837747118	Up	0.0422656	0.323543751
94045	3	10	8.81	31.49	1.837679833	Up	0.0422656	0.326244951
14623	3	10	9.52	34.02	1.837349662	Up	0.0422656	0.324140145

103889	3	10	12.47	44.56	1.837287768	Up	0.0422656	0.32503887
67568	3	10	12.48	44.59	1.837102265	Up	0.0422656	0.324439168
235435	3	10	9.25	33.04	1.836688416	Up	0.0422656	0.324738742
268373	3	10	28.43	101.5	1.835993723	Up	0.0422656	0.325640794
13555	11.48	37.93	28.36	100.81	1.829709319	Up	5.24E-05	0.00251956
68449	10	33	18.53	65.55	1.822732899	Up	0.000159499	0.006350189
106200	3	4	6.76	23.75	1.812832362	Up	0.65158	0.785085025
70640	5.29	17	4.07	14.04	1.786442236	Up	0.00651834	0.110018473
18125	2	6.42	1.37	4.72	1.784610966	Up	0.1476488	0.532085606
21815	1	4	4.93	16.88	1.775655352	Up	0.1876922	0.618284559
100101919	7	22	3.79	12.77	1.752488772	Up	0.00286392	0.060948026
18196	7	22	22.06	74.32	1.752317711	Up	0.00286392	0.060792547
218811	8	25	12.68	42.49	1.744568598	Up	0.001495894	0.038182006
108000	1.08	3.34	0.64	2.14	1.741466986	Up	0.332862	0.689334172
268294	4.12	12.84	10.86	36.24	1.738558852	Up	0.0346988	0.29796565
234854	2	6	8.16	26.87	1.719355264	Up	0.1476488	0.536734672
319171	3.35	10.29	53.39	175.41	1.716089543	Up	0.0422656	0.326547871
67636	5	13	20.89	68.45	1.712238049	Up	0.0447088	0.33728189
16660	1	3.01	4.29	13.85	1.690836423	Up	0.332862	0.693650063
70574	1	3	1.28	4.13	1.689997971	Up	0.332862	0.685919936
12162	1	3	1.82	5.87	1.689422053	Up	0.332862	0.686089844
268721	1	3	1.11	3.58	1.689399911	Up	0.332862	0.69036508
277250	2	6	1.87	6.03	1.689119732	Up	0.1476488	0.534400028
56843	1	3	1.52	4.9	1.688710426	Up	0.332862	0.699253901
330836	1	3	1.79	5.77	1.688611731	Up	0.332862	0.684563693

74121	1	3	2.33	7.51	1.688482953	Up	0.332862	0.685750112
76338	1	3	2.42	7.8	1.688467076	Up	0.332862	0.68422547
74134	1	3	2.56	8.25	1.688250309	Up	0.332862	0.695042585
12385	1	3	1.8	5.8	1.688055994	Up	0.332862	0.699783907
218038	1	3	2.07	6.67	1.688055994	Up	0.332862	0.69226311
74159	1	3	1.85	5.96	1.68778706	Up	0.332862	0.701023716
231214	1	3	1.22	3.93	1.687648165	Up	0.332862	0.696966457
73251	2	6	1.81	5.83	1.687506186	Up	0.1476488	0.533240306
240869	1	3	1.72	5.54	1.687477411	Up	0.332862	0.695915754
18711	1	3	0.59	1.9	1.687212559	Up	0.332862	0.69399767
105859	1	3	2.68	8.63	1.687127559	Up	0.332862	0.690881692
53323	1	3	1.41	4.54	1.686997135	Up	0.332862	0.688819871
16905	1	3	3.23	10.4	1.686977458	Up	0.332862	0.687793569
72479	1	3	2.55	8.21	1.686884975	Up	0.332862	0.684056484
30841	5	16	7.56	24.34	1.686871028	Up	0.01073362	0.149605447
100038862	1	3	3.19	10.27	1.686807853	Up	0.332862	0.68847743
18803	2	6	3.01	9.69	1.686733179	Up	0.1476488	0.532316146
13601	1	3	3.56	11.46	1.686657898	Up	0.332862	0.700314716
69663	1	3	1.37	4.41	1.686602763	Up	0.332862	0.691744431
71860	1	3	3.06	9.85	1.686592072	Up	0.332862	0.698020338
73754	1	3	2.97	9.56	1.686547687	Up	0.332862	0.69943048
218543	2	6	3.29	10.59	1.6865431	Up	0.1476488	0.534865331
56214	1	3	3.75	12.07	1.686463175	Up	0.332862	0.690537198
217316	2	6	4.44	14.29	1.686374335	Up	0.1476488	0.531625125
57743	2	6	5.68	18.28	1.686303236	Up	0.1476488	0.532546885
71941	1	3	3.62	11.65	1.686268352	Up	0.332862	0.701378755

73166	1	3	4.54	14.61	1.686191976	Up	0.332862	0.688306337
21950	1	3	5.55	17.86	1.686172404	Up	0.332862	0.699077411
140743	1	3	3.58	11.52	1.686109224	Up	0.332862	0.690021102
20203	1	3	4.04	13	1.686084425	Up	0.332862	0.700669037
229715	6	18	7.21	23.2	1.686053641	Up	0.0089358	0.136681603
224742	2	6	4.18	13.45	1.686031325	Up	0.1476488	0.533471848
328643	1	3	1.47	4.73	1.686024029	Up	0.332862	0.68813533
66510	1	3	4.87	15.67	1.686011502	Up	0.332862	0.689677466
56708	1	3	3.63	11.68	1.685998821	Up	0.332862	0.699960754
215446	1	3	1.93	6.21	1.685992421	Up	0.332862	0.691399077
237781	1	3	2.62	8.43	1.685965819	Up	0.332862	0.692436176
547253	1	3	0.92	2.96	1.68589141	Up	0.332862	0.693302804
68857	1	3	2.35	7.56	1.685725478	Up	0.332862	0.695566224
12579	2	6	9.77	31.43	1.685711805	Up	0.1476488	0.535564806
72399	1	3	1.89	6.08	1.685685089	Up	0.332862	0.692609328
21750	1	3	2.72	8.75	1.685676365	Up	0.332862	0.697493
67134	1	3	3.55	11.42	1.685671721	Up	0.332862	0.694345626
13197	1	3	5.58	17.95	1.685646817	Up	0.332862	0.694171605
66116	1	3	6.69	21.52	1.685599962	Up	0.332862	0.694693931
11472	2	6	4.34	13.96	1.685531994	Up	0.1476488	0.533008965
209212	1	3	2.54	8.17	1.685507581	Up	0.332862	0.691054067
20962	1	3	6.05	19.46	1.685504663	Up	0.332862	0.701734153
105651	1	3	3.65	11.74	1.685464039	Up	0.332862	0.698548475
56226	2	6	12.2	39.24	1.685443894	Up	0.1476488	0.533935534
14225	2	6	8.15	26.21	1.685245389	Up	0.1476488	0.536969259
106529	1	3	5.98	19.23	1.685141373	Up	0.332862	0.691226529

11811	1	3	5.61	18.04	1.685126663	Up	0.332862	0.69644071
16206	2	6	2.69	8.65	1.68509396	Up	0.1476488	0.536266113
229504	1	3	2.32	7.46	1.685050825	Up	0.332862	0.692955892
26961	1	3	8.03	25.82	1.685017108	Up	0.332862	0.69347639
22634	4	12	5.06	16.27	1.685004961	Up	0.0346988	0.297658469
214663	4	12	13.76	44.24	1.684870916	Up	0.0346988	0.298581918
14979	1	3	7.02	22.57	1.684863483	Up	0.332862	0.691917238
18295	1	3	2.65	8.52	1.684861071	Up	0.332862	0.69890101
71640	1	3	2	6.43	1.684818738	Up	0.332862	0.689849241
18046	2	6	7.21	23.18	1.684809402	Up	0.1476488	0.535098286
14369	3	9	4.14	13.31	1.684807898	Up	0.0703334	0.427810103
107823	6	18	5.77	18.55	1.684775963	Up	0.0089358	0.136933318
69639	2	6	10.75	34.56	1.684766558	Up	0.1476488	0.53416768
234723	1	3	3.77	12.12	1.68475327	Up	0.332862	0.690709402
18220	1	3	2.56	8.23	1.68474862	Up	0.332862	0.700491832
51793	2	6	10.3	33.11	1.684622673	Up	0.1476488	0.535798371
20918	2	6	11	35.36	1.684614751	Up	0.1476488	0.531395184
19177	2	6	14.22	45.71	1.684588354	Up	0.1476488	0.532777825
67774	1	3	4.15	13.34	1.684575425	Up	0.332862	0.690193048
15528	1	3	8.82	28.35	1.684498174	Up	0.332862	0.694868214
19207	3	9	3.92	12.6	1.684498174	Up	0.0703334	0.426873976
14873	1	3	5.74	18.45	1.684498174	Up	0.332862	0.687281564
14670	2	6	4.9	15.75	1.684498174	Up	0.1476488	0.531855266
22342	1	3	9.34	30.02	1.684429522	Up	0.332862	0.687452147
11910	1	3	3.41	10.96	1.684404154	Up	0.332862	0.686259837
18619	1	3	4.72	15.17	1.684362321	Up	0.332862	0.696615871

100043272	1	3	2.2	7.07	1.684206691	Up	0.332862	0.693129305
11883	1	3	1.92	6.17	1.684164178	Up	0.332862	0.69539159
15207	1	3	3.7	11.89	1.684151539	Up	0.332862	0.685410716
69882	2	6	4.64	14.91	1.684083547	Up	0.1476488	0.535331444
27027	1	3	4.5	14.46	1.684070646	Up	0.332862	0.685580372
66167	4	12	44.13	141.8	1.68402588	Up	0.0346988	0.298273466
106877	1	3	1.97	6.33	1.68400987	Up	0.332862	0.689162653
16418	1	3	4.55	14.62	1.684004861	Up	0.332862	0.689505776
68816	1	3	5.63	18.09	1.683985581	Up	0.332862	0.697317397
67922	1	3	3.52	11.31	1.683951595	Up	0.332862	0.694519735
228366	1	3	2.87	9.22	1.683716014	Up	0.332862	0.697668691
66606	1	3	3.63	11.66	1.683526335	Up	0.332862	0.687964407
279029	1	3	3.07	9.86	1.683348991	Up	0.332862	0.700846331
66315	1	3	2.08	6.68	1.683264574	Up	0.332862	0.698196295
55949	1	3	3.5	11.24	1.683215208	Up	0.332862	0.70013769
102339	1	3	2.42	7.77	1.682907551	Up	0.332862	0.699607149
66884	1	3	2.8	8.99	1.682894289	Up	0.332862	0.685241143
100188919	1	3	3.18	10.21	1.682884196	Up	0.332862	0.688648608
13829	1	3	1.71	5.49	1.682809824	Up	0.332862	0.68694065
77832	1	3	2.33	7.48	1.682708315	Up	0.332862	0.688991219
72729	1	3	2.14	6.87	1.682699302	Up	0.332862	0.68439454
18601	1	3	2.19	7.03	1.682593819	Up	0.332862	0.68473293
270163	3	9	1.67	5.36	1.682384898	Up	0.0703334	0.427185563
230737	1	3	2.87	9.21	1.682150419	Up	0.332862	0.692782567

11307	1	3	1.15	3.69	1.681986955	Up		0.332862	0.697141883
70533	1	3	2.16	6.93	1.68182404	Up		0.332862	0.687111065
67075	7.23	21.62	11.75	37.67	1.680755276	Up		0.00461402	0.090550143
269037	1	3	1.08	3.46	1.679740725	Up		0.332862	0.695217044
20926	1	3	1.08	3.46	1.679740725	Up		0.332862	0.693823823
75410	1	3	0.79	2.53	1.679212827	Up		0.332862	0.696265637
105988	1	3	1.01	3.23	1.677178872	Up		0.332862	0.686600075
320207	7	20.88	10.73	34.29	1.676137828	Up		0.00736542	0.119005165
327959	5.22	15.57	15.21	48.6	1.675936161	Up		0.01748392	0.199566116
76795	1	3	1.28	4.07	1.668884984	Up		0.332862	0.69837234
20652	4.82	14.29	6.73	21.38	1.667583443	Up		0.01269428	0.16175973
22275	6	17.58	27.39	85.99	1.650519629	Up		0.01431542	0.178321272
98660	62.08	181.69	66.46	208.57	1.649973462	Up		9.05E-17	2.60E-14
23888	20.02	58.49	19.76	61.83	1.645724059	Up		3.07E-06	0.000210935
19300	6.4	18.67	17.04	53.28	1.644668747	Up		0.0089358	0.137185963
79566	1	3	2.23	6.96	1.642043596	Up		0.332862	0.691571711
72133	2.07	5.99	3.22	9.98	1.631979127	Up		0.245598	0.669162069
14128	7.97	23	26.41	81.7	1.629251777	Up		0.001762438	0.043907924
101497	2	6	2.63	8.12	1.626416928	Up		0.1476488	0.534632578
69097	8	23	29.36	90.47	1.623587503	Up		0.00382204	0.078140528
14339	17	49	53.77	165.6	1.622829296	Up		1.98E-05	0.001077282
77097	16.5	47.19	9.24	28.35	1.617383978	Up		2.36E-05	0.001251192
20540	7	20	22.21	68.02	1.614749613	Up		0.00736542	0.119469122
16004	7	20	5.21	15.95	1.614201146	Up		0.00736542	0.119236692
67839	2	6	4.18	12.72	1.605523823	Up		0.1476488	0.53370359
109711	6	17	10.74	32.63	1.603204994	Up		0.01431542	0.178054723

12890	73.9	206.91	100.13	300.6	1.585970724	Up	3.85E-18	1.33E-15
20466	5	14	6.68	20.05	1.585682229	Up	0.0281424	0.265803531
72290	12.96	35.98	13.39	39.85	1.573423764	Up	0.000292726	0.010453962
76051	12	33	16.63	49.05	1.560464968	Up	0.000717824	0.021799319
19252	4	11	13.84	40.81	1.560078768	Up	0.0562194	0.360679744
213948	4	11	6.86	20.22	1.559502516	Up	0.0562194	0.36040187
14201	4	11	16.09	47.42	1.559331337	Up	0.0562194	0.360124424
116733	11	30	35.53	103.86	1.547530537	Up	0.00135757	0.036795896
331524	7	19	16.21	47.18	1.541291328	Up	0.0116419	0.158029771
68828	7	19	23.18	67.46	1.541151752	Up	0.0116419	0.156751213
18611	7	19	19.01	55.32	1.541042625	Up	0.0116419	0.157260146
252837	7	19	22.39	65.15	1.540910651	Up	0.0116419	0.157772394
216971	7	19	12.06	35.09	1.540830041	Up	0.0116419	0.15828799
105000	3	8.12	3.45	10.03	1.539653339	Up	0.1150778	0.501867072
381820	2	2	5.36	15.57	1.538464039	Up	0.93456	0.976575883
12722	22.74	61.5	39.03	113.15	1.535581217	Up	3.33E-06	0.000223479
242557	5.99	17.27	14.09	40.55	1.525030303	Up	0.00651834	0.109795763
72338	3	8.02	6.75	19.35	1.519374159	Up	0.1150778	0.499250456
56191	3	8	2.79	7.99	1.517930381	Up	0.1150778	0.499771594
238247	3	8	3.46	9.9	1.516656487	Up	0.1150778	0.500293821
67155	3	8	3.43	9.81	1.51604456	Up	0.1150778	0.498730403
52064	3	8	10.12	28.94	1.515855632	Up	0.1150778	0.501341557
17356	3	8	2.7	7.72	1.51564144	Up	0.1150778	0.501079212
226823	9	24	16.27	46.52	1.515636846	Up	0.00491522	0.095115222
56048	3	8	8	22.87	1.515384461	Up	0.1150778	0.501604177
23806	3	8	3.11	8.89	1.515268839	Up	0.1150778	0.498470783

19704	3	8	4.36	12.46	1.514904028	Up	0.1150778	0.500032571
11490	3	8	7.05	20.14	1.514368521	Up	0.1150778	0.500817141
13644	3	8	5.63	16.08	1.514060579	Up	0.1150778	0.499510889
83602	6	16	7.03	20.06	1.512725012	Up	0.022677	0.23440412
13844	3	8	1.83	5.22	1.512206158	Up	0.1150778	0.498990294
66595	4	10	16.56	47.22	1.511695369	Up	0.0896688	0.45302616
52231	39	72	129.9	367.09	1.498732383	Up	0.00045271	0.015502057
13423	1	2.62	4.13	11.61	1.491154285	Up	0.573542	0.731857534
233490	5	13	6.34	17.68	1.479563529	Up	0.0447088	0.336063166
217356	2	5	4.81	13.41	1.479200438	Up	0.245598	0.668724135
51938	5	13	9.26	25.81	1.478846043	Up	0.0447088	0.336367021
100503915	70	182	257.58	717.89	1.478742223	Up	1.25E-14	3.07E-12
75422	5	13	35.48	98.83	1.477943031	Up	0.0447088	0.336976381
109754	22	57	71.86	199.58	1.473706314	Up	1.83E-05	0.001008923
94227	7	18	6.71	18.52	1.464699427	Up	0.01820672	0.205282002
319197	7	18	16.29	44.92	1.463371324	Up	0.01820672	0.205560539
11842	7.7	19.71	14.81	40.66	1.457038575	Up	0.0116419	0.157005267
380712	10	28	56.57	155.11	1.455182624	Up	0.001650114	0.041357225
14165	9	23	13.15	36.04	1.454536212	Up	0.00761144	0.121563901
20308	8.48	21.61	18.92	51.67	1.449414795	Up	0.00942674	0.14082568
22017	7.64	19.32	18.9	51.26	1.439447244	Up	0.0116419	0.157515853
218977	3.96	10	8.97	24.26	1.43539966	Up	0.0422656	0.325339554
237806	2	5	0.96	2.59	1.431845787	Up	0.245598	0.672686293
238055	2	5	0.95	2.56	1.430144392	Up	0.245598	0.671360367
69367	48.54	121.9	94.39	254.14	1.428917535	Up	8.37E-10	1.18E-07

233805	2	5	2.37	6.37	1.426406313	Up	0.245598	0.672243736
18214	2	5	1.6	4.3	1.426264755	Up	0.245598	0.67290779
319148	1.2	3	17.84	47.93	1.425813325	Up	0.332862	0.686429914
213783	2	5	1.85	4.97	1.425720581	Up	0.245598	0.673573157
74480	2	5	1.94	5.21	1.42522672	Up	0.245598	0.671139888
70408	2	5	3.21	8.62	1.425114572	Up	0.245598	0.672022676
16796	2	5	3.9	10.46	1.423336822	Up	0.245598	0.675353919
211660	2	5	3.05	8.18	1.4232916	Up	0.245598	0.674907846
68094	4	10	5.78	15.5	1.423126818	Up	0.0896688	0.452202476
73379	10	25	10.27	27.54	1.423092378	Up	0.0061341	0.105024375
12330	2	5	3.14	8.42	1.423055674	Up	0.245598	0.674685031
217366	2	5	5.43	14.56	1.422986252	Up	0.245598	0.672464942
20475	4	10	9.41	25.23	1.422873578	Up	0.0896688	0.452751265
110750	4	10	7.43	19.92	1.422783532	Up	0.0896688	0.45192858
20425	4	10	13.01	34.88	1.422779078	Up	0.0896688	0.451655015
73671	4	10	13.1	35.12	1.422726033	Up	0.0896688	0.451381781
68099	2	5	7.08	18.98	1.422658727	Up	0.245598	0.674239841
15288	2	5	8.74	23.43	1.422651769	Up	0.245598	0.674462362
78938	8	20	18.23	48.87	1.422634543	Up	0.01458784	0.18090226
108705	2	5	5.92	15.87	1.422633047	Up	0.245598	0.669600576
11480	12	30	14.09	37.77	1.422569172	Up	0.00261848	0.060023064
106894	10	25	13.05	34.98	1.422480482	Up	0.0061341	0.10524092
216516	2	5	2.66	7.13	1.422475831	Up	0.245598	0.675577176
71198	2	5	5.32	14.26	1.422475831	Up	0.245598	0.668286775
66048	2	5	10.07	26.99	1.422361293	Up	0.245598	0.669820045
269356	2	5	4.19	11.23	1.422335779	Up	0.245598	0.670919553

17937	8	20	22.43	60.11	1.4221754	Up	0.01458784	0.18063306
93759	6	15	10.59	28.38	1.422172	Up	0.0354848	0.301911064
329739	2	5	2.34	6.27	1.421956913	Up	0.245598	0.673351222
21411	2	5	1.84	4.93	1.42188188	Up	0.245598	0.673795238
68581	2	5	3.8	10.18	1.421666238	Up	0.245598	0.670039658
226866	2	5	4.89	13.1	1.421660441	Up	0.245598	0.671801761
114893	2	5	2.99	8.01	1.421656758	Up	0.245598	0.670259416
60367	4	9.99	4.92	13.18	1.42162015	Up	0.1405032	0.588388086
68332	2	5	13.95	37.37	1.421615442	Up	0.245598	0.668505384
78892	2	5	3.2	8.57	1.421223299	Up	0.245598	0.673129433
30795	2	5	14.6	39.1	1.421200238	Up	0.245598	0.675130809
20655	2	5	21.22	56.82	1.420974175	Up	0.245598	0.674017466
387314	2	5	1.6	4.28	1.419538892	Up	0.245598	0.669381251
14391	4	10	18.2	48.52	1.4146411	Up	0.0896688	0.452476704
12572	112.9	279.89	98.76	262.56	1.410648402	Up	2.21E-20	8.75E-18
14385	2	5	6.51	17.14	1.396637661	Up	0.245598	0.670479317
108011	4.22	10.32	4.31	11.31	1.391839155	Up	0.0896688	0.453576951
56421	16	39	26.53	69.35	1.386271207	Up	0.000741428	0.022112625
23832	35.71	86.94	53.58	139.88	1.384423215	Up	4.04E-07	3.46E-05
73694	22.88	55.69	66.9	174.62	1.384140691	Up	4.18E-05	0.002060191
23874	7	17	22.59	58.8	1.380131884	Up	0.0281488	0.265562545
78688	26.03	63	60.92	158.07	1.375575739	Up	1.85E-05	0.001010324
140795	2	4	6.95	17.98	1.371308138	Up	0.397842	0.788200781
20977	27	65	73.18	188.88	1.367948626	Up	1.50E-05	0.000855823
227612	51.89	124.76	101.41	261.42	1.366169596	Up	1.52E-09	2.08E-07
23983	5	12	19.79	50.92	1.363460807	Up	0.070006	0.427380723

66089	5	12	17.36	44.66	1.363216303	Up	0.070006	0.427067394
15312	5	12	28.49	73.28	1.362963882	Up	0.070006	0.426442113
78796	2.01	2	2.33	5.99	1.362226048	Up	0.93456	0.988995773
69870	0.83	2	4.52	11.62	1.362215391	Up	0.224708	0.669937394
27370	5	12	78.66	202.07	1.361153059	Up	0.070006	0.426754525
16672	2.01	4.8	8.45	21.68	1.35934151	Up	0.397842	0.782425734
270627	7.97	18.9	6.61	16.8	1.345739056	Up	0.01820672	0.205839833
269952	2.61	6.15	4.96	12.55	1.339275338	Up	0.1476488	0.53603214
338366	3.99	9.35	3.84	9.64	1.327926836	Up	0.0703334	0.428123059
81702	3	7	2.14	5.36	1.324622204	Up	0.1846234	0.61771263
214812	6	14	4.64	11.62	1.324413358	Up	0.0547886	0.352587734
234663	3	7	4.31	10.79	1.32393509	Up	0.1846234	0.619956139
545471	337.07	787.04	836.94	2095.1	1.323823001	Up	2.55E-49	3.54E-46
208659	3	7	7.71	19.3	1.323798082	Up	0.1846234	0.618957015
67398	3	7	6.86	17.17	1.323609558	Up	0.1846234	0.616473239
22339	3	7	6.01	15.04	1.323367671	Up	0.1846234	0.615485301
18822	3	7	6.15	15.39	1.323334916	Up	0.1846234	0.619456174
243374	3	7	6.37	15.94	1.323286352	Up	0.1846234	0.619706055
13667	3	7	9.55	23.89	1.322834216	Up	0.1846234	0.61845866
277010	6	14	13.08	32.72	1.322810207	Up	0.0547886	0.352860635
234371	3	7	6.88	17.21	1.322766627	Up	0.1846234	0.620206424
12388	3	7	3.97	9.93	1.32265471	Up	0.1846234	0.617464353
24084	3	7	13.59	33.99	1.322564906	Up	0.1846234	0.619206494
19708	3	7	6.07	15.18	1.322403369	Up	0.1846234	0.615238811
14451	3	7	6.39	15.98	1.322379572	Up	0.1846234	0.617216276
14696	3	7	7.33	18.33	1.322321682	Up	0.1846234	0.614746423

67442	3	7	10.44	26.1	1.321928095	Up	0.1846234	0.620456911
69454	3	7	26.08	65.2	1.321928095	Up	0.1846234	0.615978874
18643	3	7	23.5	58.74	1.321682509	Up	0.1846234	0.616225957
69743	3	7	2.51	6.27	1.320778079	Up	0.1846234	0.616720719
19982	3	7	43.12	107.71	1.320723115	Up	0.1846234	0.615731989
74413	3	8	4.32	10.78	1.319253961	Up	0.1150778	0.500555344
66209	3	6.92	6.24	15.45	1.307988904	Up	0.289442	0.627199709
83796	1	2	2.43	6.01	1.306408677	Up	0.573542	0.727395669
66970	10	23	10.15	25.03	1.302178564	Up	0.01408234	0.176474625
319200	10	23	26.94	66.43	1.302085064	Up	0.01408234	0.17620925
77038	7	16	13.93	34.14	1.2932678	Up	0.0429834	0.32723227
240913	7	16	12.76	31.27	1.29315089	Up	0.0429834	0.326933155
56217	7	16	8.45	20.7	1.292607521	Up	0.0429834	0.327531934
13870	5	11	30.3	73.56	1.279603686	Up	0.107869	0.518531455
20913	5.07	11.45	5.6	13.57	1.276921988	Up	0.107869	0.517037989
241066	1.51	3.39	1.9	4.59	1.272494735	Up	0.332862	0.696090651
15024	4	9	9.39	22.66	1.270950798	Up	0.1405032	0.588092116
231871	4	9	8.23	19.86	1.270901287	Up	0.1405032	0.586616722
16407	4	9	7.02	16.94	1.270890939	Up	0.1405032	0.586028635
12457	4	9	8.74	21.09	1.27085391	Up	0.1405032	0.587501069
227738	8	18	13.37	32.26	1.270746973	Up	0.0337982	0.291737367
26379	4	9	11.9	28.71	1.270591757	Up	0.1405032	0.586911208
67801	4	9	14.31	34.52	1.270408792	Up	0.1405032	0.585735034
75735	4	9	3.59	8.66	1.270383181	Up	0.1405032	0.587796444
226562	4	9	2.55	6.15	1.270089163	Up	0.1405032	0.587205991
268395	8	18	31.04	74.86	1.270066491	Up	0.0337982	0.292040314

14863	4	9	24.45	58.96	1.269902059	Up	0.1405032	0.586322531
225912	5.1	13.4	12.89	30.88	1.260420489	Up	0.0447088	0.336671425
270906	32.43	72.29	56.7	135.53	1.257191592	Up	1.67E-05	0.00093258
17355	9	20	7.19	17.14	1.253303434	Up	0.0266304	0.253247495
22414	18	40	33.73	80.38	1.252804259	Up	0.001570006	0.039708267
66067	10	20	55.31	131.51	1.249560259	Up	0.0453252	0.339164559
17281	202.14	447.84	172.13	408.93	1.248355344	Up	2.69E-26	1.49E-23
99662	14	31	40.76	96.77	1.24740581	Up	0.00565008	0.101323956
231855	0.9	2	1.59	3.77	1.245537758	Up	0.224708	0.675260118
73473	9.72	21.45	6.72	15.9	1.242493627	Up	0.01772814	0.201800072
12339	10	22	18.8	44.35	1.238201443	Up	0.0210216	0.218650917
71943	10	22	32.23	76.02	1.237974837	Up	0.0210216	0.219198914
70227	5	11	5.58	13.16	1.237822462	Up	0.107869	0.517634342
211007	10	22	19.18	45.23	1.237677276	Up	0.0210216	0.218924573
106393	5	11	6.71	15.82	1.237364928	Up	0.107869	0.517335994
19265	5	11	40.6	95.7	1.237039197	Up	0.107869	0.518232072
67848	5.04	11	12.06	28.23	1.226999222	Up	0.107869	0.517933035
110524	11	24	15.93	37.28	1.226655593	Up	0.0166218	0.191035909
14082	10.38	22.52	24.27	56.47	1.218310523	Up	0.0210216	0.218377945
72108	6	13	9.4	21.84	1.216240194	Up	0.0833636	0.444659305
227358	6	13	14.21	33.01	1.215996584	Up	0.0833636	0.445230113
109905	6	13	16.32	37.9	1.215556791	Up	0.0833636	0.444944526
56868	13.89	29.94	42.97	99.31	1.20860922	Up	0.00709108	0.115923137
56210	13	28	20.31	46.9	1.207397683	Up	0.01043738	0.145720535
67469	7	15	14.91	34.26	1.200244894	Up	0.0647598	0.400644086
19208	4.71	10.1	25.06	57.58	1.200181374	Up	0.0896688	0.453301388

68691	14	30	20.21	46.43	1.199987959	Up	0.00828656	0.132092846
68178	1	2	0.92	2.11	1.197537233	Up	0.573542	0.723206998
237038	8	17	20.99	47.82	1.187911963	Up	0.0505182	0.362068856
27205	8	17	10.02	22.82	1.187416283	Up	0.0505182	0.362380985
71279	9	19	11.46	25.95	1.179127494	Up	0.0395448	0.308969278
14961	9	19	51.59	116.73	1.178012035	Up	0.0395448	0.308679438
211922	9.64	20.79	9.87	22.28	1.174627243	Up	0.0266304	0.253537252
57317	19	40	56.8	128.18	1.17420834	Up	0.0026533	0.060654146
269604	24.19	50.77	33.93	76.35	1.170066727	Up	0.000865504	0.025006454
16192	15	31.32	28.24	63.22	1.162640947	Up	0.00955148	0.14243345
338354	2.88	6.02	3.49	7.81	1.162095512	Up	0.1476488	0.53650029
100503659	23	48	51.83	115.96	1.16176794	Up	0.001081584	0.03030256
98910	1	2	1.38	3.07	1.153570389	Up	0.573542	0.730177935
18193	27	56	14.04	31.22	1.152927602	Up	0.000444816	0.015294686
26378	4	8.24	13.16	29.06	1.142875214	Up	0.215752	0.681576459
14026	3	7	10.72	23.66	1.142145168	Up	0.1846234	0.618707737
229595	18	37	30.58	67.39	1.139946119	Up	0.00479138	0.093370194
385354	19	39	27.31	60.11	1.138175706	Up	0.003815	0.078188707
230379	16.61	33.96	96.7	211.94	1.132068102	Up	0.00758062	0.121304498
14160	6.35	12.95	9	19.68	1.128733314	Up	0.1248166	0.535084456
12212	0.51	1.04	0.46	1	1.120294234	Up	0.46568	0.811162504
11819	2	4	3.17	6.87	1.115827259	Up	0.397842	0.783907952
12828	1	2	0.77	1.66	1.108252891	Up	0.573542	0.730401436
50766	1	2	1.11	2.39	1.106450942	Up	0.573542	0.716152908
21812	1	2	1.18	2.54	1.106041637	Up	0.573542	0.721457745
114715	1	2	1.05	2.26	1.105933445	Up	0.573542	0.72917387

192285	1	2	1.05	2.26	1.105933445	Up	0.573542	0.721785085
79263	1	2	2.18	4.69	1.105259788	Up	0.573542	0.727063221
16500	2	4	1.19	2.56	1.105182237	Up	0.397842	0.778378387
13631	1	2	1.06	2.28	1.10496956	Up	0.573542	0.725515808
230872	1	2	1.06	2.28	1.10496956	Up	0.573542	0.722112722
67306	1	2	1.99	4.28	1.104842366	Up	0.573542	0.727617469
320271	3	6	1.86	4	1.104697379	Up	0.289442	0.627853723
108150	1	2	1.53	3.29	1.104555931	Up	0.573542	0.718849673
20591	1	2	0.6	1.29	1.10433666	Up	0.573542	0.720695105
19249	1	2	0.8	1.72	1.10433666	Up	0.573542	0.729285297
80890	2	4	1.87	4.02	1.104157231	Up	0.397842	0.786702301
13846	1	2	1.54	3.31	1.103900866	Up	0.573542	0.720912837
320916	1	2	1.54	3.31	1.103900866	Up	0.573542	0.716475451
227696	6	10	27.54	59.19	1.103824897	Up	0.264802	0.694865166
26943	1	2	1.81	3.89	1.103780458	Up	0.573542	0.721130701
12454	1	2	2.55	5.48	1.103678646	Up	0.573542	0.722878367
14766	1	2	1.88	4.04	1.103622631	Up	0.573542	0.732756484
228410	1	2	2.35	5.05	1.103622631	Up	0.573542	0.724304596
320438	1	2	2.15	4.62	1.103556192	Up	0.573542	0.730848849
66505	1	2	1.68	3.61	1.103537604	Up	0.573542	0.727506552
381605	1	2	1.55	3.33	1.103253962	Up	0.573542	0.732869008
269536	1	2	2.09	4.49	1.103212503	Up	0.573542	0.716798285
18458	1	2	2.36	5.07	1.103198888	Up	0.573542	0.730289668
11765	3	6	2.9	6.23	1.103179263	Up	0.289442	0.628509103
14758	2	4	2.97	6.38	1.103093493	Up	0.397842	0.789704981
227720	1	2	0.81	1.74	1.103093493	Up	0.573542	0.72961978

233107	1	2	2.7	5.8	1.103093493	Up	0.573542	0.720368752
71990	1	2	1.69	3.63	1.102946302	Up	0.573542	0.731072761
67412	1	2	1.69	3.63	1.102946302	Up	0.573542	0.726399236
319710	1	2	1.42	3.05	1.102918313	Up	0.573542	0.73309416
228140	1	2	1.15	2.47	1.102877181	Up	0.573542	0.721021753
214112	1	2	2.03	4.36	1.102848408	Up	0.573542	0.728172564
71835	1	2	1.83	3.93	1.102685664	Up	0.573542	0.731184768
53890	2	4	3.73	8.01	1.102626612	Up	0.397842	0.786515391
20403	3	6	3.33	7.15	1.102421065	Up	0.289442	0.627526546
56546	1	2	2.72	5.84	1.102361718	Up	0.573542	0.720803954
15478	2	4	3.4	7.3	1.102361718	Up	0.397842	0.786328571
330369	1	2	1.02	2.19	1.102361718	Up	0.573542	0.727950424
277463	1	2	1.7	3.65	1.102361718	Up	0.573542	0.715401436
13854	1	2	2.79	5.99	1.102290881	Up	0.573542	0.718200599
68877	1	2	4.09	8.78	1.102120097	Up	0.573542	0.732082065
208043	1	2	0.75	1.61	1.102098188	Up	0.573542	0.730066236
268935	1	2	2.25	4.83	1.102098188	Up	0.573542	0.727839406
50493	1	2	1.91	4.1	1.102051271	Up	0.573542	0.716367905
56735	1	2	3.07	6.59	1.10203981	Up	0.573542	0.724854645
14571	1	2	1.16	2.49	1.102020937	Up	0.573542	0.726841758
229700	1	2	2.05	4.4	1.101879614	Up	0.573542	0.724194686
14719	1	2	2.87	6.16	1.101879614	Up	0.573542	0.730960788
235344	2	4	3.76	8.07	1.101836012	Up	0.397842	0.783722368
71520	1	2	4.24	9.1	1.101802281	Up	0.573542	0.721675939
102595	1	2	1.3	2.79	1.101753499	Up	0.573542	0.715615982
67434	1	2	0.89	1.91	1.101695397	Up	0.573542	0.73129681

93842	2	4	3.22	6.91	1.101625022	Up	0.397842	0.780396813
241118	2	4	5.07	10.88	1.101620904	Up	0.397842	0.78989341
238673	1	2	1.92	4.12	1.101538026	Up	0.573542	0.73331945
17965	1	2	3.84	8.24	1.101538026	Up	0.573542	0.726178177
234023	1	2	3.91	8.39	1.101502203	Up	0.573542	0.734109057
56847	2	4	3.91	8.39	1.101502203	Up	0.397842	0.783536871
75146	1	2	2.95	6.33	1.101490545	Up	0.573542	0.723755381
22365	1	2	2.54	5.45	1.101427733	Up	0.573542	0.720477503
103554	1	2	1.03	2.21	1.101402032	Up	0.573542	0.717768534
235559	2	4	2.61	5.6	1.10137702	Up	0.397842	0.785209507
16398	1	2	1.58	3.39	1.101360715	Up	0.573542	0.722222001
29864	1	2	3.16	6.78	1.101360715	Up	0.573542	0.71454454
83397	2	4	2.13	4.57	1.101340735	Up	0.397842	0.782056055
171388	1	2	3.78	8.11	1.10131568	Up	0.573542	0.728394839
641340	1	2	3.78	8.11	1.10131568	Up	0.573542	0.717984502
232236	1	2	3.85	8.26	1.101283336	Up	0.573542	0.730736944
216831	1	2	1.65	3.54	1.101283336	Up	0.573542	0.719825487
233020	2	4	5.57	11.95	1.101261386	Up	0.397842	0.784837193
69885	1	2	5.37	11.52	1.101146723	Up	0.573542	0.719608411
60441	1	2	4.82	10.34	1.101131134	Up	0.573542	0.721894264
19210	5	10	9.78	20.98	1.101108308	Up	0.1632572	0.565555021
13358	2	4	7.99	17.14	1.101099701	Up	0.397842	0.77966163
72145	4	8	1.86	3.99	1.101086125	Up	0.215752	0.682094374
17684	2	4	6.82	14.63	1.101086125	Up	0.397842	0.789516642
12455	1	2	3.1	6.65	1.101086125	Up	0.573542	0.732194382
229949	3	6	6.48	13.9	1.101019165	Up	0.289442	0.62834513

14370	1	2	2	4.29	1.100977648	Up	0.573542	0.73253154
11538	1	2	1.38	2.96	1.100928909	Up	0.573542	0.726952473
207615	2	4	3.52	7.55	1.100901216	Up	0.397842	0.779845296
330361	12	24	23.82	51.09	1.100867523	Up	0.027928	0.264378712
100226	1	2	2.9	6.22	1.10086168	Up	0.573542	0.72628869
59016	1	2	3.66	7.85	1.100849005	Up	0.573542	0.716045459
30957	6	12	7.32	15.7	1.100849005	Up	0.1248166	0.534808923
277978	3	6	8.08	17.33	1.100844456	Up	0.289442	0.629494742
68646	2	4	3.73	8	1.10082437	Up	0.397842	0.781133384
241556	3	6	5.32	11.41	1.100800641	Up	0.289442	0.629165852
107650	11	20	23.56	50.53	1.100800641	Up	0.0725882	0.44023791
110521	1	2	0.76	1.63	1.100800641	Up	0.573542	0.724744568
70843	1	2	4.56	9.78	1.100800641	Up	0.573542	0.723097422
27375	1	2	2.28	4.89	1.100800641	Up	0.573542	0.714972731
75731	1	2	4.63	9.93	1.100781524	Up	0.573542	0.723865157
68728	5	10	8.5	18.23	1.100779815	Up	0.1632572	0.563911648
12523	8	16	16.31	34.98	1.100773507	Up	0.074507	0.405741327
78541	1	2	3.11	6.67	1.100772181	Up	0.573542	0.728283684
20168	1	2	2.35	5.04	1.100762977	Up	0.573542	0.732981567
56460	1	2	2.35	5.04	1.100762977	Up	0.573542	0.71658303
70976	1	2	3.94	8.45	1.100755712	Up	0.573542	0.71712141
110326	2	4	4.77	10.23	1.100744974	Up	0.397842	0.788388493
73447	1	2	1.59	3.41	1.100744974	Up	0.573542	0.719283042
52808	1	2	2.42	5.19	1.100727491	Up	0.573542	0.722003477
225372	1	2	3.25	6.97	1.100718938	Up	0.573542	0.720586287
22194	1	2	4.91	10.53	1.100710507	Up	0.573542	0.718416827

226182	4	8	8.23	17.65	1.100703848	Up	0.215752	0.683132569
433759	2	4	6.85	14.69	1.100658503	Up	0.397842	0.781686725
74243	1	2	2.63	5.64	1.100632363	Up	0.573542	0.725185076
78912	4	8	8.79	18.85	1.100629453	Up	0.215752	0.682872724
22682	1	2	0.9	1.93	1.100603941	Up	0.573542	0.71852499
257962	1	2	7.2	15.44	1.100603941	Up	0.573542	0.717660599
20276	2	4	3.81	8.17	1.100545081	Up	0.397842	0.784465233
80915	2	4	9.63	20.65	1.100534078	Up	0.397842	0.784279385
12164	3	6	6.79	14.56	1.100526876	Up	0.289442	0.630318472
246103	2	4	1.94	4.16	1.100526876	Up	0.397842	0.780580826
72023	3	6	4.92	10.55	1.100512778	Up	0.289442	0.627036418
11771	2	4	3.95	8.47	1.100509316	Up	0.397842	0.781317744
20322	1	2	2.98	6.39	1.1005036	Up	0.573542	0.72540553
226098	5	10	7	15.01	1.10049715	Up	0.1632572	0.564849547
545893	2	4	12.2	26.16	1.100481393	Up	0.397842	0.781871347
622434	4	8	5.13	11	1.100472793	Up	0.215752	0.682613077
56643	1	2	2.15	4.61	1.100430091	Up	0.573542	0.724634525
57814	2	4	5.41	11.6	1.100424306	Up	0.397842	0.787263563
246703	1	2	7.63	16.36	1.100417786	Up	0.573542	0.721348697
76302	5	10	14.64	31.39	1.100389475	Up	0.1632572	0.564145831
56291	8	16	12.56	26.93	1.100377766	Up	0.074507	0.405475963
328401	1	2	5.76	12.35	1.100370325	Up	0.573542	0.733657645
30838	1	2	3.47	7.44	1.100366959	Up	0.573542	0.723974967
18107	1	2	3.68	7.89	1.100319534	Up	0.573542	0.716690642
97863	1	2	4.93	10.57	1.100315825	Up	0.573542	0.721239683
13177	2	4	12.57	26.95	1.100300623	Up	0.397842	0.782240851

100503670	19	38	62.99	135.05	1.100298924	Up	0.00544718	0.098108192
21991	3	6	12.71	27.25	1.1002922	Up	0.289442	0.629823975
14230	1	2	2.57	5.51	1.100283959	Up	0.573542	0.718633185
74718	1	2	2.64	5.66	1.100264123	Up	0.573542	0.715723303
78929	2	4	5.35	11.47	1.100254595	Up	0.397842	0.78576864
71446	1	2	2.71	5.81	1.100245312	Up	0.573542	0.727284819
52830	2	4	7.09	15.2	1.100213791	Up	0.397842	0.778561449
74577	14	28	27.18	58.27	1.100207855	Up	0.01736898	0.198526487
80901	11	22	39.28	84.21	1.100196635	Up	0.0355312	0.301997053
20856	7	14	26.42	56.64	1.100190799	Up	0.096154	0.479962468
193736	2	4	7.37	15.8	1.100188034	Up	0.397842	0.788952164
67773	2	4	9.04	19.38	1.100173893	Up	0.397842	0.787825626
66261	5	10	22.6	48.45	1.100173893	Up	0.1632572	0.564380208
386649	1	2	4.59	9.84	1.100164162	Up	0.573542	0.719391465
70375	8	13	15.58	33.4	1.100152869	Up	0.218144	0.681628323
13559	5	10	19.2	41.16	1.100136671	Up	0.1632572	0.563443866
20641	1	2	8.28	17.75	1.100116352	Up	0.573542	0.728617249
20733	1	2	5.22	11.19	1.100088324	Up	0.573542	0.731633141
66180	1	2	3.55	7.61	1.100077429	Up	0.573542	0.733544879
67011	1	2	3.55	7.61	1.100077429	Up	0.573542	0.732306733
74711	5	10	11.42	24.48	1.100040907	Up	0.1632572	0.56508451
22209	1	2	3.9	8.36	1.100028818	Up	0.573542	0.733770446
76893	3	6	9.89	21.2	1.100021839	Up	0.289442	0.628673162
13401	3	6	8.01	17.17	1.100015892	Up	0.289442	0.630153554
16432	5	10	19.09	40.92	1.099989042	Up	0.1632572	0.565319668
76936	4	8	11.15	23.9	1.099966908	Up	0.215752	0.682353627

14794	2	4	11.78	25.25	1.099943849	Up	0.397842	0.786141839
70021	2	4	9.48	20.32	1.099941438	Up	0.397842	0.785582174
13367	2	4	2.37	5.08	1.099941438	Up	0.397842	0.784651169
70207	3	6	15.13	32.43	1.099917036	Up	0.289442	0.629659316
75697	2	4	10.11	21.67	1.099916156	Up	0.397842	0.789328393
69640	2	4	2.79	5.98	1.099880362	Up	0.397842	0.788576294
105348	3	6	5.65	12.11	1.099876092	Up	0.289442	0.629001536
12390	1	2	6.14	13.16	1.099848928	Up	0.573542	0.718741413
17150	1	2	6.49	13.91	1.099832037	Up	0.573542	0.731969783
100503386	3	6	6.63	14.21	1.099825779	Up	0.289442	0.629330254
67231	1	2	3.49	7.48	1.099811234	Up	0.573542	0.716260391
67623	2	4	4.05	8.68	1.099773135	Up	0.397842	0.780764925
14693	1	2	4.26	9.13	1.09976143	Up	0.573542	0.725736463
68365	2	4	4.33	9.28	1.09975778	Up	0.397842	0.782980909
22321	3	6	4.75	10.18	1.099738143	Up	0.289442	0.62801744
215456	2	4	5.1	10.93	1.099724249	Up	0.397842	0.778927831
67702	2	4	5.52	11.83	1.099709902	Up	0.397842	0.779294558
15507	5	10	37.82	81.05	1.099662822	Up	0.1632572	0.56367766
75452	3	6	7.69	16.48	1.099660739	Up	0.289442	0.628181242
81500	2	4	7.9	16.93	1.099657415	Up	0.397842	0.779478051
233876	3	6	8.04	17.23	1.099655295	Up	0.289442	0.626873212
54633	4	8	26.09	55.91	1.099609401	Up	0.215752	0.681835318
21681	4	8	24.21	51.88	1.0995754	Up	0.215752	0.681317796
76889	1	2	3.08	6.6	1.099535674	Up	0.573542	0.732643995
12702	1	2	2.45	5.25	1.099535674	Up	0.573542	0.729731343
108112	3	6	17.85	38.25	1.099535674	Up	0.289442	0.628837306

71389	1	2	0.63	1.35	1.099535674	Up	0.573542	0.725957253
66625	2	4	2.94	6.3	1.099535674	Up	0.397842	0.785023306
215819	1	2	0.98	2.1	1.099535674	Up	0.573542	0.724964755
621239	1	2	3.15	6.75	1.099535674	Up	0.573542	0.724524515
74411	1	2	2.31	4.95	1.099535674	Up	0.573542	0.719499922
74106	1	2	2.03	4.35	1.099535674	Up	0.573542	0.719174651
666938	1	2	0.84	1.8	1.099535674	Up	0.573542	0.719066292
77006	1	2	6.23	13.35	1.099535674	Up	0.573542	0.718092534
15510	1	2	2.87	6.15	1.099535674	Up	0.573542	0.717444826
71458	1	2	0.98	2.1	1.099535674	Up	0.573542	0.71518702
238663	1	2	1.75	3.75	1.099535674	Up	0.573542	0.714758572
83924	1	2	2.24	4.8	1.099535674	Up	0.573542	0.71465154
216767	1	2	9.25	19.82	1.099431692	Up	0.573542	0.728728505
21753	3	6	8.27	17.72	1.099419369	Up	0.289442	0.629988721
66336	1	2	6.52	13.97	1.099388151	Up	0.573542	0.729396757
52024	1	2	3.86	8.27	1.099286482	Up	0.573542	0.717229183
50518	1	2	10.11	21.66	1.099250246	Up	0.573542	0.728061477
140917	2	4	3.23	6.92	1.099237873	Up	0.397842	0.783166142
18142	1	2	3.23	6.92	1.099237873	Up	0.573542	0.717336988
14453	1	2	3.16	6.77	1.099231275	Up	0.573542	0.723426252
77044	8	16	5.9	12.64	1.099209604	Up	0.074507	0.406007038
28146	1	2	2.74	5.87	1.09918461	Up	0.573542	0.718957967
12821	4	8	4.92	10.54	1.099144646	Up	0.215752	0.683392612
71609	1	2	4.92	10.54	1.099144646	Up	0.573542	0.72895112
109212	1	2	4.64	9.94	1.099121046	Up	0.573542	0.726509816
68521	1	2	2.18	4.67	1.099094415	Up	0.573542	0.730625074

29862	1	2	8.44	18.08	1.099079774	Up	0.573542	0.72276889
214301	1	2	11.47	24.57	1.099032466	Up	0.573542	0.725295286
16842	1	2	1.9	4.07	1.099029376	Up	0.573542	0.715830656
207920	1	2	1.69	3.62	1.098966451	Up	0.573542	0.722440657
67139	2	4	4.86	10.41	1.09894185	Up	0.397842	0.787638183
338337	2	4	3.17	6.79	1.098928734	Up	0.397842	0.781502191
329972	1	2	2.68	5.74	1.098817736	Up	0.573542	0.728839796
60321	2	4	4.94	10.58	1.098756681	Up	0.397842	0.787076387
381236	1	2	2.47	5.29	1.098756681	Up	0.573542	0.722331313
432555	1	2	2.26	4.84	1.098684275	Up	0.573542	0.733883282
16211	2	4	2.26	4.84	1.098684275	Up	0.397842	0.789140234
74574	2	4	4.17	8.93	1.098612792	Up	0.397842	0.786889299
20773	1	2	0.99	2.12	1.098563834	Up	0.573542	0.718308697
13115	1	2	2.76	5.91	1.098489863	Up	0.573542	0.729508252
20448	1	2	1.84	3.94	1.098489863	Up	0.573542	0.723316608
14694	1	2	5.45	11.67	1.098476426	Up	0.573542	0.724414539
66809	1	2	3.47	7.43	1.098426548	Up	0.573542	0.719716933
55983	1	2	1.63	3.49	1.098355072	Up	0.573542	0.715294212
21787	1	2	3.76	8.05	1.098256121	Up	0.573542	0.726731077
21767	1	2	3.55	7.6	1.098180394	Up	0.573542	0.721566825
93765	2	4	3.48	7.45	1.09815312	Up	0.397842	0.780029049
56873	1	2	1.35	2.89	1.098110085	Up	0.573542	0.72984294
11655	1	2	2.49	5.33	1.097989791	Up	0.573542	0.717552696
68626	1	2	2.42	5.18	1.09794505	Up	0.573542	0.726067698
14159	1	2	2.42	5.18	1.09794505	Up	0.573542	0.725074899
27401	1	2	2.14	4.58	1.097736802	Up	0.573542	0.731520997

74277	1	2	2.64	5.65	1.097712938	Up		0.573542	0.720260034
73729	1	2	2.57	5.5	1.097663259	Up		0.573542	0.727728421
11958	1	2	20.66	44.21	1.09753248	Up		0.573542	0.730513238
269181	2	4	1.86	3.98	1.097465809	Up		0.397842	0.778195412
13003	1	2	1.79	3.83	1.097384805	Up		0.573542	0.733206788
102774	1	2	2.65	5.67	1.097356375	Up		0.573542	0.72662043
66477	2	4	39.61	84.75	1.097348667	Up		0.397842	0.779111151
269702	1	2	0.86	1.84	1.097297201	Up		0.573542	0.715938041
14270	2	4	1.66	3.55	1.096635783	Up		0.397842	0.783351463
26385	1	2	2.31	4.94	1.09661819	Up		0.573542	0.720151348
279561	1	2	0.65	1.39	1.09657326	Up		0.573542	0.732419119
243547	1	2	1.3	2.78	1.09657326	Up		0.573542	0.719934075
12444	1	2	1.16	2.48	1.096215315	Up		0.573542	0.728506027
12029	1	2	1.96	4.19	1.09609659	Up		0.573542	0.729062478
77056	9	18	17.79	38.03	1.096071428	Up		0.0580022	0.362067747
230753	1	2	1.54	3.29	1.095157233	Up		0.573542	0.733996152
54189	1	2	1.26	2.69	1.094182439	Up		0.573542	0.727174003
26889	1	2	1.04	2.22	1.093976148	Up		0.573542	0.731408886
240168	1	2	1.42	3.03	1.093426864	Up		0.573542	0.725626119
213233	40.68	80.91	131.96	281.36	1.092316557	Up		5.12E-05	0.002490607
68744	1	2	1.7	3.62	1.090454951	Up		0.573542	0.717876502
67141	43	85	159.83	338.66	1.08329937	Up		3.73E-05	0.001857048
240354	9.22	18.22	12.3	26.04	1.082071133	Up		0.0580022	0.362339569
97064	1	2	1.42	3	1.079071571	Up		0.573542	0.716905961
12393	7	14.02	7.33	15.48	1.078520368	Up		0.096154	0.479674721
12000	3	4	11.81	24.88	1.074977521	Up		0.65158	0.784063222

240087	25.32	49.52	22.87	47.98	1.068976792	Up	0.001975692	0.048352156
109674	1	2	1.86	3.89	1.064467534	Up	0.573542	0.714865635
629059	9.58	18.56	18.04	37.46	1.054151561	Up	0.0580022	0.361796331
109685	10.02	19.39	37.03	76.84	1.053162969	Up	0.0653734	0.403839689
100048534	15	29	16.82	34.87	1.051808659	Up	0.01957846	0.216927251
231093	60	119	126.45	261.86	1.050228663	Up	9.49E-07	7.31E-05
216134	2.45	4.71	3.19	6.6	1.0489096	Up	0.397842	0.788764184
107448	2	4	3.49	7.22	1.048771801	Up	0.397842	0.780949111
56734	2	3	6.26	12.95	1.048717536	Up	0.623204	0.761368446
434197	12.47	24.01	29.58	61.07	1.045841793	Up	0.027928	0.264078282
72103	65.95	127	140.49	290.05	1.045834175	Up	6.26E-07	5.11E-05
140810	2.89	5.56	1.74	3.58	1.040872281	Up	0.245598	0.671580992
73106	132	253	329.36	676.78	1.039021581	Up	5.52E-12	1.12E-09
53379	12	23	37.73	77.51	1.038670352	Up	0.0400528	0.311767398
56322	3	6	7.63	15.67	1.038250218	Up	0.289442	0.627363085
217127	1	2	1.17	2.4	1.036525876	Up	0.573542	0.715079859
13411	22	42	10.39	21.27	1.033624379	Up	0.00546276	0.098176298
210673	11	21	19.85	40.62	1.033051232	Up	0.0510908	0.364915491
73068	21	40	42.08	85.94	1.030195072	Up	0.00686274	0.114209719
12725	2	4	2.54	5.18	1.028123601	Up	0.397842	0.780212888
192198	28	53	51.56	104.64	1.021110277	Up	0.00196722	0.048286837
21406	3	6	4.77	9.67	1.019526623	Up	0.289442	0.627690092
213573	11.58	21.87	46.5	94.18	1.018190006	Up	0.0510908	0.365228992
104923	9	17	36.38	73.68	1.018127518	Up	0.0839584	0.447258544
58206	9	17	34.16	69.17	1.017838481	Up	0.0839584	0.446686603
17133	9	17	32.19	65.17	1.017595418	Up	0.0839584	0.446972391

68385	10	18	53.97	109.19	1.016611143	Up	0.0930716	0.465973997
235132	23.08	43.47	17.4	35.14	1.014026885	Up	0.00606746	0.104745508
67563	17	32	44.74	90.29	1.013000956	Up	0.0172809	0.197791429
66863	8	15	17.26	34.7	1.007503198	Up	0.1083126	0.517673259
74840	8	15	24.44	49.13	1.007359954	Up	0.1083126	0.517970773
227717	8	15	18.36	36.9	1.007054758	Up	0.1083126	0.518268628
50505	12.55	23.51	12.47	25.06	1.006924949	Up	0.0400528	0.312059315
18021	63	118	69.64	139.86	1.005995302	Up	4.74E-06	0.000310832
56077	2.36	4.42	3.03	6.08	1.00475353	Up	0.397842	0.787450828
114601	6.1	11.41	11.53	23.13	1.004372753	Up	0.1835948	0.618498919
67338	59	110	115.41	230.69	0.999187232	Up	1.12E-05	0.000664447
30843	7	13	22.44	44.85	0.999035309	Up	0.1405138	0.585485894
170772	23	41	26.56	53.08	0.998913224	Up	0.01155348	0.157342892
328274	15.86	29.51	31.91	63.66	0.996378548	Up	0.01957846	0.217216488
66254	7	13	18.5	36.83	0.993356126	Up	0.1405138	0.585192858
791260	7	13	35.22	70.11	0.993225326	Up	0.1405138	0.584607665
20316	14	26	74.4	148.09	0.993099697	Up	0.0350722	0.299012066
20104	7	13	34.19	68.05	0.993020739	Up	0.1405138	0.584900115
71514	7	13	13.4	26.67	0.992984824	Up	0.1405138	0.584315507
18824	21	39	121.97	242.75	0.992944953	Up	0.00957346	0.142251358
12763	231.8	429.62	166.48	330.86	0.990872014	Up	5.04E-18	1.68E-15
17318	47	87.05	99.8	198.17	0.989626856	Up	0.000108609	0.004496196
15042	77.92	144.1	240.74	477.26	0.987299555	Up	4.47E-07	3.79E-05
117149	15.45	28.49	22.07	43.63	0.983233844	Up	0.0276514	0.262058428
71999	8.46	15.59	26.96	53.24	0.981690075	Up	0.1083126	0.518566827
13063	6.34	11.67	13.91	27.44	0.980158062	Up	0.1835948	0.617998516

227525	105.92	195.36	209.04	412.2	0.979565475	Up	5.59E-09	6.46E-07
223920	23.57	43.35	74.79	147.43	0.979112834	Up	0.00606746	0.104528643
19691	45	82	90.02	177.45	0.979095105	Up	0.000228476	0.008563733
14113	115	211	679.24	1335.55	0.975440662	Up	2.30E-09	2.99E-07
67425	6	11	10.64	20.92	0.975384701	Up	0.1835948	0.619000134
100534273	12	22	12.66	24.89	0.975288826	Up	0.0568042	0.355656695
320116	6	11	17.86	35.11	0.975149916	Up	0.1835948	0.618749425
76580	6	11	10.49	20.62	0.975029655	Up	0.1835948	0.618248616
67123	6	11	15.93	31.31	0.974877242	Up	0.1835948	0.619753481
12607	6	11	9.81	19.28	0.97478001	Up	0.1835948	0.620256732
227333	12	22	14.06	27.63	0.974638968	Up	0.0568042	0.355389284
14841	6	11	14.3	28.1	0.974554983	Up	0.1835948	0.620005004
17121	6	11	31.51	61.91	0.974362705	Up	0.1835948	0.619251046
65019	6	11	38.72	76.06	0.974058986	Up	0.1835948	0.620508664
59032	56	102	157.39	307.33	0.965444722	Up	3.94E-05	0.00195279
75210	9	9	31.31	61.06	0.963604081	Up	0.87736	0.9579468
213550	5	9	9.4	18.28	0.959533408	Up	0.242172	0.66725603
22143	17.12	31.06	66.35	129.01	0.959314527	Up	0.0241466	0.248360765
269615	1	2	1.32	2.56	0.955605881	Up	0.573542	0.722659446
68524	22.04	39.84	19.34	37.49	0.954918031	Up	0.01450208	0.180107176
75695	10	18	29.53	56.99	0.948527436	Up	0.0930716	0.465413932
234069	10	18	27.95	53.94	0.948507239	Up	0.0930716	0.466254536
13121	5	9	7.68	14.82	0.948367232	Up	0.242172	0.667919527
408022	5	9	8.78	16.94	0.94814103	Up	0.242172	0.668140985
56700	5	9	21.8	42.06	0.948120715	Up	0.242172	0.667477049
27045	5	9	25.39	48.98	0.947932379	Up	0.242172	0.667698215

17762	5	10	7.95	15.33	0.947330931	Up	0.1632572	0.56461478
24071	77	138.01	49.16	94.48	0.942524049	Up	2.75E-06	0.000192598
15461	3	5	9.23	17.71	0.940161659	Up	0.4415	0.820027121
53618	130.81	234	419.45	804.09	0.938858142	Up	8.04E-10	1.15E-07
93734	60.84	108.63	133.72	256	0.93692855	Up	3.07E-05	0.001587047
19231	14	25	80.33	153.72	0.9362941	Up	0.049088	0.362754217
20817	73	130	75.6	144.36	0.933212909	Up	6.40E-06	0.000397663
23994	9	16	32.72	62.35	0.930216812	Up	0.1198192	0.51551994
654801	31	55	97.41	185.27	0.9274875	Up	0.00358786	0.073897483
15258	13	23	21.21	40.23	0.923527117	Up	0.0624368	0.387136075
218214	133	235	178.06	337.34	0.921839935	Up	1.84E-09	2.43E-07
170942	55	97	480.09	907.05	0.917877194	Up	0.000120066	0.004897414
67315	6	11	23.6	44.58	0.917609757	Up	0.1835948	0.619502162
68428	2	4	4.85	9.15	0.915786996	Up	0.397842	0.784093624
20185	99.32	163.3	163.62	308.35	0.91421974	Up	5.90E-06	0.000372229
434232	61	107	203.25	382.18	0.91099693	Up	6.03E-05	0.002820267
233335	20	34	16.93	31.82	0.910351862	Up	0.0312172	0.271713725
231672	4	7	5.36	10.07	0.909758778	Up	0.323714	0.687850918
240174	4	7	3.37	6.33	0.909456908	Up	0.323714	0.69049582
19267	4	7	5.34	10.03	0.909409959	Up	0.323714	0.689788526
76899	4	7	5.57	10.46	0.909133619	Up	0.323714	0.689965214
77371	4	7	3.92	7.36	0.908852112	Up	0.323714	0.69067287
68944	163.72	286.63	244.52	459.02	0.908604543	Up	4.97E-11	8.27E-09
81000	4	7	2.85	5.35	0.908576972	Up	0.323714	0.688906443
15441	4	7	5.44	10.21	0.90830431	Up	0.323714	0.688026614
56307	4	7	5.35	10.04	0.908148473	Up	0.323714	0.6882024

225870	4	7	6.4	12.01	0.908092341	Up	0.323714	0.689082679
11852	4	7	11.9	22.33	0.908021678	Up	0.323714	0.687324367
76816	8	14	19.39	36.38	0.90783274	Up	0.1550182	0.539935723
237761	4	7	7.35	13.79	0.907806302	Up	0.323714	0.690141992
232187	4	7	10.73	20.13	0.907697096	Up	0.323714	0.688378276
15426	4	7	11.37	21.33	0.907651711	Up	0.323714	0.687499794
66552	4.49	7.85	5.32	9.98	0.90761357	Up	0.323714	0.689611929
56706	4	7	12.48	23.41	0.907507	Up	0.323714	0.687675311
103784	4	7	7.81	14.65	0.907506211	Up	0.323714	0.690318861
12912	4	7	3.22	6.04	0.907487861	Up	0.323714	0.686973781
433693	4	7	15.67	29.39	0.907320179	Up	0.323714	0.687149029
433813	4	7	22.24	41.71	0.907236524	Up	0.323714	0.686798622
231600	4	7	9.06	16.99	0.907102897	Up	0.323714	0.688730298
235048	4	7	7.45	13.97	0.90701969	Up	0.323714	0.689435422
22295	4	7	2.4	4.5	0.906890596	Up	0.323714	0.689259006
53357	1	2	2.16	4.03	0.899748526	Up	0.573542	0.720042695
60594	374	650	888.05	1654.69	0.897848147	Up	1.29E-22	5.95E-20
216867	19	33	72.53	135.04	0.896737055	Up	0.028843	0.261725848
11569	10	18	23.01	42.73	0.892988333	Up	0.0930716	0.465693797
252903	60.64	105.03	140.77	261.37	0.89275365	Up	7.46E-05	0.003335513
242570	11	19	18.37	34.01	0.88860738	Up	0.1018244	0.505236036
216874	7	12	10.3	18.94	0.878791993	Up	0.2018	0.645837615
22666	7	12	13.01	23.92	0.878596427	Up	0.2018	0.64608611
18108	7	12	10.71	19.69	0.878504631	Up	0.2018	0.646583673
320150	7	12	10.25	18.84	0.878175055	Up	0.2018	0.645093277
242050	7	12	4.62	8.49	0.877871702	Up	0.2018	0.646334796

66757	7	12	32.76	60.2	0.87782813	Up	0.2018	0.645589312
52662	7	12	18.89	34.71	0.877728663	Up	0.2018	0.645341199
17535	2.96	5.06	3.47	6.35	0.871820929	Up	0.245598	0.675800581
231866	20.9	35.56	26.93	49.14	0.867683628	Up	0.0229116	0.236535265
98238	10	17	23.9	43.56	0.865993336	Up	0.1306796	0.55507144
214084	33	56	58.07	105.66	0.863564376	Up	0.00566758	0.101201573
15354	13	22	56.24	102.01	0.859042086	Up	0.0864412	0.45523875
12323	3	5	4.97	9	0.85667915	Up	0.4415	0.825740953
12048	1	1	2.78	5.03	0.855473517	Up	0.947628	0.978435611
103266	8.44	14.23	14.95	27.03	0.854416027	Up	0.1550182	0.539709808
66164	35.17	59.27	81.54	147.34	0.853569286	Up	0.0049022	0.095084397
101739	45	85	114.06	206.04	0.853131507	Up	8.91E-05	0.003944198
20615	28	47	98.76	177.7	0.84744494	Up	0.01267372	0.161993893
320538	34	57	15.4	27.68	0.845913592	Up	0.00608858	0.104675773
54446	7	5	4.23	7.58	0.841540185	Up	0.67177	0.800830683
20586	3	5	3.14	5.62	0.839805571	Up	0.4415	0.824258806
106021	3	5	5.22	9.34	0.839372743	Up	0.4415	0.821126844
70790	3	5	2.04	3.65	0.839327312	Up	0.4415	0.820576614
22214	3	5	4.39	7.85	0.838471714	Up	0.4415	0.821861633
14105	3	5	5.85	10.46	0.838374322	Up	0.4415	0.819112932
67731	7.12	11.88	6.84	12.23	0.838356174	Up	0.284358	0.620383565
18011	6	10	10.88	19.45	0.838091599	Up	0.264802	0.694646104
229615	3	5	7.11	12.71	0.838042565	Up	0.4415	0.824999214
69274	3	5	4.33	7.74	0.837966541	Up	0.4415	0.822781971
20220	9	15	17.36	31.03	0.837896749	Up	0.1684114	0.578831582
59126	3	5	6.35	11.35	0.837863801	Up	0.4415	0.822413589

23857	6	10	11.33	20.25	0.837774047	Up	0.264802	0.69442718
170711	6	10	11.61	20.75	0.837743364	Up	0.264802	0.695084367
545611	2.7	4.5	4.37	7.81	0.837689269	Up	0.397842	0.788013159
78832	9	15	10.76	19.23	0.837680685	Up	0.1684114	0.579070768
21463	3	5	11.51	20.57	0.83765396	Up	0.4415	0.824813987
14778	3	5	13.43	24	0.837575101	Up	0.4415	0.821310418
380629	9	15	16.43	29.36	0.837519488	Up	0.1684114	0.579310153
67815	3	5	7.98	14.26	0.83751333	Up	0.4415	0.823704372
11907	6	10	7.98	14.26	0.83751333	Up	0.264802	0.694208394
243914	138	230	268.43	479.66	0.837466217	Up	4.20E-08	4.11E-06
14389	3	5	3.33	5.95	0.837367491	Up	0.4415	0.819478363
243771	3	5	6.05	10.81	0.837359476	Up	0.4415	0.820210203
59058	12	20	25.09	44.83	0.837352029	Up	0.1102082	0.482400017
101544	12.03	20.05	29.48	52.67	0.837244935	Up	0.1102082	0.482653912
631990	15	25	43.68	78.04	0.837240922	Up	0.073382	0.444728057
14797	3	5	13.73	24.53	0.837215608	Up	0.4415	0.8196612
56289	6	10	23.47	41.93	0.837164988	Up	0.264802	0.693771235
30953	3	5	9.83	17.56	0.837029523	Up	0.4415	0.81984412
17702	3	5	9.36	16.72	0.836994412	Up	0.4415	0.822966286
242602	3	5	10.29	18.38	0.836893784	Up	0.4415	0.819295607
20787	3	5	4.63	8.27	0.836875136	Up	0.4415	0.820943352
66589	3	5	11.13	19.88	0.836864164	Up	0.4415	0.823519727
238021	3	5	11.83	21.13	0.836842694	Up	0.4415	0.825184524
13001	3	5	19.45	34.74	0.836827599	Up	0.4415	0.821677813
69878	3	5	23.89	42.67	0.836815257	Up	0.4415	0.822597738
75668	3	5	18.56	33.15	0.83681216	Up	0.4415	0.825555393

238023	3	5	9.35	16.7	0.836809833	Up	0.4415	0.823889101
18951	3	5	9.39	16.77	0.836685626	Up	0.4415	0.823150683
114641	6	10	39.37	70.31	0.836633182	Up	0.264802	0.693989746
215351	3	5	4.06	7.25	0.836501268	Up	0.4415	0.821494074
226539	3	5	5.55	9.91	0.836397286	Up	0.4415	0.823335164
15117	3	5	4.71	8.41	0.836378741	Up	0.4415	0.822229521
74287	3	5	6.19	11.05	0.836035055	Up	0.4415	0.825369917
11770	3	5	32.61	58.21	0.835952576	Up	0.4415	0.824073912
80985	3	5	3.57	6.37	0.835369298	Up	0.4415	0.818930339
17345	3	5	1.98	3.53	0.834167753	Up	0.4415	0.820393368
107986	64	106	224.18	398.01	0.828147104	Up	0.000231148	0.008625034
329831	4	7	21.48	37.92	0.819964971	Up	0.323714	0.686623552
67464	53	87	115.27	202.87	0.81553845	Up	0.001003178	0.02848957
15366	67.5	110.69	115.38	202.86	0.814091254	Up	0.000213656	0.008155191
13390	22	36	52.88	92.77	0.810936165	Up	0.0359818	0.305514855
237775	11	18	21.6	37.89	0.810785827	Up	0.140914	0.58568701
237859	2.04	3.31	4.36	7.6	0.801671284	Up	0.623204	0.758584038
20437	8	13	25.44	44.32	0.800859211	Up	0.218144	0.681884382
243813	8	13	32.07	55.87	0.800849467	Up	0.218144	0.682397077
77031	8	13	11.99	20.88	0.800290053	Up	0.218144	0.682140633
271144	21	34	42.46	73.7	0.795560248	Up	0.0450598	0.337482084
240514	31	52	48.81	84.67	0.794674137	Up	0.00877038	0.134895253
78651	26	42	47.01	81.43	0.792592722	Up	0.0262334	0.250043667
116848	13	21	10.34	17.91	0.792529152	Up	0.1182236	0.511564522
16348	127	205	150.69	260.82	0.791470821	Up	8.27E-07	6.50E-05
267019	44.75	72	57.91	99.91	0.786816588	Up	0.00291556	0.061731233

241915	5	8	3.04	5.22	0.779978483	Up	0.351202	0.709653191
18208	5	8	5.79	9.94	0.779682504	Up	0.351202	0.709480904
12822	5	8	5.91	10.14	0.778827617	Up	0.351202	0.708277228
327978	87.34	139.74	97.24	166.82	0.778670465	Up	6.59E-05	0.003065308
67384	5	8	6.96	11.94	0.778643625	Up	0.351202	0.708620718
269019	5	8	8.89	15.25	0.778553919	Up	0.351202	0.708448931
240880	5	8	7.62	13.07	0.778396238	Up	0.351202	0.7093087
26921	10	16	12.04	20.65	0.77830639	Up	0.1807826	0.615504098
668940	5	8	5.44	9.33	0.77827043	Up	0.351202	0.708792588
18542	5	8	22.06	37.83	0.778097985	Up	0.351202	0.708964542
68098	2.04	3.06	6.79	11.62	0.775126589	Up	0.623204	0.758140422
216725	13.18	21	12.09	20.65	0.772327537	Up	0.1182236	0.511830684
320407	11.95	19.04	20.44	34.9	0.77183184	Up	0.1018244	0.505537489
26878	17	27	24.81	42.25	0.768029606	Up	0.083819	0.446801985
244654	12	19	17.03	28.92	0.763989117	Up	0.1505516	0.52925216
19070	74	117.18	178.66	303.31	0.763576397	Up	0.000320506	0.011300553
237465	12	19	21.98	37.31	0.763370974	Up	0.1505516	0.529699731
217038	12	19	32.42	55.03	0.763334238	Up	0.1505516	0.529475851
226751	7	11	5.38	9.07	0.753496378	Up	0.284358	0.620058417
84095	7	11	12.42	20.93	0.752907138	Up	0.284358	0.620220948
71904	3	5	5.43	9.15	0.752819545	Up	0.4415	0.820759942
19348	42	66	81.01	136.48	0.752517639	Up	0.00756706	0.121555031
223255	14	22	36.56	61.59	0.752430058	Up	0.125877	0.538520574
70310	28	44	67.53	113.76	0.752392907	Up	0.029509	0.267477548
67136	7	11	20	33.69	0.752320428	Up	0.284358	0.620546268
12041	10.57	16.57	24.83	41.74	0.749346339	Up	0.1807826	0.615756044

66229	18.57	29.08	47.89	80.43	0.748009284	Up	0.0666022	0.409001407
50793	184	288	194.36	326.21	0.747069671	Up	2.72E-08	2.80E-06
93723	1.06	1.67	1.49	2.5	0.746615764	Up	0.947628	0.979286213
73690	16	25	101.56	170.05	0.743626706	Up	0.1055958	0.509074538
18738	25	39	44.07	73.72	0.742259174	Up	0.0430864	0.327417292
103737	9	14	22.18	36.99	0.737875935	Up	0.23286	0.644801351
246727	48.65	75.67	68.88	114.86	0.737719419	Up	0.00478288	0.093423344
51897	9	14	16.87	28.13	0.737649579	Up	0.23286	0.645015999
215160	59.82	93	95.89	159.85	0.737266469	Up	0.001422256	0.037215699
329679	3.57	5.54	3.35	5.58	0.736104026	Up	0.4415	0.824443784
26404	699	1084	871.54	1449.22	0.733637836	Up	2.36E-26	1.40E-23
218461	20	31	30.72	51.05	0.732732745	Up	0.0749048	0.406577196
80902	26.71	41.28	46.1	76.38	0.728428169	Up	0.0346512	0.298480989
17192	11	17	44.94	74.45	0.728271724	Up	0.1922198	0.618986438
19653	1	2.09	3.53	5.84	0.726300186	Up	0.573542	0.733432147
11688	47.74	73.63	97.78	161.68	0.725529916	Up	0.00586742	0.104322226
17760	10	16	20.18	33.34	0.72432793	Up	0.1807826	0.615252358
321007	26	40	31.31	51.65	0.722144841	Up	0.0454258	0.339611933
14456	13	20	33.69	55.57	0.721985812	Up	0.1596248	0.553663177
237940	5.5	8.45	14.5	23.86	0.718541143	Up	0.351202	0.709136579
239719	80.58	123.52	64.77	106.47	0.717049331	Up	0.000433548	0.014969099
16413	17	26	61.33	100.55	0.713248226	Up	0.1115342	0.487691055
71820	60.49	92.35	225.86	369.59	0.710496931	Up	0.00247196	0.057616748
54486	8.06	12.27	16.38	26.74	0.707064108	Up	0.301392	0.650215927
218294	51.28	77.77	61.14	99.42	0.701419551	Up	0.00710216	0.115876614
259104	521.33	790.12	677.42	1100.86	0.700508522	Up	2.92E-18	1.06E-15

16573	9.49	14.37	10.49	17.04	0.699910658	Up	0.23286	0.644586846
231887	45	68	137.68	223.04	0.695983464	Up	0.01131646	0.155643411
20822	2	3	1.52	2.45	0.688710426	Up	0.623204	0.760586753
234730	2	3	3.45	5.56	0.688488521	Up	0.623204	0.757144179
56381	4	6	2.16	3.48	0.688055994	Up	0.472874	0.771677692
100033459	2	3	4.49	7.23	0.687280202	Up	0.623204	0.758806041
449521	2	3	5	8.05	0.687060688	Up	0.623204	0.761480247
223917	2	3	6.38	10.27	0.686807853	Up	0.623204	0.763498304
75616	2	3	7.25	11.67	0.686751661	Up	0.623204	0.759361617
384783	2	3	3.33	5.36	0.686710823	Up	0.623204	0.763048923
12181	2	3	5.43	8.74	0.686681082	Up	0.623204	0.76338591
58186	2	3	5.25	8.45	0.686633919	Up	0.623204	0.765414094
65099	2	3	7.4	11.91	0.686576237	Up	0.623204	0.766092552
94093	2	3	1.51	2.43	0.686407764	Up	0.623204	0.765979392
54722	2	3	6.32	10.17	0.686323216	Up	0.623204	0.765075315
13626	2	3	6.55	10.54	0.686308055	Up	0.623204	0.763161219
230866	6	9	6.42	10.33	0.686195052	Up	0.373698	0.746230156
231003	2	3	4.45	7.16	0.686154251	Up	0.623204	0.763948215
68723	10	15	6.24	10.04	0.686141335	Up	0.246158	0.669591604
93871	2	3	1.56	2.51	0.686141335	Up	0.623204	0.75780805
20359	2	3	3.58	5.76	0.686109224	Up	0.623204	0.761592082
102334	2	3	5.88	9.46	0.686024029	Up	0.623204	0.765866266
11479	2	3	4.09	6.58	0.685986741	Up	0.623204	0.758251277
15412	2	3	5.24	8.43	0.685965819	Up	0.623204	0.761256677
432467	2	3	6.21	9.99	0.68589141	Up	0.623204	0.764849629
170625	2	3	2.99	4.81	0.68589141	Up	0.623204	0.764173369

230598	2	3	3.22	5.18	0.68589141	Up	0.623204	0.762487941
14936	6	9	10.91	17.55	0.685819929	Up	0.373698	0.746051118
56215	46	69	65.16	104.81	0.685717863	Up	0.01183164	0.158536355
58994	8	12	10.37	16.68	0.685703395	Up	0.301392	0.650722063
75734	2	3	8.02	12.9	0.685696924	Up	0.623204	0.757918808
76527	4	6	15.5	24.93	0.685614667	Up	0.472874	0.771526383
71804	12	18	35.03	56.34	0.685568576	Up	0.202806	0.647562827
57437	6	9	19.94	32.07	0.685558944	Up	0.373698	0.74730619
83395	4	6	7.94	12.77	0.685547613	Up	0.472874	0.770770726
218544	4	6	9.88	15.89	0.685536178	Up	0.472874	0.772738522
19822	2	3	4.34	6.98	0.685531994	Up	0.623204	0.759139289
19142	6	9	15.47	24.88	0.685513289	Up	0.373698	0.747126636
99382	2	3	2.91	4.68	0.685489377	Up	0.623204	0.761033238
75665	2	3	7.3	11.74	0.685464039	Up	0.623204	0.75736534
70333	14	21	41.96	67.48	0.685445296	Up	0.1681674	0.578710064
67803	88	132	200.2	321.96	0.685439486	Up	0.00048825	0.016316178
110962	4	6	6.61	10.63	0.68541942	Up	0.472874	0.771829061
259277	2	3	10.31	16.58	0.685399674	Up	0.623204	0.766432232
244144	6	9	10.08	16.21	0.685388452	Up	0.373698	0.746947167
17476	4	6	6.15	9.89	0.685384111	Up	0.472874	0.772131977
21927	6	9	10.87	17.48	0.685353244	Up	0.373698	0.746409279
72296	4	6	7.91	12.72	0.685349071	Up	0.472874	0.771072811
14065	2	3	7.68	12.35	0.685332826	Up	0.623204	0.766885608
317750	30	45	103.87	167.03	0.685328215	Up	0.0424456	0.32432492
11877	4	6	6.2	9.97	0.685325289	Up	0.472874	0.772283524
21762	12	18	26.84	43.16	0.685310193	Up	0.202806	0.648060187

30934	8	12	17.91	28.8	0.685303474	Up	0.301392	0.650384552
18519	4	6	5.74	9.23	0.685279911	Up	0.472874	0.770619772
67920	8	12	29.13	46.84	0.685235374	Up	0.301392	0.65004739
67210	2	3	5.28	8.49	0.685226624	Up	0.623204	0.760363707
406220	2	3	7.88	12.67	0.68514899	Up	0.623204	0.760029384
15932	8	12	12.47	20.05	0.685140772	Up	0.301392	0.650553264
14283	12	18	47.68	76.66	0.685089762	Up	0.202806	0.647314433
215751	2	3	8.72	14.02	0.685086309	Up	0.623204	0.761703949
211347	2	3	1.81	2.91	0.685029456	Up	0.623204	0.75681268
100040843	2	3	7.06	11.35	0.684952209	Up	0.623204	0.756481471
67031	2	3	9.71	15.61	0.684927337	Up	0.623204	0.757254744
78655	4	6	11.06	17.78	0.684903939	Up	0.472874	0.772890307
64011	2	3	10.55	16.96	0.684893171	Up	0.623204	0.758362165
227624	2	3	4.23	6.8	0.684877083	Up	0.623204	0.762039748
20405	2	3	6.88	11.06	0.684870916	Up	0.623204	0.762936661
72935	2	3	6.09	9.79	0.684866632	Up	0.623204	0.766545526
69694	4	6	19.95	32.07	0.684835607	Up	0.472874	0.770468877
68119	4	6	16.79	26.99	0.684822747	Up	0.472874	0.772586796
236732	4	6	7.21	11.59	0.684809402	Up	0.472874	0.771223942
69574	2	3	12.84	20.64	0.684797768	Up	0.623204	0.762600071
13611	2	3	5.63	9.05	0.68478287	Up	0.623204	0.764962455
22340	4	6	22.85	36.73	0.684764731	Up	0.472874	0.77243513
81703	2	3	8.71	14	0.684682203	Up	0.623204	0.75758663
19383	2	3	7.92	12.73	0.684660084	Up	0.623204	0.75947283
14664	2	3	4.1	6.59	0.684654556	Up	0.623204	0.761144941
236899	2	3	2.75	4.42	0.684614751	Up	0.623204	0.757475969

13447	4	6	5.27	8.47	0.684559008	Up	0.472874	0.771375133
104130	2	3	16.24	26.1	0.684498174	Up	0.623204	0.76080993
22185	2	3	6.16	9.9	0.684498174	Up	0.623204	0.761815849
231070	2	3	5.04	8.1	0.684498174	Up	0.623204	0.756702245
66514	2	3	5.93	9.53	0.684444109	Up	0.623204	0.756923148
108153	2	3	2.57	4.13	0.684373422	Up	0.623204	0.762151747
30853	2	3	8.88	14.27	0.684353753	Up	0.623204	0.762375843
213788	2	3	7.76	12.47	0.684332908	Up	0.623204	0.760921568
67733	2	3	4.91	7.89	0.684302276	Up	0.623204	0.765188208
13144	2	3	8.09	13	0.684300015	Up	0.623204	0.758473085
109077	2	3	4.07	6.54	0.684261841	Up	0.623204	0.765301134
66766	2	3	11.7	18.8	0.684224132	Up	0.623204	0.766658853
72175	2	3	4.4	7.07	0.684206691	Up	0.623204	0.761927782
94315	2	3	6.46	10.38	0.684200374	Up	0.623204	0.764736836
170770	2	3	6.84	10.99	0.684123156	Up	0.623204	0.762263778
14027	4	6	4.17	6.7	0.684113712	Up	0.472874	0.770921739
67427	2	3	3.89	6.25	0.684086035	Up	0.623204	0.756591842
225256	2	3	3.94	6.33	0.68400987	Up	0.623204	0.765753172
216873	2	3	12.12	19.47	0.683863185	Up	0.623204	0.766318972
53376	2	3	4.14	6.65	0.683723573	Up	0.623204	0.759806664
654362	2	3	27.1	43.53	0.683717169	Up	0.623204	0.760475214
330814	4	6	3.25	5.22	0.683610089	Up	0.472874	0.770318041
227737	2	3	3.63	5.83	0.683526335	Up	0.623204	0.758029599
67727	2	3	2.31	3.71	0.683526335	Up	0.623204	0.760140792
68198	2	3	31.61	50.76	0.683311034	Up	0.623204	0.765527087
94332	2	3	3.22	5.17	0.683103592	Up	0.623204	0.763273548

73192	2	3	2.23	3.58	0.682915877	Up	0.623204	0.759918008
320487	2	3	1.72	2.76	0.682259702	Up	0.623204	0.759028174
54214	2	3	1.77	2.84	0.682141569	Up	0.623204	0.762712235
24013	77.07	115.21	68.78	110.25	0.680717635	Up	0.001247518	0.034034745
105239	2	3	3.46	5.54	0.679113938	Up	0.623204	0.758917091
77559	12.16	18.11	8.4	13.42	0.675923439	Up	0.202806	0.647811411
24086	9	13	11.78	18.75	0.670551056	Up	0.316338	0.678065043
268741	29	43	43.47	69.11	0.668874384	Up	0.0523142	0.367657482
237542	11.32	16.77	10.6	16.83	0.666970912	Up	0.258214	0.679291399
211151	33.36	49.33	347.83	550.88	0.663355717	Up	0.037673	0.318898304
100504715	24.39	36.02	23.64	37.44	0.663350399	Up	0.0698966	0.427026144
26436	166.1	245.18	251.08	397.39	0.662408455	Up	4.00E-06	0.000264422
13731	7.12	10.5	13.86	21.92	0.661320541	Up	0.392414	0.780793136
70620	6.05	9.12	7	11.07	0.661228395	Up	0.373698	0.746588489
72392	40	59	90.59	143.26	0.661212138	Up	0.0242226	0.248834882
319670	129	190	84.65	133.69	0.659309583	Up	5.27E-05	0.002518069
26457	2.03	2.99	4.87	7.69	0.659061826	Up	0.93456	0.989877006
64177	17	25	38.73	61.06	0.656776088	Up	0.1467792	0.53427372
17175	49	72	107.38	169.17	0.655748439	Up	0.01340236	0.169227675
246787	49	72	146.14	230.21	0.655599391	Up	0.01340236	0.168971269
78286	2	3	1.2	1.89	0.655351829	Up	0.623204	0.757697324
20911	106.16	155.54	263.9	414.51	0.651415556	Up	0.000318306	0.011318907
245877	23	36	48.4	76.01	0.651182187	Up	0.0507682	0.363547498
70300	71	104	280.41	440.28	0.650883511	Up	0.00310924	0.065333298
226041	13	19	10.84	17	0.64916999	Up	0.212618	0.67603912
269642	13	19	13.27	20.8	0.648415158	Up	0.212618	0.675007393

12847	13	19	18.76	29.4	0.648156327	Up	0.212618	0.67526503
100503572	79	115	233.78	366.3	0.647874189	Up	0.00212836	0.051482801
17110	13	19	69.2	108.39	0.647387718	Up	0.212618	0.675780893
231872	13	19	81.81	128.14	0.64737179	Up	0.212618	0.675522863
26431	2.06	3	2.78	4.35	0.645930518	Up	0.623204	0.757033647
75678	2	3	4.58	7.16	0.644611989	Up	0.623204	0.758695023
353047	11	16	14.27	22.26	0.641468258	Up	0.258214	0.679936296
227580	11	16	23.32	36.37	0.641181137	Up	0.258214	0.680151533
58229	11	16	26.83	41.84	0.641035797	Up	0.258214	0.679506228
80719	11	16	34.78	54.23	0.640833238	Up	0.258214	0.679721194
626848	21.93	37.56	51.75	80.67	0.640473393	Up	0.01821938	0.205146767
54004	2.07	3.01	1.63	2.54	0.639956533	Up	0.623204	0.764060776
170930	31	45	213.77	332.5	0.637294938	Up	0.0567762	0.355749066
381379	20	29	65.04	101.09	0.636241128	Up	0.1280514	0.546698666
22417	100.48	145.53	175.88	273.13	0.634996348	Up	0.000618138	0.019706997
81910	263	374	633.12	982.25	0.633611292	Up	1.25E-07	1.14E-05
72962	7.49	10.82	24.75	38.34	0.631421812	Up	0.392414	0.780233427
329877	9	13	7.69	11.91	0.63111791	Up	0.316338	0.67876444
104215	27	39	43.41	67.23	0.631077727	Up	0.0793334	0.425892401
320100	9	13	21.45	33.22	0.631074426	Up	0.316338	0.678589456
231207	9	13	8.88	13.75	0.630800037	Up	0.316338	0.677890419
20637	9	13	34.8	53.88	0.630662545	Up	0.316338	0.677367086
74360	9	13	24.66	38.18	0.630644303	Up	0.316338	0.678239757
66396	1927.49	2782.9	2760.02	4272.74	0.630482809	Up	4.11E-50	6.84E-47
75841	18	26	27.68	42.85	0.630451261	Up	0.1531068	0.536421761
14791	9	13	56.86	88.01	0.630253358	Up	0.316338	0.677541441

14325	9	13	62.85	97.27	0.630082173	Up	0.316338	0.678414561
67824	2	3	9.73	15.05	0.629251777	Up	0.623204	0.760252233
67255	26.18	37.77	55.08	85.19	0.629155531	Up	0.0975922	0.485975282
21454	224	321	622	959.85	0.625894387	Up	6.47E-07	5.18E-05
619547	3	4.32	43.3	66.71	0.623536016	Up	0.65158	0.781914794
100040591	14	20	30.73	47.07	0.615160086	Up	0.221726	0.690745805
282619	7	10	20.41	31.26	0.615041596	Up	0.392414	0.780979884
19087	7	10	11.59	17.75	0.614938458	Up	0.392414	0.780606477
20595	14	20	73.99	113.28	0.614490967	Up	0.221726	0.691004512
66943	6	8	18.92	28.91	0.61165652	Up	0.514744	0.818808034
330450	107	152	211.04	321.43	0.606988117	Up	0.000780222	0.023104012
320713	19.45	27.58	17.46	26.55	0.604658302	Up	0.159139	0.552208348
22750	128.08	181.55	218.54	332.14	0.603894114	Up	0.000282036	0.01011561
396184	12	17	13.62	20.69	0.603206942	Up	0.269178	0.702581599
76014	29	41	51.87	78.63	0.600179485	Up	0.0852988	0.450648454
69786	65.04	91.94	205.65	311.64	0.599689348	Up	0.0118562	0.158610032
18293	146.99	207.61	147.74	223.76	0.598891676	Up	9.38E-05	0.004088077
259172	3.54	5	5.54	8.39	0.598784834	Up	0.4415	0.824628844
73389	119	168	271.23	409.63	0.594804618	Up	0.00049055	0.016262417
22171	684.61	963.53	1206.31	1820.34	0.593607238	Up	1.00E-16	2.77E-14
13106	6.09	8.58	23.45	35.38	0.593346125	Up	0.514744	0.818964593
319508	14.12	19.83	26.07	39.28	0.591404347	Up	0.288324	0.628048169
320806	16	19	24.6	36.97	0.587696728	Up	0.477632	0.777004081
66648	22	31	40.31	60.56	0.587227422	Up	0.1380398	0.578654497
20499	5	7	6.5	9.76	0.58644143	Up	0.496454	0.805261936
53970	5	7	7.98	11.98	0.586167257	Up	0.496454	0.805104996

72480	5	7	8.54	12.82	0.586088287	Up	0.496454	0.805418938
51885	45	63	70.36	105.62	0.586055659	Up	0.0364686	0.309017536
23972	5	7	9.3	13.96	0.58599632	Up	0.496454	0.805890311
14287	5	7	14.87	22.32	0.58593238	Up	0.496454	0.805733125
239827	5	7	15.43	23.16	0.585897191	Up	0.496454	0.80385167
73102	5	7	5.33	8	0.585864467	Up	0.496454	0.803695279
65945	5	7	7.47	11.21	0.58560613	Up	0.496454	0.804791298
214425	5	7	8.05	12.08	0.585559766	Up	0.496454	0.804164636
74090	5	7	9.57	14.36	0.585464919	Up	0.496454	0.80432121
67877	5	7	29.32	43.99	0.585290498	Up	0.496454	0.804008123
54201	93.55	130.87	90.42	135.64	0.585068867	Up	0.00270438	0.061484005
67039	5	7	7.88	11.82	0.584962501	Up	0.496454	0.804948117
18209	5	7	6.8	10.2	0.584962501	Up	0.496454	0.804477845
223881	6.88	9.6	21.05	31.49	0.581073524	Up	0.373698	0.746767785
76441	38	53	41.91	62.68	0.580710658	Up	0.0572542	0.357935536
22202	32.72	45.62	54.98	82.18	0.579880424	Up	0.0746198	0.405824416
68183	12.14	16.93	58.1	86.81	0.579323079	Up	0.351708	0.709125822
118446	201.79	280.99	384.5	574.04	0.578167671	Up	1.23E-05	0.000726607
83813	265	369	551.74	823.69	0.578112897	Up	5.30E-07	4.45E-05
12571	2.9	4.03	1.67	2.49	0.57629764	Up	0.397842	0.782795763
19116	8.49	11.8	5.47	8.15	0.575259226	Up	0.408192	0.790634458
78803	2.24	3.12	3.72	5.54	0.574583355	Up	0.623204	0.765640113
12352	6.2	8.6	33.92	50.47	0.573289917	Up	0.514744	0.818182392
83961	13	18	44.99	66.78	0.569811726	Up	0.279178	0.609721821
11352	9.92	13.73	6.34	9.41	0.569711883	Up	0.316338	0.677715885
72981	4.21	5.82	8.1	12.02	0.569443083	Up	0.669584	0.801093956

54422	4	6	7.9	11.72	0.569048011	Up	0.472874	0.771980489
17161	133.48	184.65	214.49	318.14	0.568751387	Up	0.000486878	0.016335935
338523	19.01	26.21	13.24	19.57	0.563740634	Up	0.204058	0.65106082
239170	6.49	8.95	10.72	15.84	0.56326743	Up	0.514744	0.818338713
320795	3	4	5.85	8.64	0.562594688	Up	0.65158	0.786908154
68272	34.63	47.63	55.12	81.28	0.560324514	Up	0.0794318	0.426145717
56705	8	11	17.51	25.82	0.560309917	Up	0.408192	0.791002709
15118	48	66	54.14	79.83	0.560236124	Up	0.0398178	0.310519132
27366	115.1	156.77	189.08	278.75	0.559975035	Up	0.00217018	0.051594479
73158	8	11	8.06	11.88	0.559683092	Up	0.408192	0.790818541
240239	8	11	33.22	48.96	0.559551485	Up	0.408192	0.791186963
319195	16	22	143.62	211.55	0.558742018	Up	0.238076	0.657058174
72278	76	103	176.02	258.55	0.554703943	Up	0.0129893	0.165013687
58991	1	2	18.54	27.19	0.552434907	Up	0.573542	0.717013669
320139	10.04	13.72	20.61	30.22	0.552159156	Up	0.433206	0.811505431
56632	49	67	94.29	138.23	0.551894079	Up	0.0409174	0.316719707
69188	11	15	10.09	14.76	0.548766547	Up	0.341252	0.690553962
214469	55	75	72.85	106.52	0.548123456	Up	0.0315736	0.274528658
14788	11	15	27.62	40.38	0.547927591	Up	0.341252	0.690386067
268930	11	15	36.41	53.23	0.547904825	Up	0.341252	0.690721939
66500	341	464	251.56	367.05	0.545074061	Up	9.75E-08	9.02E-06
12802	16.99	23.12	29.11	42.46	0.544589532	Up	0.1837952	0.617923175
69091	25	34	24.18	35.25	0.543809013	Up	0.1516254	0.532577017
66704	22	29.91	80.86	117.83	0.543208793	Up	0.221148	0.689461412
76510	175.62	238.62	416.26	606.36	0.542689655	Up	0.000137867	0.005515335
50873	197.86	268.87	913.77	1330.9	0.542499192	Up	5.16E-05	0.002495274

224671	14	19	14.01	20.39	0.54140482	Up	0.288324	0.628212622
230657	98.66	133.87	275.64	400.97	0.540709038	Up	0.00460552	0.090597002
353328	62	84	48.25	70.09	0.538679682	Up	0.0250498	0.254194373
432731	4.38	5.93	7.34	10.66	0.53835547	Up	0.669584	0.801209155
74105	9.51	12.87	12.98	18.85	0.53827414	Up	0.42164	0.791800144
59125	2.96	4	4.81	6.98	0.537190142	Up	0.397842	0.778744597
242418	128.86	174.35	118.78	172.33	0.536881937	Up	0.001134696	0.031683911
18782	8.96	12.13	26.67	38.69	0.536742904	Up	0.301392	0.65089095
15361	18	21	60.65	87.89	0.535191382	Up	0.486522	0.790693274
331026	23	31	87	125.7	0.530897344	Up	0.1806016	0.615643553
384281	23	31	87.6	126.56	0.53081873	Up	0.1806016	0.615391447
55950	4	5	28.63	41.35	0.530359659	Up	0.669584	0.801901045
70560	7.67	10.32	11.68	16.85	0.528708317	Up	0.392414	0.780419908
70238	123.89	166.13	185.29	266.4	0.52380906	Up	0.001786186	0.044234684
57748	3	4	2.27	3.25	0.517747421	Up	0.65158	0.784857727
11302	3	4	3.85	5.51	0.517193873	Up	0.65158	0.781802045
214547	3	4	3.48	4.98	0.517058436	Up	0.65158	0.781351373
71302	3	4	2.53	3.62	0.516852312	Up	0.65158	0.785198723
108958	3	4	5.92	8.47	0.516764794	Up	0.65158	0.7866798
433956	3	4	5.83	8.34	0.5165515	Up	0.65158	0.783044076
76892	9	12	13.56	19.39	0.515955625	Up	0.42164	0.79126442
13560	6	8	15.12	21.62	0.515908384	Up	0.514744	0.819591432
12835	3	4	2.21	3.16	0.515878189	Up	0.65158	0.786337517
67236	3	4	9.98	14.27	0.515873614	Up	0.65158	0.785995532
13654	3	4	6.77	9.68	0.515851214	Up	0.65158	0.784971359
332110	3	4	10.33	14.77	0.515829572	Up	0.65158	0.786565672

15464	3	4	8.26	11.81	0.515795278	Up	0.65158	0.783496702
246228	9	12	19.22	27.48	0.515773668	Up	0.42164	0.792336594
17975	3	4	2.42	3.46	0.51576499	Up	0.65158	0.784517028
15410	12	16	24.27	34.7	0.515761554	Up	0.351708	0.708782337
114774	3	4	11.1	15.87	0.515742452	Up	0.65158	0.783723212
75608	3	4	12.66	18.1	0.515712293	Up	0.65158	0.782704949
67516	12	16	26.7	38.17	0.515599445	Up	0.351708	0.708954038
56395	3	4	9.29	13.28	0.515504645	Up	0.65158	0.783270324
20975	3	4	5.03	7.19	0.515433371	Up	0.65158	0.784290059
19325	9	12	17.07	24.4	0.515418089	Up	0.42164	0.791442915
54607	6	8	12.53	17.91	0.515378923	Up	0.514744	0.819748292
70044	3	4	7.29	10.42	0.515364558	Up	0.65158	0.784630562
67238	6	8	18.26	26.1	0.515363041	Up	0.514744	0.819905211
56795	3	4	19.66	28.1	0.515306809	Up	0.65158	0.785653844
17524	3	4	7.99	11.42	0.515295242	Up	0.65158	0.782931001
241943	9	12	24.16	34.53	0.51522988	Up	0.42164	0.792157697
70510	6	8	22.04	31.5	0.515227605	Up	0.514744	0.819434632
70078	9	12	33.62	48.05	0.515216706	Up	0.42164	0.79197888
73047	3	4	15.75	22.51	0.515214228	Up	0.65158	0.782253236
20535	3	4	5.01	7.16	0.515148984	Up	0.65158	0.784176624
69019	3	4	20.18	28.84	0.51514499	Up	0.65158	0.782817958
56085	3	4	5.57	7.96	0.515091103	Up	0.65158	0.781126233
14697	3	4	9.09	12.99	0.515049231	Up	0.65158	0.782140389
67789	15	20	58.48	83.57	0.515041825	Up	0.29671	0.641779025
230098	3	4	12.33	17.62	0.515041125	Up	0.65158	0.78679396
14609	3	4	6.48	9.26	0.51501838	Up	0.65158	0.783949853

12228	3	4	16.83	24.05	0.515001717	Up	0.65158	0.783383497
66805	3	4	10.28	14.69	0.514994131	Up	0.65158	0.785881603
12274	3	4	7.04	10.06	0.514982971	Up	0.65158	0.781576644
226265	6	8	15.62	22.32	0.514942574	Up	0.514744	0.818651534
11565	3	4	11.24	16.06	0.514829857	Up	0.65158	0.785312454
18053	3	4	5.9	8.43	0.514817677	Up	0.65158	0.786223489
78252	6	8	11.94	17.06	0.51481481	Up	0.514744	0.819277893
224903	3	4	6.32	9.03	0.514801429	Up	0.65158	0.781013711
12262	3	4	19.38	27.69	0.514796483	Up	0.65158	0.787022381
338364	3	4	6.6	9.43	0.514791746	Up	0.65158	0.783157183
69815	3	4	22.46	32.09	0.514765862	Up	0.65158	0.784403527
73124	6	8	8.98	12.83	0.51473382	Up	0.514744	0.818495093
14612	3	4	12.06	17.23	0.514692794	Up	0.65158	0.786109494
66165	3	4	16.26	23.23	0.514661897	Up	0.65158	0.784744128
18571	3	4	3.36	4.8	0.514573173	Up	0.65158	0.782591972
19416	3	4	12.53	17.9	0.514573173	Up	0.65158	0.782479027
15247	3	4	7.21	10.3	0.514573173	Up	0.65158	0.781689328
231413	3	4	8.33	11.9	0.514573173	Up	0.65158	0.781463993
66911	3	4	15.82	22.6	0.514573173	Up	0.65158	0.780901221
55951	3	4	22.85	32.64	0.514446892	Up	0.65158	0.783609941
52846	3	4	6.28	8.97	0.514343426	Up	0.65158	0.785540014
24047	6	8	58.18	83.1	0.514325181	Up	0.514744	0.819121213
50788	3	4	10.67	15.24	0.514302727	Up	0.65158	0.785426218
170822	3	4	4.81	6.87	0.514273205	Up	0.65158	0.783836516
75565	3	4	17.49	24.98	0.514243188	Up	0.65158	0.786451578
11798	3	4	3.09	4.41	0.513171818	Up	0.65158	0.787136641

20090	3	4	48.11	68.65	0.512922921	Up	0.65158	0.781238787
72267	25.31	33.67	43.67	62.28	0.512126413	Up	0.1899196	0.611816102
74766	348	457	1172.66	1664.86	0.505616084	Up	1.32E-06	9.84E-05
20744	24	31.7	9.75	13.81	0.502239196	Up	0.231074	0.640922251
276829	6.48	8.56	16.61	23.52	0.501835987	Up	0.514744	0.818026131
20021	81	107	347.9	492.59	0.501714661	Up	0.01760642	0.20068907
338371	4.24	5.6	5.54	7.84	0.500967678	Up	0.669584	0.799484641
12951	12	15.79	27.75	39.14	0.496155985	Up	0.451968	0.835367776
69740	519.58	682.68	2189.05	3082.91	0.493987866	Up	3.25E-09	4.09E-07
16451	16	21	20.15	28.36	0.493077694	Up	0.304416	0.654702904
52892	9.27	12.16	14.48	20.36	0.491675959	Up	0.42164	0.791621489
319430	23.67	30.98	46.24	64.89	0.488854768	Up	0.226244	0.628783007
19336	13	17	47.13	66.07	0.487349663	Up	0.361078	0.725556638
22123	13	17	40.45	56.7	0.487209033	Up	0.361078	0.725731893
66403	13	17	39.98	56.04	0.487178484	Up	0.361078	0.725907233
20522	17.33	22.61	38.65	54.06	0.484093098	Up	0.311514	0.668758512
76809	51.65	67.33	49.15	68.7	0.483118682	Up	0.0643968	0.398694771
67459	516.48	671.77	365.86	510.27	0.479969132	Up	1.15E-08	1.28E-06
195046	20	26	27.79	38.74	0.479258119	Up	0.264698	0.695030627
72169	20	26	47.46	66.14	0.478810945	Up	0.264698	0.695250018
70951	7	9	24.64	34.26	0.475522895	Up	0.529262	0.838056918
194126	27	35	64.48	89.61	0.474807995	Up	0.1984228	0.63723509
13039	2	2.6	3.83	5.32	0.474081854	Up	0.93456	0.98200199
13078	17	22	22.13	30.71	0.472705061	Up	0.311514	0.668931095
72776	30.66	39.62	48.25	66.85	0.470398618	Up	0.1710102	0.587036252
52502	24	31	56.56	78.33	0.469782838	Up	0.231074	0.641135963

627214	31	40	44.66	61.79	0.468390122	Up	0.1744572	0.596653663
75692	38	49	216.53	299.23	0.466687901	Up	0.133253	0.564561208
72795	52	67	102.56	141.68	0.46616795	Up	0.0795472	0.426489853
26447	7.75	9.98	14.48	20	0.465938398	Up	0.529262	0.838216426
269593	7	9	6.19	8.54	0.46429666	Up	0.529262	0.838375995
224674	4	5	7.97	10.99	0.463539757	Up	0.669584	0.800058654
20623	7	9	9.72	13.4	0.463204782	Up	0.529262	0.838855067
60595	49	63	84.96	117.12	0.463131787	Up	0.091102	0.45804214
76789	16.89	21.73	50.46	69.56	0.463117726	Up	0.304416	0.654872165
18624	7	9	25.5	35.15	0.463027442	Up	0.529262	0.839014879
101533	7	9	32.26	44.46	0.46276151	Up	0.529262	0.839334687
60364	7	9	20.18	27.81	0.46267757	Up	0.529262	0.837738083
263876	7	9	11.72	16.15	0.462561595	Up	0.529262	0.83789747
70059	130	169	276.83	381.44	0.462455845	Up	0.00424604	0.08513566
71843	2	3	7.97	10.96	0.459596169	Up	0.623204	0.76451135
16593	1	1	2.74	3.76	0.456556769	Up	0.947628	0.967392049
69576	317.13	405.4	1708.67	2340.89	0.45418335	Up	2.49E-05	0.001313012
67374	18	23	24.93	34.15	0.454002696	Up	0.318064	0.680712588
19261	1	1	1.74	2.38	0.451874267	Up	0.947628	0.974806847
17951	48.25	61.54	59.98	82.03	0.451670106	Up	0.1080582	0.517646679
233405	7.15	9.11	18.53	25.31	0.449844626	Up	0.529262	0.839174753
329165	33	42	38.02	51.88	0.448419948	Up	0.1809688	0.615634254
15525	11	14	15.78	21.53	0.448251114	Up	0.443228	0.821037442
26371	11	14	24	32.74	0.448019916	Up	0.443228	0.82122026
22067	10.15	12.9	7.94	10.83	0.44782233	Up	0.558534	0.715780289
94352	74.35	94.5	95.69	130.41	0.446614431	Up	0.0461504	0.34471946

19338	121.13	153.56	232.62	316.16	0.442679711	Up	0.01204746	0.160652107
14756	219	277	314	425.85	0.439580791	Up	0.000717284	0.021862711
16923	19	24	29.79	40.34	0.43738296	Up	0.32412	0.687309511
68107	230	290	695.69	940.35	0.434753247	Up	0.000607088	0.019732731
72772	2.42	3.05	5.12	6.91	0.4325419	Up	0.623204	0.763835688
74549	91.77	115.54	114.14	154.02	0.432313234	Up	0.0298386	0.269584137
382867	2061.01	2588.04	3190.44	4295.59	0.429100897	Up	4.62E-24	2.26E-21
217826	4	5	8.73	11.75	0.428607198	Up	0.669584	0.80028849
71817	12.76	15.99	78.32	105.16	0.425133377	Up	0.451968	0.835553372
216274	8	10	6.65	8.92	0.42368937	Up	0.540992	0.69641003
15166	4	5	8.39	11.25	0.423182286	Up	0.669584	0.799369937
433323	4	5	6.69	8.97	0.423101774	Up	0.669584	0.799140629
67655	8	10	14.47	19.4	0.422991731	Up	0.540992	0.696194623
233424	8	10	12.39	16.61	0.422875886	Up	0.540992	0.69673339
74737	8	10	9.19	12.32	0.422865489	Up	0.540992	0.69630231
209737	4	5	5.52	7.4	0.422857004	Up	0.669584	0.801439652
78751	12	15	16.3	21.85	0.422761315	Up	0.451968	0.834996831
319530	8	10	16.48	22.09	0.422677176	Up	0.540992	0.69705705
20515	4	5	13.37	17.92	0.422571172	Up	0.669584	0.800633491
77219	8	10	17.19	23.04	0.422571172	Up	0.540992	0.69662557
57170	8	10	25.22	33.8	0.422454971	Up	0.540992	0.69694913
56389	4	5	14.79	19.82	0.42233491	Up	0.669584	0.800748558
230376	28	35	50.4	67.54	0.422318446	Up	0.248036	0.674039045
12833	8	10	13.41	17.97	0.422281172	Up	0.540992	0.696517783
404634	28	35	99.07	132.75	0.422191704	Up	0.248036	0.673818987
21345	16	20	68.45	91.72	0.422185813	Up	0.384012	0.764990149

239114	4	5	22.18	29.72	0.42217475	Up	0.669584	0.800518457
23939	4	5	8.59	11.51	0.422157797	Up	0.669584	0.800863657
380664	4	5	5.62	7.53	0.422079734	Up	0.669584	0.801324387
11776	4	5	5.65	7.57	0.422042433	Up	0.669584	0.800173555
16661	12	15	38.11	51.06	0.42202393	Up	0.451968	0.835182262
21428	4	5	15.57	20.86	0.421970213	Up	0.669584	0.80097879
105785	8	10	38.5	51.58	0.421953327	Up	0.540992	0.69608697
19339	4	5	18.31	24.53	0.421915443	Up	0.669584	0.801554951
69202	36	45	213.39	285.85	0.421765721	Up	0.1898582	0.611855183
68032	4	5	28.26	37.85	0.421531835	Up	0.669584	0.799255267
18829	4	5	31.89	42.71	0.4214698	Up	0.669584	0.800403457
66340	4	5	41.84	56.02	0.421059132	Up	0.669584	0.799599378
14707	4	5	53.13	71.1	0.420322847	Up	0.669584	0.79982895
66359	4	5	64.3	86.03	0.4200211	Up	0.669584	0.801670283
20741	49	61	31.28	41.76	0.416881199	Up	0.1315708	0.558002358
67532	4.02	5.01	7.88	10.52	0.41686717	Up	0.669584	0.801785647
606496	41	51	121.36	161.83	0.415186088	Up	0.1692416	0.58144482
20658	7	9	5.7	7.6	0.415037499	Up	0.529262	0.837578757
53605	115.55	143.32	195.64	260.18	0.411308697	Up	0.0212536	0.220512725
28193	7.73	9.59	9.43	12.54	0.411207672	Up	0.529262	0.838695316
230801	46.57	58.77	173.2	230.27	0.410887536	Up	0.1260104	0.538814254
215494	25	31	71.05	94.44	0.410563392	Up	0.289578	0.627004564
67020	121	150	518.02	688.32	0.410071631	Up	0.01949552	0.216585076
12753	21	26	14.53	19.29	0.40881844	Up	0.334926	0.688128209
74485	215	266	360.62	478.37	0.407647506	Up	0.001989716	0.048552571
227715	22.92	28.25	95.04	125.56	0.401770193	Up	0.275594	0.712622024

74450	22	27	34.47	45.37	0.396397361	Up	0.339754	0.688359638
18521	22	27	54.26	71.39	0.395832954	Up	0.339754	0.688024588
625098	50.31	61.71	114.1	150.06	0.395240672	Up	0.1585812	0.550732122
69606	40.55	49.68	134.03	176.03	0.393265366	Up	0.200266	0.642410712
18769	7	8	45.75	60.05	0.392392503	Up	0.696088	0.80312649
12380	100.81	121.7	225.49	295.77	0.391412273	Up	0.0534976	0.3461536
99890	9	11	24.46	32.06	0.390350022	Up	0.550594	0.707020474
11766	9	11	23.17	30.36	0.389913747	Up	0.550594	0.706911383
74154	9	11	17.66	23.14	0.389903521	Up	0.550594	0.707238758
26456	9	11	13.96	18.29	0.389756134	Up	0.550594	0.706802326
19063	9	11	24.29	31.82	0.389571346	Up	0.550594	0.707129599
170737	5	5	7.51	9.83	0.388378509	Up	0.905638	0.975509877
103466	42.18	51.43	47.14	61.63	0.386681033	Up	0.204924	0.653573248
18969	3.34	4.06	9.38	12.23	0.382764576	Up	0.65158	0.782027575
13418	95	115	123.14	160.5	0.382273824	Up	0.0593982	0.369672717
70422	14	17	15.76	20.53	0.381466093	Up	0.466378	0.76332245
13713	14	17	27.32	35.57	0.380703491	Up	0.466378	0.763022284
14087	14	17	20.6	26.82	0.3806649	Up	0.466378	0.763472622
214585	28	34	24.33	31.67	0.380380547	Up	0.301242	0.650735899
66597	14	17	60.21	78.36	0.38011428	Up	0.466378	0.763172338
545276	18.51	22.45	51	66.33	0.379164279	Up	0.395962	0.786536119
68832	8.64	10.47	21.77	28.3	0.378460646	Up	0.540992	0.696841243
107769	19	23	74.55	96.74	0.375904185	Up	0.401154	0.783936692
380608	1.66	2	4.74	6.12	0.368644594	Up	0.573542	0.723645638
70350	64	77	233.3	300.88	0.367001904	Up	0.1315642	0.558258903
320352	231.37	278.3	742.19	956.99	0.366715287	Up	0.00408628	0.08213028

216152	133	161	285.53	368	0.366063432	Up	0.0256598	0.246268969
79202	263	316	421.4	542.88	0.365443021	Up	0.0023393	0.054986766
17261	5	6	6.15	7.92	0.36491402	Up	0.681698	0.791241325
13607	5	6	6.79	8.74	0.364221705	Up	0.681698	0.791683051
224143	5	6	11.84	15.24	0.364193822	Up	0.681698	0.791793559
213211	5	6	14.68	18.89	0.363770734	Up	0.681698	0.791904099
21982	5	6	17.82	22.93	0.363739018	Up	0.681698	0.790910354
69440	5	6	7.08	9.11	0.363701694	Up	0.681698	0.790139164
193670	5	6	9.94	12.79	0.363698507	Up	0.681698	0.791020647
16886	5	6	8.58	11.04	0.363690619	Up	0.681698	0.79046949
382620	15	18	13.92	17.91	0.363606126	Up	0.472344	0.772175722
18576	5	6	6.28	8.08	0.363590734	Up	0.681698	0.79057966
19062	5	6	12.63	16.25	0.363585079	Up	0.681698	0.79035935
12525	5	6	10.82	13.92	0.363458712	Up	0.681698	0.792014669
213484	5	6	9.01	11.59	0.363281555	Up	0.681698	0.7899191
332937	5	6	15.93	20.49	0.363173717	Up	0.681698	0.790029117
108012	5	6	15.2	19.55	0.363097284	Up	0.681698	0.790249242
93739	5	6	34.55	44.43	0.362848431	Up	0.681698	0.79113097
105446	5	6	21.76	27.98	0.362717406	Up	0.681698	0.79068986
329065	5.01	6	10.93	14.04	0.361249535	Up	0.681698	0.791462126
20687	10	12	16.1	20.64	0.358382282	Up	0.558534	0.715890544
68874	72	86	314.33	402.43	0.356457887	Up	0.120782	0.518589795
70423	5.15	6.14	9.56	12.23	0.355341881	Up	0.681698	0.79135171
59020	68	81	191.6	244.68	0.35279862	Up	0.136226	0.571915513
68070	21	25	10.4	13.28	0.352671618	Up	0.410238	0.792568005
69499	69	82	121.35	154.63	0.349646137	Up	0.1373266	0.575954959

74772	18.64	22.15	29.3	37.33	0.349434844	Up	0.395962	0.786348401
58223	86	102	176.01	223.82	0.34668156	Up	0.1000072	0.497108669
329650	119	141	77.03	97.87	0.345446274	Up	0.0539856	0.348498198
100043468	65	77	134.65	171.02	0.344950822	Up	0.155056	0.539615632
66177	76	90	264.81	336.16	0.344190464	Up	0.1246642	0.534706602
216119	96.2	113.8	141.64	179.66	0.34304049	Up	0.0929462	0.465906825
51810	22	26	40.13	50.85	0.341566618	Up	0.414216	0.799881953
22352	11	13	40.57	51.39	0.34107435	Up	0.565152	0.724149953
70615	28	33	51.24	64.75	0.337609717	Up	0.362498	0.728234152
73130	590.17	694.95	1053.07	1329.53	0.336314992	Up	3.25E-05	0.001671062
76784	10.39	12.23	26.24	33.12	0.335934953	Up	0.558534	0.716000834
77733	8	9.41	13.21	16.67	0.335623638	Up	0.700324	0.807006786
73095	18.64	21.93	39.29	49.56	0.33501402	Up	0.486522	0.790538872
17116	17	20	40.88	51.56	0.334857067	Up	0.482334	0.784040089
19358	35	40	112.72	142.16	0.33477207	Up	0.379588	0.756720591
66626	210	247	360.44	454.56	0.334711613	Up	0.01323264	0.167338598
230726	3.38	3.98	19	23.96	0.33462849	Up	0.923686	0.981608072
20430	182	214	187.62	236.56	0.334392523	Up	0.0212808	0.220519971
56404	42.65	50.1	45.47	57.27	0.332864599	Up	0.24319	0.669839123
229681	23	27	27.87	35.09	0.332346945	Up	0.417864	0.806552156
16563	23	27	36.18	45.54	0.331941884	Up	0.417864	0.806739291
192231	35	41	68.95	86.6	0.328816473	Up	0.321752	0.68701524
11692	302	353	1151.56	1442.84	0.325321743	Up	0.00388988	0.079332577
382018	6	7	5	6.26	0.324234562	Up	0.690126	0.798461964
193385	34.55	40.08	41.92	52.46	0.323579091	Up	0.319292	0.68211264
381077	31.01	36.19	127.32	159.3	0.323287205	Up	0.370804	0.741875471

13518	66	77	49.98	62.53	0.323197616	Up	0.1813946	0.616830595
207785	6	7	9.72	12.16	0.32311501	Up	0.690126	0.798573
103836	6	7	21.49	26.88	0.322867655	Up	0.690126	0.798018128
24010	6	7	20.3	25.39	0.322780668	Up	0.690126	0.797796394
110417	30	35	85	106.31	0.322742563	Up	0.368218	0.739189862
14357	6	7	10.81	13.52	0.322728629	Up	0.690126	0.79812904
11536	12	14	47.99	60.02	0.322709506	Up	0.570698	0.73058124
16401	24	28	16.24	20.31	0.322638607	Up	0.421212	0.791711103
72713	6	7	18.32	22.91	0.322557955	Up	0.690126	0.797685574
280411	6	7	16.09	20.12	0.322465979	Up	0.690126	0.797907246
66138	30	35	133.08	166.41	0.32244836	Up	0.368218	0.739011572
97387	6	7	11.35	14.19	0.322182292	Up	0.690126	0.798350959
57785	6	7	50.51	63.14	0.321985219	Up	0.690126	0.797574784
19240	6	7	75.18	93.92	0.321083493	Up	0.690126	0.798239984
50758	95.76	111.45	37.41	46.69	0.319689623	Up	0.1062354	0.511861473
223752	9	10	13.96	17.42	0.319445682	Up	0.703302	0.808758422
13807	3	4	8.23	10.25	0.316659574	Up	0.65158	0.785767707
109889	31	36	62.71	78.08	0.316257533	Up	0.370804	0.741697136
235040	112	130	248.49	309.23	0.315492495	Up	0.0891462	0.451207743
209131	27.33	31.69	24.24	30.15	0.314768303	Up	0.42972	0.805337865
20719	2	2	8.2	10.18	0.312041747	Up	0.93456	0.97645326
17105	38	44	246.88	306.33	0.311276695	Up	0.328368	0.692610932
383619	2.59	3	6.86	8.51	0.310950556	Up	0.623204	0.766999036
109934	13	15	19.46	24.08	0.307323682	Up	0.575366	0.716709654
19122	4311.05	4975	7731.94	9566.78	0.30720298	Up	1.07E-24	5.58E-22
11496	13	15	9.45	11.69	0.306888695	Up	0.575366	0.716495134

71923	13	15	48.81	60.37	0.306655049	Up	0.575366	0.716602378
320615	59	68	43.03	53.18	0.305540937	Up	0.23354	0.646254187
109245	28	32	60.9	74.77	0.296017305	Up	0.432114	0.809642106
27223	280	320	196.04	240.25	0.293388379	Up	0.0128183	0.163090328
52838	7	8	16.81	20.6	0.293324613	Up	0.696088	0.803795205
106369	21	24	54.32	66.56	0.293171954	Up	0.496578	0.803739649
232314	7	8	12.87	15.77	0.293170607	Up	0.696088	0.803237865
54624	7	8	24.21	29.66	0.292915518	Up	0.696088	0.803572176
215243	7	8	22.04	27	0.292835183	Up	0.696088	0.803683675
67917	7	8	15.2	18.62	0.292781749	Up	0.696088	0.803460709
57340	7	8	12	14.7	0.292781749	Up	0.696088	0.803906766
30050	38	41	98.92	121.05	0.291268934	Up	0.51661	0.820678085
66489	7	8	111.44	136.33	0.290835905	Up	0.696088	0.802903833
99681	878	1001	1005.6	1229.33	0.289815688	Up	1.35E-05	0.000772112
22130	18.42	20.99	29.53	36.08	0.289017982	Up	0.589746	0.729164408
108802	2	2	6.27	7.64	0.285107195	Up	0.93456	0.981630114
59038	73.81	83.91	341.83	416.49	0.285002839	Up	0.216326	0.682612304
17168	37	42	90.88	110.6	0.283316646	Up	0.38318	0.76369839
13522	59.91	67.94	98.84	120.18	0.282029911	Up	0.269542	0.702870254
19041	15	17	15.93	19.36	0.281332686	Up	0.582622	0.721213577
16570	90	102	87.55	106.4	0.281319067	Up	0.1771306	0.605547955
78560	30	34	36.26	44.06	0.28109057	Up	0.436342	0.816828297
12326	153.3	173.7	82.63	100.4	0.281021696	Up	0.0821604	0.439368052
50709	45	51	400.16	485.83	0.279874617	Up	0.34017	0.688699409
74320	38	43	40.82	49.53	0.279026439	Up	0.384806	0.766388398
54630	229	259	639.54	775.44	0.277980561	Up	0.0331906	0.28768644

20347	5	5	8.35	10.1	0.27450719	Up	0.905638	0.975762501
27784	1.02	1.15	1.97	2.38	0.272765944	Up	0.947628	0.967036128
12289	40	45	30.59	36.91	0.270951631	Up	0.387746	0.772058977
380614	597.85	672.55	623.92	752.61	0.270541402	Up	0.00080731	0.023737196
11443	8	9	25.08	30.25	0.270397794	Up	0.700324	0.807454067
71116	8	9	22.18	26.75	0.270279526	Up	0.700324	0.806895043
80879	8	9	22.57	27.22	0.270260648	Up	0.700324	0.806783332
13627	8	9	30.24	36.47	0.27025206	Up	0.700324	0.806336793
67143	8	9	12.04	14.52	0.270206061	Up	0.700324	0.806560001
70726	8	9	34.72	41.87	0.27014997	Up	0.700324	0.80711856
69077	32	36	124.31	149.9	0.270058026	Up	0.439914	0.82314468
19663	8	9	22.11	26.66	0.269977755	Up	0.700324	0.807230365
14852	8	9	7.78	9.38	0.269817768	Up	0.700324	0.806671651
108121	8	9	60.93	73.45	0.26960975	Up	0.700324	0.806448381
226090	17	19	21.03	25.2	0.260974884	Up	0.587776	0.726836691
14958	17	19	49.7	59.54	0.26061337	Up	0.587776	0.726944723
18226	3	3.35	5.36	6.42	0.260340297	Up	0.923686	0.985636215
237911	279.85	311.93	268.93	321.43	0.257273885	Up	0.0303012	0.268515746
68859	360	401	713.65	852.29	0.256127708	Up	0.01427784	0.1781198
16924	19	22	52.66	62.84	0.254975659	Up	0.49026	0.796301671
75770	9	10	13.34	15.9	0.253268099	Up	0.703302	0.809429591
433091	9	10	13.41	15.98	0.252958171	Up	0.703302	0.809093867
67422	9	10	19.41	23.12	0.25234128	Up	0.703302	0.809317652
69847	9	10	14.51	17.28	0.252055698	Up	0.703302	0.809205744
12827	9	10	9.28	11.05	0.251849659	Up	0.703302	0.808982021
54217	9	10	153.18	182.16	0.249978253	Up	0.703302	0.808646669

70873	858	951	3090.3	3671.7	0.248701288	Up	0.000239782	0.008907259
228136	28	31	39.45	46.84	0.247715776	Up	0.510162	0.822207632
11419	66	73	116.23	137.84	0.246012117	Up	0.314654	0.675325234
93760	19	21	15.47	18.34	0.245520442	Up	0.591384	0.73097241
108159	57	63	135.88	161.01	0.244817171	Up	0.352218	0.709638251
81013	5.43	6	13.59	16.1	0.244515232	Up	0.681698	0.791572573
16158	113.52	125	284.64	336.01	0.239365754	Up	0.1879076	0.606038426
229584	10	11	8.74	10.31	0.238339148	Up	0.705336	0.809531153
219158	20	22	36.13	42.62	0.238333338	Up	0.59273	0.732309774
12453	20	22	47.46	55.96	0.237683862	Up	0.59273	0.732418522
93679	10	11	20.55	24.23	0.237656011	Up	0.705336	0.809754533
72821	1	1.1	1.46	1.72	0.236440196	Up	0.947628	0.98923756
384309	74.57	81.91	137.26	161.67	0.236140733	Up	0.318608	0.681701509
117198	7	8	14.41	16.97	0.235916229	Up	0.696088	0.803015146
20068	10	11	146.25	172.22	0.235816069	Up	0.705336	0.809642827
245386	38	44	141.32	166.2	0.233954728	Up	0.328368	0.692786544
270066	7	7.67	6.62	7.78	0.232938938	Up	0.890562	0.967789787
57914	1	1	4.65	5.46	0.231670235	Up	0.947628	0.979407848
68031	1139	1246	1937.91	2273.01	0.23010246	Up	9.71E-05	0.004166436
224273	97	106	98.79	115.76	0.228699909	Up	0.258642	0.680202302
68767	109.73	119.79	255.15	298.64	0.227061772	Up	0.234054	0.647461215
213006	1214.38	1324.93	3061.15	3581.88	0.226643265	Up	8.01E-05	0.003565793
100039672	152	166	1426.09	1668.31	0.226322358	Up	0.1590468	0.552118658
211948	11	12	20.74	24.26	0.226163656	Up	0.706648	0.808583334
238276	11	12	10.97	12.83	0.225957645	Up	0.706648	0.808917046
67889	11	12	29.93	35	0.225762643	Up	0.706648	0.808694541

234086	20.32	22.12	87.35	101.91	0.222416016	Up	0.59273	0.732201058
15382	1	1	3.42	3.99	0.222392421	Up	0.947628	0.984421047
66385	19.29	20.98	35.36	41.23	0.221576186	Up	0.703834	0.809146548
67072	105	123	220.68	256.97	0.219644056	Up	0.0856484	0.451921583
268932	12	13	12.92	15.01	0.216317907	Up	0.707396	0.808883072
102058	24	26	21.52	25	0.216250017	Up	0.59586	0.735849074
19024	1	1	1.74	2.02	0.215267987	Up	0.947628	0.988121878
97114	26.91	29.17	291.15	337.92	0.214919121	Up	0.507336	0.81812846
77951	194.58	209.81	622.97	720.07	0.208974472	Up	0.1473594	0.536151101
24127	3.47	3.74	2.25	2.6	0.208586622	Up	0.923686	0.982235298
210582	13	14	34.88	40.27	0.207305432	Up	0.707696	0.80889264
231889	15	14	120.77	139.33	0.206243802	Up	0.99132	0.992751681
67630	188.66	202.91	179.63	207.17	0.205786789	Up	0.1617332	0.560508937
107951	5.86	6.29	11.59	13.34	0.2028781	Up	0.681698	0.790800092
20700	4.19	5.07	16.46	18.94	0.202471995	Up	0.669584	0.799943785
170740	3	3.22	3.99	4.59	0.202105407	Up	0.923686	0.985762627
13690	9.34	10	8.13	9.34	0.200167198	Up	0.703302	0.808870206
231986	14	15	32.12	36.9	0.200148923	Up	0.707634	0.808932891
326618	14	15	45.37	52.11	0.199821595	Up	0.707634	0.809044039
214058	39	46	49.92	57.32	0.199420675	Up	0.280426	0.612286735
66296	280.5	300.2	612.89	703.24	0.198388967	Up	0.094474	0.471859636
16412	43	46	68.55	78.63	0.197923188	Up	0.516758	0.82059987
75007	44	47	104.66	119.86	0.195640115	Up	0.516624	0.820543673
224617	30.11	32.16	27.94	31.99	0.195288972	Up	0.59605	0.735974484
73699	649	694	1198.04	1371.59	0.195173214	Up	0.0121185	0.161340862
15040	82.8	88.44	428.71	490.76	0.195015596	Up	0.359552	0.723014063

52665	148	158	337.16	385.9	0.194793656	Up	0.23668	0.653856003
52686	102.2	109.08	262.31	300.16	0.194458925	Up	0.322198	0.687791062
20444	15	16	22.42	25.65	0.194172548	Up	0.70728	0.808861583
224727	3	3	5.28	6.04	0.19401062	Up	0.923686	0.984752237
170938	29.43	31.39	66.65	76.23	0.193754001	Up	0.596286	0.736156648
108911	15	16	26.49	30.29	0.193393734	Up	0.70728	0.808972767
101867	76	81	176.54	201.72	0.192359028	Up	0.402318	0.785842272
56455	8.7	9.25	28.83	32.85	0.188322534	Up	0.700324	0.807342201
101490	80	85	112.73	128.42	0.187998403	Up	0.401604	0.784631811
381157	16	17	12.65	14.41	0.187932951	Up	0.706684	0.808402195
17254	236.38	249.8	615.61	700.1	0.18554434	Up	0.173735	0.594428016
319160	2.82	3	50.1	56.97	0.185391803	Up	0.623204	0.766205745
280645	455	482	696.37	790.96	0.183750685	Up	0.0509428	0.364484126
15384	26	27	71.38	81.04	0.183114273	Up	0.693712	0.801496467
434484	791.69	838.04	2204.47	2501.67	0.18245965	Up	0.0100231	0.148138926
22648	13.31	14.08	22.74	25.8	0.182138811	Up	0.707696	0.808781543
70432	139	147	103.51	117.38	0.181416461	Up	0.287074	0.625816807
320472	18	19	19	21.51	0.179008106	Up	0.704932	0.8095141
23805	36	38	26.4	29.88	0.178642219	Up	0.593136	0.732593833
20878	18	19	64.13	72.57	0.178373863	Up	0.704932	0.809402397
76022	37	39	32.65	36.91	0.176928746	Up	0.592454	0.732186207
210004	74	78	173.35	195.9	0.176429561	Up	0.449578	0.831319675
664799	497.37	523.91	1036.51	1170.59	0.175501824	Up	0.053557	0.346268685
217124	19	20	29.23	32.99	0.174578949	Up	0.703834	0.808923027
18554	20	20	33.17	37.43	0.174316036	Up	0.821908	0.911879529
17127	19	20	24.92	28.12	0.174292526	Up	0.703834	0.808811313

66308	83.62	87.89	210.31	236.98	0.172247857	Up	0.445268	0.824632768
15975	306.32	321.73	290.49	327.16	0.171507869	Up	0.1403544	0.588060908
80860	4.84	5.09	15.1	17	0.170986197	Up	0.669584	0.799714147
19355	91.59	103.62	178.98	201.3	0.169548789	Up	0.1777526	0.607175445
23821	206	215	335.33	376.74	0.16798766	Up	0.247582	0.673025097
268390	314	328	569.32	637.63	0.163479727	Up	0.1503184	0.529102964
223970	21.53	22.47	38.54	43.14	0.162669605	Up	0.701306	0.807355731
68379	4	4	9.64	10.78	0.161252127	Up	0.914182	0.979892879
66871	16.51	17.22	160.58	179.37	0.159646398	Up	0.706684	0.808513346
399566	25	26	81.36	90.83	0.158849201	Up	0.695334	0.80325895
192196	132.09	137.51	174.18	194.45	0.158820257	Up	0.37947	0.756666636
74440	4	4	5.89	6.57	0.157625736	Up	0.914182	0.983948832
12606	27	28	68.85	76.54	0.152757247	Up	0.692052	0.799689584
654470	219	227	358.35	398.26	0.152341233	Up	0.263794	0.693094371
235315	87	91	170.75	189.54	0.150616764	Up	0.443096	0.821158534
12765	82.67	85.55	182.27	202.22	0.149848564	Up	0.493404	0.80125189
68149	29	30	69.23	76.78	0.149333212	Up	0.688642	0.797188381
70911	30	31	74.02	82	0.147708774	Up	0.686902	0.795395427
30946	158	162.99	236.95	262.08	0.145424601	Up	0.39549	0.785786125
241919	143.35	147.86	107.56	118.96	0.145334892	Up	0.405986	0.787094479
18508	32	33	61.5	68	0.144948336	Up	0.683368	0.791633736
18749	1	1	1.54	1.7	0.142604395	Up	0.947628	0.958921633
52705	93.05	95.77	124.5	137.39	0.142131258	Up	0.53105	0.840088793
17199	1	1.02	1.82	2	0.13606155	Up	0.947628	0.97264248
74330	161	165	254.52	279.68	0.135998063	Up	0.3934	0.782007023
56078	22.17	22.67	42.86	46.99	0.132721918	Up	0.813674	0.903587529

14137	318	325	572.66	627.51	0.131959615	Up	0.244892	0.674080824
20449	1	1.03	0.74	0.81	0.130396637	Up	0.947628	0.986391367
229521	409.03	417.14	596.01	651.72	0.128915732	Up	0.1993966	0.640115397
100273	51	52	110.02	120.27	0.128511015	Up	0.648992	0.784124064
14751	22.63	23.03	52.62	57.42	0.125942082	Up	0.69991	0.807088568
75725	0.98	1	0.88	0.96	0.125530882	Up	0.46568	0.838002439
66603	6.37	6.48	27.2	29.64	0.123938796	Up	0.897816	0.971107102
59026	1	1	0.45	0.49	0.122856748	Up	0.947628	0.964198164
18174	2	2.03	4.41	4.8	0.12225575	Up	0.93456	0.981506217
67629	735.84	747.2	3716.86	4045.07	0.12208032	Up	0.0974446	0.485530848
14297	278.3	282.46	1743.2	1895.9	0.121144769	Up	0.31886	0.681714815
20088	4	4	32.64	35.49	0.120771517	Up	0.914182	0.983440003
216154	428.84	434.5	894.02	971.14	0.119372184	Up	0.218338	0.681978415
75965	16.26	16.46	20.98	22.77	0.118119614	Up	0.839728	0.929296009
18491	1	1	0.6	0.65	0.115477217	Up	0.947628	0.989113471
224139	1	1	0.6	0.65	0.115477217	Up	0.947628	0.968699335
19270	1	1	0.72	0.78	0.115477217	Up	0.947628	0.967867017
233789	2	2	0.85	0.92	0.11417102	Up	0.93456	0.981878
20190	2	2	0.86	0.93	0.112894056	Up	0.93456	0.979404756
16873	2.98	3	7.32	7.9	0.110009005	Up	0.623204	0.763610732
50770	1	1	0.89	0.96	0.10922907	Up	0.947628	0.970129501
226641	1	1	0.89	0.96	0.10922907	Up	0.947628	0.968937403
214459	1	1	1.28	1.38	0.108524457	Up	0.947628	0.980747834
210106	1	1	1.67	1.8	0.108148804	Up	0.947628	0.961494036
22385	1	1	1.03	1.11	0.107915339	Up	0.947628	0.958222456
104625	1	1	1.16	1.25	0.10780329	Up	0.947628	0.961611291

78795	1	1	1.29	1.39	0.107713817	Up	0.947628	0.983684205
76459	1	1	1.81	1.95	0.107484427	Up	0.947628	0.979043033
74013	1	1	1.81	1.95	0.107484427	Up	0.947628	0.971204901
56229	1	1	1.56	1.68	0.106915204	Up	0.947628	0.967748231
74023	1	1	1.83	1.97	0.106351981	Up	0.947628	0.97312262
66196	1	1	1.7	1.83	0.106308902	Up	0.947628	0.985898048
665113	1	1	1.44	1.55	0.106199404	Up	0.947628	0.976980868
15574	1	1	1.31	1.41	0.106128351	Up	0.947628	0.967154739
320213	3	3.01	3.15	3.39	0.105933445	Up	0.923686	0.981357406
360013	1	1	1.05	1.13	0.105933445	Up	0.947628	0.987379488
629378	1	1	2.37	2.55	0.105610188	Up	0.947628	0.983071012
20818	1	1	2.24	2.41	0.105534414	Up	0.947628	0.984052488
67241	1	1	1.19	1.28	0.105182237	Up	0.947628	0.973723461
71889	1	1	1.72	1.85	0.105116706	Up	0.947628	0.988245719
228005	1	1	1.06	1.14	0.10496956	Up	0.947628	0.98688518
212996	1	1	1.59	1.71	0.10496956	Up	0.947628	0.977707698
30947	1	1	2.12	2.28	0.10496956	Up	0.947628	0.964906093
76041	3	3.01	8.09	8.7	0.104875698	Up	0.923686	0.98753581
79554	1	1	2.52	2.71	0.104869118	Up	0.947628	0.966798993
109113	1	1	1.86	2	0.104697379	Up	0.947628	0.975892647
66615	1	1	2.26	2.43	0.104633541	Up	0.947628	0.983438836
76142	1	1	2.66	2.86	0.104588901	Up	0.947628	0.963726789
21969	1	1	1.73	1.86	0.104530583	Up	0.947628	0.970726651
20312	1	1	2.13	2.29	0.104494168	Up	0.947628	0.976255118
14056	1	1	2.53	2.72	0.104469267	Up	0.947628	0.988493492
24000	1	1	1.2	1.29	0.10433666	Up	0.947628	0.971683621

20589	1	1	1.2	1.29	0.10433666	Up	0.947628	0.968818355
11556	1	1	2.4	2.58	0.10433666	Up	0.947628	0.966325072
98682	1	1	2	2.15	0.10433666	Up	0.947628	0.98936168
243376	1	1	2.94	3.16	0.104108403	Up	0.947628	0.981113922
71436	1	1	1.47	1.58	0.104108403	Up	0.947628	0.980869833
140579	2	2	2.94	3.16	0.104108403	Up	0.93456	0.983616716
107831	1	1	1.07	1.15	0.104023065	Up	0.947628	0.984666907
99887	1	1	1.07	1.15	0.104023065	Up	0.947628	0.984175311
18559	1	1	2.41	2.59	0.103918951	Up	0.947628	0.969294725
67952	1	1	1.34	1.44	0.103835811	Up	0.947628	0.962315424
237716	1	1	2.28	2.45	0.103747925	Up	0.947628	0.96431608
18484	1	1	1.61	1.73	0.10371135	Up	0.947628	0.969890847
19133	1	1	2.55	2.74	0.103678646	Up	0.947628	0.967629475
20924	1	1	1.88	2.02	0.103622631	Up	0.947628	0.987750543
56531	1	1	0.94	1.01	0.103622631	Up	0.947628	0.977223025
232855	1	1	2.42	2.6	0.103504576	Up	0.947628	0.975651149
66878	16.11	16.14	29.46	31.65	0.103448069	Up	0.839728	0.929048888
13728	1	1	1.48	1.59	0.10342959	Up	0.947628	0.987503142
76757	1	1	1.48	1.59	0.10342959	Up	0.947628	0.976134264
223473	1	1	1.75	1.88	0.10337774	Up	0.947628	0.971324537
26407	3	3	3.77	4.05	0.103357385	Up	0.923686	0.982863326
17364	1	1	2.29	2.46	0.103310717	Up	0.947628	0.983929697
71538	1	1	3.1	3.33	0.103253962	Up	0.947628	0.978314217
73296	2	2	2.7	2.9	0.103093493	Up	0.93456	0.987614143
12193	1	1	1.89	2.03	0.103093493	Up	0.947628	0.977344148
319448	1	1	1.08	1.16	0.103093493	Up	0.947628	0.964551999

21355	1	1	2.7	2.9	0.103093493	Up	0.947628	0.96337356
14208	1	1	3.11	3.34	0.102933522	Up	0.947628	0.972882491
16800	2	2	3.11	3.34	0.102933522	Up	0.93456	0.976821223
24044	1	1	2.84	3.05	0.102918313	Up	0.947628	0.971563897
72843	1	1	1.76	1.89	0.102810806	Up	0.947628	0.979772936
403187	1	1	2.98	3.2	0.102759574	Up	0.947628	0.986144646
20662	2	2	1.49	1.6	0.102759574	Up	0.93456	0.982250064
171531	2	2	2.98	3.2	0.102759574	Up	0.93456	0.97694394
433809	1	1	2.71	2.91	0.102726302	Up	0.947628	0.96965231
326622	2	2	2.58	2.77	0.102514911	Up	0.93456	0.980021898
18933	1	1	1.63	1.75	0.102482958	Up	0.947628	0.975047927
74213	1	1	1.63	1.75	0.102482958	Up	0.947628	0.973603233
209012	1	1	1.63	1.75	0.102482958	Up	0.947628	0.962080599
26400	1	2	3.94	4.23	0.102462034	Up	0.573542	0.715508693
66870	5	5	5.03	5.4	0.102401007	Up	0.905638	0.976900933
77864	1	1	1.36	1.46	0.102361718	Up	0.947628	0.969771564
71709	1	1	2.04	2.19	0.102361718	Up	0.947628	0.973483036
98417	2	2	4.08	4.38	0.102361718	Up	0.93456	0.978911601
12540	1	1	3.13	3.36	0.102298576	Up	0.947628	0.965615061
20823	1	1	3.27	3.51	0.102180395	Up	0.947628	0.975168512
320683	1	1	1.09	1.17	0.102180395	Up	0.947628	0.985158994
330474	1	1	1.09	1.17	0.102180395	Up	0.947628	0.977101932
83603	1	1	2.18	2.34	0.102180395	Up	0.947628	0.968580345
66511	2	2	5.86	6.29	0.102159352	Up	0.93456	0.986611743
20935	1	1	2.59	2.78	0.102132785	Up	0.947628	0.97965121
53622	1	1	2.59	2.78	0.102132785	Up	0.947628	0.968342452

215280	1	1	1.5	1.61	0.102098188	Up	0.947628	0.970607162
15357	1	1	1.5	1.61	0.102098188	Up	0.947628	0.965378622
24017	3	3	7.23	7.76	0.102061005	Up	0.923686	0.98513089
231225	1	1	1.91	2.05	0.102051271	Up	0.947628	0.976376001
78309	5	5	4.23	4.54	0.102034634	Up	0.905638	0.976141684
23797	3	3	4.23	4.54	0.102034634	Up	0.923686	0.982486413
84004	1	1	2.32	2.49	0.102020937	Up	0.947628	0.978192853
319655	2	2	6.69	7.18	0.101977633	Up	0.93456	0.978542061
15259	4	4	3.55	3.81	0.101971973	Up	0.914182	0.982297059
51886	2	2	2.05	2.2	0.101879614	Up	0.93456	0.98874428
17909	1	1	0.82	0.88	0.101879614	Up	0.947628	0.980260143
269529	2	2	2.87	3.08	0.101879614	Up	0.93456	0.982746589
217718	1	1	1.23	1.32	0.101879614	Up	0.947628	0.961259611
54604	3	3	1.64	1.76	0.101879614	Up	0.923686	0.981482723
21808	3	3	4.24	4.55	0.101802281	Up	0.923686	0.986648422
26406	2	2	4.24	4.55	0.101802281	Up	0.93456	0.984114624
381204	1	1	3.01	3.23	0.101770678	Up	0.947628	0.978921488
22323	2	2	6.02	6.46	0.101770678	Up	0.93456	0.984987177
66877	4	4	8.62	9.25	0.101765496	Up	0.914182	0.983312878
217169	3.99	4	5.61	6.02	0.101762716	Up	0.65158	0.782366115
108101	1	1	2.6	2.79	0.101753499	Up	0.947628	0.964434025
74838	4	4	4.38	4.7	0.101729887	Up	0.914182	0.983185785
77300	6	6	4.38	4.7	0.101729887	Up	0.897816	0.971485947
104248	2	2	1.78	1.91	0.101695397	Up	0.93456	0.98423918
26554	8	8	10.82	11.61	0.101667473	Up	0.883768	0.963300174
19179	1	1	4.52	4.85	0.101661975	Up	0.947628	0.98038202

93896	4	4	5.48	5.88	0.101640262	Up		0.914182	0.982931699
24063	1	1	2.74	2.94	0.101640262	Up		0.947628	0.984912889
207565	1	1	1.37	1.47	0.101640262	Up		0.947628	0.980991862
68911	2	2	4.11	4.41	0.101640262	Up		0.93456	0.979651519
71929	2	2	4.66	5	0.10159814	Up		0.93456	0.97928142
11841	3	3	7.54	8.09	0.101575179	Up		0.923686	0.984373874
67087	2	2	5.21	5.59	0.101564911	Up		0.93456	0.977435113
97031	3	3	7.13	7.65	0.101557671	Up		0.923686	0.987282107
18162	1	1	0.96	1.03	0.101538026	Up		0.947628	0.962197997
71405	2	2	5.76	6.18	0.101538026	Up		0.93456	0.977926781
18477	1	1	4.8	5.15	0.101538026	Up		0.947628	0.960323053
53417	1	1	0.96	1.03	0.101538026	Up		0.947628	0.957989623
56838	3	3	5.35	5.74	0.101511845	Up		0.923686	0.986395175
68724	1	1	3.98	4.27	0.101467639	Up		0.947628	0.978557035
67684	3	3	6.04	6.48	0.101445264	Up		0.923686	0.985257173
71147	2	2	3.57	3.83	0.101420318	Up		0.93456	0.980639818
16846	1	1	2.06	2.21	0.101402032	Up		0.947628	0.988617426
12660	1	1	2.61	2.8	0.10137702	Up		0.947628	0.984789882
235610	1	1	2.61	2.8	0.10137702	Up		0.947628	0.967273379
231668	1	1	1.65	1.77	0.101283336	Up		0.947628	0.977828942
14221	2	2	5.5	5.9	0.101283336	Up		0.93456	0.985361602
140740	3	3	3.3	3.54	0.101283336	Up		0.923686	0.983743915
26414	1	1	1.1	1.18	0.101283336	Up		0.947628	0.958455401
210126	12	12	5.09	5.46	0.101235295	Up		0.859936	0.943751973
71409	7	7	7.98	8.56	0.10122205	Up		0.890562	0.969182109
109314	1	1	5.78	6.2	0.101198723	Up		0.947628	0.961376809

12826	17	17	17.07	18.31	0.101168733	Up	0.835082	0.92464635
381148	6	6	8.26	8.86	0.101164917	Up	0.897816	0.970602434
69754	1	1	3.58	3.84	0.101146723	Up	0.947628	0.988989413
229323	1	1	3.03	3.25	0.101121924	Up	0.947628	0.972402588
257947	1	1	6.06	6.5	0.101121924	Up	0.947628	0.968461384
11423	3	3	9.09	9.75	0.101121924	Up	0.923686	0.980981647
56443	1	1	4.27	4.58	0.101111528	Up	0.947628	0.987132272
245269	6	6	9.78	10.49	0.101108308	Up	0.897816	0.972244526
78891	6	6	15.29	16.4	0.101107408	Up	0.897816	0.971612295
17444	4	4	4.96	5.32	0.101086125	Up	0.914182	0.981536571
100270744	5	5	9.51	10.2	0.101051906	Up	0.905638	0.976394636
28113	1	1	3.86	4.14	0.10102992	Up	0.947628	0.959972314
110446	5	5	9.93	10.65	0.100987808	Up	0.905638	0.97702759
68089	1	1	3.31	3.55	0.100987808	Up	0.947628	0.981236011
68770	1	1	1.38	1.48	0.100928909	Up	0.947628	0.974084322
12795	1	1	2.76	2.96	0.100928909	Up	0.947628	0.965969936
103844	1	1	6.35	6.81	0.100898206	Up	0.947628	0.976859835
71751	5	5	4.97	5.33	0.100889681	Up	0.905638	0.977788218
50927	3	3	9.94	10.66	0.100889681	Up	0.923686	0.986901798
67840	1	1	2.9	3.11	0.10086168	Up	0.947628	0.958106025
99334	16	16	17.96	19.26	0.100820353	Up	0.839728	0.928925377
67378	3	3	6.77	7.26	0.100813714	Up	0.923686	0.982360839
232798	29	29	38	40.75	0.100800641	Up	0.787532	0.882209716
99412	3	3	4.56	4.89	0.100800641	Up	0.923686	0.985889072
72265	2	2	4.56	4.89	0.100800641	Up	0.93456	0.986486586
50884	1	1	1.52	1.63	0.100800641	Up	0.947628	0.963138218

216848	5	5	4.56	4.89	0.100800641	Up	0.905638	0.975257383
11432	20	20	28.61	30.68	0.100778985	Up	0.821908	0.912122762
242291	7	7	10.09	10.82	0.100774325	Up	0.890562	0.968675347
269623	9	9	25.85	27.72	0.100762977	Up	0.87736	0.957695469
60411	4	4	16.45	17.64	0.100762977	Up	0.914182	0.980903729
192775	1	1	2.35	2.52	0.100762977	Up	0.947628	0.961963229
66442	1	1	5.53	5.93	0.100752624	Up	0.947628	0.98836959
21778	26.07	26.08	40.37	43.29	0.100750214	Up	0.798284	0.894013616
110809	7	7	8.71	9.34	0.100749831	Up	0.890562	0.968422164
243270	4	4	8.16	8.75	0.100713865	Up	0.914182	0.981409937
11656	5	5	17.43	18.69	0.100694	Up	0.905638	0.977407756
54325	1	1	4.15	4.45	0.100694	Up	0.947628	0.981969189
78321	2	2	5.81	6.23	0.100694	Up	0.93456	0.982870799
22110	2	2	4.98	5.34	0.100694	Up	0.93456	0.987363352
26428	12	12	19.92	21.36	0.100694	Up	0.859936	0.943876462
229574	5	5	4.29	4.6	0.100656213	Up	0.905638	0.976268143
219149	1	1	2.63	2.82	0.100632363	Up	0.947628	0.96290299
54354	13	13	26.3	28.2	0.100632363	Up	0.854608	0.939143313
71876	20	20	54.54	58.48	0.100628611	Up	0.821908	0.912244427
18222	32	32	66.31	71.1	0.100623105	Up	0.777346	0.871620545
26433	13	13	26.58	28.5	0.100620815	Up	0.854608	0.939019301
16653	15	15	21.46	23.01	0.100610907	Up	0.844522	0.928672864
16981	9	9	17.03	18.26	0.10060833	Up	0.87736	0.957569853
67665	2	2	3.6	3.86	0.100603941	Up	0.93456	0.989751019
107568	17	17	18	19.3	0.100603941	Up	0.835082	0.924769407
243538	4	4	12.6	13.51	0.100603941	Up	0.914182	0.982677745

217331	1	1	1.8	1.93	0.100603941	Up	0.947628	0.966088286
21745	9	9	7.34	7.87	0.100583573	Up	0.87736	0.95744427
223527	1	1	2.77	2.97	0.100576955	Up	0.947628	0.982458583
11416	1	1	2.77	2.97	0.100576955	Up	0.947628	0.959038262
171504	8	8	14.82	15.89	0.100573677	Up	0.883768	0.963552611
213990	2	2	6.51	6.98	0.100569493	Up	0.93456	0.986987404
72502	2	2	3.74	4.01	0.100563967	Up	0.93456	0.980887205
59025	12	12	19.67	21.09	0.100562138	Up	0.859936	0.944374747
116891	5	5	8.45	9.06	0.100559709	Up	0.905638	0.975131185
20887	3	3	14.13	15.15	0.100556328	Up	0.923686	0.986142059
15416	3	3	7.62	8.17	0.100545081	Up	0.923686	0.981984311
77090	4	4	18.29	19.61	0.10053446	Up	0.914182	0.983058726
67509	2	2	5.82	6.24	0.100526876	Up	0.93456	0.989625065
269344	3	3	11.64	12.48	0.100526876	Up	0.923686	0.986015549
20529	6	6	10.67	11.44	0.100526876	Up	0.897816	0.971359633
20743	11	11	8.87	9.51	0.100511237	Up	0.865484	0.949214757
22793	42	42	82.47	88.42	0.100503327	Up	0.746636	0.849549864
625249	1	1	7.07	7.58	0.100487633	Up	0.947628	0.975289127
69276	6	6	10.12	10.85	0.100485753	Up	0.897816	0.970980886
225160	52	52	90.26	96.77	0.100473083	Up	0.719684	0.821917453
18438	4	4	10.26	11	0.100472793	Up	0.914182	0.980271704
69562	27	27	26.07	27.95	0.1004577	Up	0.794632	0.890043461
21945	68	68	169.22	181.42	0.10043342	Up	0.68202	0.79018218
27204	44	44	33.29	35.69	0.100431031	Up	0.740984	0.843811121
109359	7	7	16.23	17.4	0.100424306	Up	0.890562	0.969562528
68943	8	8	22.75	24.39	0.100423213	Up	0.883768	0.963174005

68041	3	3	9.85	10.56	0.100414205	Up	0.923686	0.987916607
68487	69	69	177.44	190.23	0.100413512	Up	0.679844	0.789970943
244216	1	1	5.55	5.95	0.100401897	Up	0.947628	0.982091492
99152	1	1	2.22	2.38	0.100401897	Up	0.947628	0.978071519
70810	3	3	12.21	13.09	0.100401897	Up	0.923686	0.984499962
17769	4	4	4.44	4.76	0.100401897	Up	0.914182	0.982170229
14421	1	1	4.44	4.76	0.100401897	Up	0.947628	0.974565887
70804	2	2	4.44	4.76	0.100401897	Up	0.93456	0.981010945
18810	35	35	15.68	16.81	0.100394165	Up	0.767652	0.861563568
233899	102	102	268.67	288.03	0.100383847	Up	0.61646	0.759148092
12537	7	7	18.18	19.49	0.100381891	Up	0.890562	0.969435688
18087	6	6	5.69	6.1	0.10038059	Up	0.897816	0.970476349
109050	14	14	20.54	22.02	0.100378287	Up	0.849476	0.933873668
67872	1	1	4.58	4.91	0.100375426	Up	0.947628	0.970965717
18245	2	2	12.63	13.54	0.1003731	Up	0.93456	0.982126012
21803	6	6	18.46	19.79	0.100369059	Up	0.897816	0.970350297
109620	10	10	6.94	7.44	0.100366959	Up	0.87128	0.952683427
17874	1	1	3.47	3.72	0.100366959	Up	0.947628	0.965496827
74122	1	1	2.36	2.53	0.100350525	Up	0.947628	0.974686352
21415	1	1	2.36	2.53	0.100350525	Up	0.947628	0.969175588
16410	5	5	4.72	5.06	0.100350525	Up	0.905638	0.977915105
230810	14	14	28.32	30.36	0.100350525	Up	0.849476	0.933997066
16528	4	4	15.41	16.52	0.100346825	Up	0.914182	0.981283336
22420	4	4	13.05	13.99	0.100346156	Up	0.914182	0.983567161
67771	3	3	10.69	11.46	0.100345191	Up	0.923686	0.981106868
319719	6	6	8.33	8.93	0.10034368	Up	0.897816	0.971233351

107939	19	19	22.63	24.26	0.100342966	Up	0.82618	0.915764457
15191	4	4	11.94	12.8	0.100340974	Up	0.914182	0.981156768
68938	2	2	7.22	7.74	0.100334729	Up	0.93456	0.987739586
74168	2	2	7.36	7.89	0.100319534	Up	0.93456	0.983865607
72560	2	2	5	5.36	0.100304906	Up	0.93456	0.981754041
19049	1	1	5	5.36	0.100304906	Up	0.947628	0.959154919
57370	3	3	10.14	10.87	0.100294288	Up	0.923686	0.981232121
13419	2	2	10.42	11.17	0.100273908	Up	0.93456	0.977803817
19933	46	46	166.22	178.18	0.100241428	Up	0.735472	0.839257064
230579	25	25	85.89	92.07	0.100240975	Up	0.80201	0.897944727
59014	5	5	16.4	17.58	0.100239256	Up	0.905638	0.977661365
50760	2	2	8.2	8.79	0.100239256	Up	0.93456	0.98887001
73137	1	1	1.39	1.49	0.100227448	Up	0.947628	0.980503928
104394	5	5	16.68	17.88	0.100227448	Up	0.905638	0.976015257
68133	3	3	15.29	16.39	0.100227448	Up	0.923686	0.980230992
59002	4	4	16.96	18.18	0.10021603	Up	0.914182	0.982043432
53332	6	6	8.62	9.24	0.100204982	Up	0.897816	0.971865089
224454	2	2	4.31	4.62	0.100204982	Up	0.93456	0.977189465
74770	4	4	14.46	15.5	0.100200663	Up	0.914182	0.981789936
67224	7	7	21.83	23.4	0.100196399	Up	0.890562	0.967663411
21453	2	2	2.92	3.13	0.100194288	Up	0.93456	0.983741146
235184	1	1	2.92	3.13	0.100194288	Up	0.947628	0.96278542
68734	20	20	29.34	31.45	0.100191146	Up	0.821908	0.912001129
22032	10	10	22.25	23.85	0.10018393	Up	0.87128	0.952182937
18991	2	2	4.45	4.77	0.10018393	Up	0.93456	0.978295856
12317	3	3	10.43	11.18	0.10018103	Up	0.923686	0.983995802

77411	3	3	7.51	8.05	0.100175875	Up	0.923686	0.98500464
53311	5	5	18.22	19.53	0.10016899	Up	0.905638	0.977281001
23937	8	8	19.89	21.32	0.100164162	Up	0.883768	0.963805181
104943	5	5	12.52	13.42	0.100150109	Up	0.905638	0.975888863
12373	3	3	7.93	8.5	0.100141975	Up	0.923686	0.98361802
69582	2	2	3.2	3.43	0.100136671	Up	0.93456	0.980763496
108014	3	3	17.67	18.94	0.100134291	Up	0.923686	0.986775094
56075	4	4	16.56	17.75	0.100116352	Up	0.914182	0.983694352
320640	1	1	1.67	1.79	0.100111485	Up	0.947628	0.984543962
56441	1	1	3.34	3.58	0.100111485	Up	0.947628	0.976013441
14600	1	1	1.67	1.79	0.100111485	Up	0.947628	0.971923159
72454	4	4	8.63	9.25	0.100092806	Up	0.914182	0.980777259
213452	10	10	10.44	11.19	0.100088324	Up	0.87128	0.952808632
266781	2	2	6.96	7.46	0.100088324	Up	0.93456	0.982498264
79043	33	33	139.21	149.21	0.100081378	Up	0.774062	0.868172247
56791	8	8	38.84	41.63	0.100080358	Up	0.883768	0.963426376
209039	5	5	7.1	7.61	0.100077429	Up	0.905638	0.975636173
18763	19	19	8.91	9.55	0.100075301	Up	0.82618	0.915642485
19732	4	4	9.05	9.7	0.100066955	Up	0.914182	0.983821576
227746	7	7	25.48	27.31	0.100064037	Up	0.890562	0.969689401
20964	7	7	14.62	15.67	0.100061869	Up	0.890562	0.968042639
68636	7	7	33.84	36.27	0.100047177	Up	0.890562	0.968801987
22115	6	6	29.11	31.2	0.10003119	Up	0.897816	0.972497649
53600	7	7	41.23	44.19	0.10002547	Up	0.890562	0.967916197
69076	3	3	19.64	21.05	0.100025304	Up	0.923686	0.983492157
67689	12	12	42.35	45.39	0.100012519	Up	0.859936	0.944125538

320557	7	7	10.31	11.05	0.100002037	Up		0.890562	0.969055368
224824	1	1	2.09	2.24	0.09999579	Up		0.947628	0.982336189
99031	5	5	4.18	4.48	0.09999579	Up		0.905638	0.97677431
20598	1	1	4.18	4.48	0.09999579	Up		0.947628	0.976738832
228812	1	1	4.18	4.48	0.09999579	Up		0.947628	0.965024182
66653	9	9	44.31	47.49	0.099991429	Up		0.87736	0.958324043
67445	6	6	29.54	31.66	0.099991429	Up		0.897816	0.971991535
20670	4	4	23.69	25.39	0.099982196	Up		0.914182	0.980145396
76561	2	2	6.69	7.17	0.099966908	Up		0.93456	0.986111306
232371	1	1	2.23	2.39	0.099966908	Up		0.947628	0.966443509
15504	2	2	13.52	14.49	0.099962443	Up		0.93456	0.983243616
69922	6	6	23.28	24.95	0.099948757	Up		0.897816	0.970854703
54638	4	4	11.85	12.7	0.099941438	Up		0.914182	0.984076122
56752	2	2	4.74	5.08	0.099941438	Up		0.93456	0.983367951
75137	3	3	2.37	2.54	0.099941438	Up		0.923686	0.983240528
11677	17	17	82.54	88.46	0.099931804	Up		0.835082	0.924400336
68845	12	12	68.61	73.53	0.099914118	Up		0.859936	0.944000984
78903	4	4	10.18	10.91	0.09991354	Up		0.914182	0.980650821
28126	2	2	7.81	8.37	0.099905074	Up		0.93456	0.98299504
100678	3	3	13.11	14.05	0.099902445	Up		0.923686	0.987789642
16497	9	9	21.2	22.72	0.09989857	Up		0.87736	0.957821118
76281	7	7	33.62	36.03	0.099878927	Up		0.890562	0.968169114
227736	5	5	20.23	21.68	0.099868437	Up		0.905638	0.976647719
20229	4	4	23.44	25.12	0.099863894	Up		0.914182	0.980019122
16783	7	7	21.07	22.58	0.099855172	Up		0.890562	0.968928661
68549	5	5	6.28	6.73	0.099841946	Up		0.905638	0.975383614

69207	3	3	6.56	7.03	0.099828874	Up		0.923686	0.983114762
72147	1	1	3.35	3.59	0.099822748	Up		0.947628	0.966561975
212733	1	1	3.49	3.74	0.099811234	Up		0.947628	0.963962419
51788	6	6	38.53	41.29	0.099810233	Up		0.897816	0.970224277
23980	9	9	50.55	54.17	0.099782999	Up		0.87736	0.958198262
67443	3	3	11.87	12.72	0.099778736	Up		0.923686	0.984247817
72198	2	2	4.05	4.34	0.099773135	Up		0.93456	0.987865061
22154	5	5	12.71	13.62	0.099762673	Up		0.905638	0.976521161
268936	4	4	4.47	4.79	0.099750825	Up		0.914182	0.97976667
12368	1	1	4.61	4.94	0.099744291	Up		0.947628	0.981724675
107221	1	1	4.89	5.24	0.099732347	Up		0.947628	0.985774795
15415	2	2	10.76	11.53	0.099714435	Up		0.93456	0.987112688
19682	4	4	21.66	23.21	0.09971328	Up		0.914182	0.981663237
216864	3	3	13.14	14.08	0.099682058	Up		0.923686	0.981733453
12514	3	3	16.22	17.38	0.099654263	Up		0.923686	0.984878422
16450	5	5	8.25	8.84	0.09965225	Up		0.905638	0.977534544
381280	11	11	8.67	9.29	0.099646603	Up		0.865484	0.949339885
67830	2	2	8.95	9.59	0.099643133	Up		0.93456	0.979774948
68159	7	7	39.72	42.56	0.099632528	Up		0.890562	0.969308882
29816	7	7	10.63	11.39	0.09962615	Up		0.890562	0.968548739
67279	2	2	11.61	12.44	0.099618513	Up		0.93456	0.987488731
75677	6	6	41.55	44.52	0.099605116	Up		0.897816	0.971738675
19183	3	3	20.99	22.49	0.099581494	Up		0.923686	0.987028536
73318	5	5	20.99	22.49	0.099581494	Up		0.905638	0.977154279
67160	1	1	4.34	4.65	0.099535674	Up		0.947628	0.969533086
70435	3	3	4.34	4.65	0.099535674	Up		0.923686	0.980105994

224997	2	2	2.52	2.7	0.099535674	Up		0.93456	0.989373252
16881	2	2	4.2	4.5	0.099535674	Up		0.93456	0.987990568
69961	1	1	8.26	8.85	0.099535674	Up		0.947628	0.98319359
231803	1	1	2.1	2.25	0.099535674	Up		0.947628	0.982703463
228564	3	3	4.76	5.1	0.099535674	Up		0.923686	0.985509835
18140	1	1	1.96	2.1	0.099535674	Up		0.947628	0.980016479
94040	2	2	1.12	1.2	0.099535674	Up		0.93456	0.986236368
73247	2	2	11.48	12.3	0.099535674	Up		0.93456	0.985236762
12161	2	2	4.48	4.8	0.099535674	Up		0.93456	0.985111953
26564	2	2	3.36	3.6	0.099535674	Up		0.93456	0.984862432
75901	2	2	2.38	2.55	0.099535674	Up		0.93456	0.983492318
104776	2	2	3.92	4.2	0.099535674	Up		0.93456	0.982622411
330721	1	1	2.8	3	0.099535674	Up		0.947628	0.970368273
72567	1	1	1.26	1.35	0.099535674	Up		0.947628	0.97001016
20286	1	1	1.12	1.2	0.099535674	Up		0.947628	0.969056481
68018	1	1	1.26	1.35	0.099535674	Up		0.947628	0.964788032
12576	1	1	2.8	3	0.099535674	Up		0.947628	0.960908188
12866	1	1	9.24	9.9	0.099535674	Up		0.947628	0.959505061
14957	1	1	8.96	9.6	0.099535674	Up		0.947628	0.959388318
69747	3	3	26.89	28.81	0.099499905	Up		0.923686	0.983869842
67706	3	3	24.51	26.26	0.099496432	Up		0.923686	0.98766271
68576	4	4	30.54	32.72	0.099472686	Up		0.914182	0.981916667
12651	3	3	11.49	12.31	0.099451964	Up		0.923686	0.984626083
12034	4	4	20.04	21.47	0.099439683	Up		0.914182	0.982804706
19332	1	1	9.67	10.36	0.099436208	Up		0.947628	0.981846917
258508	3	3	22.57	24.18	0.099407826	Up		0.923686	0.980356021

170767	2	2	6.17	6.61	0.099379782	Up		0.93456	0.988492915
20262	1	1	6.03	6.46	0.099376163	Up		0.947628	0.971444202
241525	1	1	9.26	9.92	0.099327927	Up		0.947628	0.973242729
241732	2	2	4.35	4.66	0.099314554	Up		0.93456	0.990003025
13716	2	2	4.35	4.66	0.099314554	Up		0.93456	0.985861278
320717	3	3	4.35	4.66	0.099314554	Up		0.923686	0.984121793
75571	2	2	13.05	13.98	0.099314554	Up		0.93456	0.979898407
11532	2	2	8.56	9.17	0.099310937	Up		0.93456	0.977680885
74482	1	1	8	8.57	0.099295204	Up		0.947628	0.979894692
72826	2	2	3.65	3.91	0.099272144	Up		0.93456	0.981134716
75475	2	2	3.37	3.61	0.099250246	Up		0.93456	0.978049775
231279	2	2	2.81	3.01	0.099193357	Up		0.93456	0.980392557
12417	2	2	7.31	7.83	0.099140901	Up		0.93456	0.976330667
27078	2	2	17.01	18.22	0.099139818	Up		0.93456	0.989499142
74351	1	1	2.39	2.56	0.099133192	Up		0.947628	0.958571917
230767	3	3	9.14	9.79	0.099114695	Up		0.923686	0.980731301
24115	2	2	6.47	6.93	0.09908964	Up		0.93456	0.986736932
104910	1	1	4.22	4.52	0.099079774	Up		0.947628	0.979529514
54614	1	1	2.11	2.26	0.099079774	Up		0.947628	0.968104676
16336	2	2	24.06	25.77	0.099055895	Up		0.93456	0.984613036
433466	2	2	9.85	10.55	0.099047369	Up		0.93456	0.981258519
69668	1	1	3.94	4.22	0.099047369	Up		0.947628	0.972042972
17931	2	2	3.94	4.22	0.099047369	Up		0.93456	0.980516172
214253	2	2	5.91	6.33	0.099047369	Up		0.93456	0.980268973
21380	2	2	7.6	8.14	0.099029376	Up		0.93456	0.988618581

100502825	2	2	20.69	22.16	0.099024236	Up	0.93456	0.977066687
66084	1	1	3.66	3.92	0.099010006	Up	0.947628	0.975409771
192657	1	1	1.83	1.96	0.099010006	Up	0.947628	0.972762471
237886	3	3	5.21	5.58	0.09898175	Up	0.923686	0.986268601
108755	1	1	5.07	5.43	0.098966451	Up	0.947628	0.985528382
67078	1	1	6.62	7.09	0.09895441	Up	0.947628	0.970487703
70020	1	1	6.62	7.09	0.09895441	Up	0.947628	0.966680469
245857	2	2	4.93	5.28	0.098950283	Up	0.93456	0.989121567
17904	6	6	65.65	70.31	0.098934883	Up	0.897816	0.972118014
66192	1	1	9.16	9.81	0.098905538	Up	0.947628	0.958805033
16562	3	3	2.96	3.17	0.098885665	Up	0.923686	0.982989027
17319	4	4	51.18	54.81	0.098858987	Up	0.914182	0.979640492
71660	2	2	19.74	21.14	0.098853387	Up	0.93456	0.984488386
26396	1	1	2.82	3.02	0.098853387	Up	0.947628	0.960674048
217715	1	1	4.23	4.53	0.098853387	Up	0.947628	0.974927372
239933	1	1	11.57	12.39	0.098787323	Up	0.947628	0.978678489
24110	1	1	3.81	4.08	0.098778154	Up	0.947628	0.981358132
233908	1	1	3.67	3.93	0.098749249	Up	0.947628	0.987255864
18537	1	1	3.53	3.78	0.098718051	Up	0.947628	0.976617858
217039	1	1	2.26	2.42	0.098684275	Up	0.947628	0.985405222
14681	1	1	2.26	2.42	0.098684275	Up	0.947628	0.977465302
233406	1	1	2.26	2.42	0.098684275	Up	0.947628	0.971085294
68153	1	1	4.38	4.69	0.098657053	Up	0.947628	0.972282687
20054	2	2	27.27	29.2	0.098653669	Up	0.93456	0.98014542
15399	2	2	7.63	8.17	0.098653021	Up	0.93456	0.985486473

433256	1	1	2.12	2.27	0.098628033	Up	0.947628	0.966917546
20479	2	2	4.1	4.39	0.09859703	Up	0.93456	0.988116107
170732	1	1	5.09	5.45	0.098590574	Up	0.947628	0.982948465
23833	3	3	40.16	43	0.098577391	Up	0.923686	0.987408942
22410	1	1	2.97	3.18	0.098563834	Up	0.947628	0.960440023
216821	1	1	5.52	5.91	0.098489863	Up	0.947628	0.967985832
236511	2	2	1.84	1.97	0.098489863	Up	0.93456	0.98836728
66541	1	1	7.22	7.73	0.098469577	Up	0.947628	0.974325045
67886	1	1	0.85	0.91	0.098403704	Up	0.947628	0.987998069
71995	2	2	3.97	4.25	0.098323834	Up	0.93456	0.985736311
52187	3	3	3.97	4.25	0.098323834	Up	0.923686	0.981858866
624219	1	1	7.8	8.35	0.098302074	Up	0.947628	0.982581008
21894	2	2	1.56	1.67	0.098302074	Up	0.93456	0.984363767
13822	1	1	1.56	1.67	0.098302074	Up	0.947628	0.967510747
66916	2	2	26.67	28.55	0.098272921	Up	0.93456	0.985986276
208638	1	1	4.26	4.56	0.098180394	Up	0.947628	0.962550365
12122	1	1	3.55	3.8	0.098180394	Up	0.947628	0.980625866
72002	1	1	3.55	3.8	0.098180394	Up	0.947628	0.978799974
232223	1	1	2.7	2.89	0.098110085	Up	0.947628	0.960089199
12654	1	1	3.98	4.26	0.098085	Up	0.947628	0.988865386
66152	11	11	178.54	191.1	0.098080447	Up	0.865484	0.949089663
67248	2	2	33.27	35.61	0.098060569	Up	0.93456	0.97866521
21973	1	1	1.28	1.37	0.098032083	Up	0.947628	0.985035926
12829	3	3	2.56	2.74	0.098032083	Up	0.923686	0.982737656
53883	2	2	1.28	1.37	0.098032083	Up	0.93456	0.979034843
16530	1	1	5.98	6.4	0.097926421	Up	0.947628	0.977950216

58865	1	1	3.56	3.81	0.097913757	Up	0.947628	0.979164608
54612	1	1	3.56	3.81	0.097913757	Up	0.947628	0.972522519
192160	2	2	3.56	3.81	0.097913757	Up	0.93456	0.981382352
228859	1	1	4.13	4.42	0.097904588	Up	0.947628	0.965733324
56501	1	1	1.14	1.22	0.097847323	Up	0.947628	0.973843718
233315	3	3	3.85	4.12	0.097785892	Up	0.923686	0.982109789
30936	1	1	3.28	3.51	0.097775216	Up	0.947628	0.974204669
214424	1	1	2.71	2.9	0.097760049	Up	0.947628	0.970248873
19712	1	1	1.57	1.68	0.097696674	Up	0.947628	0.971803375
70369	1	1	3.14	3.36	0.097696674	Up	0.947628	0.976496915
19192	1	1	2.57	2.75	0.097663259	Up	0.947628	0.973964005
12672	1	1	2.43	2.6	0.097555309	Up	0.947628	0.960791104
235574	1	1	1.43	1.53	0.097516506	Up	0.947628	0.975771883
116847	1	1	1.86	1.99	0.097465809	Up	0.947628	0.962667878
27103	2	2	2.72	2.91	0.097412502	Up	0.93456	0.9781728
23912	1	1	3.15	3.37	0.097396763	Up	0.947628	0.961845888
97487	1	1	0.86	0.92	0.097297201	Up	0.947628	0.965260447
404545	1	1	1.72	1.84	0.097297201	Up	0.947628	0.964080277
237987	1	1	2.87	3.07	0.097187919	Up	0.947628	0.961728575
242126	2	2	2.01	2.15	0.097141158	Up	0.93456	0.978418943
13855	1	1	1.58	1.69	0.097098688	Up	0.947628	0.98676168
71782	14.04	14	17.82	19.06	0.097050782	Up	0.849476	0.933750303
66832	1	1	3.45	3.69	0.097024454	Up	0.947628	0.98700871
17285	1	1	3.02	3.23	0.096985615	Up	0.947628	0.961142441
12727	1	1	1.44	1.54	0.096861539	Up	0.947628	0.983316198
330463	1	1	1.44	1.54	0.096861539	Up	0.947628	0.982213825

81879	1	1	0.72	0.77	0.096861539	Up	0.947628	0.97300254
338368	1	1	2.45	2.62	0.096785063	Up	0.947628	0.975530445
74245	1	1	2.45	2.62	0.096785063	Up	0.947628	0.96822355
93836	2	2	2.74	2.93	0.096724771	Up	0.93456	0.976698538
81535	1	1	2.02	2.16	0.096676019	Up	0.947628	0.983561505
20024	1	1	2.02	2.16	0.096676019	Up	0.947628	0.959271604
17988	1	1	2.31	2.47	0.09661819	Up	0.947628	0.989485831
14426	1	1	1.3	1.39	0.09657326	Up	0.947628	0.980138296
75750	1	1	3.18	3.4	0.096507981	Up	0.947628	0.963609017
224826	1	1	0.87	0.93	0.096215315	Up	0.947628	0.986267991
56036	1	1	2.76	2.95	0.096046687	Up	0.947628	0.986638212
70461	1	1	1.31	1.4	0.095860015	Up	0.947628	0.9610253
17936	1	1	1.31	1.4	0.095860015	Up	0.947628	0.958688461
278279	1	1	1.17	1.25	0.095419565	Up	0.947628	0.965851615
107515	1	1	1.32	1.41	0.095157233	Up	0.947628	0.986021331
12396	2	2	2.2	2.35	0.095157233	Up	0.93456	0.985611376
67579	2	2	1.76	1.88	0.095157233	Up	0.93456	0.979528122
15405	1	1	2.2	2.35	0.095157233	Up	0.947628	0.959855458
227545	1	1	1.93	2.06	0.09404349	Up	0.947628	0.987874291
140484	1	1	1.19	1.27	0.093866923	Up	0.947628	0.987626827
213391	1	1	1.05	1.12	0.093109404	Up	0.947628	0.985282093
18028	1	1	0.75	0.8	0.093109404	Up	0.947628	0.974445451
56758	1	1	1.2	1.28	0.093109404	Up	0.947628	0.958338914
17258	1	1	1.21	1.29	0.092364018	Up	0.947628	0.977586485
63958	1	1	1.21	1.29	0.092364018	Up	0.947628	0.9651423
58208	1	1	0.91	0.97	0.092118202	Up	0.947628	0.982825949

12499	1	1	1.37	1.46	0.091792476	Up	0.947628	0.985651574
240055	1	1	1.08	1.15	0.090602549	Up	0.947628	0.963491274
14204	17	16.88	55.72	59.3	0.089836847	Up	0.972772	0.977116829
20698	10	9	36.57	38.9	0.089109529	Up	0.94858	0.957439857
208439	1	1	0.95	1.01	0.088355874	Up	0.947628	0.972162814
52463	3	2.99	1.43	1.52	0.088056177	Up	0.754082	0.850232564
74370	1	1	0.96	1.02	0.087462841	Up	0.947628	0.96384459
68421	25.54	26.11	32.56	34.5	0.083495662	Up	0.695334	0.803147448
20493	18	18	79.96	84.69	0.082913157	Up	0.83057	0.920017701
67417	68.36	67.31	69.62	73.5	0.078242436	Up	0.747068	0.849925188
26913	60	59	94	99.11	0.076369873	Up	0.77005	0.864021851
194952	16.31	16.04	24.73	26.06	0.075574844	Up	0.839728	0.929172432
75739	12.79	12.57	17.17	18.09	0.075302369	Up	0.859936	0.944250126
75141	1.02	1	2.43	2.56	0.075187496	Up	0.947628	0.984298164
50883	10.13	9.94	30.37	31.97	0.074071836	Up	0.94858	0.958137191
74558	0.25	0.24	0.19	0.2	0.074000581	Up	0.96507	0.97184406
319179	1.01	2	16.37	17.2	0.071354243	Up	0.573542	0.72408481
242474	8.29	8.11	8.01	8.4	0.068587085	Up	0.883768	0.963678879
72656	32	32	46.76	49.01	0.067801217	Up	0.777346	0.871738014
225994	214.48	209.52	888.38	930.2	0.066364026	Up	0.633312	0.766180452
329540	10	10	10.66	11.14	0.063541795	Up	0.87128	0.952558255
109785	39	38	58.45	61.06	0.063024565	Up	0.844258	0.928505263
279028	27.49	26.78	53.42	55.8	0.062885146	Up	0.902856	0.975290767
240660	33.7	32.81	65.04	67.89	0.061871828	Up	0.871324	0.952105976
12846	42.76	41.63	135.53	141.45	0.061679945	Up	0.831976	0.921452455
104885	143	139	405.69	422.75	0.059427013	Up	0.725922	0.828814073

268857	1	2	3.37	3.51	0.058722439	Up	0.573542	0.722987878
107605	51.42	49.89	88.44	92	0.056934836	Up	0.878294	0.95821219
68794	3	3	2.25	2.34	0.056583528	Up	0.923686	0.980606176
100310872	283.8	275.24	380.64	395.83	0.056453782	Up	0.625484	0.759138326
246179	1.03	1	2.04	2.12	0.055495113	Up	0.947628	0.963255874
12015	32	31	149.24	154.96	0.054261594	Up	0.876224	0.957208862
18817	30	29	91.33	94.64	0.05136124	Up	0.88642	0.965684842
230119	19	19	26.82	27.79	0.051256597	Up	0.82618	0.915520546
17294	29	28	81.3	84.15	0.049707919	Up	0.891736	0.968812542
56386	3.03	2.93	3.48	3.6	0.0489096	Up	0.754082	0.855448715
383103	28	27	56.5	58.42	0.048211491	Up	0.89721	0.971967766
212632	10	9	11.64	12.03	0.047545584	Up	0.94858	0.958253512
21816	1	1	2.44	2.52	0.046542586	Up	0.947628	0.973362867
50907	27	26	33.93	35.03	0.046029558	Up	0.902856	0.975164171
435811	4.11	3.95	15.19	15.68	0.04580369	Up	0.804208	0.897627735
170720	104	100	175.77	181.22	0.044053329	Up	0.824654	0.914194769
229524	23	22	85.25	87.41	0.036098504	Up	0.927438	0.98358547
231004	262.52	251.01	676.15	693.11	0.035740998	Up	0.7586	0.853476284
20869	11	10	29.65	30.35	0.033664412	Up	0.962774	0.970589103
245174	602.53	574.62	580.11	593.23	0.032265069	Up	0.701956	0.807992236
140481	34.33	32.68	34.79	35.52	0.029958903	Up	0.965724	0.970623192
18426	20	19	46.29	47.15	0.026557208	Up	0.948528	0.958666524
224129	20	19	26.39	26.88	0.026541787	Up	0.948528	0.958550096
12448	20	19	44.39	45.21	0.026407209	Up	0.948528	0.958433696
330657	59	56	156.78	159.53	0.025086221	Up	0.92136	0.985555542

233575	19	18	62.61	63.59	0.022406808	Up	0.956206	0.964669026
22644	17	17	34.57	35.09	0.021539344	Up	0.835082	0.924523327
21343	75	71	223.19	226.5	0.021238661	Up	0.92468	0.980910541
21356	30.66	29	74.97	76.03	0.020255389	Up	0.88642	0.965558426
11733	5	4	9.71	9.84	0.01918702	Up	0.841222	0.925900564
209200	68.17	64.36	87.82	88.91	0.017796158	Up	0.954554	0.963119175
19946	18	17	239.54	242.23	0.016110966	Up	0.964272	0.971393137
50524	17	16	23.8	24.02	0.013274578	Up	0.972772	0.9767631
217779	17	16	47.66	48.09	0.012957975	Up	0.972772	0.976998891
229780	17	16	36.09	36.41	0.012735598	Up	0.972772	0.976880981
16880	2.44	3	5.75	5.8	0.012490944	Up	0.623204	0.759584076
100503041	67	63	114.14	115.08	0.011832658	Up	0.959072	0.967208594
65246	79.68	74.89	35.01	35.29	0.011492368	Up	0.974878	0.978759633
170756	10.88	10.22	26.82	27.02	0.010718437	Up	0.87128	0.95230801
12443	33	31	58.18	58.6	0.010377368	Up	0.972104	0.976563731
66356	26.17	25	36.72	36.97	0.009788985	Up	0.908686	0.97828648
16898	33	31	225.91	227.4	0.009484121	Up	0.972104	0.976681645
67867	161	151	589.82	592.98	0.007708703	Up	0.95741	0.965766591
16601	16	15	32.87	33.04	0.00744223	Up	0.981766	0.984131416
72179	16	15	33.49	33.66	0.007304801	Up	0.981766	0.984249986
83396	16	15	29.6	29.75	0.007292493	Up	0.981766	0.984368585
22084	223	210	242.43	243.63	0.007123555	Up	0.916974	0.981116196
232821	19	20	81.89	82.29	0.007029835	Up	0.703834	0.809034772
14433	67	60	336.89	338.48	0.006792986	Up	0.823882	0.913947757
414758	21.92	20.54	29.85	29.98	0.006269452	Up	0.941196	0.983016432
19889	9.51	8.91	14.37	14.43	0.006011238	Up	0.932788	0.98862934

28105	20.79	19.46	30.49	30.6	0.005195503	Up		0.948528	0.95878298
226040	78	73	213.16	213.87	0.004797389	Up		0.979232	0.982064538
17882	3.2	3	3.51	3.52	0.004104398	Up		0.923686	0.985383488
223254	15	14	20.62	20.64	0.001398638	Up		0.99132	0.992990697
83771	15	14	28.59	28.61	0.001008878	Up		0.99132	0.992632217
15444	15	14	67.95	67.97	0.000424572	Up		0.99132	0.992871175
100041230	9.98	0	178.97	0.001	-17.44935825	Down		0.0027532	0.061091673
319152	9.52	0	139.48	0.001	-17.08969874	Down		0.0027532	0.060287835
319191	8.44	0	135.66	0.001	-17.04963587	Down		0.00532054	0.099265052
11957	9	0	76.16	0.001	-16.21674586	Down		0.0027532	0.060767579
18545	5	0	75.79	0.001	-16.20971989	Down		0.0383984	0.310508344
67671	3	0	71.52	0.001	-16.12605912	Down		0.1434012	0.571202195
20044	6	0	69.79	0.001	-16.09073271	Down		0.01986984	0.209022678
102436	34	0	58.62	0.001	-15.83910535	Down		1.94E-10	3.04E-08
19942	6	0	57.02	0.001	-15.79918042	Down		0.01986984	0.216693235
170458	5	0	56.38	0.001	-15.78289586	Down		0.0383984	0.308112909
22121	8	0	52.91	0.001	-15.6912528	Down		0.00532054	0.096243942
14912	10	0	52.22	0.001	-15.67231484	Down		0.00142468	0.036930724
68176	8	0	50.98	0.001	-15.63764375	Down		0.00532054	0.097301568
80334	16	0	49.3	0.001	-15.58930003	Down		2.74E-05	0.001422517
72654	6	0	49.2	0.001	-15.5863707	Down		0.01986984	0.214444797
72273	4	0	49.19	0.001	-15.58607743	Down		0.074205	0.415797848
13011	7	0	48.88	0.001	-15.57695666	Down		0.01028194	0.150098286
66561	9	0	47.75	0.001	-15.54321311	Down		0.0027532	0.061584347
72514	12	0	45.76	0.001	-15.48179943	Down		0.000381486	0.013393861
69386	2.75	0	45.68	0.001	-15.47927503	Down		0.277122	0.62593164

17035	7	0	43.97	0.001	-15.42423191	Down	0.01028194	0.147765152
22226	4	0	43.63	0.001	-15.41303285	Down	0.074205	0.419755136
14227	4	0	43.48	0.001	-15.40806432	Down	0.074205	0.439473171
326620	2.72	0	43.47	0.001	-15.40773248	Down	0.277122	0.647915752
326619	2.71	0	42.78	0.001	-15.38464886	Down	0.277122	0.641784626
20195	3	0	42.47	0.001	-15.37415649	Down	0.1434012	0.529153608
67673	3	0	42.12	0.001	-15.36221782	Down	0.1434012	0.575888699
227289	6	0	41.72	0.001	-15.34845154	Down	0.01986984	0.213889959
17095	11	0	40.93	0.001	-15.32087105	Down	0.000737222	0.022145936
27425	3	0	40.33	0.001	-15.29956579	Down	0.1434012	0.542875971
319156	2	0	39.84	0.001	-15.28193003	Down	0.277122	0.639648311
67127	4	0	39.3	0.001	-15.26224169	Down	0.074205	0.405689754
55938	4	0	38.18	0.001	-15.22052948	Down	0.074205	0.417202571
319184	2.65	0	37.88	0.001	-15.20914871	Down	0.277122	0.652499197
75284	7	0	37.59	0.001	-15.19806129	Down	0.01028194	0.147510384
14109	3	0	37.17	0.001	-15.18185107	Down	0.1434012	0.556549154
18038	8	0	37	0.001	-15.17523765	Down	0.00532054	0.100163379
107732	9	0	36.26	0.001	-15.1460913	Down	0.0027532	0.060606818
20103	4	0	36.25	0.001	-15.14569337	Down	0.074205	0.413569863
64295	7	0	35.52	0.001	-15.11634396	Down	0.01028194	0.146000039
54198	7	0	34.62	0.001	-15.0793181	Down	0.01028194	0.149835416
93674	9	0	33.1	0.001	-15.0145436	Down	0.0027532	0.061917236
20471	14	0	32.99	0.001	-15.00974116	Down	0.00010215	0.004292896
100504491	4	0	32.05	0.001	-14.96803674	Down	0.074205	0.448084038
170758	5	0	31.83	0.001	-14.95809954	Down	0.0383984	0.315412721
27279	4	0	31.63	0.001	-14.94900594	Down	0.074205	0.416639545

12925	2	0	31.24	0.001	-14.93110683	Down	0.277122	0.708645409
69752	5	0	31.17	0.001	-14.92787053	Down	0.0383984	0.321765445
319182	2.29	0	31.17	0.001	-14.92787053	Down	0.277122	0.620875649
72184	9	0	31.05	0.001	-14.92230565	Down	0.0027532	0.061419242
21946	3	0	30.85	0.001	-14.91298287	Down	0.1434012	0.573122663
69071	6	0	30.68	0.001	-14.90501086	Down	0.01986984	0.210888952
13057	3	0	30.33	0.001	-14.88845788	Down	0.1434012	0.561525358
67267	2	0	30.32	0.001	-14.88798213	Down	0.277122	0.645377039
11537	4	0	29.88	0.001	-14.86689253	Down	0.074205	0.412740511
14319	4	0	29.29	0.001	-14.83812057	Down	0.074205	0.422917675
64818	8	0	29.13	0.001	-14.83021808	Down	0.00532054	0.097088187
381045	4	0	28.41	0.001	-14.79411121	Down	0.074205	0.407295386
69534	4	0	28.23	0.001	-14.78494151	Down	0.074205	0.422628203
20692	11	0	27.99	0.001	-14.77262387	Down	0.000737222	0.022226175
68263	6	0	27.46	0.001	-14.74504401	Down	0.01986984	0.21224254
21922	4	0	27.27	0.001	-14.73502708	Down	0.074205	0.425834348
51813	6	0	27.18	0.001	-14.73025784	Down	0.01986984	0.219570968
100294583	6	0	27.05	0.001	-14.72334097	Down	0.01986984	0.215002521
68701	3	0	26.93	0.001	-14.71692661	Down	0.1434012	0.527516085
68276	8	0	26.9	0.001	-14.71531855	Down	0.00532054	0.09687574
109042	4	0	26.8	0.001	-14.70994538	Down	0.074205	0.432394821
103768	7	0	26.66	0.001	-14.70238916	Down	0.01028194	0.149052304
78373	5	0	26.55	0.001	-14.69642424	Down	0.0383984	0.304008645
232910	3	0	26.12	0.001	-14.67286728	Down	0.1434012	0.546355946
225887	4	0	26.06	0.001	-14.66954946	Down	0.074205	0.440727912
497106	2	0	26.04	0.001	-14.66844183	Down	0.277122	0.641070937

77772	9	0	25.88	0.001	-14.65955	Down	0.0027532	0.062085033
435791	2	0	25.85	0.001	-14.65787666	Down	0.277122	0.714796082
20753	3	0	25.81	0.001	-14.65564252	Down	0.1434012	0.560207223
67513	4	0	25.51	0.001	-14.63877528	Down	0.074205	0.444535497
14276	4	0	25.49	0.001	-14.63764375	Down	0.074205	0.446141478
81845	5	0	25.43	0.001	-14.63424384	Down	0.0383984	0.323393812
20429	6	0	25.39	0.001	-14.63197277	Down	0.01986984	0.211699025
14430	8	0	25.31	0.001	-14.62741989	Down	0.00532054	0.100390506
27367	5	0	25.03	0.001	-14.61137067	Down	0.0383984	0.309305989
320234	11	0	25.02	0.001	-14.61079417	Down	0.000737222	0.022066274
94181	7	0	24.85	0.001	-14.60095823	Down	0.01028194	0.149573466
170654	1	0	24.63	0.001	-14.58812901	Down	0.53554	0.746311898
26462	7	0	24.61	0.001	-14.58695704	Down	0.01028194	0.146751325
107197	3	0	24.54	0.001	-14.58284763	Down	0.1434012	0.586359403
18567	4	0	24.45	0.001	-14.57754684	Down	0.074205	0.416358601
67128	14	0	24.42	0.001	-14.57577558	Down	0.00010215	0.004271324
21835	4	0	24.39	0.001	-14.57400214	Down	0.074205	0.436985
80885	7	0	24.29	0.001	-14.56807487	Down	0.01028194	0.148793083
76167	4	0	24.19	0.001	-14.56212315	Down	0.074205	0.4252478
100040608	6	0	24.04	0.001	-14.55314928	Down	0.01986984	0.210352339
11847	5	0	24.01	0.001	-14.55134778	Down	0.0383984	0.31020688
11807	3	0	24.01	0.001	-14.55134778	Down	0.1434012	0.526817389
68977	4	0	23.92	0.001	-14.54592977	Down	0.074205	0.443577446
387510	3	0	23.81	0.001	-14.53928	Down	0.1434012	0.565517244
242691	7	0	23.6	0.001	-14.52649924	Down	0.01028194	0.148534762
105638	4	0	23.01	0.001	-14.48997336	Down	0.074205	0.449061676

22695	6	0	23.01	0.001	-14.48997336	Down	0.01986984	0.21812261
13350	6	0	22.87	0.001	-14.48116875	Down	0.01986984	0.214167019
68484	2	0	22.59	0.001	-14.46339665	Down	0.277122	0.607783912
77777	6	0	22.31	0.001	-14.44540289	Down	0.01986984	0.215282472
66985	5	0	22.19	0.001	-14.43762205	Down	0.0383984	0.319832919
100039781	3	0	22.11	0.001	-14.4324114	Down	0.1434012	0.558633607
73016	7	0	22.11	0.001	-14.4324114	Down	0.01028194	0.143791635
56358	3	0	22.09	0.001	-14.4311058	Down	0.1434012	0.526584901
67836	4	0	22.07	0.001	-14.42979901	Down	0.074205	0.412189456
22264	2	0	21.99	0.001	-14.42455998	Down	0.277122	0.645919373
70645	5	0	21.8	0.001	-14.41204051	Down	0.0383984	0.313248124
94179	5	0	21.76	0.001	-14.40939094	Down	0.0383984	0.318557414
110454	3	0	21.63	0.001	-14.40074604	Down	0.1434012	0.554995993
21937	7	0	21.59	0.001	-14.39807562	Down	0.01028194	0.144033708
14431	3	0	21.54	0.001	-14.39473063	Down	0.1434012	0.549627538
207818	3	0	21.4	0.001	-14.38532318	Down	0.1434012	0.588383326
69890	9	0	21.25	0.001	-14.37517522	Down	0.0027532	0.061255019
12055	3	0	21.06	0.001	-14.36221782	Down	0.1434012	0.57394968
19241	2	0	20.92	0.001	-14.35259523	Down	0.277122	0.622888212
117109	3	0	20.84	0.001	-14.34706766	Down	0.1434012	0.575610895
20102	3	0	20.82	0.001	-14.34568245	Down	0.1434012	0.576166772
72482	3	0	20.82	0.001	-14.34568245	Down	0.1434012	0.547609631
21936	3	0	20.5	0.001	-14.32333629	Down	0.1434012	0.574502352
72083	3	0	20.43	0.001	-14.31840158	Down	0.1434012	0.554480198
11705	5	0	20.35	0.001	-14.31274117	Down	0.0383984	0.312635114
22376	6.31	0	20.31	0.001	-14.30990262	Down	0.01986984	0.218699654

21858	11	0	20.26	0.001	-14.30634655	Down	0.000737222	0.022306997
68039	2	0	20.22	0.001	-14.30349538	Down	0.277122	0.623730636
100039246	3	0	20.22	0.001	-14.30349538	Down	0.1434012	0.531747498
50529	4	0	20.13	0.001	-14.29705955	Down	0.074205	0.420612946
59050	8	0	20.01	0.001	-14.28843355	Down	0.00532054	0.096453624
69876	3	0	20	0.001	-14.28771238	Down	0.1434012	0.589546139
71325	6	0	19.6	0.001	-14.25856603	Down	0.01986984	0.217262731
21389	5	0	19.56	0.001	-14.25561875	Down	0.0383984	0.304298178
12265	15	0	19.38	0.001	-14.24228095	Down	5.29E-05	0.002513379
546143	2	0	19.34	0.001	-14.23930017	Down	0.277122	0.629864016
54194	6	0	19.3	0.001	-14.23631323	Down	0.01986984	0.218989323
240819	4	0	19.24	0.001	-14.23182118	Down	0.074205	0.425540872
68440	4	0	19.21	0.001	-14.2295699	Down	0.074205	0.410545083
64384	4	0	19.02	0.001	-14.21522963	Down	0.074205	0.413293042
72615	7	0	18.97	0.001	-14.21143206	Down	0.01028194	0.148277336
69181	6	0	18.93	0.001	-14.20838679	Down	0.01986984	0.210085055
66230	2	0	18.9	0.001	-14.20609861	Down	0.277122	0.715683477
21405	9	0	18.84	0.001	-14.20151134	Down	0.0027532	0.060446906
665180	4	0	18.83	0.001	-14.20074538	Down	0.074205	0.414680863
381339	3	0	18.77	0.001	-14.19614103	Down	0.1434012	0.532459342
66494	3	0	18.65	0.001	-14.18688801	Down	0.1434012	0.582068968
386612	4	0	18.62	0.001	-14.18456545	Down	0.074205	0.438225554
66328	2	0	18.58	0.001	-14.18146288	Down	0.277122	0.671304851
217149	2	0	18.58	0.001	-14.18146288	Down	0.277122	0.623393393
100038882	2	0	18.43	0.001	-14.16976845	Down	0.277122	0.643037413
17189	3	0	18.34	0.001	-14.16270602	Down	0.1434012	0.525425533

320208	2	0	18.32	0.001	-14.16113188	Down	0.277122	0.681825004
232983	2	0	18.32	0.001	-14.16113188	Down	0.277122	0.627635319
268482	5	0	18.24	0.001	-14.15481811	Down	0.0383984	0.302282958
12406	6	0	18.14	0.001	-14.14688684	Down	0.01986984	0.219279759
224640	7	0	18.08	0.001	-14.14210706	Down	0.01028194	0.145751316
11947	5	0	17.99	0.001	-14.13490757	Down	0.0383984	0.324378768
246782	5	0	17.93	0.001	-14.13008787	Down	0.0383984	0.318240126
219114	6	0	17.89	0.001	-14.12686577	Down	0.01986984	0.210620304
13349	3	0	17.74	0.001	-14.11471839	Down	0.1434012	0.569566294
105245	7	0	17.69	0.001	-14.11064643	Down	0.01028194	0.150626801
171095	6	0	17.66	0.001	-14.10819772	Down	0.01986984	0.211158287
18811	2	0	17.6	0.001	-14.10328781	Down	0.277122	0.711049079
16447	5	0	17.56	0.001	-14.10000522	Down	0.0383984	0.302569211
50724	4	0	17.53	0.001	-14.09753838	Down	0.074205	0.432697831
27756	2	0	17.51	0.001	-14.09589146	Down	0.277122	0.697077437
56702	2	0	17.5	0.001	-14.0950673	Down	0.277122	0.646643904
216820	4	0	17.49	0.001	-14.09424267	Down	0.074205	0.432092236
66101	2	0	17.48	0.001	-14.09341756	Down	0.277122	0.629348297
74325	4	0	17.22	0.001	-14.07179752	Down	0.074205	0.436367353
110877	8	0	17.21	0.001	-14.07095948	Down	0.00532054	0.099712192
66292	1	0	17.11	0.001	-14.06255214	Down	0.53554	0.707337832
26442	3	0	16.85	0.001	-14.04046097	Down	0.1434012	0.556808859
18439	2	0	16.84	0.001	-14.03960452	Down	0.277122	0.676424806
436199	2	0	16.82	0.001	-14.03789009	Down	0.277122	0.649192613
15467	7	0	16.76	0.001	-14.03273453	Down	0.01028194	0.146249612
68021	3	0	16.75	0.001	-14.03187348	Down	0.1434012	0.532697047

23918	4	0	16.62	0.001	-14.02063276	Down	0.074205	0.440413556
12843	13	0	16.62	0.001	-14.02063276	Down	0.000197405	0.007675749
20613	4	0	16.61	0.001	-14.01976445	Down	0.074205	0.446787124
66506	2	0	16.52	0.001	-14.01192607	Down	0.277122	0.61475131
66179	3	0	16.49	0.001	-14.00930378	Down	0.1434012	0.528450569
73162	4	0	16.23	0.001	-13.98637538	Down	0.074205	0.4451765
69583	4	0	16.17	0.001	-13.98103206	Down	0.074205	0.442623516
12345	4	0	16.17	0.001	-13.98103206	Down	0.074205	0.405157352
74192	3	0	16.11	0.001	-13.97566887	Down	0.1434012	0.536047343
67698	5	0	16.01	0.001	-13.96668569	Down	0.0383984	0.311113034
21944	4	0	16	0.001	-13.96578428	Down	0.074205	0.427307824
16825	5	0	15.98	0.001	-13.96397979	Down	0.0383984	0.312329508
76497	4	0	15.98	0.001	-13.96397979	Down	0.074205	0.423788473
14622	4	0	15.9	0.001	-13.95673915	Down	0.074205	0.442941037
668225	10	0	15.89	0.001	-13.9558315	Down	0.00142468	0.037046132
60532	6	0	15.85	0.001	-13.95219522	Down	0.01986984	0.215563153
15586	5.12	0	15.83	0.001	-13.95037364	Down	0.0383984	0.305461842
68044	3	0	15.82	0.001	-13.94946198	Down	0.1434012	0.546105897
58200	3	0	15.8	0.001	-13.94763694	Down	0.1434012	0.545856077
269951	4	0	15.77	0.001	-13.94489504	Down	0.074205	0.418900818
26425	5	0	15.76	0.001	-13.94397991	Down	0.0383984	0.32047451
57784	4	0	15.69	0.001	-13.93755773	Down	0.074205	0.428196813
19354	7.06	0	15.61	0.001	-13.93018292	Down	0.01028194	0.151159051
69101	3	0	15.5	0.001	-13.9199806	Down	0.1434012	0.572023675
14678	5	0	15.48	0.001	-13.91811785	Down	0.0383984	0.313555531
106039	7	0	15.48	0.001	-13.91811785	Down	0.01028194	0.146500039

78593	9	0	15.39	0.001	-13.90970561	Down	0.0027532	0.062253742
260423	2	0	15.3	0.001	-13.90124403	Down	0.277122	0.612628098
11370	5	0	15.28	0.001	-13.89935692	Down	0.0383984	0.317607442
74127	4	0	15.26	0.001	-13.89746734	Down	0.074205	0.447759104
17283	6	0	15.25	0.001	-13.89652162	Down	0.01986984	0.208495509
54123	4	0	15.21	0.001	-13.89273253	Down	0.074205	0.414402554
72124	4	0	15.18	0.001	-13.88988417	Down	0.074205	0.416920868
232816	8	0	15.12	0.001	-13.88417052	Down	0.00532054	0.099042983
52815	5	0	15.08	0.001	-13.88034881	Down	0.0383984	0.307816076
22433	5	0	15.05	0.001	-13.87747587	Down	0.0383984	0.306340447
12798	4	0	15.04	0.001	-13.87651695	Down	0.074205	0.449388504
11837	3	0	15.03	0.001	-13.87555739	Down	0.1434012	0.536288263
57320	2	0	15.01	0.001	-13.87363636	Down	0.277122	0.672087485
66079	1	0	14.92	0.001	-13.86495992	Down	0.53554	0.844462448
14872	3	0	14.91	0.001	-13.86399264	Down	0.1434012	0.54485908
232313	4	0	14.82	0.001	-13.85525783	Down	0.074205	0.413016592
53607	3	0	14.81	0.001	-13.85428402	Down	0.1434012	0.575333358
80796	2	0	14.74	0.001	-13.8474489	Down	0.277122	0.608104473
19989	2	0	14.73	0.001	-13.84646981	Down	0.277122	0.6442951
77744	5	0	14.69	0.001	-13.84254678	Down	0.0383984	0.316977268
330962	1	0	14.66	0.001	-13.83959748	Down	0.53554	0.788433889
71753	7	0	14.64	0.001	-13.83762793	Down	0.01028194	0.147256494
57816	2	0	14.64	0.001	-13.83762793	Down	0.277122	0.646825291
76252	4	0	14.63	0.001	-13.83664215	Down	0.074205	0.418333201
20422	1	0	14.56	0.001	-13.82972274	Down	0.53554	0.736444941
29818	6	0	14.56	0.001	-13.82972274	Down	0.01986984	0.21142831

76072	3	0	14.54	0.001	-13.82773965	Down	0.1434012	0.570110552
319189	1	0	14.5	0.001	-13.82376528	Down	0.53554	0.769509297
217138	3	0	14.48	0.001	-13.82177398	Down	0.1434012	0.594539803
11908	5	0	14.46	0.001	-13.81977993	Down	0.0383984	0.315101663
77634	3	0	14.45	0.001	-13.81878187	Down	0.1434012	0.534845982
27981	3	0	14.43	0.001	-13.81678368	Down	0.1434012	0.557068807
22070	2	0	14.42	0.001	-13.81578354	Down	0.277122	0.705609597
57423	1	0	14.4	0.001	-13.81378119	Down	0.53554	0.742086318
259301	1	0	14.37	0.001	-13.81077244	Down	0.53554	0.790111408
20091	2	0	14.36	0.001	-13.80976813	Down	0.277122	0.671109477
14620	4	0	14.34	0.001	-13.8077574	Down	0.074205	0.428791531
16373	5	0	14.33	0.001	-13.80675099	Down	0.0383984	0.309906
67963	7	0	14.31	0.001	-13.80473605	Down	0.01028194	0.148020801
53381	2	0	14.3	0.001	-13.80372753	Down	0.277122	0.704962446
320816	5	0	14.29	0.001	-13.8027183	Down	0.0383984	0.30575415
58867	2	0	14.27	0.001	-13.80069771	Down	0.277122	0.687107319
13041	3	0	14.19	0.001	-13.79258697	Down	0.1434012	0.524041012
66743	4	0	14.18	0.001	-13.79156991	Down	0.074205	0.42032662
215061	3	0	14.05	0.001	-13.77828251	Down	0.1434012	0.585783694
110557	2	0	14.04	0.001	-13.77725532	Down	0.277122	0.696866776
319162	1	0	14.04	0.001	-13.77725532	Down	0.53554	0.741715769
67729	5	0	13.99	0.001	-13.77210834	Down	0.0383984	0.307519814
11461	4	0	13.97	0.001	-13.7700444	Down	0.074205	0.441989839
22272	1	0	13.95	0.001	-13.7679775	Down	0.53554	0.702321251
269589	4	0	13.93	0.001	-13.76590764	Down	0.074205	0.427603743
66968	4	0	13.93	0.001	-13.76590764	Down	0.074205	0.411365626

53624	2	0	13.92	0.001	-13.76487159	Down	0.277122	0.641427583
11674	3	0	13.87	0.001	-13.75968017	Down	0.1434012	0.541398088
71963	5	0	13.84	0.001	-13.75655632	Down	0.0383984	0.307224122
14447	3	0	13.82	0.001	-13.75447	Down	0.1434012	0.575056089
12709	3	0	13.8	0.001	-13.75238065	Down	0.1434012	0.577561174
381979	5	0	13.8	0.001	-13.75238065	Down	0.0383984	0.309605704
26914	4	0	13.74	0.001	-13.74609438	Down	0.074205	0.430886117
211064	4	0	13.73	0.001	-13.74504401	Down	0.074205	0.405423378
52530	2	0	13.71	0.001	-13.74294095	Down	0.277122	0.679814906
71765	4	0	13.69	0.001	-13.74083483	Down	0.074205	0.436675958
215303	5	0	13.64	0.001	-13.73555602	Down	0.0383984	0.321441737
244421	8	0	13.62	0.001	-13.73343908	Down	0.00532054	0.098821905
83964	4	0	13.6	0.001	-13.73131903	Down	0.074205	0.430585638
72084	2	0	13.58	0.001	-13.72919586	Down	0.277122	0.668192455
629499	7	0	13.54	0.001	-13.72494012	Down	0.01028194	0.150362079
11993	3	0	13.52	0.001	-13.72280753	Down	0.1434012	0.525888667
76293	3	0	13.47	0.001	-13.71746223	Down	0.1434012	0.594836184
67695	1	0	13.44	0.001	-13.71424552	Down	0.53554	0.772979764
226999	7	0	13.42	0.001	-13.71209705	Down	0.01028194	0.145010208
83701	6	0	13.39	0.001	-13.70886834	Down	0.01986984	0.20981845
381867	4	0	13.38	0.001	-13.7077905	Down	0.074205	0.434219272
20973	3	0	13.37	0.001	-13.70671184	Down	0.1434012	0.549880823
259004	2	0	13.36	0.001	-13.70563239	Down	0.277122	0.685881071
66432	3	0	13.36	0.001	-13.70563239	Down	0.1434012	0.545107988
19729	2	0	13.33	0.001	-13.70238916	Down	0.277122	0.612790901
28064	4	0	13.27	0.001	-13.69588075	Down	0.074205	0.4124648

12350	3	0	13.25	0.001	-13.69370474	Down		0.1434012	0.57980631
73828	4	0	13.23	0.001	-13.69152544	Down		0.074205	0.424662865
106407	3	0	13.19	0.001	-13.68715694	Down		0.1434012	0.522434932
17714	8	0	13.16	0.001	-13.68387187	Down		0.00532054	0.097731155
59021	4	0	13.12	0.001	-13.6794801	Down		0.074205	0.408102978
56233	8	0	13.1	0.001	-13.67727919	Down		0.00532054	0.098164553
259144	2	0	13.06	0.001	-13.67286728	Down		0.277122	0.679014182
19156	5	0	13.01	0.001	-13.66733334	Down		0.0383984	0.324708421
14533	1	0	12.9	0.001	-13.65508345	Down		0.53554	0.72957242
66249	3	0	12.9	0.001	-13.65508345	Down		0.1434012	0.540417294
21376	3	0	12.86	0.001	-13.65060302	Down		0.1434012	0.538952749
19659	5	0	12.79	0.001	-13.64272864	Down		0.0383984	0.301997246
231724	3	0	12.78	0.001	-13.64160022	Down		0.1434012	0.555771488
110960	5	0	12.75	0.001	-13.63820963	Down		0.0383984	0.313863543
67199	2	0	12.74	0.001	-13.63707766	Down		0.277122	0.61376954
22327	3	0	12.71	0.001	-13.63367641	Down		0.1434012	0.531984568
18207	2	0	12.71	0.001	-13.63367641	Down		0.277122	0.617715554
16372	5	0	12.7	0.001	-13.63254088	Down		0.0383984	0.303143346
11853	2	0	12.68	0.001	-13.63026713	Down		0.277122	0.691848833
66521	2	0	12.68	0.001	-13.63026713	Down		0.277122	0.617881072
241593	2	0	12.67	0.001	-13.6291289	Down		0.277122	0.655094364
15496	3	0	12.66	0.001	-13.62798978	Down		0.1434012	0.537254113
232946	3.46	0	12.65	0.001	-13.62684976	Down		0.1434012	0.577840865
72982	3	0	12.61	0.001	-13.62228066	Down		0.1434012	0.525194272
66576	1	0	12.6	0.001	-13.62113611	Down		0.53554	0.745188686
18781	3	0	12.58	0.001	-13.6188443	Down		0.1434012	0.534366944

217845	2	0	12.55	0.001	-13.61539974	Down	0.277122	0.69372207
215900	2	0	12.53	0.001	-13.61309879	Down	0.277122	0.708210123
26563	4	0	12.5	0.001	-13.60964047	Down	0.074205	0.445819354
24116	4	0	12.47	0.001	-13.60617384	Down	0.074205	0.409184761
110012	2	0	12.46	0.001	-13.60501645	Down	0.277122	0.70195804
21849	6	0	12.36	0.001	-13.59339112	Down	0.01986984	0.212788853
11848	4	0	12.31	0.001	-13.58754314	Down	0.074205	0.445497695
26942	6	0	12.3	0.001	-13.5863707	Down	0.01986984	0.216126717
13168	1	0	12.3	0.001	-13.5863707	Down	0.53554	0.699894509
71912	2	0	12.28	0.001	-13.58402294	Down	0.277122	0.679214186
69129	2	0	12.17	0.001	-13.57104155	Down	0.277122	0.712587195
22428	2	0	12.15	0.001	-13.56866869	Down	0.277122	0.656773615
66176	2	0	12.13	0.001	-13.56629193	Down	0.277122	0.655280523
12062	3	0	12.1	0.001	-13.56271943	Down	0.1434012	0.52938837
66053	4	0	12.09	0.001	-13.56152662	Down	0.074205	0.423497809
20650	8	0	12.09	0.001	-13.56152662	Down	0.00532054	0.09603517
68052	1	0	12.08	0.001	-13.56033283	Down	0.53554	0.745064093
100756	5	0	12.04	0.001	-13.55554777	Down	0.0383984	0.319193893
140577	8	0	12.03	0.001	-13.55434902	Down	0.00532054	0.09948812
13167	1	0	12.03	0.001	-13.55434902	Down	0.53554	0.776346401
320581	4	0	12.02	0.001	-13.55314928	Down	0.074205	0.441042718
26448	3	0	12.01	0.001	-13.55194853	Down	0.1434012	0.588964159
106722	4	0	11.97	0.001	-13.54713553	Down	0.074205	0.420899663
73822	3	0	11.94	0.001	-13.54351522	Down	0.1434012	0.55396536
213171	2	0	11.92	0.001	-13.54109662	Down	0.277122	0.618544035
20204	2	0	11.91	0.001	-13.53988579	Down	0.277122	0.689573015

102308570	2	0	11.91	0.001	-13.53988579	Down	0.277122	0.629004954
11984	2	0	11.9	0.001	-13.53867395	Down	0.277122	0.700040122
12974	5	0	11.87	0.001	-13.53503231	Down	0.0383984	0.301143343
16858	1	0	11.82	0.001	-13.52894242	Down	0.53554	0.764492767
14311	3	0	11.81	0.001	-13.52772134	Down	0.1434012	0.583777586
19981	1	0	11.73	0.001	-13.51791539	Down	0.53554	0.808019645
110172	2	0	11.71	0.001	-13.51545346	Down	0.277122	0.644655343
67429	6	0	11.67	0.001	-13.51051694	Down	0.01986984	0.214723297
234582	4	0	11.67	0.001	-13.51051694	Down	0.074205	0.407833425
212679	5	0	11.65	0.001	-13.50804233	Down	0.0383984	0.32111868
380713	5	0	11.64	0.001	-13.50680344	Down	0.0383984	0.314481384
240121	3	0	11.62	0.001	-13.50432245	Down	0.1434012	0.566053788
329581	2	0	11.62	0.001	-13.50432245	Down	0.277122	0.62221591
107753	1	0	11.61	0.001	-13.50308035	Down	0.53554	0.70970351
66399	2	0	11.6	0.001	-13.50183718	Down	0.277122	0.661483695
69029	1	0	11.59	0.001	-13.50059295	Down	0.53554	0.784547243
257926	2	0	11.59	0.001	-13.50059295	Down	0.277122	0.666454382
65105	2	0	11.58	0.001	-13.49934763	Down	0.277122	0.619208422
69596	2	0	11.56	0.001	-13.49685378	Down	0.277122	0.709081231
100169864	2	0	11.53	0.001	-13.49310489	Down	0.277122	0.707992681
55942	2	0	11.52	0.001	-13.4918531	Down	0.277122	0.710610836
16705	1	0	11.51	0.001	-13.49060021	Down	0.53554	0.769243629
67044	1	0	11.49	0.001	-13.48809118	Down	0.53554	0.766596996
69612	6	0	11.48	0.001	-13.48683502	Down	0.01986984	0.217835229
71566	5	0	11.46	0.001	-13.48431942	Down	0.0383984	0.317923469

69305	2	0	11.46	0.001	-13.48431942	Down	0.277122	0.641963297
56513	2	0	11.46	0.001	-13.48431942	Down	0.277122	0.634718459
22437	10	0	11.42	0.001	-13.47927503	Down	0.00142468	0.036816032
59001	1	0	11.41	0.001	-13.47801117	Down	0.53554	0.705657694
56698	3	0	11.39	0.001	-13.47548013	Down	0.1434012	0.587803638
68499	1	0	11.35	0.001	-13.47040468	Down	0.53554	0.802490247
329777	8	0	11.26	0.001	-13.45891921	Down	0.00532054	0.098601811
14977	4	0	11.23	0.001	-13.45507031	Down	0.074205	0.424371
433416	1	0	11.18	0.001	-13.44863257	Down	0.53554	0.704764881
13638	2	0	11.16	0.001	-13.44604941	Down	0.277122	0.678015925
217449	5	0	11.15	0.001	-13.44475609	Down	0.0383984	0.320153393
71932	4	0	11.14	0.001	-13.44346161	Down	0.074205	0.405956479
66821	3	0	11.13	0.001	-13.44216597	Down	0.1434012	0.591592159
20410	5	0	11.13	0.001	-13.44216597	Down	0.0383984	0.303719664
22218	2	0	11.11	0.001	-13.4395712	Down	0.277122	0.627293842
213436	5	0	11.11	0.001	-13.4395712	Down	0.0383984	0.302856006
106344	2	0	11.1	0.001	-13.43827206	Down	0.277122	0.707775372
22640	3	0	11.08	0.001	-13.43567026	Down	0.1434012	0.587225091
59030	3	0	11.08	0.001	-13.43567026	Down	0.1434012	0.549374487
18102	2	0	11.06	0.001	-13.43306377	Down	0.277122	0.698979134
59052	3	0	10.97	0.001	-13.42127591	Down	0.1434012	0.523351485
19668	4	0	10.96	0.001	-13.41996018	Down	0.074205	0.430285578
56695	1	0	10.94	0.001	-13.41732512	Down	0.53554	0.715285448
14064	4	0	10.92	0.001	-13.41468524	Down	0.074205	0.421762162
214766	3	0	10.92	0.001	-13.41468524	Down	0.1434012	0.548364607
224624	4	0	10.91	0.001	-13.41336348	Down	0.074205	0.42612823

50996	4	0	10.89	0.001	-13.41071633	Down	0.074205	0.437604398
80876	1	0	10.87	0.001	-13.40806432	Down	0.53554	0.711402992
28084	2	0	10.85	0.001	-13.40540742	Down	0.277122	0.68344166
18538	2	0	10.84	0.001	-13.40407714	Down	0.277122	0.696445836
22068	5.22	0	10.83	0.001	-13.40274562	Down	0.0383984	0.314791218
12739	2	0	10.81	0.001	-13.4000789	Down	0.277122	0.700252706
108811	2	0	10.8	0.001	-13.39874369	Down	0.277122	0.615407569
14272	3	0	10.78	0.001	-13.39606956	Down	0.1434012	0.563647324
210135	6	0	10.77	0.001	-13.39473063	Down	0.01986984	0.21697761
66825	2	0	10.72	0.001	-13.38801729	Down	0.277122	0.637526171
12412	2	0	10.71	0.001	-13.38667086	Down	0.277122	0.703886496
20892	1	0	10.71	0.001	-13.38667086	Down	0.53554	0.824769265
18130	8	0	10.64	0.001	-13.37721053	Down	0.00532054	0.099937276
13445	2	0	10.64	0.001	-13.37721053	Down	0.277122	0.685269588
433702	5	0	10.59	0.001	-13.37041497	Down	0.0383984	0.310810395
233571	3	0	10.58	0.001	-13.36905201	Down	0.1434012	0.54685673
17701	3	0	10.5	0.001	-13.35810171	Down	0.1434012	0.528684708
57275	1	0	10.49	0.001	-13.35672706	Down	0.53554	0.825380319
14077	1	0	10.48	0.001	-13.3553511	Down	0.53554	0.718398894
268706	14	0	10.45	0.001	-13.35121532	Down	0.00010215	0.004249967
237362	2	0	10.44	0.001	-13.34983409	Down	0.277122	0.668773829
68463	1	0	10.43	0.001	-13.34845154	Down	0.53554	0.757346761
21664	3	0	10.38	0.001	-13.34151882	Down	0.1434012	0.572572642
67675	1	0	10.38	0.001	-13.34151882	Down	0.53554	0.757733096
83554	3	0	10.38	0.001	-13.34151882	Down	0.1434012	0.555254251
74614	3	0	10.36	0.001	-13.33873638	Down	0.1434012	0.546606223

59022	1	0	10.33	0.001	-13.33455263	Down	0.53554	0.729691885
68523	1	0	10.3	0.001	-13.33035672	Down	0.53554	0.797892272
230075	1	0	10.27	0.001	-13.32614856	Down	0.53554	0.842546481
12518	2	0	10.27	0.001	-13.32614856	Down	0.277122	0.696235556
245525	5	0	10.24	0.001	-13.32192809	Down	0.0383984	0.323721465
381112	5	0	10.22	0.001	-13.31910758	Down	0.0383984	0.312024498
23954	3	0	10.19	0.001	-13.31486643	Down	0.1434012	0.584922248
20352	6	0	10.17	0.001	-13.31203206	Down	0.01986984	0.213063065
118445	4	0	10.14	0.001	-13.30777003	Down	0.074205	0.419185204
12854	1	0	10.14	0.001	-13.30777003	Down	0.53554	0.710608889
11651	4	0	10.12	0.001	-13.30492167	Down	0.074205	0.448735323
20319	3	0	10.12	0.001	-13.30492167	Down	0.1434012	0.548616729
216558	4	0	10.12	0.001	-13.30492167	Down	0.074205	0.413847054
18292	2	0	10.12	0.001	-13.30492167	Down	0.277122	0.613606217
56069	1	0	10.11	0.001	-13.30349538	Down	0.53554	0.802201321
71994	3	0	10.11	0.001	-13.30349538	Down	0.1434012	0.530801328
211577	3	0	10.1	0.001	-13.30206767	Down	0.1434012	0.592473379
268752	3	0	10.07	0.001	-13.29777606	Down	0.1434012	0.594243718
66194	2	0	10.07	0.001	-13.29777606	Down	0.277122	0.714574578
69540	2	0	10.06	0.001	-13.29634268	Down	0.277122	0.706907468
14173	1	0	10.06	0.001	-13.29634268	Down	0.53554	0.819913218
20363	3	0	10.06	0.001	-13.29634268	Down	0.1434012	0.552426567
19242	3	0	10.02	0.001	-13.29059489	Down	0.1434012	0.538709429
15403	1	0	10	0.001	-13.28771238	Down	0.53554	0.710269101
76737	2	0	9.99	0.001	-13.28626896	Down	0.277122	0.686698083
12804	3	0	9.98	0.001	-13.2848241	Down	0.1434012	0.561789729

320040	4	0	9.95	0.001	-13.28048081	Down	0.074205	0.424955131
76905	2	0	9.93	0.001	-13.277578	Down	0.277122	0.698344083
20665	4	0	9.91	0.001	-13.27466934	Down	0.074205	0.415238605
21813	7	0	9.89	0.001	-13.27175481	Down	0.01028194	0.149312431
54131	3	0	9.86	0.001	-13.26737193	Down	0.1434012	0.592179347
18646	3	0	9.86	0.001	-13.26737193	Down	0.1434012	0.576723724
382406	4	0	9.85	0.001	-13.26590801	Down	0.074205	0.446464067
108160	2	0	9.85	0.001	-13.26590801	Down	0.277122	0.665492687
69363	1	0	9.84	0.001	-13.2644426	Down	0.53554	0.844302452
11669	3	0	9.83	0.001	-13.2629757	Down	0.1434012	0.541643843
11364	3	0	9.82	0.001	-13.26150731	Down	0.1434012	0.588673599
20608	2	0	9.81	0.001	-13.26003742	Down	0.277122	0.659402963
230809	7	0	9.81	0.001	-13.26003742	Down	0.01028194	0.144520309
18036	3	0	9.8	0.001	-13.25856603	Down	0.1434012	0.586071407
381104	2	0	9.78	0.001	-13.25561875	Down	0.277122	0.643216782
16669	2	0	9.75	0.001	-13.2511865	Down	0.277122	0.646281436
52432	3	0	9.74	0.001	-13.24970606	Down	0.1434012	0.580088179
72281	4	0	9.71	0.001	-13.24525558	Down	0.074205	0.423207543
28018	7	0	9.68	0.001	-13.24079133	Down	0.01028194	0.147003475
69556	2	0	9.67	0.001	-13.23930017	Down	0.277122	0.615736225
15438	3	0	9.64	0.001	-13.23481743	Down	0.1434012	0.550388093
13628	3	0	9.59	0.001	-13.2273151	Down	0.1434012	0.593947927
14375	3	0	9.58	0.001	-13.22580994	Down	0.1434012	0.588093339
68694	1	0	9.57	0.001	-13.22430321	Down	0.53554	0.810665516
22319	3	0	9.57	0.001	-13.22430321	Down	0.1434012	0.545606486
19262	5	0	9.57	0.001	-13.22430321	Down	0.0383984	0.301712074

16149	2	0	9.51	0.001	-13.21522963	Down	0.277122	0.711268403
214952	4	0	9.51	0.001	-13.21522963	Down	0.074205	0.42147427
73835	1	0	9.5	0.001	-13.2137118	Down	0.53554	0.750459471
218236	7	0	9.5	0.001	-13.2137118	Down	0.01028194	0.144764844
18261	1	0	9.49	0.001	-13.21219237	Down	0.53554	0.711857562
16578	4	0	9.47	0.001	-13.20914871	Down	0.074205	0.439786186
72536	4.34	0	9.47	0.001	-13.20914871	Down	0.074205	0.434524845
20005	1	0	9.46	0.001	-13.20762447	Down	0.53554	0.828449217
15519	4	0	9.42	0.001	-13.20151134	Down	0.074205	0.407026898
54351	2	0	9.4	0.001	-13.19844504	Down	0.277122	0.691226667
77721	2	0	9.39	0.001	-13.19690944	Down	0.277122	0.688337959
14870	1	0	9.37	0.001	-13.19383333	Down	0.53554	0.844622506
52377	2	0	9.36	0.001	-13.19229281	Down	0.277122	0.697499142
621823	1.45	0	9.35	0.001	-13.19075065	Down	0.53554	0.795045199
230234	6	0	9.33	0.001	-13.18766137	Down	0.01986984	0.218410751
110312	1	0	9.32	0.001	-13.18611424	Down	0.53554	0.747062588
20867	3	0	9.31	0.001	-13.18456545	Down	0.1434012	0.561261235
66349	2	0	9.29	0.001	-13.18146288	Down	0.277122	0.615079264
68949	1	0	9.24	0.001	-13.17367714	Down	0.53554	0.820668203
436022	3	0	9.24	0.001	-13.17367714	Down	0.1434012	0.544610399
66904	3	0	9.21	0.001	-13.16898544	Down	0.1434012	0.581218405
234865	8	0	9.19	0.001	-13.16584915	Down	0.00532054	0.098382696
75406	1	0	9.19	0.001	-13.16584915	Down	0.53554	0.737175904
11459	2	0	9.18	0.001	-13.16427844	Down	0.277122	0.663768613
66917	3	0	9.17	0.001	-13.16270602	Down	0.1434012	0.568751852
268807	3	0	9.14	0.001	-13.15797845	Down	0.1434012	0.590421269

216441	3	0	9.13	0.001	-13.15639914	Down	0.1434012	0.541889821
76813	3	0	9.13	0.001	-13.15639914	Down	0.1434012	0.539928229
77914	1	0	9.12	0.001	-13.15481811	Down	0.53554	0.778380496
76491	2	0	9.12	0.001	-13.15481811	Down	0.277122	0.673658242
230935	4	0	9.12	0.001	-13.15481811	Down	0.074205	0.431488333
55925	2	0	9.04	0.001	-13.14210706	Down	0.277122	0.704316482
70834	10	0	9.04	0.001	-13.14210706	Down	0.00142468	0.037162264
17841	1	0	9.03	0.001	-13.14051027	Down	0.53554	0.833095595
232947	4	0	9.03	0.001	-13.14051027	Down	0.074205	0.426422517
64450	4	0	9.03	0.001	-13.14051027	Down	0.074205	0.41804997
71893	3	0	9.03	0.001	-13.14051027	Down	0.1434012	0.527749396
230596	2	0	8.94	0.001	-13.12605912	Down	0.277122	0.649741381
268860	6	0	8.94	0.001	-13.12605912	Down	0.01986984	0.213613616
20509	3	0	8.94	0.001	-13.12605912	Down	0.1434012	0.530093907
193838	2	0	8.93	0.001	-13.12444446	Down	0.277122	0.67622644
216618	2	0	8.92	0.001	-13.12282799	Down	0.277122	0.659969136
13036	2	0	8.91	0.001	-13.12120972	Down	0.277122	0.670524037
353169	5	0	8.9	0.001	-13.11958962	Down	0.0383984	0.308708296
17242	1	0	8.86	0.001	-13.11309098	Down	0.53554	0.772042332
13609	4	0	8.86	0.001	-13.11309098	Down	0.074205	0.429985937
16493	4	0	8.85	0.001	-13.11146174	Down	0.074205	0.442306451
13732	1	0	8.83	0.001	-13.10819772	Down	0.53554	0.728379918
666747	2.96	0	8.82	0.001	-13.10656294	Down	0.277122	0.681422034
26384	3	0	8.79	0.001	-13.10164745	Down	0.1434012	0.527282981
58522	2	0	8.77	0.001	-13.09836113	Down	0.277122	0.618378161
378702	4	0	8.73	0.001	-13.09176594	Down	0.074205	0.444215687

66496	1	0	8.72	0.001	-13.09011242	Down	0.53554	0.84318417
110954	1	0	8.71	0.001	-13.088457	Down	0.53554	0.770440584
100039139	1	0	8.71	0.001	-13.088457	Down	0.53554	0.721890222
23997	2	0	8.69	0.001	-13.08514046	Down	0.277122	0.645196464
56323	3	0	8.68	0.001	-13.08347933	Down	0.1434012	0.591006134
71281	2	0	8.67	0.001	-13.08181628	Down	0.277122	0.609229105
14810	5	0	8.67	0.001	-13.08181628	Down	0.0383984	0.30142744
27260	2	0	8.65	0.001	-13.07848442	Down	0.277122	0.625592014
380686	2	0	8.65	0.001	-13.07848442	Down	0.277122	0.608425373
227154	3	0	8.64	0.001	-13.0768156	Down	0.1434012	0.544361946
83766	2	0	8.61	0.001	-13.07179752	Down	0.277122	0.711927188
67586	2	0	8.61	0.001	-13.07179752	Down	0.277122	0.674841136
432628	4	0	8.58	0.001	-13.06676193	Down	0.074205	0.438848475
17423	5	0	8.56	0.001	-13.06339508	Down	0.0383984	0.315724394
13010	1	0	8.54	0.001	-13.06002035	Down	0.53554	0.778108668
66443	3	0	8.54	0.001	-13.06002035	Down	0.1434012	0.527050082
217364	5	0	8.53	0.001	-13.05833003	Down	0.0383984	0.306047018
192986	3.03	0	8.52	0.001	-13.05663772	Down	0.1434012	0.569023074
18035	2	0	8.52	0.001	-13.05663772	Down	0.277122	0.656586606
66645	3	0	8.5	0.001	-13.05324713	Down	0.1434012	0.522892807
16815	1	0	8.49	0.001	-13.05154884	Down	0.53554	0.765543436
216456	3	0	8.49	0.001	-13.05154884	Down	0.1434012	0.554222659
235135	2	0	8.47	0.001	-13.04814625	Down	0.277122	0.692056471
102644	3	0	8.46	0.001	-13.04644195	Down	0.1434012	0.572298026
353310	4	0	8.42	0.001	-13.03960452	Down	0.074205	0.418616817

100037282	2	0	8.4	0.001	-13.03617361	Down	0.277122	0.671500338
17901	1	0	8.4	0.001	-13.03617361	Down	0.53554	0.690352957
69480	6	0	8.36	0.001	-13.02928723	Down	0.01986984	0.20955252
211770	5	0	8.31	0.001	-13.02063276	Down	0.0383984	0.308410315
53418	3	0	8.3	0.001	-13.01889562	Down	0.1434012	0.551915534
13865	3	0	8.3	0.001	-13.01889562	Down	0.1434012	0.531273992
18972	2	0	8.3	0.001	-13.01889562	Down	0.277122	0.611815379
103236	3	0	8.29	0.001	-13.01715639	Down	0.1434012	0.580370324
68961	2	0	8.25	0.001	-13.0101784	Down	0.277122	0.700678263
56720	2	0	8.24	0.001	-13.00842862	Down	0.277122	0.61327983
12539	3	0	8.22	0.001	-13.00492268	Down	0.1434012	0.577002604
94219	4	0	8.21	0.001	-13.00316651	Down	0.074205	0.443896337
77974	2	0	8.18	0.001	-12.99788513	Down	0.277122	0.663386698
228543	2	0	8.18	0.001	-12.99788513	Down	0.277122	0.662053449
69860	3	0	8.17	0.001	-12.99612036	Down	0.1434012	0.529858519
13056	3	0	8.16	0.001	-12.99435344	Down	0.1434012	0.553708299
442827	5	0	8.15	0.001	-12.99258434	Down	0.0383984	0.31634959
69352	6	0	8.12	0.001	-12.98726401	Down	0.01986984	0.213337985
235036	2	0	8.11	0.001	-12.9854862	Down	0.277122	0.620708523
107260	2	0	8.08	0.001	-12.98013958	Down	0.277122	0.70046542
12054	2	0	8.08	0.001	-12.98013958	Down	0.277122	0.64992451
68695	1	0	8.05	0.001	-12.97477307	Down	0.53554	0.787737023
20556	2	0	8.05	0.001	-12.97477307	Down	0.277122	0.636470373
230899	1	0	8.05	0.001	-12.97477307	Down	0.53554	0.700775018
11871	1	0	8.01	0.001	-12.96758653	Down	0.53554	0.734018834
17067	1	0	8	0.001	-12.96578428	Down	0.53554	0.696612215

30923	1	0	7.98	0.001	-12.96217303	Down	0.53554	0.821576021
18190	8	0	7.98	0.001	-12.96217303	Down	0.00532054	0.097515888
74316	1	0	7.97	0.001	-12.96036401	Down	0.53554	0.831541023
66272	1	0	7.96	0.001	-12.95855272	Down	0.53554	0.759411783
69790	1	0	7.95	0.001	-12.95673915	Down	0.53554	0.774592098
231050	3	0	7.94	0.001	-12.95492329	Down	0.1434012	0.59513286
16194	4	0	7.94	0.001	-12.95492329	Down	0.074205	0.422339128
229658	5	0	7.93	0.001	-12.95310515	Down	0.0383984	0.312941319
20811	3	0	7.92	0.001	-12.95128471	Down	0.1434012	0.58150165
20616	4	0	7.92	0.001	-12.95128471	Down	0.074205	0.437914755
17134	2	0	7.88	0.001	-12.94397991	Down	0.277122	0.60794415
213272	2	0	7.84	0.001	-12.93663794	Down	0.277122	0.619707649
54397	2	0	7.83	0.001	-12.93479659	Down	0.277122	0.607303703
56742	2	0	7.79	0.001	-12.92740761	Down	0.277122	0.651393266
215748	4	0	7.77	0.001	-12.92369888	Down	0.074205	0.415518038
73710	2.21	0	7.76	0.001	-12.92184094	Down	0.277122	0.678215342
27762	5	0	7.75	0.001	-12.9199806	Down	0.0383984	0.304588262
12070	1	0	7.74	0.001	-12.91811785	Down	0.53554	0.729333607
21838	2	0	7.72	0.001	-12.91438513	Down	0.277122	0.61393295
69723	1	0	7.71	0.001	-12.91251514	Down	0.53554	0.828141301
76898	4	0	7.71	0.001	-12.91251514	Down	0.074205	0.420040684
387511	1	0	7.71	0.001	-12.91251514	Down	0.53554	0.731969175
228094	2	0	7.7	0.001	-12.91064273	Down	0.277122	0.680015382
76130	3	0	7.69	0.001	-12.90876788	Down	0.1434012	0.543865718
212090	1	0	7.69	0.001	-12.90876788	Down	0.53554	0.713795986
66153	1	0	7.68	0.001	-12.9068906	Down	0.53554	0.824311569

22278	2	0	7.66	0.001	-12.90312868	Down	0.277122	0.62137757
74729	2	0	7.65	0.001	-12.90124403	Down	0.277122	0.610842957
102954	2	0	7.64	0.001	-12.89935692	Down	0.277122	0.661293995
56738	3	0	7.64	0.001	-12.89935692	Down	0.1434012	0.537012325
57436	2	0	7.64	0.001	-12.89935692	Down	0.277122	0.607623758
107526	1	0	7.63	0.001	-12.89746734	Down	0.53554	0.806119454
381337	1	0	7.6	0.001	-12.8917837	Down	0.53554	0.838424899
66455	2	0	7.6	0.001	-12.8917837	Down	0.277122	0.656212909
13603	2	0	7.59	0.001	-12.88988417	Down	0.277122	0.71413198
114602	2	0	7.59	0.001	-12.88988417	Down	0.277122	0.691433932
23991	1	0	7.58	0.001	-12.88798213	Down	0.53554	0.841591754
71846	1	0	7.58	0.001	-12.88798213	Down	0.53554	0.700224441
56709	2	0	7.57	0.001	-12.88607758	Down	0.277122	0.633671932
13163	3	0	7.57	0.001	-12.88607758	Down	0.1434012	0.540662159
19084	4	0	7.57	0.001	-12.88607758	Down	0.074205	0.404626347
227615	1	0	7.56	0.001	-12.88417052	Down	0.53554	0.84238721
433182	2	0	7.56	0.001	-12.88417052	Down	0.277122	0.643755489
66371	2	0	7.56	0.001	-12.88417052	Down	0.277122	0.631589198
381853	2	0	7.55	0.001	-12.88226093	Down	0.277122	0.702171791
70325	3	0	7.53	0.001	-12.87843415	Down	0.1434012	0.585209115
12512	1	0	7.52	0.001	-12.87651695	Down	0.53554	0.794195035
78925	3	0	7.52	0.001	-12.87651695	Down	0.1434012	0.56205435
14726	2	0	7.52	0.001	-12.87651695	Down	0.277122	0.62831939
107702	2	0	7.49	0.001	-12.87075	Down	0.277122	0.675830059
23897	1	0	7.48	0.001	-12.86882255	Down	0.53554	0.825074679
74443	2	0	7.48	0.001	-12.86882255	Down	0.277122	0.684862537

50868	5	0	7.47	0.001	-12.86689253	Down	0.0383984	0.32241482
244058	4	0	7.47	0.001	-12.86689253	Down	0.074205	0.431187015
75416	3	0	7.46	0.001	-12.86495992	Down	0.1434012	0.56258434
71983	2	0	7.45	0.001	-12.86302471	Down	0.277122	0.655466789
20333	2	0	7.45	0.001	-12.86302471	Down	0.277122	0.624913865
66235	1	0	7.45	0.001	-12.86302471	Down	0.53554	0.712312714
103080	2	0	7.43	0.001	-12.8591465	Down	0.277122	0.69961534
71667	4	0	7.43	0.001	-12.8591465	Down	0.074205	0.434830849
66401	1	0	7.43	0.001	-12.8591465	Down	0.53554	0.702431958
69928	1	0	7.42	0.001	-12.85720347	Down	0.53554	0.773382218
69511	1	0	7.42	0.001	-12.85720347	Down	0.53554	0.716090043
66043	1	0	7.41	0.001	-12.85525783	Down	0.53554	0.74681219
13179	2	0	7.4	0.001	-12.85330956	Down	0.277122	0.65696073
665150	1	0	7.4	0.001	-12.85330956	Down	0.53554	0.69901621
22088	2	0	7.38	0.001	-12.8494051	Down	0.277122	0.610034963
18802	3	0	7.37	0.001	-12.8474489	Down	0.1434012	0.564447202
17427	2	0	7.36	0.001	-12.84549005	Down	0.277122	0.706690825
71564	1	0	7.35	0.001	-12.84352853	Down	0.53554	0.816308544
67938	1	0	7.35	0.001	-12.84352853	Down	0.53554	0.776211172
69871	1	0	7.35	0.001	-12.84352853	Down	0.53554	0.719907648
67732	1	0	7.33	0.001	-12.83959748	Down	0.53554	0.766728895
238317	8	0	7.33	0.001	-12.83959748	Down	0.00532054	0.096664221
74254	2	0	7.32	0.001	-12.83762793	Down	0.277122	0.624406218
223922	3	0	7.31	0.001	-12.83565569	Down	0.1434012	0.578681564
78977	2	0	7.31	0.001	-12.83565569	Down	0.277122	0.677219431
18648	2	0	7.29	0.001	-12.8317031	Down	0.277122	0.653794205

109346	2	0	7.29	0.001	-12.8317031	Down	0.277122	0.640714688
20130	1	0	7.29	0.001	-12.8317031	Down	0.53554	0.70221058
27681	1	0	7.28	0.001	-12.82972274	Down	0.53554	0.716896451
100038860	1	0	7.27	0.001	-12.82773965	Down	0.53554	0.740852592
67707	1	0	7.27	0.001	-12.82773965	Down	0.53554	0.740360249
217371	2	0	7.25	0.001	-12.82376528	Down	0.277122	0.630897992
667281	1	0	7.24	0.001	-12.82177398	Down	0.53554	0.767521244
214359	2	0	7.22	0.001	-12.81778312	Down	0.277122	0.703457035
234384	1	0	7.22	0.001	-12.81778312	Down	0.53554	0.749701941
73804	3	0	7.22	0.001	-12.81778312	Down	0.1434012	0.526352618
66090	1	0	7.2	0.001	-12.81378119	Down	0.53554	0.774861475
100504162	1	0	7.19	0.001	-12.81177606	Down	0.53554	0.801335792
22668	4	0	7.19	0.001	-12.81177606	Down	0.074205	0.433609414
11409	2	0	7.19	0.001	-12.81177606	Down	0.277122	0.614587463
17221	2	0	7.16	0.001	-12.80574387	Down	0.277122	0.658649575
258745	1	0	7.16	0.001	-12.80574387	Down	0.53554	0.697593666
13619	4	0	7.15	0.001	-12.80372753	Down	0.074205	0.417767121
259122	1	0	7.13	0.001	-12.79968636	Down	0.53554	0.71632026
226861	3	0	7.11	0.001	-12.79563384	Down	0.1434012	0.540172651
68180	1	0	7.1	0.001	-12.79360331	Down	0.53554	0.783306089
68673	1	0	7.1	0.001	-12.79360331	Down	0.53554	0.759282389
100042150	3	0	7.1	0.001	-12.79360331	Down	0.1434012	0.530329505
100637	2	0	7.09	0.001	-12.79156991	Down	0.277122	0.63003611
503610	3	0	7.08	0.001	-12.78953364	Down	0.1434012	0.593357228
55944	2	0	7.08	0.001	-12.78953364	Down	0.277122	0.68222845

228413	3	0	7.08	0.001	-12.78953364	Down	0.1434012	0.566860516
140703	2	0	7.07	0.001	-12.7874945	Down	0.277122	0.651025455
108946	8	0	7.06	0.001	-12.78545247	Down	0.00532054	0.097947375
56462	2	0	7.05	0.001	-12.78340754	Down	0.277122	0.703242501
223227	4	0	7.05	0.001	-12.78340754	Down	0.074205	0.431790073
22689	4	0	7.04	0.001	-12.78135971	Down	0.074205	0.406758765
11785	2	0	7.03	0.001	-12.77930897	Down	0.277122	0.622720001
258334	1	0	7.02	0.001	-12.77725532	Down	0.53554	0.751852259
19288	2	0	7.02	0.001	-12.77725532	Down	0.277122	0.644835616
217232	6	0	7.02	0.001	-12.77725532	Down	0.01986984	0.20823292
14527	2	0	7.01	0.001	-12.77519873	Down	0.277122	0.67424917
19152	1	0	7.01	0.001	-12.77519873	Down	0.53554	0.732089427
51812	2	0	6.99	0.001	-12.77107674	Down	0.277122	0.6770206
74469	2	0	6.98	0.001	-12.76901132	Down	0.277122	0.709517588
258645	1	0	6.95	0.001	-12.76279726	Down	0.53554	0.837794386
18010	4	0	6.95	0.001	-12.76279726	Down	0.074205	0.404891675
244958	2	0	6.94	0.001	-12.76071995	Down	0.277122	0.678414876
74769	5	0	6.88	0.001	-12.74819285	Down	0.0383984	0.309006853
171530	1	0	6.87	0.001	-12.74609438	Down	0.53554	0.824464078
20256	2	0	6.86	0.001	-12.74399286	Down	0.277122	0.654722363
77318	1	0	6.85	0.001	-12.74188827	Down	0.53554	0.695959447
16175	2	0	6.84	0.001	-12.73978061	Down	0.277122	0.682026667
13033	2	0	6.83	0.001	-12.73766986	Down	0.277122	0.685066002
56196	2	0	6.83	0.001	-12.73766986	Down	0.277122	0.607143803
79456	4	0	6.82	0.001	-12.73555602	Down	0.074205	0.444855767
80751	2	0	6.82	0.001	-12.73555602	Down	0.277122	0.62952011

16403	6	0	6.8	0.001	-12.73131903	Down	0.01986984	0.215844567
16948	5	0	6.79	0.001	-12.72919586	Down	0.0383984	0.31417216
52466	2	0	6.79	0.001	-12.72919586	Down	0.277122	0.66243383
22381	1	0	6.76	0.001	-12.72280753	Down	0.53554	0.829374342
170706	1	0	6.76	0.001	-12.72280753	Down	0.53554	0.751345193
14712	3	0	6.75	0.001	-12.72067179	Down	0.1434012	0.580935436
109594	1	0	6.73	0.001	-12.71639079	Down	0.53554	0.728618107
18114	2	0	6.72	0.001	-12.71424552	Down	0.277122	0.612140208
93687	2	0	6.71	0.001	-12.71209705	Down	0.277122	0.621042866
93897	3	0	6.7	0.001	-12.70994538	Down	0.1434012	0.539196288
258218	1	0	6.69	0.001	-12.7077905	Down	0.53554	0.739868561
269954	3	0	6.69	0.001	-12.7077905	Down	0.1434012	0.524501708
65107	3	0	6.68	0.001	-12.70563239	Down	0.1434012	0.576445114
66708	1	0	6.67	0.001	-12.70347105	Down	0.53554	0.746687054
84094	2	0	6.64	0.001	-12.69696753	Down	0.277122	0.673461496
76539	2	0	6.63	0.001	-12.69479315	Down	0.277122	0.696025404
216292	2	0	6.62	0.001	-12.6926155	Down	0.277122	0.626612001
12321	3	0	6.61	0.001	-12.69043456	Down	0.1434012	0.551660372
11843	2	0	6.61	0.001	-12.69043456	Down	0.277122	0.624237185
71836	2	0	6.6	0.001	-12.68825031	Down	0.277122	0.661104404
69664	1	0	6.59	0.001	-12.68606275	Down	0.53554	0.793770634
19763	2	0	6.59	0.001	-12.68606275	Down	0.277122	0.662624184
110958	2	0	6.58	0.001	-12.68387187	Down	0.277122	0.635593209
13346	3	0	6.57	0.001	-12.68167766	Down	0.1434012	0.533888763
13204	3	0	6.57	0.001	-12.68167766	Down	0.1434012	0.530565311
269132	4	0	6.56	0.001	-12.6794801	Down	0.074205	0.417484655

66772	1	0	6.55	0.001	-12.67727919	Down	0.53554	0.835125251
56177	1	0	6.55	0.001	-12.67727919	Down	0.53554	0.784685392
632687	3	0	6.55	0.001	-12.67727919	Down	0.1434012	0.535566151
224093	3	0	6.54	0.001	-12.67507492	Down	0.1434012	0.557589432
17022	2	0	6.5	0.001	-12.666224	Down	0.277122	0.71435321
65970	4	0	6.5	0.001	-12.666224	Down	0.074205	0.406223556
56017	2	0	6.48	0.001	-12.6617781	Down	0.277122	0.642142067
102545	1	0	6.48	0.001	-12.6617781	Down	0.53554	0.720489627
234366	5	0	6.47	0.001	-12.65955	Down	0.0383984	0.320796271
56068	3	0	6.47	0.001	-12.65955	Down	0.1434012	0.552938547
70031	1	0	6.45	0.001	-12.65508345	Down	0.53554	0.796466191
80884	2	0	6.42	0.001	-12.64835758	Down	0.277122	0.606983986
15481	2	0	6.41	0.001	-12.64610864	Down	0.277122	0.688132546
11737	2	0	6.41	0.001	-12.64610864	Down	0.277122	0.633846114
93737	3	0	6.4	0.001	-12.64385619	Down	0.1434012	0.56311533
66973	1	0	6.4	0.001	-12.64385619	Down	0.53554	0.708124637
56716	3	0	6.39	0.001	-12.64160022	Down	0.1434012	0.537496119
228368	3	0	6.38	0.001	-12.63934071	Down	0.1434012	0.582353043
104444	1	0	6.38	0.001	-12.63934071	Down	0.53554	0.765806554
244666	2	0	6.38	0.001	-12.63934071	Down	0.277122	0.632108597
69903	3	0	6.35	0.001	-12.63254088	Down	0.1434012	0.580652742
112406	2	0	6.35	0.001	-12.63254088	Down	0.277122	0.667998888
210044	4	0	6.35	0.001	-12.63254088	Down	0.074205	0.429387903
20167	1	0	6.35	0.001	-12.63254088	Down	0.53554	0.741839244
56224	3	0	6.35	0.001	-12.63254088	Down	0.1434012	0.539440048
57267	2	0	6.35	0.001	-12.63254088	Down	0.277122	0.616889289

12259	1	0	6.34	0.001	-12.63026713	Down	0.53554	0.74656196
380660	2	0	6.33	0.001	-12.62798978	Down	0.277122	0.657897906
212898	4	0	6.3	0.001	-12.62113611	Down	0.074205	0.429686712
56644	2	0	6.29	0.001	-12.6188443	Down	0.277122	0.614260032
27426	2	0	6.27	0.001	-12.61424973	Down	0.277122	0.700891235
19934	2	0	6.26	0.001	-12.61194694	Down	0.277122	0.688543494
71268	3	0	6.26	0.001	-12.61194694	Down	0.1434012	0.549121668
234988	2	0	6.26	0.001	-12.61194694	Down	0.277122	0.624744558
72748	1	0	6.25	0.001	-12.60964047	Down	0.53554	0.717127187
269328	3	0	6.24	0.001	-12.60733031	Down	0.1434012	0.535325879
66156	3	0	6.23	0.001	-12.60501645	Down	0.1434012	0.567129936
54197	1	0	6.23	0.001	-12.60501645	Down	0.53554	0.713338937
14228	2	0	6.22	0.001	-12.60269887	Down	0.277122	0.61954115
66260	1	0	6.22	0.001	-12.60269887	Down	0.53554	0.700664833
74318	1	0	6.21	0.001	-12.60037755	Down	0.53554	0.76187867
105428	2	0	6.21	0.001	-12.60037755	Down	0.277122	0.608907357
77305	4	0	6.2	0.001	-12.5980525	Down	0.074205	0.447110648
75847	3	0	6.2	0.001	-12.5980525	Down	0.1434012	0.571475759
69161	1	0	6.19	0.001	-12.59572369	Down	0.53554	0.720955887
20680	3	0	6.16	0.001	-12.58871464	Down	0.1434012	0.579524714
237400	3	0	6.15	0.001	-12.5863707	Down	0.1434012	0.540907246
110805	1	0	6.14	0.001	-12.58402294	Down	0.53554	0.711971296
665155	3	0	6.12	0.001	-12.57931594	Down	0.1434012	0.570655851
101869	3	0	6.12	0.001	-12.57931594	Down	0.1434012	0.527982914
73739	1	0	6.12	0.001	-12.57931594	Down	0.53554	0.699125877
17237	3	0	6.11	0.001	-12.57695666	Down	0.1434012	0.589255005

16994	1	0	6.11	0.001	-12.57695666	Down	0.53554	0.760318775
24070	1	0	6.1	0.001	-12.57459353	Down	0.53554	0.812883681
71393	2	0	6.08	0.001	-12.56985561	Down	0.277122	0.640181056
219140	5	0	6.07	0.001	-12.5674808	Down	0.0383984	0.303431231
69189	1	0	6.05	0.001	-12.56271943	Down	0.53554	0.816607722
57315	2	0	6.03	0.001	-12.55794229	Down	0.277122	0.655839637
277353	2	0	6.02	0.001	-12.55554777	Down	0.277122	0.645738494
14761	1	0	6.01	0.001	-12.55314928	Down	0.53554	0.820215045
74116	2	0	6	0.001	-12.55074679	Down	0.277122	0.647370062
55934	1	0	6	0.001	-12.55074679	Down	0.53554	0.690566921
60322	2	0	5.99	0.001	-12.54834029	Down	0.277122	0.622048061
93675	2	0	5.99	0.001	-12.54834029	Down	0.277122	0.618212376
71927	3	0	5.99	0.001	-12.54834029	Down	0.1434012	0.525656998
17128	3	0	5.98	0.001	-12.54592977	Down	0.1434012	0.559944338
56371	2	0	5.97	0.001	-12.54351522	Down	0.277122	0.702385672
69581	3	0	5.97	0.001	-12.54351522	Down	0.1434012	0.574225883
231642	1	0	5.96	0.001	-12.54109662	Down	0.53554	0.763314207
28015	2	0	5.95	0.001	-12.53867395	Down	0.277122	0.68101954
20980	3	0	5.94	0.001	-12.53624722	Down	0.1434012	0.560470355
66220	1	0	5.94	0.001	-12.53624722	Down	0.53554	0.728856451
67229	3.04	0	5.94	0.001	-12.53624722	Down	0.1434012	0.534606355
668166	5	0	5.93	0.001	-12.53381639	Down	0.0383984	0.319513086
232409	2	0	5.93	0.001	-12.53381639	Down	0.277122	0.652314614
278507	3	0	5.93	0.001	-12.53381639	Down	0.1434012	0.52612054
223664	2	0	5.92	0.001	-12.53138146	Down	0.277122	0.709299342
268301	4	0	5.92	0.001	-12.53138146	Down	0.074205	0.437294479

29861	2	0	5.91	0.001	-12.52894242	Down		0.277122	0.712367057
232970	2	0	5.9	0.001	-12.52649924	Down		0.277122	0.708863253
66114	1	0	5.9	0.001	-12.52649924	Down		0.53554	0.792782128
52323	2.93	0	5.9	0.001	-12.52649924	Down		0.277122	0.653053572
258219	1	0	5.9	0.001	-12.52649924	Down		0.53554	0.732330048
21974	5	0	5.9	0.001	-12.52649924	Down		0.0383984	0.306928998
21400	1	0	5.89	0.001	-12.52405192	Down		0.53554	0.812587225
11857	1	0	5.89	0.001	-12.52405192	Down		0.53554	0.691316838
26568	2	0	5.88	0.001	-12.52160044	Down		0.277122	0.695815378
69009	1	0	5.88	0.001	-12.52160044	Down		0.53554	0.769775149
18131	7	0	5.87	0.001	-12.51914479	Down		0.01028194	0.144276598
71934	2	0	5.86	0.001	-12.51668495	Down		0.277122	0.672871947
67866	1	0	5.85	0.001	-12.51422091	Down		0.53554	0.801624094
20344	3	0	5.85	0.001	-12.51422091	Down		0.1434012	0.564180324
18807	2	0	5.84	0.001	-12.51175265	Down		0.277122	0.654350784
14573	3	0	5.83	0.001	-12.50928017	Down		0.1434012	0.590713557
81840	5	0	5.83	0.001	-12.50928017	Down		0.0383984	0.316036683
71756	4	0	5.83	0.001	-12.50928017	Down		0.074205	0.414959546
66142	1	0	5.82	0.001	-12.50680344	Down		0.53554	0.798035161
76487	2	0	5.81	0.001	-12.50432245	Down		0.277122	0.699403143
29813	2	0	5.81	0.001	-12.50432245	Down		0.277122	0.666839839
72999	2	0	5.8	0.001	-12.50183718	Down		0.277122	0.713248426
11949	1	0	5.8	0.001	-12.50183718	Down		0.53554	0.811699151
171282	2	0	5.79	0.001	-12.49934763	Down		0.277122	0.686085142
81897	3	0	5.79	0.001	-12.49934763	Down		0.1434012	0.55785011
214616	2	0	5.79	0.001	-12.49934763	Down		0.277122	0.664533764

234577	2	0	5.79	0.001	-12.49934763	Down	0.277122	0.654165152
170835	3	0	5.79	0.001	-12.49934763	Down	0.1434012	0.547358434
76612	2	0	5.78	0.001	-12.49685378	Down	0.277122	0.609068189
50498	1	0	5.76	0.001	-12.4918531	Down	0.53554	0.805245454
214669	3	0	5.76	0.001	-12.4918531	Down	0.1434012	0.55628969
54646	3	0	5.76	0.001	-12.4918531	Down	0.1434012	0.552170933
16369	2	0	5.76	0.001	-12.4918531	Down	0.277122	0.640536712
67760	4	0	5.75	0.001	-12.48934624	Down	0.074205	0.438536793
436062	1	0	5.75	0.001	-12.48934624	Down	0.53554	0.724707813
66840	2	0	5.75	0.001	-12.48934624	Down	0.277122	0.625083264
104418	3	0	5.74	0.001	-12.48683502	Down	0.1434012	0.590129271
29869	5	0	5.74	0.001	-12.48683502	Down	0.0383984	0.30663444
66175	1	0	5.73	0.001	-12.48431942	Down	0.53554	0.799610325
78829	2	0	5.73	0.001	-12.48431942	Down	0.277122	0.626782322
18772	4	0	5.73	0.001	-12.48431942	Down	0.074205	0.406490984
74030	4	0	5.72	0.001	-12.48179943	Down	0.074205	0.435137283
17454	3	0	5.72	0.001	-12.48179943	Down	0.1434012	0.5508963
21888	4	0	5.71	0.001	-12.47927503	Down	0.074205	0.44167368
14727	1	0	5.71	0.001	-12.47927503	Down	0.53554	0.774322909
17181	3	0	5.71	0.001	-12.47927503	Down	0.1434012	0.557328998
71781	2	0	5.7	0.001	-12.4767462	Down	0.277122	0.650107742
238831	2	0	5.69	0.001	-12.47421294	Down	0.277122	0.683036778
68818	1	0	5.68	0.001	-12.47167521	Down	0.53554	0.76318348
230815	4	0	5.67	0.001	-12.46913302	Down	0.074205	0.436059184
226757	6	0	5.66	0.001	-12.46658634	Down	0.01986984	0.217548603
22003	1	0	5.65	0.001	-12.46403515	Down	0.53554	0.835281788

56375	2	0	5.65	0.001	-12.46403515	Down	0.277122	0.664342311
116701	2	0	5.65	0.001	-12.46403515	Down	0.277122	0.65733528
26395	2	0	5.64	0.001	-12.46147945	Down	0.277122	0.670914216
83431	2	0	5.64	0.001	-12.46147945	Down	0.277122	0.611328781
23837	1	0	5.63	0.001	-12.45891921	Down	0.53554	0.697812142
12314	1	0	5.62	0.001	-12.45635442	Down	0.53554	0.738519778
105298	2	0	5.6	0.001	-12.45121111	Down	0.277122	0.679614548
230484	3	0	5.6	0.001	-12.45121111	Down	0.1434012	0.542382448
68776	1	0	5.6	0.001	-12.45121111	Down	0.53554	0.708800436
67273	1	0	5.59	0.001	-12.44863257	Down	0.53554	0.752614143
19823	1	0	5.58	0.001	-12.44604941	Down	0.53554	0.784133088
14786	2	0	5.58	0.001	-12.44604941	Down	0.277122	0.651577327
268490	1	0	5.58	0.001	-12.44604941	Down	0.53554	0.708462375
21832	2	0	5.57	0.001	-12.44346161	Down	0.277122	0.627977168
245666	5	0	5.56	0.001	-12.44086917	Down	0.0383984	0.318875336
320655	2	0	5.55	0.001	-12.43827206	Down	0.277122	0.716128001
64655	1	0	5.55	0.001	-12.43827206	Down	0.53554	0.747313154
54138	2	0	5.55	0.001	-12.43827206	Down	0.277122	0.616229867
101540	3	0	5.54	0.001	-12.43567026	Down	0.1434012	0.553451477
214505	1	0	5.54	0.001	-12.43567026	Down	0.53554	0.724472174
67164	1	0	5.53	0.001	-12.43306377	Down	0.53554	0.795897185
66705	1	0	5.53	0.001	-12.43306377	Down	0.53554	0.778924723
214944	5	0	5.53	0.001	-12.43306377	Down	0.0383984	0.311416264
30806	3	0	5.53	0.001	-12.43306377	Down	0.1434012	0.531510639
14840	1	0	5.53	0.001	-12.43306377	Down	0.53554	0.703874323
12387	3	0	5.52	0.001	-12.43045255	Down	0.1434012	0.591299002

22229	2	0	5.5	0.001	-12.4252159	Down	0.277122	0.679414308
212892	3	0	5.5	0.001	-12.4252159	Down	0.1434012	0.563913698
19662	1	0	5.5	0.001	-12.4252159	Down	0.53554	0.724943605
108075	3	0	5.49	0.001	-12.42259043	Down	0.1434012	0.571749586
12332	1	0	5.48	0.001	-12.41996018	Down	0.53554	0.807726725
553127	1	0	5.48	0.001	-12.41996018	Down	0.53554	0.731128522
16678	2	0	5.47	0.001	-12.41732512	Down	0.277122	0.616065232
230676	9	0	5.46	0.001	-12.41468524	Down	0.0027532	0.060929195
17171	2	0	5.44	0.001	-12.40939094	Down	0.277122	0.67013431
110196	1	0	5.41	0.001	-12.40141288	Down	0.53554	0.744441754
56692	1	0	5.41	0.001	-12.40141288	Down	0.53554	0.739009675
110911	1	0	5.41	0.001	-12.40141288	Down	0.53554	0.711175924
64685	1	0	5.41	0.001	-12.40141288	Down	0.53554	0.69509099
17420	2	0	5.37	0.001	-12.39070637	Down	0.277122	0.640358834
381410	2.99	0	5.35	0.001	-12.38532318	Down	0.277122	0.664725328
68592	1	0	5.35	0.001	-12.38532318	Down	0.53554	0.744317411
18551	2	0	5.35	0.001	-12.38532318	Down	0.277122	0.642320936
227059	4	0	5.34	0.001	-12.38262403	Down	0.074205	0.447434641
80291	1	0	5.34	0.001	-12.38262403	Down	0.53554	0.74419311
19217	3	0	5.33	0.001	-12.37991982	Down	0.1434012	0.569294554
104027	4	0	5.33	0.001	-12.37991982	Down	0.074205	0.408643154
21807	4	0	5.32	0.001	-12.37721053	Down	0.074205	0.422050448
13655	3	0	5.28	0.001	-12.36632221	Down	0.1434012	0.523810968
74117	2	0	5.27	0.001	-12.36358725	Down	0.277122	0.709735969
381546	1	0	5.27	0.001	-12.36358725	Down	0.53554	0.806849238
236790	3	0	5.27	0.001	-12.36358725	Down	0.1434012	0.56284971

66388	1	0	5.26	0.001	-12.36084708	Down	0.53554	0.788712981
58521	2	0	5.26	0.001	-12.36084708	Down	0.277122	0.668386134
67381	1	0	5.23	0.001	-12.35259523	Down	0.53554	0.775940857
16680	2	0	5.23	0.001	-12.35259523	Down	0.277122	0.627123242
14252	2	0	5.22	0.001	-12.34983409	Down	0.277122	0.632976163
103850	1	0	5.22	0.001	-12.34983409	Down	0.53554	0.697702887
107146	1	0	5.21	0.001	-12.34706766	Down	0.53554	0.739254867
381404	2	0	5.21	0.001	-12.34706766	Down	0.277122	0.612465382
66413	1	0	5.19	0.001	-12.34151882	Down	0.53554	0.827833613
66700	2	0	5.19	0.001	-12.34151882	Down	0.277122	0.662243585
234130	1	0	5.19	0.001	-12.34151882	Down	0.53554	0.727547484
239027	2	0	5.18	0.001	-12.33873638	Down	0.277122	0.658837761
66939	2	0	5.17	0.001	-12.33594857	Down	0.277122	0.64357582
16973	4	0	5.16	0.001	-12.33315535	Down	0.074205	0.443259013
11540	2	0	5.16	0.001	-12.33315535	Down	0.277122	0.658461497
93746	1	0	5.15	0.001	-12.33035672	Down	0.53554	0.82737251
242785	3	0	5.15	0.001	-12.33035672	Down	0.1434012	0.533649993
11945	1	0	5.14	0.001	-12.32755264	Down	0.53554	0.775535736
56420	1	0	5.12	0.001	-12.32192809	Down	0.53554	0.723061551
22625	3	0	5.1	0.001	-12.31628153	Down	0.1434012	0.567939736
14381	2	0	5.09	0.001	-12.31344994	Down	0.277122	0.685677122
237625	2	0	5.09	0.001	-12.31344994	Down	0.277122	0.665877032
209239	2	0	5.07	0.001	-12.30777003	Down	0.277122	0.711487862
266690	2	0	5.07	0.001	-12.30777003	Down	0.277122	0.643935259
66350	1	0	5.07	0.001	-12.30777003	Down	0.53554	0.707899657
217869	3	0	5.06	0.001	-12.30492167	Down	0.1434012	0.581785171

194597	2	0	5.06	0.001	-12.30492167	Down	0.277122	0.614423704
20280	2	0	5.05	0.001	-12.30206767	Down	0.277122	0.61197775
56046	2	0	5.04	0.001	-12.29920802	Down	0.277122	0.653238573
268469	2	0	5.04	0.001	-12.29920802	Down	0.277122	0.640892763
13353	1	0	5.03	0.001	-12.29634268	Down	0.53554	0.841273993
192786	6	0	5.03	0.001	-12.29634268	Down	0.01986984	0.211970434
20849	2	0	5.03	0.001	-12.29634268	Down	0.277122	0.63038058
231327	3	0	5.01	0.001	-12.29059489	Down	0.1434012	0.59365243
224481	1	0	5.01	0.001	-12.29059489	Down	0.53554	0.811255842
381199	3	0	5.01	0.001	-12.29059489	Down	0.1434012	0.578962341
22095	3	0	5.01	0.001	-12.29059489	Down	0.1434012	0.556030468
20740	6	0	5.01	0.001	-12.29059489	Down	0.01986984	0.209287264
12819	4	0	5	0.001	-12.28771238	Down	0.074205	0.43544415
97165	2	0	4.99	0.001	-12.2848241	Down	0.277122	0.680215977
12642	1	0	4.99	0.001	-12.2848241	Down	0.53554	0.786902409
13491	1	0	4.99	0.001	-12.2848241	Down	0.53554	0.78371937
13819	4	0	4.99	0.001	-12.2848241	Down	0.074205	0.408372887
15439	1	0	4.99	0.001	-12.2848241	Down	0.53554	0.697266209
21855	1.22	0	4.98	0.001	-12.28193003	Down	0.53554	0.829992241
52708	2	0	4.98	0.001	-12.28193003	Down	0.277122	0.641249211
103765	1	0	4.97	0.001	-12.27903014	Down	0.53554	0.822334073
29820	3	0	4.97	0.001	-12.27903014	Down	0.1434012	0.538223448
14299	3	0	4.97	0.001	-12.27903014	Down	0.1434012	0.523122045
84036	3	0	4.96	0.001	-12.27612441	Down	0.1434012	0.592767703
109828	2	0	4.96	0.001	-12.27612441	Down	0.277122	0.626952736
319638	2	0	4.95	0.001	-12.27321281	Down	0.277122	0.666647055

15387	2	0	4.94	0.001	-12.27029533	Down	0.277122	0.687722088
16854	1	0	4.94	0.001	-12.27029533	Down	0.53554	0.753504961
57438	2	0	4.94	0.001	-12.27029533	Down	0.277122	0.651761493
380785	2	0	4.93	0.001	-12.26737193	Down	0.277122	0.684659193
18230	2	0	4.93	0.001	-12.26737193	Down	0.277122	0.656399705
69035	5.16	0	4.92	0.001	-12.2644426	Down	0.0383984	0.322740491
15374	1	0	4.92	0.001	-12.2644426	Down	0.53554	0.761488096
27999	2	0	4.91	0.001	-12.26150731	Down	0.277122	0.681623459
52150	3	0	4.91	0.001	-12.26150731	Down	0.1434012	0.551150755
19366	2	0	4.9	0.001	-12.25856603	Down	0.277122	0.68731212
11966	2	0	4.9	0.001	-12.25856603	Down	0.277122	0.67169594
66849	3	0	4.9	0.001	-12.25856603	Down	0.1434012	0.552682439
16195	4	0	4.9	0.001	-12.25856603	Down	0.074205	0.42118677
80517	2	0	4.9	0.001	-12.25856603	Down	0.277122	0.609712364
19703	1	0	4.89	0.001	-12.25561875	Down	0.53554	0.825839203
67878	4	0	4.89	0.001	-12.25561875	Down	0.074205	0.43575145
16995	1	0	4.89	0.001	-12.25561875	Down	0.53554	0.758765255
107392	1	0	4.88	0.001	-12.25266543	Down	0.53554	0.754653402
14528	2	0	4.86	0.001	-12.2467406	Down	0.277122	0.705825578
50909	2	0	4.86	0.001	-12.2467406	Down	0.277122	0.610519503
52055	3	0	4.85	0.001	-12.24376903	Down	0.1434012	0.595429833
12313	3	0	4.85	0.001	-12.24376903	Down	0.1434012	0.577281754
74603	1	0	4.85	0.001	-12.24376903	Down	0.53554	0.762139275
20129	3	0	4.84	0.001	-12.24079133	Down	0.1434012	0.593062319
26415	1	0	4.84	0.001	-12.24079133	Down	0.53554	0.76686084
100061	2	0	4.84	0.001	-12.24079133	Down	0.277122	0.630552957

76551	4	0	4.84	0.001	-12.24079133	Down	0.074205	0.41191448
319446	1	0	4.84	0.001	-12.24079133	Down	0.53554	0.709025989
59091	3	0	4.83	0.001	-12.23780747	Down	0.1434012	0.539684028
619605	1	0	4.82	0.001	-12.23481743	Down	0.53554	0.798178101
68014	2	0	4.82	0.001	-12.23481743	Down	0.277122	0.634893216
74996	4	0	4.82	0.001	-12.23481743	Down	0.074205	0.409456104
17974	2	0	4.82	0.001	-12.23481743	Down	0.277122	0.608585949
102580	2	0	4.81	0.001	-12.23182118	Down	0.277122	0.697710185
12569	3	0	4.81	0.001	-12.23182118	Down	0.1434012	0.570928892
102032	1	0	4.8	0.001	-12.22881869	Down	0.53554	0.827526154
56332	3	0	4.79	0.001	-12.22580994	Down	0.1434012	0.584063331
170791	2	0	4.79	0.001	-12.22580994	Down	0.277122	0.672283429
17123	1	0	4.79	0.001	-12.22580994	Down	0.53554	0.751725428
12741	1	0	4.79	0.001	-12.22580994	Down	0.53554	0.751598641
19883	1	0	4.78	0.001	-12.2227949	Down	0.53554	0.818557741
108954	4	0	4.78	0.001	-12.2227949	Down	0.074205	0.411091748
235283	2	0	4.77	0.001	-12.21977355	Down	0.277122	0.662814648
233806	1	0	4.77	0.001	-12.21977355	Down	0.53554	0.759670702
68075	1	0	4.77	0.001	-12.21977355	Down	0.53554	0.702653475
56436	1	0	4.76	0.001	-12.21674586	Down	0.53554	0.809193452
11689	2	0	4.75	0.001	-12.2137118	Down	0.277122	0.692472121
71146	2	0	4.75	0.001	-12.2137118	Down	0.277122	0.683847023
231863	2	0	4.74	0.001	-12.21067134	Down	0.277122	0.702599684
71607	1	0	4.74	0.001	-12.21067134	Down	0.53554	0.697375327
76872	3	0	4.73	0.001	-12.20762447	Down	0.1434012	0.589837561
338467	3	0	4.73	0.001	-12.20762447	Down	0.1434012	0.587514222

66855	2	0	4.71	0.001	-12.20151134	Down	0.277122	0.625761781
14621	1	0	4.71	0.001	-12.20151134	Down	0.53554	0.699564889
14118	7	0	4.7	0.001	-12.19844504	Down	0.01028194	0.145256405
56095	2	0	4.69	0.001	-12.19537221	Down	0.277122	0.631762236
21402	1	0	4.68	0.001	-12.19229281	Down	0.53554	0.840639189
16906	2	0	4.68	0.001	-12.19229281	Down	0.277122	0.675236358
68337	1	0	4.68	0.001	-12.19229281	Down	0.53554	0.752741274
231044	1	0	4.68	0.001	-12.19229281	Down	0.53554	0.695199429
208258	1	0	4.67	0.001	-12.18920683	Down	0.53554	0.809781635
12223	2	0	4.67	0.001	-12.18920683	Down	0.277122	0.680818471
319934	5	0	4.65	0.001	-12.183015	Down	0.0383984	0.317292042
19309	2	0	4.65	0.001	-12.183015	Down	0.277122	0.648827282
93960	3	0	4.65	0.001	-12.183015	Down	0.1434012	0.535806639
11474	2	0	4.65	0.001	-12.183015	Down	0.277122	0.608264881
70584	2	0	4.63	0.001	-12.17679648	Down	0.277122	0.617550124
216169	1	0	4.62	0.001	-12.17367714	Down	0.53554	0.735715427
228359	2	0	4.62	0.001	-12.17367714	Down	0.277122	0.606664605
12909	1	0	4.61	0.001	-12.17055104	Down	0.53554	0.838740512
216869	1	0	4.61	0.001	-12.17055104	Down	0.53554	0.769908144
93742	4	0	4.61	0.001	-12.17055104	Down	0.074205	0.41163987
53857	1	0	4.6	0.001	-12.16741815	Down	0.53554	0.781520228
14121	1	0	4.6	0.001	-12.16741815	Down	0.53554	0.6961769
74112	2	0	4.59	0.001	-12.16427844	Down	0.277122	0.661863422
223453	1	0	4.59	0.001	-12.16427844	Down	0.53554	0.756960819
69780	2	0	4.59	0.001	-12.16427844	Down	0.277122	0.626441772
230459	3	0	4.58	0.001	-12.16113188	Down	0.1434012	0.567669546

67166	2	0	4.58	0.001	-12.16113188	Down	0.277122	0.632455338
15205	1	0	4.57	0.001	-12.15797845	Down	0.53554	0.831851473
107449	4	0	4.55	0.001	-12.15165083	Down	0.074205	0.427012313
27390	2	0	4.55	0.001	-12.15165083	Down	0.277122	0.63506807
68758	1	0	4.55	0.001	-12.15165083	Down	0.53554	0.726006572
228785	2	0	4.55	0.001	-12.15165083	Down	0.277122	0.616724301
20317	1	0	4.54	0.001	-12.14847658	Down	0.53554	0.835908524
52174	1	0	4.54	0.001	-12.14847658	Down	0.53554	0.776075991
14728	1	0	4.54	0.001	-12.14847658	Down	0.53554	0.748317102
66071	1	0	4.54	0.001	-12.14847658	Down	0.53554	0.748191461
24055	2	0	4.53	0.001	-12.14529533	Down	0.277122	0.712147054
70362	1	0	4.53	0.001	-12.14529533	Down	0.53554	0.836693267
76890	1	0	4.53	0.001	-12.14529533	Down	0.53554	0.781657313
14241	2	0	4.53	0.001	-12.14529533	Down	0.277122	0.67267566
619310	2	0	4.53	0.001	-12.14529533	Down	0.277122	0.619874237
52245	2	0	4.53	0.001	-12.14529533	Down	0.277122	0.617054365
16184	3	0	4.53	0.001	-12.14529533	Down	0.1434012	0.522206295
83672	1	0	4.52	0.001	-12.14210706	Down	0.53554	0.763968514
74492	2	0	4.52	0.001	-12.14210706	Down	0.277122	0.648644771
17130	2	0	4.52	0.001	-12.14210706	Down	0.277122	0.637349962
101489	2	0	4.52	0.001	-12.14210706	Down	0.277122	0.617384782
381510	2	0	4.5	0.001	-12.13570929	Down	0.277122	0.699827667
26918	2	0	4.5	0.001	-12.13570929	Down	0.277122	0.697921357
218138	1	0	4.5	0.001	-12.13570929	Down	0.53554	0.728260882
227632	4	0	4.49	0.001	-12.13249973	Down	0.074205	0.427900073
66237	1	0	4.49	0.001	-12.13249973	Down	0.53554	0.727785128

18518	1	0	4.47	0.001	-12.12605912	Down	0.53554	0.766333334
81018	2	0	4.47	0.001	-12.12605912	Down	0.277122	0.650291078
71829	1	0	4.47	0.001	-12.12605912	Down	0.53554	0.717820287
57296	1	0	4.44	0.001	-12.11634396	Down	0.53554	0.810813017
268783	3	0	4.44	0.001	-12.11634396	Down	0.1434012	0.570383071
192897	4	0	4.44	0.001	-12.11634396	Down	0.074205	0.433001266
232227	3	0	4.43	0.001	-12.11309098	Down	0.1434012	0.547861058
53620	1	0	4.42	0.001	-12.10983065	Down	0.53554	0.721072547
17246	2	0	4.42	0.001	-12.10983065	Down	0.277122	0.619042191
16341	1	0	4.41	0.001	-12.10656294	Down	0.53554	0.751218533
240725	3	0	4.41	0.001	-12.10656294	Down	0.1434012	0.536770754
58809	1	0	4.41	0.001	-12.10656294	Down	0.53554	0.701878775
66627	1	0	4.39	0.001	-12.10000522	Down	0.53554	0.835595038
108657	2	0	4.39	0.001	-12.10000522	Down	0.277122	0.707124245
18430	3	0	4.39	0.001	-12.10000522	Down	0.1434012	0.584635662
239217	4	0	4.39	0.001	-12.10000522	Down	0.074205	0.440099647
68797	1	0	4.39	0.001	-12.10000522	Down	0.53554	0.788015622
192287	2	0	4.38	0.001	-12.09671515	Down	0.277122	0.675632043
69672	1	0	4.37	0.001	-12.09341756	Down	0.53554	0.786208246
66193	1	0	4.37	0.001	-12.09341756	Down	0.53554	0.756061815
226519	5	0	4.37	0.001	-12.09341756	Down	0.0383984	0.305170092
233781	2	0	4.37	0.001	-12.09341756	Down	0.277122	0.610196391
100087	1	0	4.36	0.001	-12.09011242	Down	0.53554	0.747438501
54122	3	0	4.35	0.001	-12.08679969	Down	0.1434012	0.558372197
69597	2	0	4.35	0.001	-12.08679969	Down	0.277122	0.650841705
59029	1	0	4.35	0.001	-12.08679969	Down	0.53554	0.735593982

67089	1	0	4.34	0.001	-12.08347933	Down	0.53554	0.758636081
234814	1	0	4.33	0.001	-12.08015131	Down	0.53554	0.837951926
14200	1	0	4.33	0.001	-12.08015131	Down	0.53554	0.822941522
68775	1	0	4.33	0.001	-12.08015131	Down	0.53554	0.808166184
72306	2	0	4.31	0.001	-12.07347215	Down	0.277122	0.624575342
72433	1	0	4.3	0.001	-12.07012094	Down	0.53554	0.69748448
71774	2	0	4.29	0.001	-12.06676193	Down	0.277122	0.61804668
230936	2	0	4.29	0.001	-12.06676193	Down	0.277122	0.610681187
100764	1	0	4.28	0.001	-12.06339508	Down	0.53554	0.820970586
12286	5	0	4.28	0.001	-12.06339508	Down	0.0383984	0.316663118
72674	2	0	4.28	0.001	-12.06339508	Down	0.277122	0.673855103
230903	1	0	4.28	0.001	-12.06339508	Down	0.53554	0.734623861
14760	1	0	4.28	0.001	-12.06339508	Down	0.53554	0.726835482
109900	1	0	4.27	0.001	-12.06002035	Down	0.53554	0.836850392
74412	2	0	4.27	0.001	-12.06002035	Down	0.277122	0.621712635
54613	1	0	4.26	0.001	-12.05663772	Down	0.53554	0.829065738
66811	1	0	4.26	0.001	-12.05663772	Down	0.53554	0.780561979
15425	1	0	4.26	0.001	-12.05663772	Down	0.53554	0.703318867
68251	1	0	4.26	0.001	-12.05663772	Down	0.53554	0.702542699
80297	3	0	4.24	0.001	-12.04984855	Down	0.1434012	0.524732359
19700	1	0	4.23	0.001	-12.04644195	Down	0.53554	0.785930924
13803	3	0	4.22	0.001	-12.04302728	Down	0.1434012	0.560733734
12766	1	0	4.22	0.001	-12.04302728	Down	0.53554	0.722124184
16328	5	0	4.21	0.001	-12.03960452	Down	0.0383984	0.322089805
226695	1	0	4.21	0.001	-12.03960452	Down	0.53554	0.736932089
68401	1	0	4.21	0.001	-12.03960452	Down	0.53554	0.710949001

65115	2	0	4.2	0.001	-12.03617361	Down	0.277122	0.660347125
74410	2	0	4.2	0.001	-12.03617361	Down	0.277122	0.638231985
14465	2	0	4.2	0.001	-12.03617361	Down	0.277122	0.631416255
16332	3	0	4.19	0.001	-12.03273453	Down	0.1434012	0.543617943
12334	2	0	4.18	0.001	-12.02928723	Down	0.277122	0.713027879
23871	3	0	4.17	0.001	-12.02583167	Down	0.1434012	0.585496264
22169	2	0	4.17	0.001	-12.02583167	Down	0.277122	0.623561969
26386	1	0	4.16	0.001	-12.02236781	Down	0.53554	0.812439077
108671	1	0	4.16	0.001	-12.02236781	Down	0.53554	0.779060899
232941	1	0	4.16	0.001	-12.02236781	Down	0.53554	0.74332416
67903	1	0	4.15	0.001	-12.01889562	Down	0.53554	0.741099009
208154	2	0	4.15	0.001	-12.01889562	Down	0.277122	0.633149962
67477	2	0	4.13	0.001	-12.01192607	Down	0.277122	0.653423679
381845	2	0	4.12	0.001	-12.00842862	Down	0.277122	0.680617521
19182	1	0	4.11	0.001	-12.00492268	Down	0.53554	0.709477526
78506	2	0	4.1	0.001	-12.00140819	Down	0.277122	0.613116767
103199	2	0	4.09	0.001	-11.99788513	Down	0.277122	0.65959158
22334	1	0	4.08	0.001	-11.99435344	Down	0.53554	0.817956744
58184	2	0	4.08	0.001	-11.99435344	Down	0.277122	0.695395706
13821	4	0	4.08	0.001	-11.99435344	Down	0.074205	0.433914129
215031	1	0	4.07	0.001	-11.99081308	Down	0.53554	0.826451843
53333	1	0	4.06	0.001	-11.98726401	Down	0.53554	0.735836912
245945	3	0	4.05	0.001	-11.98370619	Down	0.1434012	0.573398071
12946	1	0	4.05	0.001	-11.98370619	Down	0.53554	0.770174272
71206	1	0	4.05	0.001	-11.98370619	Down	0.53554	0.757475495
68066	1	0	4.04	0.001	-11.98013958	Down	0.53554	0.74097578

59004	1	0	4.04	0.001	-11.98013958	Down	0.53554	0.735229886
75420	2	0	4.03	0.001	-11.97656412	Down	0.277122	0.697288226
12053	2	0	4.03	0.001	-11.97656412	Down	0.277122	0.696656242
227674	2	0	4.03	0.001	-11.97656412	Down	0.277122	0.69518606
108156	2	0	4.03	0.001	-11.97656412	Down	0.277122	0.65565316
12475	1	0	4.03	0.001	-11.97656412	Down	0.53554	0.693468462
70415	2	0	4.02	0.001	-11.97297979	Down	0.277122	0.674052079
105511	1	0	4	0.001	-11.96578428	Down	0.53554	0.7696422
12226	3	0	4	0.001	-11.96578428	Down	0.1434012	0.547107467
224024	2	0	3.99	0.001	-11.96217303	Down	0.277122	0.660158077
66978	1	0	3.99	0.001	-11.96217303	Down	0.53554	0.725770088
66314	2	0	3.98	0.001	-11.95855272	Down	0.277122	0.678814296
112419	2	0	3.98	0.001	-11.95855272	Down	0.277122	0.639116453
68047	1	0	3.98	0.001	-11.95855272	Down	0.53554	0.694332867
66274	3	0	3.96	0.001	-11.95128471	Down	0.1434012	0.568210183
15446	1	0	3.94	0.001	-11.94397991	Down	0.53554	0.799323469
17681	1	0	3.94	0.001	-11.94397991	Down	0.53554	0.713567388
13449	1	0	3.94	0.001	-11.94397991	Down	0.53554	0.698358931
545260	1	0	3.93	0.001	-11.9403136	Down	0.53554	0.718167339
17218	2	0	3.92	0.001	-11.93663794	Down	0.277122	0.648462363
104110	2	0	3.92	0.001	-11.93663794	Down	0.277122	0.627464534
12224	1	0	3.92	0.001	-11.93663794	Down	0.53554	0.708913194
71323	1	0	3.91	0.001	-11.93295289	Down	0.53554	0.820366042
11747	1	0	3.9	0.001	-11.92925841	Down	0.53554	0.811551328
12266	3	0	3.9	0.001	-11.92925841	Down	0.1434012	0.566591351
99543	1	0	3.9	0.001	-11.92925841	Down	0.53554	0.724001355

71062	1	0	3.89	0.001	-11.92555444	Down	0.53554	0.795755061
74448	1	0	3.89	0.001	-11.92555444	Down	0.53554	0.755549057
13388	2	0	3.89	0.001	-11.92555444	Down	0.277122	0.624068244
15451	1	0	3.88	0.001	-11.92184094	Down	0.53554	0.770973761
23993	1	0	3.88	0.001	-11.92184094	Down	0.53554	0.70454203
13669	3	0	3.87	0.001	-11.91811785	Down	0.1434012	0.565785389
104718	2	0	3.87	0.001	-11.91811785	Down	0.277122	0.663959736
68083	1	0	3.86	0.001	-11.91438513	Down	0.53554	0.81690712
12176	1	0	3.86	0.001	-11.91438513	Down	0.53554	0.779878953
76687	2	0	3.85	0.001	-11.91064273	Down	0.277122	0.693304919
14042	2	0	3.85	0.001	-11.91064273	Down	0.277122	0.615571853
56753	1	0	3.84	0.001	-11.9068906	Down	0.53554	0.700114429
12593	2	0	3.83	0.001	-11.90312868	Down	0.277122	0.682632375
18457	2	0	3.83	0.001	-11.90312868	Down	0.277122	0.64718837
78748	2	0	3.83	0.001	-11.90312868	Down	0.277122	0.636646097
244585	4	0	3.82	0.001	-11.89935692	Down	0.074205	0.42908951
26417	1	0	3.82	0.001	-11.89935692	Down	0.53554	0.725061559
23924	1	0	3.81	0.001	-11.89557528	Down	0.53554	0.754781223
72181	2	0	3.8	0.001	-11.8917837	Down	0.277122	0.703028098
12036	1	0	3.8	0.001	-11.8917837	Down	0.53554	0.789831326
56228	2	0	3.8	0.001	-11.8917837	Down	0.277122	0.676028192
19275	2	0	3.79	0.001	-11.88798213	Down	0.277122	0.69164132
18806	1.97	0	3.79	0.001	-11.88798213	Down	0.53554	0.705099421
433864	2	0	3.78	0.001	-11.88417052	Down	0.277122	0.706474314
434756	1	0	3.78	0.001	-11.88417052	Down	0.53554	0.82522747
101568	1	0	3.78	0.001	-11.88417052	Down	0.53554	0.783443801

171486	2	0	3.78	0.001	-11.88417052	Down		0.277122	0.667225741
381314	2	0	3.78	0.001	-11.88417052	Down		0.277122	0.636997835
101831	1	0	3.78	0.001	-11.88417052	Down		0.53554	0.693144865
14686	1	0	3.77	0.001	-11.88034881	Down		0.53554	0.83076591
69544	1	0	3.75	0.001	-11.87267488	Down		0.53554	0.780835525
98363	1	0	3.75	0.001	-11.87267488	Down		0.53554	0.737419881
435145	1	0	3.75	0.001	-11.87267488	Down		0.53554	0.723883746
16512	2	0	3.74	0.001	-11.86882255	Down		0.277122	0.701530929
170755	3.02	0	3.74	0.001	-11.86882255	Down		0.1434012	0.56231922
66717	2	0	3.74	0.001	-11.86882255	Down		0.277122	0.670329117
18700	2	0	3.73	0.001	-11.86495992	Down		0.277122	0.695605479
12151	2	0	3.73	0.001	-11.86495992	Down		0.277122	0.619374741
226123	1	0	3.72	0.001	-11.86108691	Down		0.53554	0.832939877
13394	1	0	3.72	0.001	-11.86108691	Down		0.53554	0.771507677
227290	1	0	3.72	0.001	-11.86108691	Down		0.53554	0.768845469
107767	2	0	3.71	0.001	-11.85720347	Down		0.277122	0.686289334
26408	3	0	3.71	0.001	-11.85720347	Down		0.1434012	0.57284752
269401	3	0	3.71	0.001	-11.85720347	Down		0.1434012	0.567399613
20866	2	0	3.71	0.001	-11.85720347	Down		0.277122	0.638408683
74148	3	0	3.71	0.001	-11.85720347	Down		0.1434012	0.528216638
406219	1	0	3.7	0.001	-11.85330956	Down		0.53554	0.759929799
13857	1	0	3.7	0.001	-11.85330956	Down		0.53554	0.730529236
24100	1	0	3.7	0.001	-11.85330956	Down		0.53554	0.703429888
235431	2	0	3.69	0.001	-11.8494051	Down		0.277122	0.712807469
208643	3	0	3.69	0.001	-11.8494051	Down		0.1434012	0.569838293
11933	1	0	3.69	0.001	-11.8494051	Down		0.53554	0.738764645

234094	3	0	3.69	0.001	-11.8494051	Down	0.1434012	0.533411437
12258	1	0	3.68	0.001	-11.84549005	Down	0.53554	0.842068847
71704	2	0	3.68	0.001	-11.84549005	Down	0.277122	0.693513432
67661	3	0	3.68	0.001	-11.84549005	Down	0.1434012	0.564981716
18655	1	0	3.68	0.001	-11.84549005	Down	0.53554	0.696285678
15982	1	0	3.68	0.001	-11.84549005	Down	0.53554	0.690139127
11541	1	0	3.67	0.001	-11.84156435	Down	0.53554	0.809634509
74868	2	0	3.67	0.001	-11.84156435	Down	0.277122	0.688749152
56321	1	0	3.67	0.001	-11.84156435	Down	0.53554	0.792218372
66962	1	0	3.66	0.001	-11.83762793	Down	0.53554	0.84398264
286940	5	0	3.66	0.001	-11.83762793	Down	0.0383984	0.324049783
19204	2	0	3.66	0.001	-11.83762793	Down	0.277122	0.694976541
76509	1	0	3.66	0.001	-11.83762793	Down	0.53554	0.734744986
12982	1	0	3.66	0.001	-11.83762793	Down	0.53554	0.711062445
12724	2	0	3.65	0.001	-11.83368075	Down	0.277122	0.611166754
105278	1	0	3.65	0.001	-11.83368075	Down	0.53554	0.694765878
216344	1	0	3.64	0.001	-11.82972274	Down	0.53554	0.801191719
75823	1	0	3.64	0.001	-11.82972274	Down	0.53554	0.787876298
208084	2	0	3.64	0.001	-11.82972274	Down	0.277122	0.633323857
11861	2	0	3.63	0.001	-11.82575383	Down	0.277122	0.645557716
16785	1	0	3.63	0.001	-11.82575383	Down	0.53554	0.698468392
70750	3.03	0	3.62	0.001	-11.82177398	Down	0.1434012	0.583206933
237988	2	0	3.62	0.001	-11.82177398	Down	0.277122	0.677816626
76477	1	0	3.62	0.001	-11.82177398	Down	0.53554	0.737786149
26562	2	0	3.62	0.001	-11.82177398	Down	0.277122	0.634543798
66716	1	0	3.62	0.001	-11.82177398	Down	0.53554	0.713110632

108670	1	0	3.62	0.001	-11.82177398	Down	0.53554	0.712882473
56295	1	0	3.61	0.001	-11.81778312	Down	0.53554	0.777972825
110639	2	0	3.61	0.001	-11.81778312	Down	0.277122	0.664150968
109305	1	0	3.61	0.001	-11.81778312	Down	0.53554	0.717935934
18082	1	0	3.61	0.001	-11.81778312	Down	0.53554	0.702764286
105833	1	0	3.6	0.001	-11.81378119	Down	0.53554	0.838898407
225363	2	0	3.6	0.001	-11.81378119	Down	0.277122	0.701104336
14673	1	0	3.6	0.001	-11.81378119	Down	0.53554	0.818407409
12686	1	0	3.6	0.001	-11.81378119	Down	0.53554	0.810370675
20844	2	0	3.6	0.001	-11.81378119	Down	0.277122	0.687517043
15402	1	0	3.6	0.001	-11.81378119	Down	0.53554	0.79518707
21824	2	0	3.6	0.001	-11.81378119	Down	0.277122	0.660725548
22059	1	0	3.6	0.001	-11.81378119	Down	0.53554	0.740606339
20336	2	0	3.6	0.001	-11.81378119	Down	0.277122	0.609551193
56532	1	0	3.59	0.001	-11.80976813	Down	0.53554	0.735351211
233280	1	0	3.59	0.001	-11.80976813	Down	0.53554	0.728975681
100910	2	0	3.59	0.001	-11.80976813	Down	0.277122	0.631935369
20474	3	0	3.59	0.001	-11.80976813	Down	0.1434012	0.535085823
67487	2	0	3.59	0.001	-11.80976813	Down	0.277122	0.623224909
68750	4	0	3.58	0.001	-11.80574387	Down	0.074205	0.441357974
13681	1	0	3.58	0.001	-11.80574387	Down	0.53554	0.743448172
319229	1	0	3.58	0.001	-11.80574387	Down	0.53554	0.71128944
67949	1	0	3.57	0.001	-11.80170836	Down	0.53554	0.803792991
223722	1	0	3.57	0.001	-11.80170836	Down	0.53554	0.785376866
114128	1	0	3.56	0.001	-11.79766153	Down	0.53554	0.843503377
67109	1	0	3.56	0.001	-11.79766153	Down	0.53554	0.833407208

15110	1	0	3.56	0.001	-11.79766153	Down	0.53554	0.824616643
213498	3	0	3.56	0.001	-11.79766153	Down	0.1434012	0.584349356
13848	2	0	3.56	0.001	-11.79766153	Down	0.277122	0.701317568
73847	1	0	3.56	0.001	-11.79766153	Down	0.53554	0.810223335
13510	3	0	3.56	0.001	-11.79766153	Down	0.1434012	0.573673743
14682	3	0	3.55	0.001	-11.79360331	Down	0.1434012	0.578401059
12416	2	0	3.55	0.001	-11.79360331	Down	0.277122	0.678614527
239126	1	0	3.55	0.001	-11.79360331	Down	0.53554	0.750712321
232237	3	0	3.55	0.001	-11.79360331	Down	0.1434012	0.538466329
434178	1.99	0	3.54	0.001	-11.78953364	Down	0.53554	0.779469711
68262	1	0	3.54	0.001	-11.78953364	Down	0.53554	0.779197122
71914	2	0	3.54	0.001	-11.78953364	Down	0.277122	0.669356215
414801	2	0	3.54	0.001	-11.78953364	Down	0.277122	0.653979626
57354	3.99	0	3.53	0.001	-11.78545247	Down	0.1434012	0.550134341
108989	4	0	3.53	0.001	-11.78545247	Down	0.074205	0.407564228
73230	2	0	3.52	0.001	-11.78135971	Down	0.277122	0.71590567
110147	2	0	3.52	0.001	-11.78135971	Down	0.277122	0.637702478
16527	2	0	3.51	0.001	-11.77725532	Down	0.277122	0.715239504
101744	1	0	3.51	0.001	-11.77725532	Down	0.53554	0.830920817
107566	1	0	3.5	0.001	-11.77313921	Down	0.53554	0.796750999
227331	3	0	3.5	0.001	-11.77313921	Down	0.1434012	0.56099736
234684	1	0	3.5	0.001	-11.77313921	Down	0.53554	0.723531148
14420	2	0	3.5	0.001	-11.77313921	Down	0.277122	0.611653093
19271	4	0	3.49	0.001	-11.76901132	Down	0.074205	0.439160601
54326	2	0	3.49	0.001	-11.76901132	Down	0.277122	0.665300681
12288	7	0	3.49	0.001	-11.76901132	Down	0.01028194	0.14550344

73703	1	0	3.48	0.001	-11.76487159	Down	0.53554	0.701216104
245860	2	0	3.47	0.001	-11.76071995	Down	0.277122	0.658085663
107527	1	0	3.46	0.001	-11.75655632	Down	0.53554	0.793912051
13992	1	0	3.46	0.001	-11.75655632	Down	0.53554	0.774996233
108013	2	0	3.46	0.001	-11.75655632	Down	0.277122	0.667805434
219094	2	0	3.45	0.001	-11.75238065	Down	0.277122	0.683239159
320712	2	0	3.45	0.001	-11.75238065	Down	0.277122	0.628148233
214601	1	0	3.45	0.001	-11.75238065	Down	0.53554	0.709138819
12042	1	0	3.45	0.001	-11.75238065	Down	0.53554	0.706552773
17534	3	0	3.45	0.001	-11.75238065	Down	0.1434012	0.523581126
12461	1	0	3.44	0.001	-11.74819285	Down	0.53554	0.831230804
11767	1	0	3.44	0.001	-11.74819285	Down	0.53554	0.789411575
233040	1	0	3.44	0.001	-11.74819285	Down	0.53554	0.718978435
99237	2	0	3.44	0.001	-11.74819285	Down	0.277122	0.626271636
243967	1	0	3.43	0.001	-11.74399286	Down	0.53554	0.800472129
20459	1.16	0	3.42	0.001	-11.73978061	Down	0.53554	0.844782624
22213	1	0	3.41	0.001	-11.73555602	Down	0.53554	0.810518068
224796	3	0	3.41	0.001	-11.73555602	Down	0.1434012	0.550642079
70719	2	0	3.41	0.001	-11.73555602	Down	0.277122	0.634369233
107589	4	0	3.41	0.001	-11.73555602	Down	0.074205	0.409727807
106639	1	0	3.4	0.001	-11.73131903	Down	0.53554	0.817356629
66958	1	0	3.4	0.001	-11.73131903	Down	0.53554	0.812735426
269053	2	0	3.4	0.001	-11.73131903	Down	0.277122	0.652683884
12797	1	0	3.4	0.001	-11.73131903	Down	0.53554	0.748694278
52331	1	0	3.4	0.001	-11.73131903	Down	0.53554	0.733535529
18788	1	0	3.4	0.001	-11.73131903	Down	0.53554	0.71151658

17686	2	0	3.39	0.001	-11.72706956	Down	0.277122	0.676623287
15081	1	0	3.39	0.001	-11.72706956	Down	0.53554	0.781931627
68040	1	0	3.39	0.001	-11.72706956	Down	0.53554	0.747814791
74362	4	0	3.39	0.001	-11.72706956	Down	0.074205	0.409999871
231549	2	0	3.39	0.001	-11.72706956	Down	0.277122	0.611004812
108077	2	0	3.37	0.001	-11.71853288	Down	0.277122	0.690398851
268980	2	0	3.37	0.001	-11.71853288	Down	0.277122	0.666261821
11815	1	0	3.37	0.001	-11.71853288	Down	0.53554	0.764230551
381903	1	0	3.37	0.001	-11.71853288	Down	0.53554	0.690459923
66822	1	0	3.36	0.001	-11.71424552	Down	0.53554	0.814964949
52013	2	0	3.36	0.001	-11.71424552	Down	0.277122	0.630725427
194655	2	0	3.36	0.001	-11.71424552	Down	0.277122	0.620207682
620631	1	0	3.36	0.001	-11.71424552	Down	0.53554	0.706440764
12018	1	0	3.35	0.001	-11.70994538	Down	0.53554	0.801479917
57028	1	0	3.35	0.001	-11.70994538	Down	0.53554	0.77324802
108943	1.97	0	3.35	0.001	-11.70994538	Down	0.53554	0.710722223
64294	1	0	3.34	0.001	-11.70563239	Down	0.53554	0.819460894
70317	1	0	3.34	0.001	-11.70563239	Down	0.53554	0.722475412
69585	1	0	3.34	0.001	-11.70563239	Down	0.53554	0.701657745
16007	1	0	3.33	0.001	-11.70130646	Down	0.53554	0.804809164
76815	1	0	3.33	0.001	-11.70130646	Down	0.53554	0.706664818
74754	2	0	3.32	0.001	-11.69696753	Down	0.277122	0.677418379
79264	3	0	3.32	0.001	-11.69696753	Down	0.1434012	0.558895262
72404	2	0	3.32	0.001	-11.69696753	Down	0.277122	0.628661985
54006	1	0	3.32	0.001	-11.69696753	Down	0.53554	0.719791365
104383	1	0	3.32	0.001	-11.69696753	Down	0.53554	0.694441069

15377	1	0	3.31	0.001	-11.6926155	Down	0.53554	0.783581561
228012	2	0	3.3	0.001	-11.68825031	Down	0.277122	0.66741886
224860	2	0	3.29	0.001	-11.68387187	Down	0.277122	0.715017725
22201	2	0	3.29	0.001	-11.68387187	Down	0.277122	0.647551857
238875	1	0	3.28	0.001	-11.6794801	Down	0.53554	0.729811389
67776	2	0	3.28	0.001	-11.6794801	Down	0.277122	0.631243406
22774	2	0	3.27	0.001	-11.67507492	Down	0.277122	0.692680133
14718	1	0	3.27	0.001	-11.67507492	Down	0.53554	0.750080515
271047	1	0	3.26	0.001	-11.67065625	Down	0.53554	0.76409951
68177	1	0	3.25	0.001	-11.666224	Down	0.53554	0.823093524
22626	1	0	3.25	0.001	-11.666224	Down	0.53554	0.722358298
217578	3	0	3.24	0.001	-11.6617781	Down	0.1434012	0.542136022
19060	1	0	3.24	0.001	-11.6617781	Down	0.53554	0.700444568
72129	2	0	3.24	0.001	-11.6617781	Down	0.277122	0.609873621
108652	1	0	3.23	0.001	-11.65731845	Down	0.53554	0.778244558
83679	3	0	3.23	0.001	-11.65731845	Down	0.1434012	0.559157163
74533	2	0	3.22	0.001	-11.65284497	Down	0.277122	0.713910886
75687	2	0	3.22	0.001	-11.65284497	Down	0.277122	0.705178031
21885	1	0	3.22	0.001	-11.65284497	Down	0.53554	0.772711694
15275	2	0	3.22	0.001	-11.65284497	Down	0.277122	0.663005222
98366	1	0	3.22	0.001	-11.65284497	Down	0.53554	0.69163873
116905	1	0	3.21	0.001	-11.64835758	Down	0.53554	0.801768323
15979	1	0	3.21	0.001	-11.64835758	Down	0.53554	0.794761609
270091	1	0	3.21	0.001	-11.64835758	Down	0.53554	0.791936794
114566	1	0	3.21	0.001	-11.64835758	Down	0.53554	0.721305979
110075	3	0	3.21	0.001	-11.64835758	Down	0.1434012	0.533173094

12876	1	0	3.21	0.001	-11.64835758	Down	0.53554	0.692713872
230796	2	0	3.2	0.001	-11.64385619	Down	0.277122	0.669161974
53859	2	0	3.2	0.001	-11.64385619	Down	0.277122	0.664917002
20502	2	0	3.2	0.001	-11.64385619	Down	0.277122	0.657710257
72825	1	0	3.19	0.001	-11.63934071	Down	0.53554	0.799036819
72446	1.98	0	3.19	0.001	-11.63934071	Down	0.53554	0.782343459
209478	2	0	3.18	0.001	-11.63481105	Down	0.277122	0.691019527
13711	1	0	3.17	0.001	-11.63026713	Down	0.53554	0.819159621
442801	2	0	3.17	0.001	-11.63026713	Down	0.277122	0.682834516
242521	2	0	3.17	0.001	-11.63026713	Down	0.277122	0.634194764
13016	1	0	3.16	0.001	-11.62570884	Down	0.53554	0.806703175
74685	1	0	3.16	0.001	-11.62570884	Down	0.53554	0.795328992
67864	1	0	3.16	0.001	-11.62570884	Down	0.53554	0.761618243
53951	2	0	3.16	0.001	-11.62570884	Down	0.277122	0.638762372
14634	4	0	3.16	0.001	-11.62570884	Down	0.074205	0.414124618
244867	3	0	3.14	0.001	-11.61654884	Down	0.1434012	0.5596817
71461	2	0	3.14	0.001	-11.61654884	Down	0.277122	0.665684804
67050	2	0	3.14	0.001	-11.61654884	Down	0.277122	0.638585478
30044	1	0	3.13	0.001	-11.61194694	Down	0.53554	0.778516481
68652	2	0	3.13	0.001	-11.61194694	Down	0.277122	0.620374539
217341	1	0	3.12	0.001	-11.60733031	Down	0.53554	0.801912604
67026	1	0	3.12	0.001	-11.60733031	Down	0.53554	0.757990873
28114	2	0	3.12	0.001	-11.60733031	Down	0.277122	0.629176579
241075	4	0	3.11	0.001	-11.60269887	Down	0.074205	0.426717211
74775	1	0	3.11	0.001	-11.60269887	Down	0.53554	0.726954052
11905	1	0	3.11	0.001	-11.60269887	Down	0.53554	0.724236688

109168	3	0	3.11	0.001	-11.60269887	Down	0.1434012	0.522663769
56524	1	0	3.09	0.001	-11.59339112	Down	0.53554	0.837164821
71529	2	0	3.09	0.001	-11.59339112	Down	0.277122	0.663195905
70420	1	0	3.09	0.001	-11.59339112	Down	0.53554	0.756832259
17339	1	0	3.09	0.001	-11.59339112	Down	0.53554	0.690888115
72462	2.07	0	3.08	0.001	-11.58871464	Down	0.277122	0.715461422
211612	3	0	3.08	0.001	-11.58871464	Down	0.1434012	0.555512749
20255	1	0	3.08	0.001	-11.58871464	Down	0.53554	0.74406885
76089	3	0	3.07	0.001	-11.58402294	Down	0.1434012	0.554737975
22165	1	0	3.07	0.001	-11.58402294	Down	0.53554	0.735472576
20907	1	0	3.06	0.001	-11.57931594	Down	0.53554	0.805536576
30941	1	0	3.06	0.001	-11.57931594	Down	0.53554	0.711743865
218518	1	0	3.06	0.001	-11.57931594	Down	0.53554	0.702986014
56030	3	0	3.05	0.001	-11.57459353	Down	0.1434012	0.58349212
227120	3	0	3.05	0.001	-11.57459353	Down	0.1434012	0.579243391
11810	1	0	3.05	0.001	-11.57459353	Down	0.53554	0.802345758
22697	1	0	3.04	0.001	-11.56985561	Down	0.53554	0.834343445
15170	1	0	3.04	0.001	-11.56985561	Down	0.53554	0.815710844
29809	2	0	3.04	0.001	-11.56985561	Down	0.277122	0.684049885
171210	1	0	3.04	0.001	-11.56985561	Down	0.53554	0.755933561
69470	2	0	3.04	0.001	-11.56985561	Down	0.277122	0.651209309
22746	1	0	3.04	0.001	-11.56985561	Down	0.53554	0.73438173
233033	2	0	3.04	0.001	-11.56985561	Down	0.277122	0.632802459
100090	1	0	3.03	0.001	-11.56510208	Down	0.53554	0.80075981
17922	3	0	3.03	0.001	-11.56510208	Down	0.1434012	0.563381202
216157	1	0	3.03	0.001	-11.56510208	Down	0.53554	0.761227936

11787	3	0	3.03	0.001	-11.56510208	Down	0.1434012	0.543370394
21353	1	0	3.03	0.001	-11.56510208	Down	0.53554	0.693792362
218232	2	0	3.02	0.001	-11.56033283	Down	0.277122	0.649558355
16997	3	0	3.02	0.001	-11.56033283	Down	0.1434012	0.548112717
12315	1	0	3.02	0.001	-11.56033283	Down	0.53554	0.718051618
18845	5	0	3.01	0.001	-11.55554777	Down	0.0383984	0.323066821
16402	2	0	3.01	0.001	-11.55554777	Down	0.277122	0.654908311
66771	2	0	3.01	0.001	-11.55554777	Down	0.277122	0.633497847
22153	1	0	3	0.001	-11.55074679	Down	0.53554	0.833251372
75050	2	0	3	0.001	-11.55074679	Down	0.277122	0.674446377
67057	1	0	3	0.001	-11.55074679	Down	0.53554	0.736201609
224762	1	0	3	0.001	-11.55074679	Down	0.53554	0.720373156
70178	1	0	2.99	0.001	-11.54592977	Down	0.53554	0.768712841
20354	2	0	2.98	0.001	-11.54109662	Down	0.277122	0.704746993
70997	1	0	2.98	0.001	-11.54109662	Down	0.53554	0.781383191
114741	2	0	2.98	0.001	-11.54109662	Down	0.277122	0.668967845
232944	1	0	2.98	0.001	-11.54109662	Down	0.53554	0.718630598
67500	2	0	2.98	0.001	-11.54109662	Down	0.277122	0.61344298
67845	1	0	2.97	0.001	-11.53624722	Down	0.53554	0.842227998
381678	1	0	2.97	0.001	-11.53624722	Down	0.53554	0.74718785
67048	2	0	2.97	0.001	-11.53624722	Down	0.277122	0.644115129
14199	1	0	2.96	0.001	-11.53138146	Down	0.53554	0.79975383
16687	1	0	2.95	0.001	-11.52649924	Down	0.53554	0.835438384
320299	1	0	2.95	0.001	-11.52649924	Down	0.53554	0.814815933
66841	1	0	2.95	0.001	-11.52649924	Down	0.53554	0.77445748
23834	2	0	2.95	0.001	-11.52649924	Down	0.277122	0.657147952

16576	2	0	2.95	0.001	-11.52649924	Down	0.277122	0.652130136
18605	3	0	2.95	0.001	-11.52649924	Down	0.1434012	0.534127746
140559	1	0	2.95	0.001	-11.52649924	Down	0.53554	0.697157125
13166	1	0	2.95	0.001	-11.52649924	Down	0.53554	0.695524948
394435	1	0	2.94	0.001	-11.52160044	Down	0.53554	0.804954541
77805	2	0	2.94	0.001	-11.52160044	Down	0.277122	0.645015989
12566	1	0	2.94	0.001	-11.52160044	Down	0.53554	0.730409497
67283	1	0	2.94	0.001	-11.52160044	Down	0.53554	0.710835594
68015	1	0	2.93	0.001	-11.51668495	Down	0.53554	0.834499689
235406	2	0	2.93	0.001	-11.51668495	Down	0.277122	0.694767147
223753	2	0	2.93	0.001	-11.51668495	Down	0.277122	0.641606055
16870	1	0	2.93	0.001	-11.51668495	Down	0.53554	0.704876359
16525	1	0	2.92	0.001	-11.51175265	Down	0.53554	0.811403558
12466	1	0	2.92	0.001	-11.51175265	Down	0.53554	0.770573809
67557	1	0	2.92	0.001	-11.51175265	Down	0.53554	0.70309693
13506	2	0	2.91	0.001	-11.50680344	Down	0.277122	0.659026054
212123	1	0	2.91	0.001	-11.50680344	Down	0.53554	0.713681669
21872	3	0	2.9	0.001	-11.50183718	Down	0.1434012	0.528919054
100502766	1	0	2.89	0.001	-11.49685378	Down	0.53554	0.782206133
18569	1	0	2.89	0.001	-11.49685378	Down	0.53554	0.764623943
67180	1	0	2.89	0.001	-11.49685378	Down	0.53554	0.75033311
71519	1	0	2.89	0.001	-11.49685378	Down	0.53554	0.72541565
71954	1	0	2.89	0.001	-11.49685378	Down	0.53554	0.7095905
242735	1	0	2.89	0.001	-11.49685378	Down	0.53554	0.704208018
242384	2	0	2.88	0.001	-11.4918531	Down	0.277122	0.669939617

269955	1	0	2.88	0.001	-11.4918531	Down	0.53554	0.772577729
231717	1	0	2.88	0.001	-11.4918531	Down	0.53554	0.755036994
65221	1	0	2.87	0.001	-11.48683502	Down	0.53554	0.807580344
192652	3	0	2.87	0.001	-11.48683502	Down	0.1434012	0.565249353
217340	2	0	2.87	0.001	-11.48683502	Down	0.277122	0.647006779
17872	1	0	2.87	0.001	-11.48683502	Down	0.53554	0.71391034
225913	1	0	2.87	0.001	-11.48683502	Down	0.53554	0.705881251
14824	1	0	2.87	0.001	-11.48683502	Down	0.53554	0.696721129
30791	1	0	2.87	0.001	-11.48683502	Down	0.53554	0.692498577
664883	3	0	2.86	0.001	-11.48179943	Down	0.1434012	0.586647682
216161	2	0	2.86	0.001	-11.48179943	Down	0.277122	0.698767322
223272	1	0	2.86	0.001	-11.48179943	Down	0.53554	0.736688434
80837	1	0	2.86	0.001	-11.48179943	Down	0.53554	0.733173468
17129	3	0	2.86	0.001	-11.48179943	Down	0.1434012	0.531037555
56079	2	0	2.86	0.001	-11.48179943	Down	0.277122	0.606824253
50850	2	0	2.85	0.001	-11.4767462	Down	0.277122	0.698132656
68291	1	0	2.85	0.001	-11.4767462	Down	0.53554	0.797178594
103220	1	0	2.85	0.001	-11.4767462	Down	0.53554	0.783168425
225280	1	0	2.85	0.001	-11.4767462	Down	0.53554	0.755805349
239368	1	0	2.84	0.001	-11.47167521	Down	0.53554	0.804663839
16571	2	0	2.84	0.001	-11.47167521	Down	0.277122	0.681220727
19882	2	0	2.84	0.001	-11.47167521	Down	0.277122	0.637878883
171212	2	0	2.83	0.001	-11.46658634	Down	0.277122	0.648280057
13046	2	0	2.83	0.001	-11.46658634	Down	0.277122	0.622383849
65100	1	0	2.82	0.001	-11.46147945	Down	0.53554	0.830146859
353172	1	0	2.82	0.001	-11.46147945	Down	0.53554	0.709816556

14182	2	0	2.81	0.001	-11.45635442	Down	0.277122	0.709954483
17220	1	0	2.81	0.001	-11.45635442	Down	0.53554	0.808459423
72149	1	0	2.81	0.001	-11.45635442	Down	0.53554	0.721539563
235442	2	0	2.81	0.001	-11.45635442	Down	0.277122	0.617219529
15200	1	0	2.81	0.001	-11.45635442	Down	0.53554	0.701326462
67976	1	0	2.81	0.001	-11.45635442	Down	0.53554	0.698906578
11836	1	0	2.8	0.001	-11.45121111	Down	0.53554	0.833563101
66663	1	0	2.8	0.001	-11.45121111	Down	0.53554	0.79179608
18141	2	0	2.8	0.001	-11.45121111	Down	0.277122	0.636821917
57813	1	0	2.8	0.001	-11.45121111	Down	0.53554	0.712198872
269788	2	0	2.79	0.001	-11.44604941	Down	0.277122	0.692264234
57764	1	0	2.79	0.001	-11.44604941	Down	0.53554	0.725297581
67834	1	0	2.78	0.001	-11.44086917	Down	0.53554	0.833719053
107733	1	0	2.78	0.001	-11.44086917	Down	0.53554	0.785792336
69602	1	0	2.78	0.001	-11.44086917	Down	0.53554	0.764361636
22282	1	0	2.78	0.001	-11.44086917	Down	0.53554	0.732570827
23908	2	0	2.78	0.001	-11.44086917	Down	0.277122	0.628490641
223775	1	0	2.78	0.001	-11.44086917	Down	0.53554	0.707562455
108888	1	0	2.77	0.001	-11.43567026	Down	0.53554	0.845263342
103534	1	0	2.77	0.001	-11.43567026	Down	0.53554	0.802056937
80281	2	0	2.77	0.001	-11.43567026	Down	0.277122	0.620541486
27219	1	0	2.76	0.001	-11.43045255	Down	0.53554	0.838267182
328162	1	0	2.76	0.001	-11.43045255	Down	0.53554	0.781246203
432769	1	0	2.76	0.001	-11.43045255	Down	0.53554	0.715630053
246788	1	0	2.76	0.001	-11.43045255	Down	0.53554	0.693900396
22384	1	0	2.75	0.001	-11.4252159	Down	0.53554	0.787597798

77035	1	0	2.75	0.001	-11.4252159	Down	0.53554	0.779333393
15278	1	0	2.75	0.001	-11.4252159	Down	0.53554	0.756318456
23880	2	0	2.75	0.001	-11.4252159	Down	0.277122	0.634020391
223918	1	0	2.74	0.001	-11.41996018	Down	0.53554	0.822637685
12662	2	0	2.74	0.001	-11.41996018	Down	0.277122	0.694348739
20971	1	0	2.74	0.001	-11.41996018	Down	0.53554	0.766465143
170768	2	0	2.74	0.001	-11.41996018	Down	0.277122	0.652868676
381983	2	0	2.74	0.001	-11.41996018	Down	0.277122	0.611490894
67053	1	0	2.73	0.001	-11.41468524	Down	0.53554	0.823397698
210029	1	0	2.73	0.001	-11.41468524	Down	0.53554	0.820517094
53610	1	0	2.73	0.001	-11.41468524	Down	0.53554	0.783995134
22235	1	0	2.73	0.001	-11.41468524	Down	0.53554	0.768580259
234776	2	0	2.73	0.001	-11.41468524	Down	0.277122	0.621210173
72148	1	0	2.72	0.001	-11.40939094	Down	0.53554	0.786346981
67465	2	0	2.72	0.001	-11.40939094	Down	0.277122	0.649375433
11421	2	0	2.72	0.001	-11.40939094	Down	0.277122	0.63228192
78834	1	0	2.71	0.001	-11.40407714	Down	0.53554	0.798893571
216859	1	0	2.71	0.001	-11.40407714	Down	0.53554	0.754909087
76688	1	0	2.71	0.001	-11.40407714	Down	0.53554	0.739500222
16456	1	0	2.71	0.001	-11.40407714	Down	0.53554	0.730649015
66569	1	0	2.71	0.001	-11.40407714	Down	0.53554	0.695416408
14058	1.01	0	2.7	0.001	-11.39874369	Down	0.53554	0.815114019
319480	2	0	2.7	0.001	-11.39874369	Down	0.277122	0.69288827
667823	1	0	2.7	0.001	-11.39874369	Down	0.53554	0.790391689
20439	1	0	2.7	0.001	-11.39874369	Down	0.53554	0.785515308
71974	1	0	2.7	0.001	-11.39874369	Down	0.53554	0.73888714

13340	1	0	2.7	0.001	-11.39874369	Down	0.53554	0.73803053
19347	2	0	2.7	0.001	-11.39874369	Down	0.277122	0.635418066
19009	2	0	2.69	0.001	-11.39339046	Down	0.277122	0.642499906
69017	1	0	2.68	0.001	-11.38801729	Down	0.53554	0.781109262
69178	1	0	2.68	0.001	-11.38801729	Down	0.53554	0.773516462
71951	1	0	2.68	0.001	-11.38801729	Down	0.53554	0.697048074
74479	1	0	2.67	0.001	-11.38262403	Down	0.53554	0.775670729
15206	1	0	2.67	0.001	-11.38262403	Down	0.53554	0.751091916
207215	1	0	2.67	0.001	-11.38262403	Down	0.53554	0.749954281
100101806	1	0	2.67	0.001	-11.38262403	Down	0.53554	0.719442741
18984	1	0	2.66	0.001	-11.37721053	Down	0.53554	0.790952847
104009	1	0	2.66	0.001	-11.37721053	Down	0.53554	0.742952374
228482	2	0	2.66	0.001	-11.37721053	Down	0.277122	0.629692016
80883	2	0	2.65	0.001	-11.37177664	Down	0.277122	0.713689929
13427	1	0	2.65	0.001	-11.37177664	Down	0.53554	0.826758505
232430	1	0	2.65	0.001	-11.37177664	Down	0.53554	0.770840398
12389	1	0	2.65	0.001	-11.37177664	Down	0.53554	0.711630204
19876	3	0	2.64	0.001	-11.36632221	Down	0.1434012	0.578120826
319885	1	0	2.64	0.001	-11.36632221	Down	0.53554	0.777837029
12359	1	0	2.64	0.001	-11.36632221	Down	0.53554	0.720140326
18983	1	0	2.64	0.001	-11.36632221	Down	0.53554	0.700995492
22156	1	0	2.63	0.001	-11.36084708	Down	0.53554	0.792500149
216739	1	0	2.63	0.001	-11.36084708	Down	0.53554	0.757089422
224045	1	0	2.63	0.001	-11.36084708	Down	0.53554	0.69282157
15925	2	0	2.62	0.001	-11.3553511	Down	0.277122	0.702813826

240595	2	0	2.62	0.001	-11.3553511	Down		0.277122	0.675038689
228858	1	0	2.61	0.001	-11.34983409	Down		0.53554	0.814518066
110854	1	0	2.61	0.001	-11.34983409	Down		0.53554	0.806411209
53625	1	0	2.61	0.001	-11.34983409	Down		0.53554	0.80582791
70599	2	0	2.61	0.001	-11.34983409	Down		0.277122	0.668579925
226594	1	0	2.61	0.001	-11.34983409	Down		0.53554	0.740483274
14829	1	0	2.6	0.001	-11.34429591	Down		0.53554	0.831385884
109135	2	0	2.6	0.001	-11.34429591	Down		0.277122	0.701744419
627367	1	0	2.6	0.001	-11.34429591	Down		0.53554	0.766069854
235567	3	0	2.6	0.001	-11.34429591	Down		0.1434012	0.541152556
17350	1	0	2.59	0.001	-11.33873638	Down		0.53554	0.839056362
94094	1	0	2.59	0.001	-11.33873638	Down		0.53554	0.768050386
76800	2	0	2.59	0.001	-11.33873638	Down		0.277122	0.610357904
227327	1	0	2.58	0.001	-11.33315535	Down		0.53554	0.801047697
66468	1	0	2.58	0.001	-11.33315535	Down		0.53554	0.756190114
207181	3	0	2.58	0.001	-11.33315535	Down		0.1434012	0.52962334
320997	1	0	2.57	0.001	-11.32755264	Down		0.53554	0.843822825
230735	2	0	2.57	0.001	-11.32755264	Down		0.277122	0.683644282
230775	2	0	2.57	0.001	-11.32755264	Down		0.277122	0.654536521
100037258	2	0	2.57	0.001	-11.32755264	Down		0.277122	0.608746611
22756	1	0	2.56	0.001	-11.32192809	Down		0.53554	0.832784216
12335	1	0	2.56	0.001	-11.32192809	Down		0.53554	0.768978143
665775	4	0	2.56	0.001	-11.32192809	Down		0.074205	0.424079536
13076	1	0	2.55	0.001	-11.31628153	Down		0.53554	0.807287743
26382	1	0	2.55	0.001	-11.31628153	Down		0.53554	0.715515148
381801	1	0	2.54	0.001	-11.31061278	Down		0.53554	0.834812353

241639	2	0	2.54	0.001	-11.31061278	Down	0.277122	0.690812511
244698	2	0	2.54	0.001	-11.31061278	Down	0.277122	0.639470927
74343	1	0	2.54	0.001	-11.31061278	Down	0.53554	0.7170118
12263	1	0	2.54	0.001	-11.31061278	Down	0.53554	0.708237181
17913	2	0	2.53	0.001	-11.30492167	Down	0.277122	0.71346911
210853	1	0	2.53	0.001	-11.30492167	Down	0.53554	0.821121861
69538	2	0	2.53	0.001	-11.30492167	Down	0.277122	0.669745037
70445	1	0	2.53	0.001	-11.30492167	Down	0.53554	0.72482569
67439	1	0	2.53	0.001	-11.30492167	Down	0.53554	0.712085066
215335	2	0	2.53	0.001	-11.30492167	Down	0.277122	0.623056515
14086	1	0	2.52	0.001	-11.29920802	Down	0.53554	0.806557166
23877	1	0	2.52	0.001	-11.29920802	Down	0.53554	0.788154995
382090	2	0	2.52	0.001	-11.29920802	Down	0.277122	0.63717385
215384	3	0	2.51	0.001	-11.29347165	Down	0.1434012	0.564714333
65967	1	0	2.51	0.001	-11.29347165	Down	0.53554	0.770707081
13649	2	0	2.51	0.001	-11.29347165	Down	0.277122	0.632628851
230784	1	0	2.51	0.001	-11.29347165	Down	0.53554	0.704653438
13361	2	0	2.51	0.001	-11.29347165	Down	0.277122	0.615900684
407823	3	0	2.5	0.001	-11.28771238	Down	0.1434012	0.586936245
231842	1	0	2.5	0.001	-11.28771238	Down	0.53554	0.736323255
75964	1	0	2.49	0.001	-11.28193003	Down	0.53554	0.737908319
233802	1	0	2.49	0.001	-11.28193003	Down	0.53554	0.730289797
215387	1	0	2.49	0.001	-11.28193003	Down	0.53554	0.720722682
16969	2	0	2.48	0.001	-11.27612441	Down	0.277122	0.650474517
208665	1	0	2.48	0.001	-11.27612441	Down	0.53554	0.727904008
70312	1	0	2.47	0.001	-11.27029533	Down	0.53554	0.827679855

52690	1	0	2.47	0.001	-11.27029533	Down	0.53554	0.819611613
67070	1	0	2.47	0.001	-11.27029533	Down	0.53554	0.817656576
71544	2	0	2.47	0.001	-11.27029533	Down	0.277122	0.68445597
93790	1	0	2.47	0.001	-11.27029533	Down	0.53554	0.763837563
17969	1	0	2.47	0.001	-11.27029533	Down	0.53554	0.732811765
14784	1	0	2.47	0.001	-11.27029533	Down	0.53554	0.728141886
16538	1	0	2.46	0.001	-11.2644426	Down	0.53554	0.798321093
18733	1	0	2.46	0.001	-11.2644426	Down	0.53554	0.770041185
224044	1	0	2.46	0.001	-11.2644426	Down	0.53554	0.767389072
104816	1	0	2.46	0.001	-11.2644426	Down	0.53554	0.73974574
19294	1	0	2.46	0.001	-11.2644426	Down	0.53554	0.707225574
71340	1	0	2.45	0.001	-11.25856603	Down	0.53554	0.843024658
58996	2	0	2.45	0.001	-11.25856603	Down	0.277122	0.710173133
99377	1	0	2.45	0.001	-11.25856603	Down	0.53554	0.739377524
320736	1	0	2.45	0.001	-11.25856603	Down	0.53554	0.722007184
228983	1	0	2.45	0.001	-11.25856603	Down	0.53554	0.721773298
106581	1	0	2.44	0.001	-11.25266543	Down	0.53554	0.776887786
18751	1	0	2.44	0.001	-11.25266543	Down	0.53554	0.76083803
56072	1	0	2.44	0.001	-11.25266543	Down	0.53554	0.719558912
66270	1	0	2.43	0.001	-11.2467406	Down	0.53554	0.789971342
74186	1	0	2.43	0.001	-11.2467406	Down	0.53554	0.77540079
53972	1	0	2.43	0.001	-11.2467406	Down	0.53554	0.760189072
12988	1	0	2.42	0.001	-11.24079133	Down	0.53554	0.715400279
214899	4	0	2.42	0.001	-11.24079133	Down	0.074205	0.410272296
20897	1	0	2.41	0.001	-11.23481743	Down	0.53554	0.841750725
223669	1	0	2.41	0.001	-11.23481743	Down	0.53554	0.829683176

20620	1	0	2.4	0.001	-11.22881869	Down	0.53554	0.844942802
76438	1	0	2.4	0.001	-11.22881869	Down	0.53554	0.793064307
66408	2	0	2.4	0.001	-11.22881869	Down	0.277122	0.68041669
14011	2	0	2.4	0.001	-11.22881869	Down	0.277122	0.648097853
231128	2	0	2.4	0.001	-11.22881869	Down	0.277122	0.646100354
214290	2	0	2.4	0.001	-11.22881869	Down	0.277122	0.639293641
54673	1	0	2.39	0.001	-11.2227949	Down	0.53554	0.749197771
381373	1	0	2.39	0.001	-11.2227949	Down	0.53554	0.745937118
50918	1	0	2.39	0.001	-11.2227949	Down	0.53554	0.738152781
240444	1	0	2.39	0.001	-11.2227949	Down	0.53554	0.699674728
192176	3	0	2.39	0.001	-11.2227949	Down	0.1434012	0.524963214
74407	1	0	2.38	0.001	-11.21674586	Down	0.53554	0.837636906
72344	2	0	2.38	0.001	-11.21674586	Down	0.277122	0.689160837
64652	2	0	2.38	0.001	-11.21674586	Down	0.277122	0.663577601
12394	2	0	2.38	0.001	-11.21674586	Down	0.277122	0.660914922
14645	1	0	2.38	0.001	-11.21674586	Down	0.53554	0.714597232
79565	1	0	2.37	0.001	-11.21067134	Down	0.53554	0.836222244
226351	1	0	2.37	0.001	-11.21067134	Down	0.53554	0.759023734
105827	1	0	2.37	0.001	-11.21067134	Down	0.53554	0.723413692
64817	4	0	2.36	0.001	-11.20457114	Down	0.074205	0.433305126
235415	1	0	2.35	0.001	-11.19844504	Down	0.53554	0.844142516
19298	1	0	2.35	0.001	-11.19844504	Down	0.53554	0.814071673
77595	2	0	2.35	0.001	-11.19844504	Down	0.277122	0.661673504
12534	1	0	2.35	0.001	-11.19844504	Down	0.53554	0.720606135
72701	1	0	2.34	0.001	-11.19229281	Down	0.53554	0.805682217
268291	1	0	2.34	0.001	-11.19229281	Down	0.53554	0.780015463

240726	3	0	2.34	0.001	-11.19229281	Down	0.1434012	0.558111031
17164	1	0	2.33	0.001	-11.18611424	Down	0.53554	0.820064104
22388	1	0	2.33	0.001	-11.18611424	Down	0.53554	0.803938001
23830	1	0	2.33	0.001	-11.18611424	Down	0.53554	0.800615943
67006	1	0	2.33	0.001	-11.18611424	Down	0.53554	0.721189244
102323	1	0	2.33	0.001	-11.18611424	Down	0.53554	0.717589105
77582	1	0	2.33	0.001	-11.18611424	Down	0.53554	0.712540508
212880	2	0	2.33	0.001	-11.18611424	Down	0.277122	0.62255188
13511	2	0	2.33	0.001	-11.18611424	Down	0.277122	0.621880303
223693	1	0	2.33	0.001	-11.18611424	Down	0.53554	0.69574213
140476	2	0	2.32	0.001	-11.17990909	Down	0.277122	0.705393748
71733	1	0	2.32	0.001	-11.17990909	Down	0.53554	0.797321227
56347	1	0	2.32	0.001	-11.17990909	Down	0.53554	0.790672168
99889	1	0	2.32	0.001	-11.17990909	Down	0.53554	0.780288625
13476	1	0	2.31	0.001	-11.17367714	Down	0.53554	0.762791568
106347	1	0	2.31	0.001	-11.17367714	Down	0.53554	0.740114323
319901	2	0	2.31	0.001	-11.17367714	Down	0.277122	0.616559402
15239	1	0	2.31	0.001	-11.17367714	Down	0.53554	0.699235578
27225	1	0	2.31	0.001	-11.17367714	Down	0.53554	0.694008463
54608	1	0	2.31	0.001	-11.17367714	Down	0.53554	0.690781017
211499	1	0	2.3	0.001	-11.16741815	Down	0.53554	0.831075781
74442	2	0	2.3	0.001	-11.16741815	Down	0.277122	0.638055385
207704	1	0	2.29	0.001	-11.16113188	Down	0.53554	0.794903379
329278	2	0	2.29	0.001	-11.16113188	Down	0.277122	0.625422338
18685	1	0	2.29	0.001	-11.16113188	Down	0.53554	0.70398552
12774	1	0	2.29	0.001	-11.16113188	Down	0.53554	0.693576395

227210	1	0	2.28	0.001	-11.15481811	Down	0.53554	0.822485851
56055	1	0	2.28	0.001	-11.15481811	Down	0.53554	0.81362577
54125	1	0	2.28	0.001	-11.15481811	Down	0.53554	0.784961835
20147	2	0	2.28	0.001	-11.15481811	Down	0.277122	0.628833423
11932	1	0	2.27	0.001	-11.14847658	Down	0.53554	0.834031132
109349	1	0	2.27	0.001	-11.14847658	Down	0.53554	0.821273192
192185	1	0	2.27	0.001	-11.14847658	Down	0.53554	0.785653798
76960	1	0	2.27	0.001	-11.14847658	Down	0.53554	0.78303081
12589	1	0	2.27	0.001	-11.14847658	Down	0.53554	0.780972369
20399	1	0	2.27	0.001	-11.14847658	Down	0.53554	0.748442785
66092	1	0	2.27	0.001	-11.14847658	Down	0.53554	0.74258096
14107	5	0	2.27	0.001	-11.14847658	Down	0.0383984	0.304878899
235627	3	0	2.27	0.001	-11.14847658	Down	0.1434012	0.524271259
17219	1	0	2.26	0.001	-11.14210706	Down	0.53554	0.840005342
230789	1	0	2.26	0.001	-11.14210706	Down	0.53554	0.771374128
71707	1	0	2.26	0.001	-11.14210706	Down	0.53554	0.752868447
108897	1	0	2.26	0.001	-11.14210706	Down	0.53554	0.706328791
74103	1	0	2.25	0.001	-11.13570929	Down	0.53554	0.821424579
319535	2	0	2.25	0.001	-11.13570929	Down	0.277122	0.673264865
233904	2	0	2.25	0.001	-11.13570929	Down	0.277122	0.649009896
66868	1	0	2.25	0.001	-11.13570929	Down	0.53554	0.741592335
15898	1	0	2.25	0.001	-11.13570929	Down	0.53554	0.722826981
80752	1	0	2.24	0.001	-11.12928302	Down	0.53554	0.777429927
214105	1	0	2.24	0.001	-11.12928302	Down	0.53554	0.705993083
12494	1	0	2.23	0.001	-11.12282799	Down	0.53554	0.794619889
193003	1	0	2.23	0.001	-11.12282799	Down	0.53554	0.768315231

20843	2	0	2.23	0.001	-11.12282799	Down	0.277122	0.627806197
12531	1	0	2.22	0.001	-11.11634396	Down	0.53554	0.776481676
19164	1	0	2.22	0.001	-11.11634396	Down	0.53554	0.770307405
19821	1	0	2.22	0.001	-11.11634396	Down	0.53554	0.767256946
230721	1	0	2.21	0.001	-11.10983065	Down	0.53554	0.841909756
14175	1	0	2.21	0.001	-11.10983065	Down	0.53554	0.717242611
16594	1	0	2.21	0.001	-11.10983065	Down	0.53554	0.708012129
319748	1	0	2.21	0.001	-11.10983065	Down	0.53554	0.698030755
80903	1	0	2.21	0.001	-11.10983065	Down	0.53554	0.695633522
234964	1	0	2.2	0.001	-11.10328781	Down	0.53554	0.813328772
11838	1	0	2.2	0.001	-11.10328781	Down	0.53554	0.731008586
233537	1	0	2.2	0.001	-11.10328781	Down	0.53554	0.714711843
19672	1	0	2.19	0.001	-11.09671515	Down	0.53554	0.78510013
230316	1	0	2.19	0.001	-11.09671515	Down	0.53554	0.734139759
218460	1	0	2.19	0.001	-11.09671515	Down	0.53554	0.712426593
24132	1	0	2.18	0.001	-11.09011242	Down	0.53554	0.818257132
79362	2	0	2.18	0.001	-11.09011242	Down	0.277122	0.694139724
56494	1	0	2.18	0.001	-11.09011242	Down	0.53554	0.782068856
53374	2	0	2.18	0.001	-11.09011242	Down	0.277122	0.651945763
75744	1	0	2.18	0.001	-11.09011242	Down	0.53554	0.735108601
276950	1	0	2.18	0.001	-11.09011242	Down	0.53554	0.727309995
381201	1	0	2.17	0.001	-11.08347933	Down	0.53554	0.800903728
67998	1	0	2.17	0.001	-11.08347933	Down	0.53554	0.722241222
15566	1	0	2.17	0.001	-11.08347933	Down	0.53554	0.701547283
27387	1	0	2.16	0.001	-11.0768156	Down	0.53554	0.827987428
231713	1	0	2.16	0.001	-11.0768156	Down	0.53554	0.777294321

74241	1	0	2.16	0.001	-11.0768156	Down	0.53554	0.771240627
216021	1	0	2.16	0.001	-11.0768156	Down	0.53554	0.76937644
99138	1	0	2.15	0.001	-11.07012094	Down	0.53554	0.807873158
12292	2	0	2.15	0.001	-11.07012094	Down	0.277122	0.689985686
16885	1	0	2.15	0.001	-11.07012094	Down	0.53554	0.776752369
109333	2	0	2.15	0.001	-11.07012094	Down	0.277122	0.658273526
114714	1	0	2.15	0.001	-11.07012094	Down	0.53554	0.694224699
72475	1	0	2.14	0.001	-11.06339508	Down	0.53554	0.714139157
59057	1	0	2.13	0.001	-11.05663772	Down	0.53554	0.836379193
14186	1	0	2.13	0.001	-11.05663772	Down	0.53554	0.696068157
105837	1	0	2.12	0.001	-11.04984855	Down	0.53554	0.796181587
213760	1	0	2.12	0.001	-11.04984855	Down	0.53554	0.762530517
114229	1	0	2.12	0.001	-11.04984855	Down	0.53554	0.734866151
74153	1	0	2.12	0.001	-11.04984855	Down	0.53554	0.733414803
54601	1	0	2.12	0.001	-11.04984855	Down	0.53554	0.695307901
102443350	1	0	2.12	0.001	-11.04984855	Down	0.53554	0.693037067
50528	1	0	2.11	0.001	-11.04302728	Down	0.53554	0.812290984
231637	1	0	2.11	0.001	-11.04302728	Down	0.53554	0.755420976
27373	1	0	2.11	0.001	-11.04302728	Down	0.53554	0.707113351
27007	1	0	2.11	0.001	-11.04302728	Down	0.53554	0.696830077
102657	1	0	2.1	0.001	-11.03617361	Down	0.53554	0.843663071
103841	1	0	2.1	0.001	-11.03617361	Down	0.53554	0.8365362
228994	1	0	2.1	0.001	-11.03617361	Down	0.53554	0.808606122
71795	2	0	2.1	0.001	-11.03617361	Down	0.277122	0.642858144
53791	1	0	2.1	0.001	-11.03617361	Down	0.53554	0.736080003
56489	1	0	2.09	0.001	-11.02928723	Down	0.53554	0.734502776

234733	2.05	0	2.08	0.001	-11.02236781	Down	0.277122	0.710391917
72293	1	0	2.08	0.001	-11.02236781	Down	0.53554	0.793629268
52477	1	0	2.08	0.001	-11.02236781	Down	0.53554	0.755292939
18574	1	0	2.07	0.001	-11.01541505	Down	0.53554	0.789551442
237107	1.5	0	2.07	0.001	-11.01541505	Down	0.53554	0.773785091
217310	1	0	2.07	0.001	-11.01541505	Down	0.53554	0.765674973
12177	1	0	2.07	0.001	-11.01541505	Down	0.53554	0.714368121
214531	1	0	2.07	0.001	-11.01541505	Down	0.53554	0.710042757
101861	1	0	2.06	0.001	-11.00842862	Down	0.53554	0.82829523
171207	1	0	2.06	0.001	-11.00842862	Down	0.53554	0.82172752
21341	1	0	2.06	0.001	-11.00842862	Down	0.53554	0.811847029
72171	1	0	2.06	0.001	-11.00842862	Down	0.53554	0.761097923
630146	1	0	2.06	0.001	-11.00842862	Down	0.53554	0.725533758
229707	1	0	2.06	0.001	-11.00842862	Down	0.53554	0.709929638
70497	1	0	2.05	0.001	-11.00140819	Down	0.53554	0.817806632
70999	1	0	2.05	0.001	-11.00140819	Down	0.53554	0.800040995
16801	1	0	2.05	0.001	-11.00140819	Down	0.53554	0.796323863
74493	2	0	2.05	0.001	-11.00140819	Down	0.277122	0.673068349
76932	1	0	2.05	0.001	-11.00140819	Down	0.53554	0.745562714
209032	2	0	2.04	0.001	-10.99435344	Down	0.277122	0.707558196
53312	1	0	2.04	0.001	-10.99435344	Down	0.53554	0.727191309
12667	1	0	2.04	0.001	-10.99435344	Down	0.53554	0.706216853
319899	2	0	2.03	0.001	-10.98726401	Down	0.277122	0.707341154
21423	1	0	2.03	0.001	-10.98726401	Down	0.53554	0.792359235
14679	1	0	2.03	0.001	-10.98726401	Down	0.53554	0.731608659
232539	2	0	2.03	0.001	-10.98726401	Down	0.277122	0.614915243

213491	1	0	2.03	0.001	-10.98726401	Down		0.53554	0.697921431
56316	1	0	2.02	0.001	-10.98013958	Down		0.53554	0.814220417
213753	1	0	2.02	0.001	-10.98013958	Down		0.53554	0.780425278
269639	1	0	2.02	0.001	-10.98013958	Down		0.53554	0.777158762
269523	1	0	2.02	0.001	-10.98013958	Down		0.53554	0.765017741
16526	1	0	2.02	0.001	-10.98013958	Down		0.53554	0.725651904
83921	2	0	2.01	0.001	-10.97297979	Down		0.277122	0.706041691
319776	1	0	2.01	0.001	-10.97297979	Down		0.53554	0.81931023
108099	1	0	2.01	0.001	-10.97297979	Down		0.53554	0.753759868
210789	2	0	2.01	0.001	-10.97297979	Down		0.277122	0.636119217
327958	2	0	2.01	0.001	-10.97297979	Down		0.277122	0.620040915
68659	2	0	2.01	0.001	-10.97297979	Down		0.277122	0.618709998
54609	1	0	2	0.001	-10.96578428	Down		0.53554	0.75721807
75974	2	0	2	0.001	-10.96578428	Down		0.277122	0.653608889
17268	1	0	2	0.001	-10.96578428	Down		0.53554	0.716781139
212139	1	0	2	0.001	-10.96578428	Down		0.53554	0.710382327
240427	3	0	2	0.001	-10.96578428	Down		0.1434012	0.532934964
17169	1	0	2	0.001	-10.96578428	Down		0.53554	0.70610495
16907	1	0	1.98	0.001	-10.95128471	Down		0.53554	0.815860187
50791	2	0	1.98	0.001	-10.95128471	Down		0.277122	0.69455788
67547	1	0	1.98	0.001	-10.95128471	Down		0.53554	0.803068722
75172	1	0	1.98	0.001	-10.95128471	Down		0.53554	0.791514803
381091	1	0	1.98	0.001	-10.95128471	Down		0.53554	0.78899227
53945	1	0	1.98	0.001	-10.95128471	Down		0.53554	0.694657574
104416	1	0	1.97	0.001	-10.94397991	Down		0.53554	0.81377435
17096	1	0	1.97	0.001	-10.94397991	Down		0.53554	0.786485764

545474	1	0	1.97	0.001	-10.94397991	Down	0.53554	0.753632393
231051	5	0	1.97	0.001	-10.94397991	Down	0.0383984	0.311720084
219151	1	0	1.97	0.001	-10.94397991	Down	0.53554	0.748945939
22422	1	0	1.96	0.001	-10.93663794	Down	0.53554	0.805973655
11790	1	0	1.96	0.001	-10.93663794	Down	0.53554	0.749071834
14904	1	0	1.95	0.001	-10.92925841	Down	0.53554	0.828603261
71718	1	0	1.95	0.001	-10.92925841	Down	0.53554	0.793346687
245468	1	0	1.95	0.001	-10.92925841	Down	0.53554	0.758506951
16543	1	0	1.95	0.001	-10.92925841	Down	0.53554	0.75657527
101359	1	0	1.95	0.001	-10.92925841	Down	0.53554	0.726598458
71729	1	0	1.95	0.001	-10.92925841	Down	0.53554	0.720839266
71740	1	0	1.95	0.001	-10.92925841	Down	0.53554	0.69879698
237403	1	0	1.94	0.001	-10.92184094	Down	0.53554	0.794336602
19777	1	0	1.94	0.001	-10.92184094	Down	0.53554	0.743820454
20349	2	0	1.94	0.001	-10.92184094	Down	0.277122	0.621545057
230971	2	0	1.94	0.001	-10.92184094	Down	0.277122	0.612953791
320974	1	0	1.93	0.001	-10.91438513	Down	0.53554	0.839372451
15505	1	0	1.93	0.001	-10.91438513	Down	0.53554	0.835751752
213827	1	0	1.93	0.001	-10.91438513	Down	0.53554	0.775131038
14360	1	0	1.93	0.001	-10.91438513	Down	0.53554	0.705545969
75273	1	0	1.93	0.001	-10.91438513	Down	0.53554	0.694874215
18949	1	0	1.93	0.001	-10.91438513	Down	0.53554	0.692068386
26390	2	0	1.92	0.001	-10.9068906	Down	0.277122	0.647733753
11804	1	0	1.92	0.001	-10.9068906	Down	0.53554	0.734260725
170935	1	0	1.92	0.001	-10.9068906	Down	0.53554	0.730170136
20874	2	0	1.92	0.001	-10.9068906	Down	0.277122	0.614096448

57745	1	0	1.91	0.001	-10.89935692	Down	0.53554	0.793487952
224897	1	0	1.91	0.001	-10.89935692	Down	0.53554	0.777701281
245902	1	0	1.9	0.001	-10.8917837	Down	0.53554	0.77702325
13207	1	0	1.9	0.001	-10.8917837	Down	0.53554	0.737297872
50753	1	0	1.9	0.001	-10.8917837	Down	0.53554	0.71597499
77110	1	0	1.89	0.001	-10.88417052	Down	0.53554	0.823549869
14733	1	0	1.89	0.001	-10.88417052	Down	0.53554	0.763052798
209692	1	0	1.89	0.001	-10.88417052	Down	0.53554	0.74932375
211673	2	0	1.89	0.001	-10.88417052	Down	0.277122	0.646462619
67996	1	0	1.89	0.001	-10.88417052	Down	0.53554	0.73754193
60597	1	0	1.88	0.001	-10.87651695	Down	0.53554	0.814666973
66660	1	0	1.88	0.001	-10.87651695	Down	0.53554	0.792077558
12790	1	0	1.88	0.001	-10.87651695	Down	0.53554	0.791655417
320225	1	0	1.88	0.001	-10.87651695	Down	0.53554	0.775805769
23872	1	0	1.88	0.001	-10.87651695	Down	0.53554	0.771107171
66867	1	0	1.88	0.001	-10.87651695	Down	0.53554	0.765280498
19714	3	0	1.87	0.001	-10.86882255	Down	0.1434012	0.582637395
12338	1	0	1.87	0.001	-10.86882255	Down	0.53554	0.78857341
118449	2	0	1.87	0.001	-10.86882255	Down	0.277122	0.65602622
21384	1	0	1.87	0.001	-10.86882255	Down	0.53554	0.708575026
12009	1	0	1.87	0.001	-10.86882255	Down	0.53554	0.698577887
72415	1	0	1.86	0.001	-10.86108691	Down	0.53554	0.691746094
16510	2	0	1.85	0.001	-10.85330956	Down	0.277122	0.688954933
208449	1	0	1.85	0.001	-10.85330956	Down	0.53554	0.769110863
22591	1	0	1.84	0.001	-10.84549005	Down	0.53554	0.721656411
13482	1	0	1.83	0.001	-10.83762793	Down	0.53554	0.838109524

331491	1	0	1.83	0.001	-10.83762793	Down	0.53554	0.789131989
13109	1	0	1.83	0.001	-10.83762793	Down	0.53554	0.737053976
70769	1	0	1.83	0.001	-10.83762793	Down	0.53554	0.728498993
74020	1	0	1.82	0.001	-10.82972274	Down	0.53554	0.808752875
66854	1	0	1.82	0.001	-10.82972274	Down	0.53554	0.794053517
19330	1	0	1.82	0.001	-10.82972274	Down	0.53554	0.784823589
69981	1	0	1.82	0.001	-10.82972274	Down	0.53554	0.779742492
66713	1	0	1.82	0.001	-10.82972274	Down	0.53554	0.754397891
74521	1	0	1.82	0.001	-10.82972274	Down	0.53554	0.753122924
218624	1	0	1.82	0.001	-10.82972274	Down	0.53554	0.732209717
242864	1	0	1.82	0.001	-10.82972274	Down	0.53554	0.715744995
23802	1	0	1.81	0.001	-10.82177398	Down	0.53554	0.817206738
17957	1	0	1.81	0.001	-10.82177398	Down	0.53554	0.763444979
68054	1	0	1.81	0.001	-10.82177398	Down	0.53554	0.724119002
13608	1	0	1.81	0.001	-10.82177398	Down	0.53554	0.723296273
16649	1	0	1.8	0.001	-10.81378119	Down	0.53554	0.816458106
11928	1	0	1.8	0.001	-10.81378119	Down	0.53554	0.800184654
74016	1	0	1.79	0.001	-10.80574387	Down	0.53554	0.815412322
227619	1	0	1.79	0.001	-10.80574387	Down	0.53554	0.787319495
67198	1	0	1.79	0.001	-10.80574387	Down	0.53554	0.733294116
69219	1	0	1.79	0.001	-10.80574387	Down	0.53554	0.712768448
20218	1	0	1.78	0.001	-10.79766153	Down	0.53554	0.805099971
14190	1	0	1.78	0.001	-10.79766153	Down	0.53554	0.803213472
233328	2	0	1.78	0.001	-10.79766153	Down	0.277122	0.687927256
74043	1	0	1.77	0.001	-10.78953364	Down	0.53554	0.809487437
14282	1	0	1.77	0.001	-10.78953364	Down	0.53554	0.753377572

209462	1	0	1.77	0.001	-10.78953364	Down	0.53554	0.690246025
225164	1	0	1.76	0.001	-10.78135971	Down	0.53554	0.837007577
23988	1	0	1.76	0.001	-10.78135971	Down	0.53554	0.70767482
226922	2	0	1.75	0.001	-10.77313921	Down	0.277122	0.690605619
19418	2	0	1.75	0.001	-10.77313921	Down	0.277122	0.682430353
209645	1	0	1.75	0.001	-10.77313921	Down	0.53554	0.785238474
52123	1	0	1.75	0.001	-10.77313921	Down	0.53554	0.780698728
67842	1	0	1.75	0.001	-10.77313921	Down	0.53554	0.768447722
17916	1	0	1.75	0.001	-10.77313921	Down	0.53554	0.74856851
15184	1	0	1.75	0.001	-10.77313921	Down	0.53554	0.747563889
53604	1	0	1.74	0.001	-10.76487159	Down	0.53554	0.731848964
269717	1	0	1.74	0.001	-10.76487159	Down	0.53554	0.713453144
97212	1	0	1.74	0.001	-10.76487159	Down	0.53554	0.70778722
71567	1	0	1.73	0.001	-10.75655632	Down	0.53554	0.840163714
12978	1	0	1.73	0.001	-10.75655632	Down	0.53554	0.819762388
70012	1	0	1.73	0.001	-10.75655632	Down	0.53554	0.782480832
20558	1	0	1.73	0.001	-10.75655632	Down	0.53554	0.754142552
78913	2	0	1.72	0.001	-10.74819285	Down	0.277122	0.7036717
226178	1	0	1.72	0.001	-10.74819285	Down	0.53554	0.807141521
107684	1	0	1.72	0.001	-10.74819285	Down	0.53554	0.745437996
14229	1	0	1.72	0.001	-10.74819285	Down	0.53554	0.714482658
242667	1	0	1.72	0.001	-10.74819285	Down	0.53554	0.700004452
76088	2	0	1.71	0.001	-10.73978061	Down	0.277122	0.71082989
106957	1	0	1.71	0.001	-10.73978061	Down	0.53554	0.834655992
319765	1	0	1.71	0.001	-10.73978061	Down	0.53554	0.825992278
66412	1	0	1.71	0.001	-10.73978061	Down	0.53554	0.715170653

241576	1	0	1.71	0.001	-10.73978061	Down	0.53554	0.691853492
234356	1	0	1.7	0.001	-10.73131903	Down	0.53554	0.744690565
16439	3	0	1.7	0.001	-10.73131903	Down	0.1434012	0.532221849
269994	1	0	1.7	0.001	-10.73131903	Down	0.53554	0.70431932
59033	3	0	1.69	0.001	-10.72280753	Down	0.1434012	0.591885608
218440	1	0	1.69	0.001	-10.72280753	Down	0.53554	0.716435424
242083	1	0	1.69	0.001	-10.72280753	Down	0.53554	0.690995246
268977	2	0	1.68	0.001	-10.71424552	Down	0.277122	0.659214455
27418	1	0	1.68	0.001	-10.71424552	Down	0.53554	0.742828528
80911	1	0	1.68	0.001	-10.71424552	Down	0.53554	0.717704677
67722	1	0	1.68	0.001	-10.71424552	Down	0.53554	0.704096751
99526	1	0	1.67	0.001	-10.70563239	Down	0.53554	0.782618254
16878	1	0	1.67	0.001	-10.70563239	Down	0.53554	0.761357994
16780	1	0	1.67	0.001	-10.70563239	Down	0.53554	0.741962761
67974	1	0	1.67	0.001	-10.70563239	Down	0.53554	0.71049559
69116	4	0	1.67	0.001	-10.70563239	Down	0.074205	0.408913778
217653	1	0	1.66	0.001	-10.69696753	Down	0.53554	0.816757394
13032	1	0	1.66	0.001	-10.69696753	Down	0.53554	0.813031991
320119	1	0	1.66	0.001	-10.69696753	Down	0.53554	0.71482649
140557	1	0	1.65	0.001	-10.68825031	Down	0.53554	0.757861963
17765	1	0	1.65	0.001	-10.68825031	Down	0.53554	0.733656296
68964	1	0	1.65	0.001	-10.68825031	Down	0.53554	0.731488565
235339	1	0	1.65	0.001	-10.68825031	Down	0.53554	0.692175884
235582	1	0	1.64	0.001	-10.6794801	Down	0.53554	0.799897386
242687	1	0	1.64	0.001	-10.6794801	Down	0.53554	0.740237266
408065	1	0	1.64	0.001	-10.6794801	Down	0.53554	0.702099943

19683	0.78	0	1.63	0.001	-10.67065625	Down	0.96507	0.972079345
70099	1	0	1.63	0.001	-10.67065625	Down	0.53554	0.834187259
104175	1	0	1.63	0.001	-10.67065625	Down	0.53554	0.817506575
80292	1	0	1.62	0.001	-10.6617781	Down	0.53554	0.814369214
20467	1	0	1.62	0.001	-10.6617781	Down	0.53554	0.799466871
71902	2	0	1.62	0.001	-10.6617781	Down	0.277122	0.667032734
241627	1	0	1.62	0.001	-10.6617781	Down	0.53554	0.749449771
23887	1	0	1.62	0.001	-10.6617781	Down	0.53554	0.742704723
235623	1	0	1.62	0.001	-10.6617781	Down	0.53554	0.726361588
67712	1	0	1.61	0.001	-10.65284497	Down	0.53554	0.834968773
108857	2	0	1.61	0.001	-10.65284497	Down	0.277122	0.685473294
50997	1	0	1.61	0.001	-10.65284497	Down	0.53554	0.782893243
68519	1	0	1.61	0.001	-10.65284497	Down	0.53554	0.710155911
320209	1	0	1.6	0.001	-10.64385619	Down	0.53554	0.705434279
13194	1	0	1.6	0.001	-10.64385619	Down	0.53554	0.696939058
19335	1	0	1.59	0.001	-10.63481105	Down	0.53554	0.838582676
72349	1	0	1.59	0.001	-10.63481105	Down	0.53554	0.725179551
70598	1	0	1.59	0.001	-10.63481105	Down	0.53554	0.699345314
19417	1	0	1.58	0.001	-10.62570884	Down	0.53554	0.824921944
74427	1	0	1.58	0.001	-10.62570884	Down	0.53554	0.784409143
56453	1	0	1.58	0.001	-10.62570884	Down	0.53554	0.774188384
99512	1	0	1.58	0.001	-10.62570884	Down	0.53554	0.714253621
320007	1	0	1.57	0.001	-10.61654884	Down	0.53554	0.82614541
269855	1	0	1.57	0.001	-10.61654884	Down	0.53554	0.815263143
93704	1.06	0	1.57	0.001	-10.61654884	Down	0.53554	0.738275073
108829	2	0	1.57	0.001	-10.61654884	Down	0.277122	0.640003376

78771	1	0	1.56	0.001	-10.60733031	Down		0.53554	0.816159037
108058	1	0	1.56	0.001	-10.60733031	Down		0.53554	0.726243211
70650	1	0	1.56	0.001	-10.60733031	Down		0.53554	0.708687713
211548	1	0	1.56	0.001	-10.60733031	Down		0.53554	0.702875132
231659	2	0	1.56	0.001	-10.60733031	Down		0.277122	0.615243373
101943	1	0	1.55	0.001	-10.5980525	Down		0.53554	0.802924025
238386	1	0	1.55	0.001	-10.5980525	Down		0.53554	0.753887386
235611	2	0	1.55	0.001	-10.5980525	Down		0.277122	0.607463689
229776	1	0	1.54	0.001	-10.58871464	Down		0.53554	0.787180417
14115	1	0	1.54	0.001	-10.58871464	Down		0.53554	0.772176112
235469	1	0	1.54	0.001	-10.58871464	Down		0.53554	0.76488643
11764	1	0	1.54	0.001	-10.58871464	Down		0.53554	0.726124872
329941	1	0	1.54	0.001	-10.58871464	Down		0.53554	0.692606208
109264	1	0	1.53	0.001	-10.57931594	Down		0.53554	0.825533223
74239	1	0	1.53	0.001	-10.57931594	Down		0.53554	0.76266102
227648	2	0	1.52	0.001	-10.56985561	Down		0.277122	0.698555638
14235	1	0	1.52	0.001	-10.56985561	Down		0.53554	0.813477244
17126	2	0	1.52	0.001	-10.56985561	Down		0.277122	0.690192207
380768	1	0	1.52	0.001	-10.56985561	Down		0.53554	0.794478221
116940	1	0	1.52	0.001	-10.56985561	Down		0.53554	0.729094951
111173	2	0	1.52	0.001	-10.56985561	Down		0.277122	0.625252755
228980	1	0	1.51	0.001	-10.56033283	Down		0.53554	0.827218923
12419	2	0	1.51	0.001	-10.56033283	Down		0.277122	0.704101424
57385	1	0	1.51	0.001	-10.56033283	Down		0.53554	0.791374239
101613	1	0	1.51	0.001	-10.56033283	Down		0.53554	0.760059413
76367	1	0	1.51	0.001	-10.56033283	Down		0.53554	0.759153039

16897	1	0	1.51	0.001	-10.56033283	Down	0.53554	0.727666287
12550	1	0	1.5	0.001	-10.55074679	Down	0.53554	0.830456269
20362	1	0	1.5	0.001	-10.55074679	Down	0.53554	0.822789575
378431	1	0	1.5	0.001	-10.55074679	Down	0.53554	0.81199496
18759	1	0	1.5	0.001	-10.55074679	Down	0.53554	0.803503127
108686	2	0	1.5	0.001	-10.55074679	Down	0.277122	0.671891656
14084	1	0	1.5	0.001	-10.55074679	Down	0.53554	0.709364588
216835	1	0	1.5	0.001	-10.55074679	Down	0.53554	0.698140113
209760	1	0	1.49	0.001	-10.54109662	Down	0.53554	0.829220011
22063	1	0	1.49	0.001	-10.54109662	Down	0.53554	0.808899681
20848	1	0	1.49	0.001	-10.54109662	Down	0.53554	0.790531903
19134	1	0	1.49	0.001	-10.54109662	Down	0.53554	0.76200895
67269	1	0	1.49	0.001	-10.54109662	Down	0.53554	0.755164945
70693	1	0	1.49	0.001	-10.54109662	Down	0.53554	0.725888311
13666	1	0	1.48	0.001	-10.53138146	Down	0.53554	0.845744608
72117	1	0	1.48	0.001	-10.53138146	Down	0.53554	0.786624597
328833	1	0	1.48	0.001	-10.53138146	Down	0.53554	0.736566668
65111	1	0	1.47	0.001	-10.52160044	Down	0.53554	0.823245583
76895	1	0	1.47	0.001	-10.52160044	Down	0.53554	0.797463912
66864	1	0	1.47	0.001	-10.52160044	Down	0.53554	0.762400058
107476	2	0	1.47	0.001	-10.52160044	Down	0.277122	0.638939363
20357	1	0	1.47	0.001	-10.52160044	Down	0.53554	0.716665864
14000	1	0	1.46	0.001	-10.51175265	Down	0.53554	0.830611061
20788	1	0	1.46	0.001	-10.51175265	Down	0.53554	0.810076048
217365	1	0	1.46	0.001	-10.51175265	Down	0.53554	0.778652514
27377	1	0	1.46	0.001	-10.51175265	Down	0.53554	0.707450125

70572	1	0	1.45	0.001	-10.50183718	Down	0.53554	0.837479485
74002	1	0	1.45	0.001	-10.50183718	Down	0.53554	0.767653461
12874	2	0	1.45	0.001	-10.50183718	Down	0.277122	0.650658059
101772	1	0	1.45	0.001	-10.50183718	Down	0.53554	0.74531332
13211	1	0	1.45	0.001	-10.50183718	Down	0.53554	0.703207881
244329	1	0	1.44	0.001	-10.4918531	Down	0.53554	0.827065393
11772	1	0	1.44	0.001	-10.4918531	Down	0.53554	0.826298598
102093	1	0	1.44	0.001	-10.4918531	Down	0.53554	0.784271091
19645	1	0	1.44	0.001	-10.4918531	Down	0.53554	0.757604274
56711	1	0	1.44	0.001	-10.4918531	Down	0.53554	0.730768832
71148	1	0	1.44	0.001	-10.4918531	Down	0.53554	0.709251686
26420	1	0	1.43	0.001	-10.48179943	Down	0.53554	0.746436908
21859	1	0	1.43	0.001	-10.48179943	Down	0.53554	0.733052861
17119	1	0	1.43	0.001	-10.48179943	Down	0.53554	0.71265446
215632	1	0	1.42	0.001	-10.47167521	Down	0.53554	0.733897948
246086	1	0	1.42	0.001	-10.47167521	Down	0.53554	0.724589974
52036	1	0	1.42	0.001	-10.47167521	Down	0.53554	0.69110241
71472	1	0	1.41	0.001	-10.46147945	Down	0.53554	0.802634787
13058	1	0	1.41	0.001	-10.46147945	Down	0.53554	0.735958438
24131	1	0	1.41	0.001	-10.46147945	Down	0.53554	0.732932293
230806	1	0	1.4	0.001	-10.45121111	Down	0.53554	0.797036011
75991	1	0	1.4	0.001	-10.45121111	Down	0.53554	0.786763478
13026	1	0	1.4	0.001	-10.45121111	Down	0.53554	0.763706656
271849	1	0	1.39	0.001	-10.44086917	Down	0.53554	0.771641271
74388	1	0	1.39	0.001	-10.44086917	Down	0.53554	0.743076261
16531	1	0	1.39	0.001	-10.44086917	Down	0.53554	0.718862452

57915	1	0	1.39	0.001	-10.44086917	Down	0.53554	0.704430658
26362	1	0	1.39	0.001	-10.44086917	Down	0.53554	0.694549305
17196	1	0	1.39	0.001	-10.44086917	Down	0.53554	0.691424102
107508	1	0	1.38	0.001	-10.43045255	Down	0.53554	0.810960571
74048	1	0	1.38	0.001	-10.43045255	Down	0.53554	0.733777102
320181	1	0	1.37	0.001	-10.41996018	Down	0.53554	0.843343743
268780	1	0	1.37	0.001	-10.41996018	Down	0.53554	0.840322146
67890	1	0	1.37	0.001	-10.41996018	Down	0.53554	0.813922984
320244	1	0	1.37	0.001	-10.41996018	Down	0.53554	0.783857228
11834	1	0	1.36	0.001	-10.40939094	Down	0.53554	0.75954122
103573	1	0	1.36	0.001	-10.40939094	Down	0.53554	0.743944631
106794	1	0	1.35	0.001	-10.39874369	Down	0.53554	0.764755164
71947	1	0	1.34	0.001	-10.38801729	Down	0.53554	0.830301535
208691	1	0	1.34	0.001	-10.38801729	Down	0.53554	0.816009584
109205	1	0	1.34	0.001	-10.38801729	Down	0.53554	0.705322624
76408	1	0	1.33	0.001	-10.37721053	Down	0.53554	0.825686185
72584	1	0	1.33	0.001	-10.37721053	Down	0.53554	0.790251523
57257	1	0	1.33	0.001	-10.37721053	Down	0.53554	0.734987356
60611	1	0	1.33	0.001	-10.37721053	Down	0.53554	0.718746506
20564	1	0	1.33	0.001	-10.37721053	Down	0.53554	0.717473569
330788	1.14	0	1.32	0.001	-10.36632221	Down	0.53554	0.826605146
53902	1	0	1.32	0.001	-10.36632221	Down	0.53554	0.754014948
52357	1	0	1.32	0.001	-10.36632221	Down	0.53554	0.731728791
279653	2	0	1.31	0.001	-10.3553511	Down	0.277122	0.711707457
235633	1	0	1.31	0.001	-10.3553511	Down	0.53554	0.741222279
78177	1	0	1.31	0.001	-10.3553511	Down	0.53554	0.737664019

235439	3	0	1.31	0.001	-10.3553511	Down	0.1434012	0.544113719
223770	1	0	1.3	0.001	-10.34429591	Down	0.53554	0.743572224
71175	2	0	1.3	0.001	-10.34429591	Down	0.277122	0.635943784
16190	1	0	1.3	0.001	-10.34429591	Down	0.53554	0.707001165
16782	1	0	1.29	0.001	-10.33315535	Down	0.53554	0.832473069
233977	1	0	1.29	0.001	-10.33315535	Down	0.53554	0.731248497
52837	1	0	1.29	0.001	-10.33315535	Down	0.53554	0.695850771
107581	1	0	1.28	0.001	-10.32192809	Down	0.53554	0.778788595
50523	1	0	1.28	0.001	-10.32192809	Down	0.53554	0.773919476
270086	1	0	1.28	0.001	-10.32192809	Down	0.53554	0.767918032
216860	1	0	1.28	0.001	-10.32192809	Down	0.53554	0.752233008
74340	1	0	1.28	0.001	-10.32192809	Down	0.53554	0.748820087
18854	1	0	1.27	0.001	-10.31061278	Down	0.53554	0.815561556
68802	1	0	1.27	0.001	-10.31061278	Down	0.53554	0.698249505
66313	1	0	1.25	0.001	-10.28771238	Down	0.53554	0.82952873
74488	1	0	1.25	0.001	-10.28771238	Down	0.53554	0.807434017
17929	1	0	1.25	0.001	-10.28771238	Down	0.53554	0.774726763
17533	1	0	1.25	0.001	-10.28771238	Down	0.53554	0.758119826
109079	1	0	1.25	0.001	-10.28771238	Down	0.53554	0.751979133
195727	1	0	1.25	0.001	-10.28771238	Down	0.53554	0.699784601
268515	2	0	1.24	0.001	-10.27612441	Down	0.277122	0.7084277
110616	1	0	1.24	0.001	-10.27612441	Down	0.53554	0.803358273
12123	1	0	1.24	0.001	-10.27612441	Down	0.53554	0.800328366
243842	1	0	1.24	0.001	-10.27612441	Down	0.53554	0.792923192
104831	1	0	1.24	0.001	-10.27612441	Down	0.53554	0.717358071
19250	2	0	1.24	0.001	-10.27612441	Down	0.277122	0.61887605

83997	1	0	1.24	0.001	-10.27612441	Down	0.53554	0.69639449
66074	2	0	1.23	0.001	-10.2644426	Down	0.277122	0.643396251
14245	1	0	1.22	0.001	-10.25266543	Down	0.53554	0.804083064
20250	1	0	1.22	0.001	-10.25266543	Down	0.53554	0.72921426
54667	1	0	1.22	0.001	-10.25266543	Down	0.53554	0.721422752
12928	1	0	1.22	0.001	-10.25266543	Down	0.53554	0.718283098
15277	1	0	1.21	0.001	-10.24079133	Down	0.53554	0.832006785
11856	1	0	1.21	0.001	-10.24079133	Down	0.53554	0.790812483
17999	1	0	1.21	0.001	-10.24079133	Down	0.53554	0.744566139
244895	2	0	1.21	0.001	-10.24079133	Down	0.277122	0.639825794
215008	2	0	1.2	0.001	-10.22881869	Down	0.277122	0.66955057
18124	1	0	1.2	0.001	-10.22881869	Down	0.53554	0.739991421
73122	1	0	1.2	0.001	-10.22881869	Down	0.53554	0.732691276
233532	2	0	1.19	0.001	-10.21674586	Down	0.277122	0.686493648
53601	1	0	1.19	0.001	-10.21674586	Down	0.53554	0.78015202
216439	1	0	1.19	0.001	-10.21674586	Down	0.53554	0.760448522
26419	1	0	1.19	0.001	-10.21674586	Down	0.53554	0.749575835
16554	1	0	1.19	0.001	-10.21674586	Down	0.53554	0.729930932
52028	1	0	1.18	0.001	-10.20457114	Down	0.53554	0.845584125
70144	1	0	1.18	0.001	-10.20457114	Down	0.53554	0.744815033
74513	1	0	1.18	0.001	-10.20457114	Down	0.53554	0.743696318
226252	1	0	1.18	0.001	-10.20457114	Down	0.53554	0.742333557
105722	1	0	1.18	0.001	-10.20457114	Down	0.53554	0.718514728
108837	1	0	1.17	0.001	-10.19229281	Down	0.53554	0.80277938
74352	1	0	1.17	0.001	-10.19229281	Down	0.53554	0.773650753
17776	1	0	1.17	0.001	-10.19229281	Down	0.53554	0.729452994

74302	1	0	1.17	0.001	-10.19229281	Down	0.53554	0.72671695
208618	1	0	1.17	0.001	-10.19229281	Down	0.53554	0.719094455
26409	1	0	1.17	0.001	-10.19229281	Down	0.53554	0.693360563
269180	1	0	1.16	0.001	-10.17990909	Down	0.53554	0.842705813
224105	1	0	1.16	0.001	-10.17990909	Down	0.53554	0.840956471
544963	1	0	1.16	0.001	-10.17990909	Down	0.53554	0.791233725
14260	2	0	1.15	0.001	-10.16741815	Down	0.277122	0.704531672
223435	2	0	1.15	0.001	-10.16741815	Down	0.277122	0.667612091
68655	1	0	1.14	0.001	-10.15481811	Down	0.53554	0.82983768
107746	1	0	1.14	0.001	-10.15481811	Down	0.53554	0.808312777
16494	1	0	1.14	0.001	-10.15481811	Down	0.53554	0.75083881
14020	1	0	1.14	0.001	-10.15481811	Down	0.53554	0.750206791
192193	1	0	1.14	0.001	-10.15481811	Down	0.53554	0.74320019
24046	1	0	1.14	0.001	-10.15481811	Down	0.53554	0.703763162
21825	1	0	1.14	0.001	-10.15481811	Down	0.53554	0.700334487
22289	1	0	1.13	0.001	-10.14210706	Down	0.53554	0.839688777
12816	2	0	1.13	0.001	-10.14210706	Down	0.277122	0.699191074
21826	1	0	1.13	0.001	-10.14210706	Down	0.53554	0.779606078
208922	1	0	1.13	0.001	-10.14210706	Down	0.53554	0.758377866
226251	1	0	1.12	0.001	-10.12928302	Down	0.53554	0.836065355
22284	2	0	1.12	0.001	-10.12928302	Down	0.277122	0.693930834
67016	1	0	1.12	0.001	-10.12928302	Down	0.53554	0.775265891
17540	1	0	1.12	0.001	-10.12928302	Down	0.53554	0.745812274
76469	2	0	1.12	0.001	-10.12928302	Down	0.277122	0.644475171
66691	1	0	1.12	0.001	-10.12928302	Down	0.53554	0.716205133
17888	1	0	1.11	0.001	-10.11634396	Down	0.53554	0.804518567

54710	1	0	1.11	0.001	-10.11634396	Down	0.53554	0.767124865
15568	1	0	1.11	0.001	-10.11634396	Down	0.53554	0.722592564
233332	1	0	1.1	0.001	-10.10328781	Down	0.53554	0.763575795
16775	1	0	1.1	0.001	-10.10328781	Down	0.53554	0.760967954
21814	1	0	1.1	0.001	-10.10328781	Down	0.53554	0.753250227
11864	1	0	1.1	0.001	-10.10328781	Down	0.53554	0.752487055
230861	1	0	1.1	0.001	-10.10328781	Down	0.53554	0.722944247
20928	1.21	0	1.1	0.001	-10.10328781	Down	0.53554	0.706776898
140630	1	0	1.1	0.001	-10.10328781	Down	0.53554	0.700554683
319974	1	0	1.1	0.001	-10.10328781	Down	0.53554	0.693252698
12663	1	0	1.09	0.001	-10.09011242	Down	0.53554	0.845423703
76901	1	0	1.09	0.001	-10.09011242	Down	0.53554	0.747940305
67300	1	0	1.08	0.001	-10.0768156	Down	0.53554	0.736810241
223455	1	0	1.07	0.001	-10.06339508	Down	0.53554	0.705211005
226089	1	0	1.06	0.001	-10.04984855	Down	0.53554	0.820819366
70827	1	0	1.06	0.001	-10.04984855	Down	0.53554	0.742209917
67991	1	0	1.05	0.001	-10.03617361	Down	0.53554	0.8407978
329015	1	0	1.05	0.001	-10.03617361	Down	0.53554	0.831696219
545622	1	0	1.05	0.001	-10.03617361	Down	0.53554	0.797606648
330355	2	0	1.05	0.001	-10.03617361	Down	0.277122	0.684252867
54387	1	0	1.04	0.001	-10.02236781	Down	0.53554	0.828911522
109181	1	0	1.03	0.001	-10.00842862	Down	0.53554	0.828757363
11783	1	0	1.03	0.001	-10.00842862	Down	0.53554	0.792641113
668212	1	0	1.02	0.001	-9.994353437	Down	0.53554	0.762922161
75786	1	0	1.02	0.001	-9.994353437	Down	0.53554	0.75236001
70797	1	0	1.02	0.001	-9.994353437	Down	0.53554	0.71402473

20496	1	0	1.02	0.001	-9.994353437	Down	0.53554	0.701989342
666048	1	0	1.01	0.001	-9.980139578	Down	0.53554	0.833875064
70292	1	0	1.01	0.001	-9.980139578	Down	0.53554	0.765411944
320165	1	0	1.01	0.001	-9.980139578	Down	0.53554	0.758894472
226255	1	0	1.01	0.001	-9.980139578	Down	0.53554	0.752106049
219105	0.8	0	1.01	0.001	-9.980139578	Down	0.96507	0.97020025
110880	1	0	1.01	0.001	-9.980139578	Down	0.53554	0.6915314
78937	1	0	1	0.001	-9.965784285	Down	0.53554	0.824159116
94216	1	0	1	0.001	-9.965784285	Down	0.53554	0.745687473
17919	1	0	1	0.001	-9.965784285	Down	0.53554	0.731368511
27397	1	0	0.99	0.001	-9.951284715	Down	0.53554	0.7888526
320707	1	0	0.99	0.001	-9.951284715	Down	0.53554	0.739132251
70354	1	0	0.98	0.001	-9.936637939	Down	0.53554	0.720023968
18080	1	0	0.98	0.001	-9.936637939	Down	0.53554	0.713224766
226151	1	0	0.97	0.001	-9.921840937	Down	0.53554	0.841115202
72672	1	0	0.97	0.001	-9.921840937	Down	0.53554	0.813180354
27428	1	0	0.97	0.001	-9.921840937	Down	0.53554	0.789691359
56013	1	0	0.96	0.001	-9.906890596	Down	0.53554	0.82385438
19364	1	0	0.96	0.001	-9.906890596	Down	0.53554	0.788294417
26401	1	0	0.96	0.001	-9.906890596	Down	0.53554	0.772845706
668303	1	0	0.96	0.001	-9.906890596	Down	0.53554	0.765938182
27494	1	0	0.96	0.001	-9.906890596	Down	0.53554	0.748065862
18846	1	0	0.96	0.001	-9.906890596	Down	0.53554	0.74134559
67448	1	0	0.96	0.001	-9.906890596	Down	0.53554	0.723766175
545389	1	0	0.96	0.001	-9.906890596	Down	0.53554	0.706889013
14367	1	0	0.96	0.001	-9.906890596	Down	0.53554	0.691960922

18212	1	0	0.95	0.001	-9.891783703	Down	0.53554	0.750585875
14616	1	0	0.95	0.001	-9.891783703	Down	0.53554	0.739622961
17925	1	0	0.94	0.001	-9.876516947	Down	0.53554	0.804373347
109689	1	0	0.94	0.001	-9.876516947	Down	0.53554	0.793205472
226101	1	0	0.94	0.001	-9.876516947	Down	0.53554	0.768182786
18607	1	0	0.94	0.001	-9.876516947	Down	0.53554	0.765149097
17933	1	0	0.94	0.001	-9.876516947	Down	0.53554	0.746186929
70097	1	0	0.93	0.001	-9.861086906	Down	0.53554	0.760578314
108937	1	0	0.93	0.001	-9.861086906	Down	0.53554	0.732450417
225876	1	0	0.92	0.001	-9.845490051	Down	0.53554	0.742457238
77531	1	0	0.92	0.001	-9.845490051	Down	0.53554	0.730050514
18007	1	0	0.91	0.001	-9.829722735	Down	0.53554	0.83984703
11595	1	0	0.91	0.001	-9.829722735	Down	0.53554	0.839530584
28240	1	0	0.91	0.001	-9.829722735	Down	0.53554	0.79603936
16975	1	0	0.91	0.001	-9.829722735	Down	0.53554	0.752995664
73945	1	0	0.91	0.001	-9.829722735	Down	0.53554	0.716550626
11603	1	0	0.9	0.001	-9.813781191	Down	0.53554	0.817056901
77480	1	0	0.9	0.001	-9.813781191	Down	0.53554	0.694116564
319636	1	0	0.89	0.001	-9.797661526	Down	0.53554	0.832162155
217692	1	0	0.89	0.001	-9.797661526	Down	0.53554	0.771774912
12914	1	0	0.89	0.001	-9.797661526	Down	0.53554	0.76699283
622675	1	0	0.87	0.001	-9.764871591	Down	0.53554	0.791093261
11515	1	0	0.87	0.001	-9.764871591	Down	0.53554	0.767785724
373864	1	0	0.87	0.001	-9.764871591	Down	0.53554	0.747689319
76707	1	0	0.87	0.001	-9.764871591	Down	0.53554	0.744939542
19775	1	0	0.87	0.001	-9.764871591	Down	0.53554	0.728737259

218333	1	0	0.87	0.001	-9.764871591	Down	0.53554	0.715055895
20192	2	0	0.86	0.001	-9.74819285	Down	0.277122	0.623899394
74326	1	0	0.85	0.001	-9.731319031	Down	0.53554	0.798750375
243548	1	0	0.85	0.001	-9.731319031	Down	0.53554	0.798464135
12035	1	0	0.85	0.001	-9.731319031	Down	0.53554	0.694982586
15165	1	0	0.84	0.001	-9.714245518	Down	0.53554	0.776616999
17910	1	0	0.84	0.001	-9.714245518	Down	0.53554	0.72742872
12648	1	0	0.84	0.001	-9.714245518	Down	0.53554	0.692929302
74570	1	0	0.83	0.001	-9.696967526	Down	0.53554	0.805390989
239528	1	0	0.83	0.001	-9.696967526	Down	0.53554	0.724354412
68795	1	0	0.82	0.001	-9.6794801	Down	0.53554	0.761748434
66725	1	0	0.81	0.001	-9.661778098	Down	0.53554	0.804228179
11906	2	0	0.81	0.001	-9.661778098	Down	0.277122	0.660536282
329260	1	0	0.81	0.001	-9.661778098	Down	0.53554	0.723648642
20742	1	0	0.81	0.001	-9.661778098	Down	0.53554	0.704987872
171463	1	0	0.81	0.001	-9.661778098	Down	0.53554	0.70110578
12361	1	0	0.81	0.001	-9.661778098	Down	0.53554	0.699455084
235626	1	0	0.8	0.001	-9.64385619	Down	0.53554	0.77756558
245622	1	0	0.8	0.001	-9.64385619	Down	0.53554	0.751471895
228850	1	0	0.79	0.001	-9.625708843	Down	0.53554	0.750965342
232906	1	0	0.79	0.001	-9.625708843	Down	0.53554	0.700885237
231464	1	0	0.78	0.001	-9.607330314	Down	0.53554	0.82400672
100705	1	0	0.78	0.001	-9.607330314	Down	0.53554	0.698687416
217351	1	0	0.76	0.001	-9.569855608	Down	0.53554	0.806995353
381695	1	0	0.74	0.001	-9.531381461	Down	0.53554	0.821879074
16485	1	0	0.74	0.001	-9.531381461	Down	0.53554	0.81810691

237860	1	0	0.74	0.001	-9.531381461	Down	0.53554	0.754525625
17222	1	0	0.74	0.001	-9.531381461	Down	0.53554	0.741468942
330409	1	0	0.72	0.001	-9.491853096	Down	0.53554	0.795470964
234353	1	0	0.72	0.001	-9.491853096	Down	0.53554	0.772309938
18027	1	0	0.72	0.001	-9.491853096	Down	0.53554	0.74982809
22253	1	0	0.72	0.001	-9.491853096	Down	0.53554	0.720256722
214133	1	0	0.72	0.001	-9.491853096	Down	0.53554	0.719210513
194401	1	0	0.7	0.001	-9.451211112	Down	0.53554	0.812142945
74055	1	0	0.7	0.001	-9.451211112	Down	0.53554	0.787458622
381626	1	0	0.7	0.001	-9.451211112	Down	0.53554	0.703652035
269774	2	0	0.69	0.001	-9.430452552	Down	0.277122	0.612302751
70675	1	0	0.68	0.001	-9.409390936	Down	0.53554	0.832628614
192119	1	0	0.68	0.001	-9.409390936	Down	0.53554	0.774053907
16438	1	0	0.67	0.001	-9.388017285	Down	0.53554	0.782755725
16561	1	0	0.66	0.001	-9.366322214	Down	0.53554	0.837322123
11303	1	0	0.65	0.001	-9.344295908	Down	0.53554	0.819009068
76559	1	0	0.65	0.001	-9.344295908	Down	0.53554	0.762269644
93736	1	0	0.65	0.001	-9.344295908	Down	0.53554	0.696503335
68564	1	0	0.64	0.001	-9.321928095	Down	0.53554	0.787041388
18129	1	0	0.63	0.001	-9.299208018	Down	0.53554	0.758248824
26405	1	0	0.62	0.001	-9.276124405	Down	0.53554	0.845103042
83946	1	0	0.62	0.001	-9.276124405	Down	0.53554	0.839214377
320365	1	0	0.62	0.001	-9.276124405	Down	0.53554	0.832317583
20356	1	0	0.62	0.001	-9.276124405	Down	0.53554	0.759800228
20511	1	0	0.6	0.001	-9.22881869	Down	0.53554	0.79660857
12168	1	0	0.6	0.001	-9.22881869	Down	0.53554	0.789271757

16776	1	0	0.58	0.001	-9.17990909	Down	0.53554	0.715859974
241431	1	0	0.56	0.001	-9.129283017	Down	0.53554	0.756703743
11920	1	0	0.56	0.001	-9.129283017	Down	0.53554	0.712996534
432530	1	0	0.54	0.001	-9.076815597	Down	0.53554	0.705769455
207806	1	0	0.53	0.001	-9.049848549	Down	0.53554	0.799180118
110084	1	0	0.52	0.001	-9.022367813	Down	0.53554	0.691209608
225631	1	0	0.5	0.001	-8.965784285	Down	0.53554	0.81110818
22283	1	0	0.42	0.001	-8.714245518	Down	0.53554	0.81885857
319565	1	0	0.31	0.001	-8.276124405	Down	0.53554	0.756446841
100039192	3	0.02	14.77	0.09	-7.358529109	Down	0.1434012	0.548869082
26875	3.08	0.04	1.02	0.02	-5.672425342	Down	0.1434012	0.5365294
27061	23	1	117.98	5.5	-4.422966885	Down	3.42E-06	0.000227584
74237	18	1	41.75	2.49	-4.06755855	Down	7.45E-05	0.003349729
69136	17	1	84.9	5.35	-3.988153757	Down	0.000137091	0.005510809
66938	16	1	56.43	3.78	-3.90000421	Down	0.00025173	0.009227512
66102	16	1	43.98	2.95	-3.898060745	Down	0.00025173	0.009268342
12842	44	3	49.35	3.61	-3.772979342	Down	5.56E-10	8.26E-08
20932	14	1	32.83	2.51	-3.709255481	Down	0.000841518	0.024483466
216974	14	1	62.19	4.76	-3.707649139	Down	0.000841518	0.024569373
108800	15.4	1.12	10.35	0.81	-3.67556505	Down	0.000460962	0.015719938
69539	13	1	52.73	4.35	-3.599536691	Down	0.001530976	0.038839181
68020	13.28	1.06	85.92	7.36	-3.545216322	Down	0.001530976	0.038957955
69564	16.85	1.36	61.94	5.37	-3.52787739	Down	0.00025173	0.009309535
19988	12	1	63.88	5.7	-3.486330488	Down	0.00277454	0.060437035
27410	12	1	12.42	1.11	-3.484033592	Down	0.00277454	0.060595662
14814	11	1	16.77	1.63	-3.362938819	Down	0.00500604	0.095539584

19317	11	1	15.78	1.54	-3.357094949	Down		0.00500604	0.095759216
50794	20	2	20.84	2.23	-3.224239662	Down		0.00012732	0.005167966
20055	10	1	111.07	11.9	-3.222435719	Down		0.00898672	0.134979237
114674	10	1	19.32	2.07	-3.222392421	Down		0.00898672	0.135468292
216197	10	1	23.03	2.47	-3.220931464	Down		0.00898672	0.135714151
17390	19	2	41.51	4.69	-3.145799104	Down		0.000225686	0.00853606
19349	10	1	31.03	3.52	-3.140016363	Down		0.00898672	0.135223322
321022	9	1	18.08	2.15	-3.071986113	Down		0.01603832	0.191196076
17210	9	1	17.23	2.05	-3.071226887	Down		0.01603832	0.193693557
73836	9	1	28.39	3.38	-3.070287697	Down		0.01603832	0.194540613
101100	9	1	19.31	2.3	-3.069642398	Down		0.01603832	0.19174549
16599	9	1	26.19	3.12	-3.069398125	Down		0.01603832	0.192853845
20249	9	1	12.41	1.48	-3.067834035	Down		0.01603832	0.192575557
19099	9	1	20.45	2.44	-3.06714779	Down		0.01603832	0.193412842
72587	9	1	12.99	1.55	-3.06706131	Down		0.01603832	0.194257439
26360	9	1	17.51	2.09	-3.066604236	Down		0.01603832	0.194824614
72033	9	1	6.78	0.81	-3.06529146	Down		0.01603832	0.19229807
232341	9	1	6.09	0.73	-3.060473859	Down		0.01603832	0.191470388
56279	17	2	70.46	8.88	-2.988172893	Down		0.000700646	0.021592872
19671	17	2	79.96	10.08	-2.987782833	Down		0.000700646	0.021434101
105734	17	2	23.4	2.95	-2.98772167	Down		0.000700646	0.021513193
14312	33	4	48.57	6.36	-2.932966816	Down		1.70E-06	0.000122853
18045	8	1	21.08	2.82	-2.902107799	Down		0.0284266	0.263405054
11717	8	1	13.45	1.8	-2.901537361	Down		0.0284266	0.26577274
320799	8	1	11.88	1.59	-2.901436166	Down		0.0284266	0.263698705
228790	8	1	7.99	1.07	-2.900584707	Down		0.0284266	0.2645836

22160	24	3	97.7	13.09	-2.899893465	Down	5.87E-05	0.002759121
11298	8	1	39.77	5.33	-2.899473124	Down	0.0284266	0.265176837
66461	8	1	41.26	5.53	-2.899392436	Down	0.0284266	0.266671633
73121	16	2	65.05	8.72	-2.899149017	Down	0.00122616	0.033784362
12643	8	1	32.81	4.4	-2.898560165	Down	0.0284266	0.266972617
98193	8	1	14.24	1.91	-2.898304603	Down	0.0284266	0.263993012
26388	8	1	30.19	4.05	-2.898076944	Down	0.0284266	0.267274281
101437	8	1	21.99	2.95	-2.898060745	Down	0.0284266	0.266071697
110052	8	1	20.64	2.77	-2.897485089	Down	0.0284266	0.266371327
11350	9.92	1.25	8.97	1.22	-2.878226837	Down	0.01603832	0.193975088
56176	31	4	227.8	31.38	-2.85985049	Down	5.10E-06	0.000326424
227753	8	1	20.19	2.82	-2.839873843	Down	0.0284266	0.263112056
67795	23	3	113.24	15.83	-2.838650494	Down	0.000101784	0.004299203
21665	93	12	197.83	27.66	-2.838388159	Down	1.03E-15	2.68E-13
319909	23	3	52.06	7.28	-2.838164956	Down	0.000101784	0.004321138
67480	11.02	1.47	22.21	3.17	-2.808654647	Down	0.00500604	0.095979859
224008	15	2	30.44	4.35	-2.806881053	Down	0.00213482	0.05134057
11950	15	2	61.98	8.86	-2.806424151	Down	0.00213482	0.050899247
270035	15	2	47.84	6.84	-2.806149159	Down	0.00213482	0.051045509
101488	15	2	21.62	3.27	-2.725003982	Down	0.00213482	0.051192614
268481	7	0.99	17.19	2.61	-2.719447833	Down	0.01028194	0.150892456
245683	7	1	17.92	2.74	-2.709322839	Down	0.0499732	0.364440839
12902	7	1	7.52	1.15	-2.709098801	Down	0.0499732	0.367338337
54128	7	1	26.28	4.02	-2.708697869	Down	0.0499732	0.368314435
16949	7	1	14.05	2.15	-2.708161566	Down	0.0499732	0.366044892
18599	7	1	12.47	1.91	-2.706816922	Down	0.0499732	0.367663127

69674	7	1	36.3	5.56	-2.70681276	Down	0.0499732	0.364760524
353190	7	1	14.03	2.15	-2.706106444	Down	0.0499732	0.367014119
330064	7	1	13.57	2.08	-2.705765287	Down	0.0499732	0.365722953
20607	7	1	11.92	1.83	-2.703468682	Down	0.0499732	0.367988493
433938	7	1	7.62	1.17	-2.703282468	Down	0.0499732	0.366690474
72993	7	1	6.7	1.03	-2.701516758	Down	0.0499732	0.36508077
17884	13.9	2	15.36	2.37	-2.696219252	Down	0.0063572	0.108398076
319166	11.84	1.71	150.31	23.2	-2.695744283	Down	0.00500604	0.095320958
66729	8	1	30.54	4.81	-2.666591263	Down	0.0284266	0.264879886
271981	15.51	2.3	15.71	2.5	-2.651683181	Down	0.00213482	0.051489383
71888	8	1.19	32.29	5.15	-2.648443104	Down	0.0284266	0.264287976
353326	47	7	59.92	9.57	-2.646446794	Down	5.36E-08	5.13E-06
96875	15	3	46.65	7.68	-2.602698865	Down	0.00698098	0.114799871
504186	13	2	34.8	5.74	-2.599964664	Down	0.0063572	0.10862066
74201	6.94	1.09	5.85	0.98	-2.57758297	Down	0.0869852	0.447068468
74006	6	1	10.49	1.78	-2.559065532	Down	0.0869852	0.449009832
214111	6	1	7.64	1.36	-2.489965987	Down	0.0869852	0.449567608
18578	6	1	9.54	1.7	-2.48845452	Down	0.0869852	0.445966635
236690	6	1	11.05	1.97	-2.487778835	Down	0.0869852	0.445417753
12156	6	1	11.27	2.01	-2.487220109	Down	0.0869852	0.450126772
16779	6	1	7.12	1.27	-2.487048744	Down	0.0869852	0.444596959
223697	6	1	10.82	1.93	-2.487027747	Down	0.0869852	0.448175758
223723	6	1	10.65	1.9	-2.486782107	Down	0.0869852	0.442964412
16549	6	1	10.03	1.79	-2.486290113	Down	0.0869852	0.441882692
378460	6	1	17.65	3.15	-2.48624445	Down	0.0869852	0.449288547
20238	6	1	3.81	0.68	-2.486184346	Down	0.0869852	0.445143819

52163	6	1	28.46	5.08	-2.48603526	Down	0.0869852	0.448731463
68097	6	1	16.3	2.91	-2.485780906	Down	0.0869852	0.45096813
19353	6	1	17.7	3.16	-2.485752897	Down	0.0869852	0.445692025
276905	6	1	17.98	3.21	-2.485747818	Down	0.0869852	0.450406876
70351	6	1	10.53	1.88	-2.485700869	Down	0.0869852	0.447898422
12684	6	1	34.61	6.18	-2.485510198	Down	0.0869852	0.442693486
214804	6	1	7.28	1.3	-2.485426827	Down	0.0869852	0.453227207
18712	6	1	22.96	4.1	-2.485426827	Down	0.0869852	0.447344777
17256	6	1	49.39	8.82	-2.485368408	Down	0.0869852	0.447621428
19035	6	1	42.21	7.54	-2.484948401	Down	0.0869852	0.442422891
225895	12	2	36.72	6.56	-2.484798339	Down	0.01085764	0.150326826
71361	6	1	27.37	4.89	-2.484689064	Down	0.0869852	0.448453438
71310	6	1	7.78	1.39	-2.484685272	Down	0.0869852	0.451249283
21887	6	1	7.78	1.39	-2.484685272	Down	0.0869852	0.450687328
207165	6	1	3.47	0.62	-2.484595542	Down	0.0869852	0.441613087
57274	6	1	21.49	3.84	-2.484487267	Down	0.0869852	0.451530786
70382	12	2	45.44	8.12	-2.484411202	Down	0.01085764	0.150077114
75563	6	1	19.41	3.47	-2.48379255	Down	0.0869852	0.442152626
102103	6	1	9.62	1.72	-2.483628329	Down	0.0869852	0.443779184
20747	6	1	13.98	2.5	-2.483364361	Down	0.0869852	0.44134381
75732	6	1	24.38	4.36	-2.483298086	Down	0.0869852	0.452660318
545554	6	1	15.6	2.79	-2.483209002	Down	0.0869852	0.446241584
385668	6	1	12.69	2.27	-2.482927867	Down	0.0869852	0.443507261
242406	6	1	6.82	1.22	-2.482890591	Down	0.0869852	0.45181264
74185	6	1	13.47	2.41	-2.482644799	Down	0.0869852	0.4467925
381022	6	1	2.01	0.36	-2.48112669	Down	0.0869852	0.452094846

227835	5.98	1	15.57	2.79	-2.480431917	Down	0.1495702	0.531188064
70683	6	1	4.52	0.81	-2.48032896	Down	0.0869852	0.452943585
26374	6	1	7.92	1.42	-2.479609501	Down	0.0869852	0.444051441
319622	6	1	5.8	1.04	-2.479469372	Down	0.0869852	0.44323567
242747	5.98	1	9.34	1.68	-2.474961317	Down	0.1495702	0.535992091
19365	6	1	24.33	4.47	-2.444389584	Down	0.0869852	0.444324033
12955	17	3	104.31	19.73	-2.402414612	Down	0.00251354	0.058259516
63955	34	6	69.8	13.21	-2.40159657	Down	1.33E-05	0.000766838
109095	17	3	37.82	7.16	-2.401117871	Down	0.00251354	0.058422252
241638	6	1	9.42	1.8	-2.387730153	Down	0.0869852	0.444870221
277854	5.59	1	4.69	0.9	-2.381591016	Down	0.1495702	0.527585262
234135	9	4	15.37	2.96	-2.376448084	Down	0.226072	0.63041056
13424	11	2	5.08	0.99	-2.359328067	Down	0.01839512	0.206010489
66993	11	2	42.73	8.33	-2.358860916	Down	0.01839512	0.206288131
55982	10.61	1.93	12.07	2.36	-2.354566911	Down	0.00898672	0.134736031
19326	37.57	7	41.3	8.25	-2.323675757	Down	9.10E-06	0.000561149
207212	32	6	20.76	4.17	-2.315687155	Down	3.64E-05	0.001834846
18412	5	1	17.63	3.56	-2.308083328	Down	0.1495702	0.527138346
231805	21	4	54.56	11.14	-2.292094412	Down	0.000998358	0.028449784
52668	7	1	47.05	9.71	-2.276651522	Down	0.0499732	0.365401579
107723	56	11	62.55	13.17	-2.247754539	Down	6.20E-08	5.86E-06
12894	5	1	7.72	1.65	-2.226134823	Down	0.1495702	0.536917012
14284	5	1	5.66	1.21	-2.225795006	Down	0.1495702	0.531641877
22134	5	1	6.64	1.42	-2.225292312	Down	0.1495702	0.529155457
16319	5	1	10.42	2.23	-2.224239662	Down	0.1495702	0.531869074
16419	5	1	10.23	2.19	-2.22380337	Down	0.1495702	0.532324052

19197	5	1	75.62	16.19	-2.223664864	Down	0.1495702	0.537380671
20277	5	1	12.47	2.67	-2.223549818	Down	0.1495702	0.537148742
17306	5	1	10.32	2.21	-2.223324696	Down	0.1495702	0.530508795
233038	5	1	22.18	4.75	-2.223259947	Down	0.1495702	0.534151774
382014	5	1	8.87	1.9	-2.222934686	Down	0.1495702	0.533693668
12057	5	1	13.91	2.98	-2.222738184	Down	0.1495702	0.532096466
66775	5	1	16.15	3.46	-2.222690222	Down	0.1495702	0.530735025
17357	5	1	21.05	4.51	-2.222620895	Down	0.1495702	0.529605802
228866	10	2	24.41	5.23	-2.222589444	Down	0.0308804	0.269345711
243529	5	1	30.43	6.52	-2.222550464	Down	0.1495702	0.530056914
22627	5	1	16.24	3.48	-2.222392421	Down	0.1495702	0.528930571
18938	15	3	112.7	24.15	-2.222392421	Down	0.00698098	0.114573441
15983	10	2	34.11	7.31	-2.222251444	Down	0.0308804	0.270195382
433294	5	1	31.59	6.77	-2.222240198	Down	0.1495702	0.538077663
52430	15	3	81.61	17.49	-2.222215653	Down	0.00698098	0.115484562
56538	5	1	26.55	5.69	-2.222211303	Down	0.1495702	0.53507035
12913	15	3	64.34	13.79	-2.222093479	Down	0.00698098	0.115255426
17970	5	1	9.33	2	-2.221877081	Down	0.1495702	0.53300798
71870	10	2	37.13	7.96	-2.221744979	Down	0.0308804	0.26962834
245944	5	1	7.37	1.58	-2.221740061	Down	0.1495702	0.527809005
229487	5	1	14.6	3.13	-2.221733807	Down	0.1495702	0.528032938
216871	5	1	28.08	6.02	-2.221707544	Down	0.1495702	0.535530824
19012	5	1	20.85	4.47	-2.221700647	Down	0.1495702	0.526692185
69089	10	2	26.54	5.69	-2.221667813	Down	0.0308804	0.271336651
338372	10	2	19.73	4.23	-2.221661388	Down	0.0308804	0.269911563
56397	5	1	19.45	4.17	-2.221650866	Down	0.1495702	0.532779809

623230	5	1	24.86	5.33	-2.221618858	Down	0.1495702	0.532551833
105014	5	1	23.04	4.94	-2.22155777	Down	0.1495702	0.529380533
13864	20	4	60.77	13.03	-2.221522208	Down	0.00164738	0.041413441
72401	10	2	26.49	5.68	-2.221485009	Down	0.0308804	0.271050431
67871	5	1	14.97	3.21	-2.221429019	Down	0.1495702	0.536223022
245688	5	1	14.83	3.18	-2.221419927	Down	0.1495702	0.531414874
69073	5	1	19.4	4.16	-2.221401219	Down	0.1495702	0.530961448
28106	5	1	37.82	8.11	-2.221375544	Down	0.1495702	0.528705877
234373	5	1	8.58	1.84	-2.221271881	Down	0.1495702	0.535300488
67622	5	1	19.63	4.21	-2.221168034	Down	0.1495702	0.533236347
80744	5	1	9.93	2.13	-2.220940287	Down	0.1495702	0.537612801
212706	5	1	12.96	2.78	-2.22090893	Down	0.1495702	0.53484041
81630	5	1	12.54	2.69	-2.22085927	Down	0.1495702	0.537845131
20516	15	3	27.45	5.89	-2.22046661	Down	0.00698098	0.114347903
56456	5	1	17.15	3.68	-2.220430905	Down	0.1495702	0.528481373
67285	5	1	17.94	3.85	-2.220249539	Down	0.1495702	0.536685483
211488	5	1	7.5	1.61	-2.219829907	Down	0.1495702	0.527361709
217734	15	3	19.7	4.23	-2.219466061	Down	0.00698098	0.115027197
218210	5	1	5.54	1.19	-2.218924403	Down	0.1495702	0.535761358
227334	10	2	13.22	2.84	-2.218759342	Down	0.0308804	0.270764814
17387	5	1	12.94	2.78	-2.218680829	Down	0.1495702	0.529831262
320938	5	1	8.42	1.81	-2.217830536	Down	0.1495702	0.52825706
20481	5	1	6.09	1.31	-2.216875416	Down	0.1495702	0.53346491
22215	5	1	8.6	1.85	-2.216811389	Down	0.1495702	0.536454153
19679	10	2	9.56	2.06	-2.214366281	Down	0.0308804	0.270479798
17064	4.99	1	5.01	1.08	-2.213779291	Down	0.253238	0.682381282

97998	5	1	3.89	0.85	-2.194235409	Down	0.1495702	0.534610668
16179	7.7	1.59	13.45	2.99	-2.169388783	Down	0.0499732	0.366367398
231727	24	5	114.76	25.62	-2.163277492	Down	0.000651646	0.020539191
235312	9.46	5	37.19	8.39	-2.148172032	Down	0.370266	0.742405635
232969	5	1	27.84	6.3	-2.143735477	Down	0.1495702	0.533922623
66898	28	6	57.79	13.28	-2.121564723	Down	0.000259694	0.009477692
330671	14	3	30.24	6.95	-2.121373257	Down	0.01150002	0.157129173
239102	14	3	10.66	2.45	-2.121353784	Down	0.01150002	0.156871584
110385	5.65	1.23	11.83	2.75	-2.10494655	Down	0.1495702	0.534381122
14950	5	2	11.3	2.65	-2.092258508	Down	0.337326	0.691522455
65964	4.68	1.03	4.65	1.1	-2.079727192	Down	0.253238	0.669587988
227800	9	2	12.1	2.88	-2.070866331	Down	0.0512964	0.362956926
69572	9	2	27.98	6.66	-2.07080188	Down	0.0512964	0.366069764
234542	9	2	31.63	7.53	-2.070571786	Down	0.0512964	0.363885204
368202	9	2	65.4	15.57	-2.070521691	Down	0.0512964	0.364818243
78369	9	2	68.55	16.32	-2.070515609	Down	0.0512964	0.363575251
20807	9	2	14.7	3.5	-2.070389328	Down	0.0512964	0.3623407
110355	9	2	17.85	4.25	-2.070389328	Down	0.0512964	0.362033371
19881	9	2	40.06	9.54	-2.07010125	Down	0.0512964	0.362648551
69737	9	2	13.18	3.14	-2.069513906	Down	0.0512964	0.364506699
72873	9	2	27.7	6.6	-2.069348047	Down	0.0512964	0.36513032
17918	9	2	5.12	1.22	-2.069262662	Down	0.0512964	0.365442932
17132	22	5	40.44	9.85	-2.037587368	Down	0.001710536	0.042742853
67023	7	2	55.42	13.53	-2.034244872	Down	0.136159	0.575700731
213522	35	8	77.78	19.06	-2.028851116	Down	6.71E-05	0.003102947
328329	13	3	8.1	2	-2.017921908	Down	0.01877756	0.209728962

72831	13	3	22.85	5.65	-2.015871393	Down	0.01877756	0.209447824
74763	13	3	37.69	9.32	-2.015779935	Down	0.01877756	0.209167439
170460	13	3	34.98	8.65	-2.015758251	Down	0.01877756	0.210010856
101994	13	3	21.65	5.36	-2.014062119	Down	0.01877756	0.208887803
17879	12.89	3	14.19	3.54	-2.003053324	Down	0.0303596	0.268176467
319185	4.28	1	71.68	17.99	-1.994375451	Down	0.253238	0.685935351
67097	17	4	209.85	52.87	-1.988837231	Down	0.00710618	0.115715311
110168	17	4	86.86	21.9	-1.987761083	Down	0.00710618	0.115489304
13589	11.85	2.79	10.76	2.72	-1.983999521	Down	0.01839512	0.206566523
67573	25	6	30.64	7.88	-1.959148762	Down	0.001074994	0.030219679
319151	4.01	0.97	61.14	15.81	-1.951279185	Down	0.074205	0.410818234
14729	29	7	88.24	22.83	-1.950501931	Down	0.000425298	0.014745436
72055	9	4	24.2	6.28	-1.946170583	Down	0.226072	0.630833371
50875	4.03	0.99	7.52	1.98	-1.925232232	Down	0.074205	0.448409444
237387	43.73	10.83	67.8	18.01	-1.912487092	Down	1.10E-05	0.000655873
18541	4	1	2.81	0.75	-1.90560763	Down	0.253238	0.675815715
269424	8	2	9.59	2.56	-1.905387005	Down	0.0841744	0.446693356
18145	4	1	5.13	1.37	-1.904782933	Down	0.253238	0.676683814
52468	4	1	5.54	1.48	-1.9042888	Down	0.253238	0.67387061
207214	4	1	4.08	1.09	-1.904241017	Down	0.253238	0.676901188
76952	4	1	6.4	1.71	-1.90407558	Down	0.253238	0.674301887
11774	4	1	6.66	1.78	-1.903644936	Down	0.253238	0.668101902
384569	4	1	3.18	0.85	-1.903492019	Down	0.253238	0.668737987
263764	4	1	4.6	1.23	-1.902975546	Down	0.253238	0.670867048
15526	4	1	8.75	2.34	-1.902774487	Down	0.253238	0.680837932
57294	4	1	79.21	21.19	-1.90229899	Down	0.253238	0.673224728

64424	4	1	7.55	2.02	-1.902121351	Down	0.253238	0.685489069
16909	4	1	16.48	4.41	-1.901865682	Down	0.253238	0.678208368
70737	8	2	10.65	2.85	-1.901819606	Down	0.0841744	0.447549637
231042	4	1	9.23	2.47	-1.901819606	Down	0.253238	0.683931645
64291	4	1	9.23	2.47	-1.901819606	Down	0.253238	0.680618023
22186	4	1	54.32	14.54	-1.90145621	Down	0.253238	0.675382499
76646	4	1	22.6	6.05	-1.901315725	Down	0.253238	0.673439884
21953	8	2	84.08	22.51	-1.901196613	Down	0.0841744	0.445273479
20324	4	1	8.74	2.34	-1.90112475	Down	0.253238	0.682823525
68598	4	1	19.31	5.17	-1.901111979	Down	0.253238	0.677336354
17330	4	1	10.27	2.75	-1.900932658	Down	0.253238	0.673009709
17688	12	3	18.71	5.01	-1.90092705	Down	0.0303596	0.268461458
66437	4	1	28.53	7.64	-1.900835204	Down	0.253238	0.672151004
268903	4	1	5.9	1.58	-1.900790396	Down	0.253238	0.680178631
634731	4	1	8.14	2.18	-1.900700659	Down	0.253238	0.684820734
224090	4	1	6.31	1.69	-1.900616759	Down	0.253238	0.672580082
231130	4	1	13.74	3.68	-1.900604333	Down	0.253238	0.684375901
226517	8	2	9.11	2.44	-1.900569906	Down	0.0841744	0.445556732
231691	4	1	23.52	6.3	-1.900464326	Down	0.253238	0.679739806
238123	4	1	8.96	2.4	-1.900464326	Down	0.253238	0.672365475
22724	12	3	21.69	5.81	-1.900419984	Down	0.0303596	0.267325113
27280	4	1	18.33	4.91	-1.900411856	Down	0.253238	0.670226908
67811	8	2	27.7	7.42	-1.900394884	Down	0.0841744	0.446978419
67009	4	1	13.29	3.56	-1.900391958	Down	0.253238	0.67646658
20227	16	4	20.98	5.62	-1.900372642	Down	0.01138882	0.156122523
217835	4	1	7.13	1.91	-1.900329438	Down	0.253238	0.682160375

67628	4	1	19.71	5.28	-1.900317942	Down	0.253238	0.683709733
17085	4	1	12.43	3.33	-1.900232214	Down	0.253238	0.679959147
20324	4	1	8.74	2.34	-1.90112475	Down	0.253238	0.682823525
68598	4	1	19.31	5.17	-1.901111979	Down	0.253238	0.677336354
17330	4	1	10.27	2.75	-1.900932658	Down	0.253238	0.673009709
17688	12	3	18.71	5.01	-1.90092705	Down	0.0303596	0.268461458
66437	4	1	28.53	7.64	-1.900835204	Down	0.253238	0.672151004
268903	4	1	5.9	1.58	-1.900790396	Down	0.253238	0.680178631
634731	4	1	8.14	2.18	-1.900700659	Down	0.253238	0.684820734
224090	4	1	6.31	1.69	-1.900616759	Down	0.253238	0.672580082
231130	4	1	13.74	3.68	-1.900604333	Down	0.253238	0.684375901
226517	8	2	9.11	2.44	-1.900569906	Down	0.0841744	0.445556732
231691	4	1	23.52	6.3	-1.900464326	Down	0.253238	0.679739806
238123	4	1	8.96	2.4	-1.900464326	Down	0.253238	0.672365475
22724	12	3	21.69	5.81	-1.900419984	Down	0.0303596	0.267325113
27280	4	1	18.33	4.91	-1.900411856	Down	0.253238	0.670226908
67811	8	2	27.7	7.42	-1.900394884	Down	0.0841744	0.446978419
67009	4	1	13.29	3.56	-1.900391958	Down	0.253238	0.67646658
20227	16	4	20.98	5.62	-1.900372642	Down	0.01138882	0.156122523
217835	4	1	7.13	1.91	-1.900329438	Down	0.253238	0.682160375
67628	4	1	19.71	5.28	-1.900317942	Down	0.253238	0.683709733
17085	4	1	12.43	3.33	-1.900232214	Down	0.253238	0.679959147
56018	4	1	22.83	6.12	-1.899327302	Down	0.253238	0.681498512
75871	4	1	13.13	3.52	-1.899219582	Down	0.253238	0.676249486
225875	12	3	27.49	7.37	-1.899170382	Down	0.0303596	0.26789208
68188	4	1	6.49	1.74	-1.899131172	Down	0.253238	0.685266146

16371	4	1	12.98	3.48	-1.899131172	Down	0.253238	0.670013799
15160	4	1	12.23	3.28	-1.898656684	Down	0.253238	0.672794827
75607	4	1	3.84	1.03	-1.898461974	Down	0.253238	0.669375285
27050	4	1	14.39	3.86	-1.898393839	Down	0.253238	0.682602332
19205	4	1	9.17	2.46	-1.898263418	Down	0.253238	0.678863852
66995	4	1	9.58	2.57	-1.898257297	Down	0.253238	0.668525824
12879	4	1	15.32	4.11	-1.898205998	Down	0.253238	0.681057983
18746	4	1	11.07	2.97	-1.898120386	Down	0.253238	0.68171899
64010	4	1	10.66	2.86	-1.898120386	Down	0.253238	0.677118701
93762	4	1	5.74	1.54	-1.898120386	Down	0.253238	0.679520606
212974	4	1	9.02	2.42	-1.898120386	Down	0.253238	0.679301547
223650	4	1	1.23	0.33	-1.898120386	Down	0.253238	0.671508412
268417	4	1	7.08	1.9	-1.897749942	Down	0.253238	0.674949839
80898	4	1	6.67	1.79	-1.897727174	Down	0.253238	0.670440152
239845	4	1	5.81	1.56	-1.896992135	Down	0.253238	0.685043367
74355	8	2	7.56	2.03	-1.896906507	Down	0.0841744	0.445840345
18709	4	1	8.49	2.28	-1.896730729	Down	0.253238	0.675599038
227449	4	1	4.58	1.23	-1.896689283	Down	0.253238	0.671722473
100503884	4	1	8.08	2.17	-1.89666025	Down	0.253238	0.684153701
268420	4	1	4.43	1.19	-1.896345125	Down	0.253238	0.67408618
243372	4	1	6.55	1.76	-1.895919478	Down	0.253238	0.683044862
18844	4	1	2.94	0.79	-1.895891597	Down	0.253238	0.6751661
69228	4	1	7.33	1.97	-1.895617569	Down	0.253238	0.681939611
75547	4	1	2.12	0.57	-1.89503044	Down	0.253238	0.677990154
207521	4	1	4.61	1.24	-1.89442663	Down	0.253238	0.668950285
238377	4	1.01	9.14	2.46	-1.89353585	Down	0.253238	0.679082629

66394	4	1.01	13.12	3.55	-1.88587679	Down	0.253238	0.674733717
245050	3	1	7.22	1.98	-1.866498407	Down	0.420186	0.80728878
227634	6	1.55	5	1.38	-1.857259828	Down	0.0869852	0.446516872
234852	23	6	70.86	19.82	-1.838014503	Down	0.00268852	0.06129089
15248	5	1	7.87	2.21	-1.832317266	Down	0.1495702	0.526915171
11799	4.01	1.05	7.86	2.21	-1.830482943	Down	0.253238	0.671294488
399603	19	5	22.79	6.43	-1.825510282	Down	0.00691518	0.11485272
78656	12.2	3.23	18.07	5.13	-1.816565775	Down	0.0303596	0.267042528
12825	49	13	58.75	16.71	-1.813877118	Down	1.08E-05	0.000652457
18201	15	4	28.56	8.16	-1.807354922	Down	0.0180886	0.205061636
66733	15	4	26.26	7.51	-1.805982103	Down	0.0180886	0.205341392
243846	4	1	12.35	3.56	-1.794561896	Down	0.253238	0.67193667
69537	4	1	16.59	4.8	-1.789207575	Down	0.253238	0.668313796
218490	3	1	22.88	6.63	-1.787006277	Down	0.420186	0.809157072
13548	11	3	12.15	3.55	-1.775065384	Down	0.048545	0.359699862
216551	11	3	20.26	5.92	-1.774965093	Down	0.048545	0.36002045
78670	11	3	58.84	17.2	-1.774388682	Down	0.048545	0.359379844
23942	11	3	23.42	6.85	-1.773565183	Down	0.048545	0.360663344
100040531	4.72	1.3	42.07	12.4	-1.762451697	Down	0.253238	0.685712137
72075	29	8	84.7	25.05	-1.757551366	Down	0.00101735	0.028793773
67768	5.67	1.57	23.29	6.93	-1.748783382	Down	0.1495702	0.530282759
22004	18	5	57.6	17.15	-1.747860235	Down	0.01084494	0.150401243
57434	4.28	1.19	8.88	2.65	-1.744567317	Down	0.253238	0.669800826
93706	18.04	5.05	25.71	7.72	-1.735656858	Down	0.01084494	0.15065233
19367	496	139	1636.18	491.53	-1.734980094	Down	7.54E-43	6.27E-40
21678	4	1	9.68	2.94	-1.719190892	Down	0.253238	0.67777208

67302	7	2	7.59	2.32	-1.70997508	Down	0.136159	0.575116263
381352	7	2	11.74	3.59	-1.709376659	Down	0.136159	0.574532981
27207	7	2	82	25.09	-1.708511439	Down	0.136159	0.576286388
216190	7	2	15.88	4.86	-1.708182694	Down	0.136159	0.573950881
407831	7	2	25.38	7.77	-1.707705565	Down	0.136159	0.575408349
93670	7	2	38.31	11.73	-1.707518012	Down	0.136159	0.576579664
67457	14	4	29.65	9.08	-1.707267902	Down	0.0284464	0.262710871
103142	14	4	34.87	10.68	-1.707074717	Down	0.0284464	0.263002772
15559	21	6	68.36	20.94	-1.706890955	Down	0.00653692	0.109664741
67291	7	2	17.04	5.22	-1.706803624	Down	0.136159	0.572790212
11539	56	16	81.54	24.98	-1.70673448	Down	6.27E-06	0.00039252
30935	7	2	14.36	4.4	-1.70648032	Down	0.136159	0.572500778
21429	7	2	10.18	3.12	-1.706119627	Down	0.136159	0.572211636
14593	7	2	19.74	6.05	-1.706114942	Down	0.136159	0.573079939
78754	7	2	20.13	6.17	-1.706004778	Down	0.136159	0.571922786
210711	7	2	11.22	3.44	-1.705592206	Down	0.136159	0.575993411
57138	7	2	7.72	2.37	-1.703713788	Down	0.136159	0.574241784
70118	34	10	205.39	64.74	-1.665636671	Down	0.00059047	0.019343704
215113	17	5	15.79	4.98	-1.664793524	Down	0.01685708	0.193472776
330217	34	10	72.1	22.74	-1.664767005	Down	0.00059047	0.019267847
53608	71	21	109.52	34.73	-1.656940036	Down	6.00E-07	4.94E-05
15482	33.67	10	93.83	29.88	-1.650869117	Down	0.000896994	0.025826599
14422	77.72	23.14	127.21	40.61	-1.647305153	Down	2.35E-07	2.08E-05
20116	10	3	94.22	30.28	-1.637668127	Down	0.0766598	0.413943021
67739	10	3	26.63	8.56	-1.637369727	Down	0.0766598	0.414480959
243912	10	3	49.24	15.83	-1.637169506	Down	0.0766598	0.413674576

15936	10	3	44.06	14.17	-1.636629737	Down		0.0766598	0.415020297
56212	10	3	54.01	17.37	-1.636628794	Down		0.0766598	0.413406478
14184	20	6	33.79	10.87	-1.63624441	Down		0.0100731	0.148613945
12301	10	3	31.89	10.26	-1.636073367	Down		0.0766598	0.414211815
67333	10	3	12.23	3.94	-1.634156869	Down		0.0766598	0.412871324
13170	23	7	92.89	30.3	-1.616205499	Down		0.00605832	0.104805157
230661	23	7	50.89	16.61	-1.615330118	Down		0.00605832	0.105023502
320560	9.47	2.89	6.4	2.09	-1.614568963	Down		0.0512964	0.363265825
66395	13	4	4.77	1.57	-1.603224707	Down		0.044249	0.335638951
66475	13	4	160.78	52.97	-1.601840542	Down		0.044249	0.335028143
22330	13	4	16.59	5.47	-1.600701148	Down		0.044249	0.334116088
101113	13	4	58.09	19.16	-1.600192269	Down		0.044249	0.334419554
18221	13	4	64.97	21.43	-1.600141855	Down		0.044249	0.334723572
83984	42	13	210.7	69.9	-1.591825953	Down		0.000207854	0.00800719
328778	16	5	75.9	25.42	-1.578135855	Down		0.0259498	0.24790848
218772	16	5	40.01	13.41	-1.577051391	Down		0.0259498	0.248193432
56086	16	5	39.26	13.22	-1.570337996	Down		0.0259498	0.24847904
18572	35	11	38.5	12.97	-1.569679966	Down		0.000827838	0.02425507
26364	10	3	21.51	7.28	-1.562997169	Down		0.0766598	0.413138728
70891	19	6	67.92	22.99	-1.562829993	Down		0.01538816	0.188856754
74238	19	6	81.76	27.68	-1.562551253	Down		0.01538816	0.188301293
12526	11.68	3.7	56.12	19.06	-1.55796689	Down		0.048545	0.36034161
67171	3.28	1.04	8.97	3.05	-1.556298742	Down		0.420186	0.792467748
57741	7	2.23	16.88	5.77	-1.54867168	Down		0.136159	0.574824474
69358	31	10	228.47	78.72	-1.537202614	Down		0.00203358	0.049333584
74901	28	9	26.66	9.19	-1.536540014	Down		0.0033484	0.069481388

209497	1	1	3.87	1.35	-1.519374159	Down		0.947628	0.986514774
19765	10	3	16.26	5.68	-1.517364423	Down		0.0766598	0.414750452
78339	3	1	4.36	1.53	-1.510796482	Down		0.420186	0.795171186
56409	3	1	9.32	3.3	-1.49786393	Down		0.420186	0.799535263
11988	3	1	2.59	0.92	-1.493246332	Down		0.420186	0.798804594
67299	3	1	2.84	1.01	-1.491535637	Down		0.420186	0.801919199
210510	3	1	2.84	1.01	-1.491535637	Down		0.420186	0.798075258
217866	3	1	2.98	1.06	-1.491248066	Down		0.420186	0.809719246
207304	3	1	2.22	0.79	-1.490635118	Down		0.420186	0.793546915
77264	3	1	3.23	1.15	-1.489900304	Down		0.420186	0.791929265
385674	3	1	3.37	1.2	-1.489714186	Down		0.420186	0.801551514
19159	3	1	5.36	1.91	-1.488660362	Down		0.420186	0.795533039
53761	3	1	2.89	1.03	-1.488425155	Down		0.420186	0.79971814
80877	3	1	2.02	0.72	-1.488286481	Down		0.420186	0.803208754
22642	3	1	5.89	2.1	-1.487878306	Down		0.420186	0.796257733
218699	3	1	7.18	2.56	-1.487840034	Down		0.420186	0.80803506
101471	3	1	3.73	1.33	-1.487749385	Down		0.420186	0.794267993
242109	3	1	3.87	1.38	-1.487665299	Down		0.420186	0.806357866
64009	3	1	4.15	1.48	-1.487514161	Down		0.420186	0.806543877
666060	3	1	4.15	1.48	-1.487514161	Down		0.420186	0.804132407
54635	3	1	5.72	2.04	-1.487445995	Down		0.420186	0.803393315
21769	3	1	7.57	2.7	-1.487333893	Down		0.420186	0.80357796
54644	3	1	4.71	1.68	-1.487265827	Down		0.420186	0.801735314
434204	3	1	6.56	2.34	-1.487187285	Down		0.420186	0.809344376
21848	3	1	3.28	1.17	-1.487187285	Down		0.420186	0.807661748
108903	3	1	5.13	1.83	-1.487115177	Down		0.420186	0.800633777

319176	3	1	46	16.41	-1.487058622	Down	0.420186	0.805058187
19247	3	1	3.56	1.27	-1.487048744	Down	0.420186	0.799169761
78653	3	1	40.25	14.36	-1.486933034	Down	0.420186	0.806916156
16998	3	1	3.84	1.37	-1.486930418	Down	0.420186	0.805429096
621893	12	4	197.66	70.52	-1.486916566	Down	0.068001	0.416056118
239017	3	1	5.83	2.08	-1.486912355	Down	0.420186	0.79390729
50932	3	1	4.12	1.47	-1.486828182	Down	0.420186	0.790496881
381457	3	1	12.5	4.46	-1.48681248	Down	0.420186	0.791749933
76156	3	1	4.96	1.77	-1.48659076	Down	0.420186	0.80265558
12145	3	1	7.65	2.73	-1.486558797	Down	0.420186	0.791391513
225861	3	1	11.85	4.23	-1.486157491	Down	0.420186	0.796439113
17131	3	1	4.51	1.61	-1.486066745	Down	0.420186	0.806171941
229011	6	2	18.32	6.54	-1.486056963	Down	0.216528	0.682990708
330817	3	1	15.35	5.48	-1.485990857	Down	0.420186	0.793006964
75617	15	5	149.6	53.41	-1.485928386	Down	0.0395278	0.309126714
407243	3	1	11.96	4.27	-1.485909415	Down	0.420186	0.7999011
319322	3	1	6.19	2.21	-1.48589304	Down	0.420186	0.798987136
13866	6	2	8.01	2.86	-1.485787096	Down	0.216528	0.681956657
276770	3	1	16.72	5.97	-1.485772011	Down	0.420186	0.803947507
17869	6	2	16.86	6.02	-1.485769144	Down	0.216528	0.680668488
68193	3	1	30.61	10.93	-1.485709644	Down	0.420186	0.791033418
14969	3	1	12.18	4.35	-1.485426827	Down	0.420186	0.80228722
14661	6	2	12.74	4.55	-1.485426827	Down	0.216528	0.678362006
76223	6	2	30.8	11	-1.485426827	Down	0.216528	0.681698633
320484	3	1	5.46	1.95	-1.485426827	Down	0.420186	0.800817157
26363	3	1	10.36	3.7	-1.485426827	Down	0.420186	0.794809663

52589	3	1	5.88	2.1	-1.485426827	Down	0.420186	0.792827144
18432	6	2	9.52	3.4	-1.485426827	Down	0.216528	0.67861751
18830	6	2	22.48	8.03	-1.485170143	Down	0.216528	0.67989792
13885	3	1	10.19	3.64	-1.485143696	Down	0.420186	0.804502463
381990	9	3	18.7	6.68	-1.485118262	Down	0.1193492	0.514029344
12468	3	1	8.23	2.94	-1.485076276	Down	0.420186	0.808595677
14789	6	2	14.5	5.18	-1.485028897	Down	0.216528	0.681183171
77254	3	1	18.11	6.47	-1.484948929	Down	0.420186	0.808408718
217700	3	1	11.14	3.98	-1.484908897	Down	0.420186	0.797893132
71949	3	1	9.88	3.53	-1.484842858	Down	0.420186	0.790675646
15945	3	1	18.36	6.56	-1.484798339	Down	0.420186	0.794087601
30924	3	1	12.65	4.52	-1.484742707	Down	0.420186	0.797347253
192156	3	1	11.53	4.12	-1.48467627	Down	0.420186	0.810470029
18472	3	1	3.61	1.29	-1.484627771	Down	0.420186	0.790139595
15434	3	1	6.94	2.48	-1.484595542	Down	0.420186	0.801367799
67781	9	3	30.81	11.01	-1.484584213	Down	0.1193492	0.514562017
83962	3	1	6.66	2.38	-1.484560604	Down	0.420186	0.802839887
77630	12	4	25.8	9.22	-1.48453241	Down	0.068001	0.416362267
56613	3	1	6.38	2.28	-1.4845226	Down	0.420186	0.805614679
67923	3	1	21.35	7.63	-1.484481108	Down	0.420186	0.809906812
12017	6	2	29.94	10.7	-1.484463425	Down	0.216528	0.680154582
77058	3	1	11.78	4.21	-1.484447401	Down	0.420186	0.806729974
77134	9	3	22.58	8.07	-1.484404907	Down	0.1193492	0.514295543
270685	3	1	5.54	1.98	-1.484385546	Down	0.420186	0.790318198
11551	3	1	5.26	1.88	-1.484330138	Down	0.420186	0.796076436
19376	9	3	35.42	12.66	-1.484286807	Down	0.1193492	0.515630682

268470	6	2	10.1	3.61	-1.484284551	Down	0.216528	0.679385176
74782	3	1	9.4	3.36	-1.484199524	Down	0.420186	0.799352471
73635	6	2	27.92	9.98	-1.484187221	Down	0.216528	0.681440805
66901	12	4	33.68	12.04	-1.484056746	Down	0.068001	0.41575042
26932	3	1	4.14	1.48	-1.484033592	Down	0.420186	0.796620576
70155	3	1	4.14	1.48	-1.484033592	Down	0.420186	0.793186866
192292	9	3	28.28	10.11	-1.483999123	Down	0.1193492	0.5153631
57776	6	2	13.37	4.78	-1.483916942	Down	0.216528	0.680925732
54132	3	1	13.37	4.78	-1.483916942	Down	0.420186	0.798622135
217333	3	1	9.23	3.3	-1.483864623	Down	0.420186	0.792647406
64292	3	1	5.51	1.97	-1.483856689	Down	0.420186	0.810282203
11513	18	6	20.22	7.23	-1.483715445	Down	0.0232892	0.239838407
100986	3	1	1.65	0.59	-1.483679165	Down	0.420186	0.791570683
24056	3	1	8.11	2.9	-1.483649014	Down	0.420186	0.796983749
16514	3	1	6.46	2.31	-1.483641313	Down	0.420186	0.794448468
19684	3	1	4.81	1.72	-1.483628329	Down	0.420186	0.789961072
22192	3	1	15.8	5.65	-1.483601786	Down	0.420186	0.800267271
29806	9	3	12.36	4.42	-1.483560469	Down	0.1193492	0.515095795
434246	6	2	16.33	5.84	-1.483484517	Down	0.216528	0.677596648
20905	6	2	17.7	6.33	-1.483471956	Down	0.216528	0.677851576
23890	3	1	10.68	3.82	-1.483267104	Down	0.420186	0.804687619
109052	3	1	6.15	2.2	-1.483082887	Down	0.420186	0.804317393
94229	3	1	3.69	1.32	-1.483082887	Down	0.420186	0.800084143
103968	3	1	10.79	3.86	-1.483022112	Down	0.420186	0.79771109
74012	6	2	9.56	3.42	-1.483014293	Down	0.216528	0.682731901
381085	3	1	5.73	2.05	-1.482911229	Down	0.420186	0.807475221

109161	3	1	6.82	2.44	-1.482890591	Down	0.420186	0.805800347
104318	3	1	5.45	1.95	-1.482782106	Down	0.420186	0.808969853
70248	3	1	9.67	3.46	-1.482743852	Down	0.420186	0.79752913
380728	3	1	5.31	1.9	-1.482712442	Down	0.420186	0.80100062
233895	3	1	8.69	3.11	-1.482441597	Down	0.420186	0.795352071
16667	3	1	12.88	4.61	-1.482293938	Down	0.420186	0.794990383
230738	3	1	7.18	2.57	-1.482215485	Down	0.420186	0.793727062
224132	6	2	8.8	3.15	-1.482151695	Down	0.216528	0.679129095
232878	3	1	6.9	2.47	-1.48208532	Down	0.420186	0.798257467
218639	3	1	5.95	2.13	-1.482036238	Down	0.420186	0.792288173
22761	3	1	5.92	2.12	-1.481532911	Down	0.420186	0.801184167
16971	3	1	1.34	0.48	-1.48112669	Down	0.420186	0.805243599
16890	3	1	7.23	2.59	-1.481043549	Down	0.420186	0.794629024
229096	3	1	3.88	1.39	-1.480971769	Down	0.420186	0.80487286
23959	3	1	5.61	2.01	-1.480805269	Down	0.420186	0.796802121
12190	9	3	5.47	1.96	-1.480687179	Down	0.1193492	0.513763421
84092	3	1	4.8	1.72	-1.480625841	Down	0.420186	0.807848361
14012	3	1	6.25	2.24	-1.480357457	Down	0.420186	0.790854491
20667	3	1	4.52	1.62	-1.48032896	Down	0.420186	0.803762691
216188	3	1	3.32	1.19	-1.480221668	Down	0.420186	0.791212425
106042	3	1	4.91	1.76	-1.480147596	Down	0.420186	0.809531768
11622	3	1	3.71	1.33	-1.479992941	Down	0.420186	0.79336685
99470	3	1	3.04	1.09	-1.479743189	Down	0.420186	0.795895221
327954	3	1	1.45	0.52	-1.479469372	Down	0.420186	0.798439759
270210	3	1	3.15	1.13	-1.479029056	Down	0.420186	0.810094464
239857	3	1	2.09	0.75	-1.478540442	Down	0.420186	0.802103167

94281	14.94	5	36.75	13.19	-1.478299685	Down	0.05952	0.370153901
16671	2.99	1	12.73	4.57	-1.477966349	Down	0.678286	0.802961702
244059	3	1	2.2	0.79	-1.477578965	Down	0.420186	0.808221846
74180	3	1	1.33	0.48	-1.470319935	Down	0.420186	0.805986101
16001	3	1	1.66	0.6	-1.468148836	Down	0.420186	0.800450482
11568	59	20	96.86	35.21	-1.459915776	Down	3.42E-05	0.001733599
16157	2.96	1.01	11.39	4.15	-1.456584505	Down	0.678286	0.801251818
68024	2.88	1	44.06	16.38	-1.427534138	Down	0.678286	0.805827785
99311	14.06	4.88	29.34	10.91	-1.427217769	Down	0.0284464	0.262419617
68106	66	23	314.67	117.53	-1.420810584	Down	1.73E-05	0.000959907
24014	17.51	6.12	24.58	9.22	-1.41464626	Down	0.0348934	0.298711915
73833	20	7	106.2	39.84	-1.414494214	Down	0.0206816	0.215924208
227292	80	28	192.58	72.26	-1.414188723	Down	2.33E-06	0.000166012
72151	17	6	65.77	24.88	-1.402443185	Down	0.0348934	0.298098544
27008	17	6	16.89	6.39	-1.402281492	Down	0.0348934	0.298404914
22146	6.44	2.28	21.98	8.33	-1.399802986	Down	0.216528	0.679641452
18708	5	2	5.43	2.06	-1.398307861	Down	0.337326	0.686448923
29805	14	5	75.74	28.99	-1.385500088	Down	0.05952	0.369601433
218820	14	5	22.02	8.43	-1.385209933	Down	0.05952	0.369877461
73422	14	5	35.08	13.43	-1.385189443	Down	0.05952	0.369325817
22151	2.79	1	11.56	4.44	-1.380509816	Down	0.678286	0.801934897
27056	50	18	190.77	73.61	-1.373860638	Down	0.000275842	0.009936283
212168	25	9	38.43	14.83	-1.373714379	Down	0.01094942	0.151094733
81601	8	3	30.06	11.61	-1.372477037	Down	0.1828086	0.619865673
230577	2	1	6.05	2.34	-1.370426613	Down	0.678286	0.807210785
208144	45.97	16.59	72.57	28.07	-1.370344206	Down	0.000501044	0.016544393

110532	9.48	3.43	9.62	3.73	-1.366861264	Down	0.1193492	0.514828768
19941	11	4	138.1	53.77	-1.360839943	Down	0.1031016	0.509749503
269261	11	4	120.17	46.8	-1.360496343	Down	0.1031016	0.510052565
70625	11	4	24.28	9.46	-1.359856333	Down	0.1031016	0.509446801
230734	22	8	95.86	37.36	-1.359434485	Down	0.0182349	0.205044058
18764	11	4	14.06	5.48	-1.359348796	Down	0.1031016	0.510963915
319945	11	4	33.35	13	-1.359175138	Down	0.1031016	0.509144459
57783	11	4	27.73	10.81	-1.359081093	Down	0.1031016	0.510355987
73647	11	4	31.57	12.31	-1.358723499	Down	0.1031016	0.51065977
21416	11	4	18.33	7.15	-1.358191639	Down	0.1031016	0.511268423
433698	12.3	4.5	19.84	7.78	-1.350569965	Down	0.068001	0.416668867
18739	30	11	44.22	17.38	-1.347270943	Down	0.00584996	0.104234512
67604	6	2	19.22	7.57	-1.344243131	Down	0.216528	0.680411438
12794	16	6	80.09	31.57	-1.343067859	Down	0.0517124	0.364351296
21749	4.21	1.56	13.01	5.17	-1.331384776	Down	0.253238	0.683266342
242466	8	3	5.06	2.03	-1.317657658	Down	0.1828086	0.619613182
67945	32	12	501.05	201.12	-1.316898023	Down	0.0051575	0.097757534
235281	8	3	12.82	5.15	-1.315751925	Down	0.1828086	0.618605271
56012	24	9	183.01	73.53	-1.315517592	Down	0.01599712	0.194893171
69227	8	3	14.98	6.02	-1.315202232	Down	0.1828086	0.618353805
56431	8	3	28.19	11.33	-1.315035617	Down	0.1828086	0.618856941
67921	8	3	39.98	16.07	-1.314908543	Down	0.1828086	0.619360896
320844	16	6	42.21	16.97	-1.314598264	Down	0.0517124	0.363735317
71111	16	6	39.97	16.07	-1.314547644	Down	0.0517124	0.364043046
432478	8	3	15.52	6.24	-1.314510623	Down	0.1828086	0.618102544
227157	8	3	22.23	8.94	-1.314161212	Down	0.1828086	0.62011837

227094	8	3	15.66	6.3	-1.313660479	Down	0.1828086	0.619108816
234839	8	3	6.48	2.61	-1.311944006	Down	0.1828086	0.620371273
14792	101	38	271.54	109.53	-1.30983866	Down	5.39E-07	4.48E-05
69900	7.53	2.84	15.92	6.44	-1.305707742	Down	0.136159	0.573660273
54151	6	2	28.42	11.55	-1.299013703	Down	0.216528	0.678873206
12813	29	11	61.96	25.2	-1.29791341	Down	0.00847668	0.131349077
19018	21	8	187.24	76.41	-1.293055305	Down	0.0266594	0.253233867
56631	13	5	51.5	21.23	-1.278468061	Down	0.088496	0.448462373
66629	13	5	32.88	13.56	-1.277853121	Down	0.088496	0.448735659
68625	13	5	22.33	9.21	-1.27771019	Down	0.088496	0.448189419
17754	31	12	17.84	7.4	-1.269518439	Down	0.0074231	0.11970468
226971	7.08	2.74	17.01	7.07	-1.266601021	Down	0.136159	0.573369959
65973	18	16	41.41	17.22	-1.26589406	Down	0.897186	0.972194909
170789	18	7	106.13	44.23	-1.262735376	Down	0.044735	0.33595662
232807	36	14	81.01	33.78	-1.261930679	Down	0.00397554	0.080881341
237823	6.84	2.66	7.16	2.99	-1.259814103	Down	0.216528	0.682214876
17069	10	4	30.36	12.68	-1.259617045	Down	0.1539756	0.539238623
54383	2	1	4.33	1.81	-1.258377328	Down	0.678286	0.79269913
13804	64	25	426.25	178.42	-1.256422491	Down	0.000118348	0.004875117
52250	23	9	40.34	16.93	-1.252629111	Down	0.0231696	0.238902406
15369	33	13	179.11	75.62	-1.244006133	Down	0.00648604	0.110143549
12517	33	13	170.46	71.97	-1.243965674	Down	0.00648604	0.110368791
56449	104	41	425.98	180	-1.24278879	Down	1.05E-06	7.86E-05
98170	38	15	71.83	30.4	-1.240515192	Down	0.00347958	0.071845125
14218	5	2	3.2	1.37	-1.223896012	Down	0.337326	0.688638284
77853	5	2	6.82	2.92	-1.22380337	Down	0.337326	0.686113333

320817	5	2	4.11	1.76	-1.223562965	Down	0.337326	0.689484069
229714	5	2	10.62	4.55	-1.222845316	Down	0.337326	0.690841655
209361	5	2	6.93	2.97	-1.222392421	Down	0.337326	0.685610563
386454	5	2	23.52	10.08	-1.222392421	Down	0.337326	0.69101173
66528	5	2	36.05	15.45	-1.222392421	Down	0.337326	0.687289335
15331	10	4	54.45	23.34	-1.222127488	Down	0.1539756	0.537654623
69627	10	4	51.86	22.23	-1.222114258	Down	0.1539756	0.53720376
69156	10	4	30.07	12.89	-1.222072604	Down	0.1539756	0.539011766
94254	5	2	14.58	6.25	-1.222062625	Down	0.337326	0.689145506
70681	5	2	13.46	5.77	-1.222035186	Down	0.337326	0.689992538
16476	5	2	10.52	4.51	-1.221935366	Down	0.337326	0.684941348
213389	5	2	9.68	4.15	-1.221895711	Down	0.337326	0.689653476
69253	5	2	37.95	16.27	-1.221885635	Down	0.337326	0.686952924
70918	5	2	9.19	3.94	-1.221869232	Down	0.337326	0.690331935
76178	10	4	17.33	7.43	-1.221837539	Down	0.1539756	0.536528881
94067	10	4	56.35	24.16	-1.221795156	Down	0.1539756	0.5387851
72947	20	8	71.3	30.57	-1.221785524	Down	0.0385982	0.302425256
623661	10	4	43.66	18.72	-1.221731695	Down	0.1539756	0.536978612
11504	5	2	6.81	2.92	-1.221686429	Down	0.337326	0.685945661
104570	5	2	6.53	2.8	-1.221656165	Down	0.337326	0.690501758
107173	45	18	174.91	75	-1.221650273	Down	0.001644244	0.041459862
69151	5	2	19.03	8.16	-1.221634504	Down	0.337326	0.690162195
69257	5	2	6.11	2.62	-1.221605568	Down	0.337326	0.684774249
67983	10	4	42	18.01	-1.221591147	Down	0.1539756	0.537429097
56293	5	2	22.48	9.64	-1.221536984	Down	0.337326	0.687121088
77613	10	4	21.92	9.4	-1.221515136	Down	0.1539756	0.538332339

70093	5	2	10.89	4.67	-1.221509499	Down	0.337326	0.687963149
217219	5	2	10.82	4.64	-1.221503789	Down	0.337326	0.685778071
27059	5	2	5.2	2.23	-1.221467913	Down	0.337326	0.688300551
71913	25	10	72.03	30.89	-1.221458033	Down	0.0200914	0.210289987
216148	20	8	33.67	14.44	-1.221392979	Down	0.0385982	0.302140755
20970	10	4	13.43	5.76	-1.221318588	Down	0.1539756	0.536753652
68023	5	2	21.94	9.41	-1.221296898	Down	0.337326	0.686616841
668158	5	2	21.94	9.41	-1.221296898	Down	0.337326	0.68510853
109267	5	2	12.31	5.28	-1.221220927	Down	0.337326	0.689822965
72542	5	2	16.25	6.97	-1.221209157	Down	0.337326	0.686281087
12400	5	2	12.1	5.19	-1.221200604	Down	0.337326	0.689314746
73390	5	2	15.76	6.76	-1.221172383	Down	0.337326	0.687794571
64138	5	2	23.38	10.03	-1.220953324	Down	0.337326	0.690671665
226525	10	4	6.62	2.84	-1.220940287	Down	0.1539756	0.537880339
14972	5	2	20.3	8.71	-1.220735103	Down	0.337326	0.687457665
319518	5	2	11.56	4.96	-1.220729372	Down	0.337326	0.685443137
233208	5	2	7.97	3.42	-1.220583399	Down	0.337326	0.686784841
330812	15	6	10.3	4.42	-1.220526063	Down	0.075736	0.410553261
55946	5	2	7.55	3.24	-1.220482831	Down	0.337326	0.688807275
22062	5	2	7.48	3.21	-1.220464973	Down	0.337326	0.685275792
53419	5	2	7.06	3.03	-1.22035039	Down	0.337326	0.691181888
14344	5	2	11.3	4.85	-1.22026612	Down	0.337326	0.691692865
18483	5	2	13.35	5.73	-1.220232698	Down	0.337326	0.691863359
15186	5	2	13.93	5.98	-1.219977868	Down	0.337326	0.684607231
21873	5	2	7.22	3.1	-1.219730622	Down	0.337326	0.688469376
229055	10	4	8.92	3.83	-1.219699318	Down	0.1539756	0.538558624

338355	5	2	7.66	3.29	-1.219256808	Down		0.337326	0.688131808
666173	15	6	7.45	3.2	-1.21916852	Down		0.075736	0.410820897
76055	5	2	6.96	2.99	-1.218941822	Down		0.337326	0.69135213
329908	5	2	3.14	1.35	-1.217805152	Down		0.337326	0.688976349
71453	7.47	3	62.45	26.85	-1.217779484	Down		0.274784	0.715642461
57752	5	2	3.65	1.57	-1.217131905	Down		0.337326	0.687626077
13400	10	4	25.48	10.99	-1.213173891	Down		0.1539756	0.538106244
56403	2	2	4.83	2.11	-1.19478019	Down		0.93456	0.97878839
19944	22	9	216.49	94.87	-1.190276534	Down		0.0332504	0.287904868
75620	22	9	121.6	53.31	-1.189665142	Down		0.0332504	0.287605591
70484	12.2	5	36.11	15.87	-1.186096292	Down		0.1297564	0.552560391
192950	29	12	39.41	17.48	-1.172856565	Down		0.01504346	0.18544686
17764	106	44	92.5	41.17	-1.167859917	Down		2.67E-06	0.000188076
104111	12	5	18.45	8.24	-1.162904574	Down		0.1297564	0.553126539
20775	12	5	29.33	13.1	-1.162810259	Down		0.1297564	0.552277752
16324	12	5	18.85	8.42	-1.162672385	Down		0.1297564	0.55284332
271377	36	15	48.08	21.48	-1.162442903	Down		0.00693464	0.114946493
19743	1.82	0.76	5.28	2.37	-1.15565087	Down		0.53554	0.806265305
415115	31	13	183.59	82.51	-1.153846592	Down		0.01299992	0.164896851
67446	31	13	141.29	63.51	-1.153603685	Down		0.01299992	0.164645867
71069	19	8	12.07	5.45	-1.147097541	Down		0.0553052	0.355362602
56294	19	8	31.09	14.04	-1.146907681	Down		0.0553052	0.355637225
68038	90	38	151.22	68.46	-1.143315762	Down		2.14E-05	0.001142036
20166	22	10	51.02	23.1	-1.143170047	Down		0.056436	0.354150796
13430	26	11	56.62	25.68	-1.140666547	Down		0.0246148	0.252552097
69104	442.25	187.93	719.04	327.61	-1.134092635	Down		3.66E-21	1.52E-18

574402	80	34	102.5	46.71	-1.133820559	Down	6.97E-05	0.003206086
230393	40	17	46.03	20.98	-1.133559765	Down	0.00520428	0.098420032
214763	7.01	3	11.46	5.26	-1.123472339	Down	0.274784	0.712742414
19921	7	3	65.31	29.98	-1.123303525	Down	0.274784	0.714747629
13134	7	3	8.8	4.04	-1.123148231	Down	0.274784	0.712298338
100102	7	3	13.35	6.13	-1.122880763	Down	0.274784	0.71296466
21357	7	3	31.01	14.24	-1.12278438	Down	0.274784	0.713187044
20931	7	3	40.94	18.8	-1.12277844	Down	0.274784	0.710969423
101543	7	3	24.15	11.09	-1.122763824	Down	0.274784	0.714301051
16011	7	3	7.97	3.66	-1.122736076	Down	0.274784	0.713409568
66899	7	3	15.94	7.32	-1.122736076	Down	0.274784	0.711190564
66830	7	3	10.8	4.96	-1.122619287	Down	0.274784	0.714971127
98432	7	3	7.62	3.5	-1.122436076	Down	0.274784	0.712076507
22187	7	3	39.58	18.18	-1.122419413	Down	0.274784	0.71363223
260299	7	3	21.9	10.06	-1.122300565	Down	0.274784	0.71586652
330171	7	3	15.17	6.97	-1.121990524	Down	0.274784	0.71163326
229503	161	69	379.7	174.46	-1.121963704	Down	2.00E-08	2.10E-06
207375	7	3	6.05	2.78	-1.121850259	Down	0.274784	0.71452427
17188	7	3	19.76	9.08	-1.121818744	Down	0.274784	0.713855031
12616	14	6	33.25	15.28	-1.121709797	Down	0.1095002	0.481326553
29876	21	9	34.55	15.88	-1.121474798	Down	0.0472524	0.352318298
57753	7	3	11.77	5.41	-1.121413821	Down	0.274784	0.716315058
105787	7	3	10.05	4.62	-1.121230745	Down	0.274784	0.711854814
27221	7	3	14.18	6.52	-1.120913663	Down	0.274784	0.712520307
12217	37	16	15.41	7.14	-1.109870882	Down	0.00836766	0.133130591
19652	8	4	20.86	9.67	-1.109151363	Down	0.325592	0.689026203

19646	74	32	112.22	52.03	-1.108914205	Down	0.000173195	0.006765989
16647	23	10	74.94	34.93	-1.101269335	Down	0.0403838	0.313756863
208263	23	10	37.2	17.34	-1.101198723	Down	0.0403838	0.314050093
66874	30.8	13.41	32.68	15.25	-1.099598741	Down	0.0182003	0.206047206
252966	34	15	77.48	36.65	-1.080010756	Down	0.01345812	0.169417574
73728	34	15	57.58	27.24	-1.079841086	Down	0.01345812	0.169161656
21957	16	7	101.82	48.17	-1.079814142	Down	0.0925584	0.464242584
240832	199.23	88.38	226.85	107.91	-1.071910094	Down	1.55E-09	2.08E-07
330938	9	4	10.67	5.08	-1.070659774	Down	0.226072	0.631044989
57912	9	4	19.3	9.19	-1.070464081	Down	0.226072	0.628935176
100072	11	5	8.86	4.22	-1.0700637	Down	0.1873432	0.621812033
29817	9	4	55.07	26.23	-1.070048801	Down	0.226072	0.629145522
71706	9	4	25.02	11.92	-1.069697554	Down	0.226072	0.631256749
84113	9	4	32.07	15.28	-1.06957981	Down	0.226072	0.629777406
105450	27	12	45.9	21.87	-1.069540933	Down	0.0295372	0.267441829
16162	18	8	64.03	30.51	-1.06946583	Down	0.078367	0.421247937
80889	27	12	46.96	22.38	-1.069222372	Down	0.0295372	0.267151132
12769	9	4	13.91	6.63	-1.069041644	Down	0.226072	0.628724971
107271	9	4	21.02	10.02	-1.068880161	Down	0.226072	0.631680696
208198	9	4	19.95	9.51	-1.0688715	Down	0.226072	0.630199368
24136	9	4	6.48	3.09	-1.068386975	Down	0.226072	0.628514905
78266	9	4	12.98	6.19	-1.068279069	Down	0.226072	0.629988316
208211	47	21	179.53	85.98	-1.062151927	Down	0.00400596	0.08090678
16202	47	21	181.62	86.99	-1.062001611	Down	0.00400596	0.080710879
66302	38	17	137.02	65.71	-1.060201643	Down	0.00995778	0.147698195
338348	47	21	119.5	57.36	-1.058893689	Down	0.00400596	0.081103633

75894	7	3	19.03	9.14	-1.058009491	Down		0.274784	0.711411843
66471	2	1	4.62	2.22	-1.057333175	Down		0.678286	0.804220263
26451	78	35	480.84	231.21	-1.056353119	Down		0.000210774	0.00808226
329559	40	18	57.97	27.97	-1.051426232	Down		0.00856822	0.132274877
12831	20	9	15.81	7.63	-1.051082406	Down		0.0664554	0.408401317
22040	9	4	57.51	27.77	-1.050285661	Down		0.226072	0.629356009
14688	9	4	19.22	9.31	-1.045755263	Down		0.226072	0.630621895
216227	11.1	5.02	19.28	9.34	-1.045610597	Down		0.1873432	0.62280574
213989	26.26	11.87	85.76	41.56	-1.045109252	Down		0.0246148	0.252241072
55981	126	57	387.3	187.83	-1.044023994	Down		2.88E-06	0.000199991
110595	11	5	52.86	25.75	-1.037603993	Down		0.1873432	0.622060162
225182	11	5	20.36	9.92	-1.037325536	Down		0.1873432	0.62230849
378937	22	10	78.09	38.05	-1.037241359	Down		0.056436	0.35441808
13124	11	5	38.11	18.57	-1.037195793	Down		0.1873432	0.622557016
71986	11	5	39.75	19.37	-1.037130906	Down		0.1873432	0.623553107
74841	22	10	31.19	15.2	-1.037012229	Down		0.056436	0.353883916
18707	7	3	9.52	4.64	-1.036836768	Down		0.274784	0.715194765
78926	2	1	5.17	2.52	-1.036740547	Down		0.678286	0.789042053
233905	11	5	11.59	5.65	-1.036557794	Down		0.1873432	0.623303785
227723	11	5	8.55	4.17	-1.035877036	Down		0.1873432	0.623054663
319146	4.96	2.26	23.09	11.28	-1.033501105	Down		0.512406	0.819790488
20409	12.07	5.52	47.7	23.38	-1.028714336	Down		0.1297564	0.553410048
13542	9	4	17.43	8.55	-1.027576244	Down		0.226072	0.629566637
71950	67.28	31.01	226.67	111.99	-1.017223547	Down		0.000924166	0.026426066
56213	13	6	42.78	21.16	-1.015596855	Down		0.1561068	0.542818505
105663	13	6	30.75	15.21	-1.015566257	Down		0.1561068	0.543045436

229007	12	5	35.31	17.52	-1.011074046	Down		0.1297564	0.553693848
19106	38.51	17.87	59.26	29.49	-1.006832804	Down		0.00995778	0.147435387
74126	28	13	54.05	26.91	-1.006152227	Down		0.0348272	0.298452246
234374	148	69	474.52	237.16	-1.000608193	Down		9.59E-07	7.32E-05
20851	2.13	1	2.9	1.45	-1	Down		0.678286	0.802276874
107999	15	7	63	31.51	-0.999542074	Down		0.1306474	0.555218088
668173	15	7	61.74	30.89	-0.999065612	Down		0.1306474	0.555501796
353170	12	6	23.02	11.55	-0.994994982	Down		0.219154	0.683500912
330192	17	8	45.52	22.96	-0.987377916	Down		0.1097288	0.48156822
14874	17	8	57.81	29.16	-0.987328353	Down		0.1097288	0.481822346
21754	17	8	31.44	15.86	-0.987208447	Down		0.1097288	0.480807449
225908	17	8	20.2	10.19	-0.987201241	Down		0.1097288	0.48207674
71978	34	16	56.04	28.27	-0.987185073	Down		0.0216922	0.224503478
546071	17	8	21.27	10.73	-0.987169957	Down		0.1097288	0.481060772
71254	51	24	107.77	54.38	-0.986807574	Down		0.00471758	0.092364666
84585	17	8	26.23	13.24	-0.986314685	Down		0.1097288	0.481314362
110332	87	41	236.39	119.43	-0.985003729	Down		0.000216016	0.008207622
330460	19	9	45.18	22.94	-0.977818879	Down		0.0924322	0.463889226
232934	19	9	58.54	29.73	-0.977503283	Down		0.0924322	0.464169183
328059	7	4	26.62	13.52	-0.97741542	Down		0.458738	0.843384644
59041	38	18	78.28	39.76	-0.977325999	Down		0.01588576	0.194104859
71602	24.38	11.6	32.93	16.79	-0.971800282	Down		0.0479912	0.356867538
56094	4.3	2.04	11.59	5.91	-0.971650531	Down		0.512406	0.823113963
77087	21	10	16.03	8.18	-0.970601677	Down		0.0780566	0.419850658
545192	21	10	30.2	15.42	-0.969745784	Down		0.0780566	0.420122231
12777	23	11	90.3	46.29	-0.964025425	Down		0.0660582	0.407163172

224650	23	11	26.34	13.51	-0.963227671	Down	0.0660582	0.406861793
237928	27	13	95.53	49.31	-0.954073612	Down	0.0475652	0.354016126
71508	8.4	4.04	23.17	11.96	-0.954040655	Down	0.325592	0.688675911
16495	27	13	51.76	26.72	-0.95391761	Down	0.0475652	0.354333061
69926	29	14	12.94	6.7	-0.949604617	Down	0.0404534	0.314004423
18166	25.49	12.4	56.89	29.66	-0.939656483	Down	0.0560084	0.359603315
94064	8.92	4.35	74.75	39.03	-0.937490117	Down	0.325592	0.689727859
223332	12.24	6	19.03	10.01	-0.926833587	Down	0.219154	0.684013666
66965	246	121	538.22	283.83	-0.923168916	Down	3.29E-09	4.08E-07
19285	63	31	131.25	69.24	-0.922639793	Down	0.00293268	0.061936117
54135	28	14	94.59	49.94	-0.921491849	Down	0.0546016	0.352201483
225131	4	2	5.36	2.83	-0.921430948	Down	0.512406	0.823272896
239463	24.36	12.03	78.67	41.66	-0.917150744	Down	0.0760384	0.411924171
237436	82.91	40.96	96	50.85	-0.916786632	Down	0.000584398	0.019220458
194388	2	1	1.22	0.65	-0.908369525	Down	0.678286	0.805252933
16440	2	1	1.48	0.79	-0.905672617	Down	0.678286	0.791365368
75788	2	1	2.51	1.34	-0.905454363	Down	0.678286	0.800002524
80750	2	1	2.06	1.1	-0.905140814	Down	0.678286	0.803304555
74376	4	2	3.22	1.72	-0.904652123	Down	0.512406	0.825664277
320808	2	1	2.34	1.25	-0.904580435	Down	0.678286	0.794708224
17158	2	1	2.19	1.17	-0.90442234	Down	0.678286	0.800342854
239555	2	1	2.62	1.4	-0.904139985	Down	0.678286	0.796727528
22770	2	1	2.62	1.4	-0.904139985	Down	0.678286	0.789814974
71592	2	1	1.89	1.01	-0.904030941	Down	0.678286	0.806633958
68709	2	1	3.33	1.78	-0.903644936	Down	0.678286	0.791032629
230857	2	1	2.75	1.47	-0.903615464	Down	0.678286	0.794037395

268448	2	1	3.03	1.62	-0.903323981	Down		0.678286	0.796615075
23966	2	1	1.01	0.54	-0.903323981	Down		0.678286	0.788931759
18779	2	1	1.87	1	-0.90303827	Down		0.678286	0.794260879
22222	2	1	1.72	0.92	-0.902702799	Down		0.678286	0.801593212
171211	4	2	3.29	1.76	-0.902512155	Down		0.512406	0.824865608
102693	2	1	2.43	1.3	-0.902444691	Down		0.678286	0.799322731
22240	2	1	2.43	1.3	-0.902444691	Down		0.678286	0.797065076
106759	2	1	4.71	2.52	-0.902303326	Down		0.678286	0.789925515
94186	2	1	3.42	1.83	-0.902152677	Down		0.678286	0.800569902
18441	2	1	3.7	1.98	-0.90202484	Down		0.678286	0.806057956
53319	2	1	5.83	3.12	-0.901949854	Down		0.678286	0.793925701
75456	2	1	8.09	4.33	-0.901772678	Down		0.678286	0.788270643
67245	2	1	3.83	2.05	-0.901720482	Down		0.678286	0.798983268
270198	4	2	7.94	4.25	-0.901676166	Down		0.512406	0.820105852
12614	4	2	2.41	1.29	-0.901662081	Down		0.512406	0.820263626
75580	2	1	1.7	0.91	-0.901596296	Down		0.678286	0.790810958
20969	2	1	4.39	2.35	-0.901560183	Down		0.678286	0.791254424
19951	2	1	28.32	15.16	-0.901551512	Down		0.678286	0.797628294
80904	2	1	6.65	3.56	-0.901477099	Down		0.678286	0.801024383
56208	2	1	6.65	3.56	-0.901477099	Down		0.678286	0.800115935
230972	2	1	4.95	2.65	-0.901436166	Down		0.678286	0.79909639
24075	2	1	17.67	9.46	-0.901389951	Down		0.678286	0.798305206
67772	4	2	3.25	1.74	-0.901352412	Down		0.512406	0.822637532
66171	2	1	15.67	8.39	-0.901262464	Down		0.678286	0.79943595
14685	2	1	6.07	3.25	-0.901256798	Down		0.678286	0.798192307
66932	2	1	2.54	1.36	-0.901221846	Down		0.678286	0.795716595

50762	2	1	6.91	3.7	-0.90116044	Down	0.678286	0.80399114
12492	2	1	2.82	1.51	-0.901146613	Down	0.678286	0.805482775
71779	4	2	6.2	3.32	-0.901084974	Down	0.512406	0.823750063
170721	2	1	3.1	1.66	-0.901084974	Down	0.678286	0.792921861
243834	2	1	3.1	1.66	-0.901084974	Down	0.678286	0.79281048
22083	2	1	3.1	1.66	-0.901084974	Down	0.678286	0.790921778
214854	2	1	5.21	2.79	-0.90101825	Down	0.678286	0.801138085
103136	2	1	5.21	2.79	-0.90101825	Down	0.678286	0.791143511
66940	4	2	16.75	8.97	-0.900981205	Down	0.512406	0.822161652
110902	4	2	7.6	4.07	-0.900970624	Down	0.512406	0.821844704
75219	4	2	5.77	3.09	-0.900964481	Down	0.512406	0.825344624
57755	2	1	11.54	6.18	-0.900964481	Down	0.678286	0.790367989
17165	2	1	6.05	3.24	-0.900941329	Down	0.678286	0.797853804
110279	8	4	8.16	4.37	-0.900935872	Down	0.325592	0.689376853
76429	2	1	8.44	4.52	-0.900920226	Down	0.678286	0.789704464
280408	2	1	9	4.82	-0.900891855	Down	0.678286	0.802048857
72465	2	1	4.5	2.41	-0.900891855	Down	0.678286	0.788380752
16598	2	1	7.45	3.99	-0.900851679	Down	0.678286	0.788711264
216724	2	1	5.06	2.71	-0.900844533	Down	0.678286	0.798870178
71862	2	1	8.01	4.29	-0.900824595	Down	0.678286	0.790257324
56429	2	1	8.29	4.44	-0.900812425	Down	0.678286	0.798644093
69379	2	1	16.86	9.03	-0.900806643	Down	0.678286	0.800910715
65112	2	1	2.95	1.58	-0.900790396	Down	0.678286	0.798757119
387609	6	3	9.13	4.89	-0.900780395	Down	0.404054	0.78389679
26450	2	1	6.18	3.31	-0.900775621	Down	0.678286	0.80250502
56199	4	2	6.18	3.31	-0.900775621	Down	0.512406	0.822320217

58227	2	1	3.23	1.73	-0.900762127	Down	0.678286	0.795380187
407800	6.01	3	11.09	5.94	-0.900724529	Down	0.404054	0.785545171
66271	4	2	36.35	19.47	-0.90070248	Down	0.512406	0.825504419
11470	2	1	8.98	4.81	-0.900678551	Down	0.678286	0.804449516
13198	6	3	49.51	26.52	-0.900639174	Down	0.404054	0.785361676
67278	2	1	9.39	5.03	-0.900566758	Down	0.678286	0.797290268
67099	2	1	5.04	2.7	-0.900464326	Down	0.678286	0.807095353
67200	2	1	5.04	2.7	-0.900464326	Down	0.678286	0.806518692
19386	2	1	1.4	0.75	-0.900464326	Down	0.678286	0.804908415
66083	2	1	8.12	4.35	-0.900464326	Down	0.678286	0.796390265
244911	6	3	30.24	16.2	-0.900464326	Down	0.404054	0.784994941
20826	6	3	32.59	17.46	-0.900375793	Down	0.404054	0.78867777
211286	6	3	16.5	8.84	-0.90034775	Down	0.404054	0.784628549
73603	2	1	14.82	7.94	-0.900334535	Down	0.678286	0.801707075
109032	6	3	21.11	11.31	-0.900327649	Down	0.404054	0.788862819
110213	2	1	5.45	2.92	-0.900287861	Down	0.678286	0.80686459
54140	4	2	10.06	5.39	-0.900273127	Down	0.512406	0.82343189
232440	8	4	29.62	15.87	-0.900269512	Down	0.325592	0.688500898
17179	4	2	14.67	7.86	-0.900267653	Down	0.512406	0.818060308
18022	2	1	9.22	4.94	-0.900255709	Down	0.678286	0.801479382
381668	2	1	4.33	2.32	-0.90024222	Down	0.678286	0.806403458
68226	8	4	21.09	11.3	-0.900236322	Down	0.325592	0.687278293
226830	2	1	8.38	4.49	-0.900234799	Down	0.678286	0.794932085
23927	2	1	16.2	8.68	-0.900226865	Down	0.678286	0.799662483
330216	6	3	31.28	16.76	-0.900218364	Down	0.404054	0.787938442
68017	4	2	18.85	10.1	-0.900209231	Down	0.512406	0.821053404

93841	16	8	94.25	50.5	-0.900209231	Down	0.1516874	0.532345363
16367	10	5	7.26	3.89	-0.900199393	Down	0.265898	0.694674178
231474	2	1	9.91	5.31	-0.900173196	Down	0.678286	0.8046789
13191	2	1	3.21	1.72	-0.900164732	Down	0.678286	0.797966606
19042	4	2	9.63	5.16	-0.900164732	Down	0.512406	0.817589708
70314	2	1	5.3	2.84	-0.90010143	Down	0.678286	0.799775798
382073	4	2	23.29	12.48	-0.900092705	Down	0.512406	0.82518489
71452	4	2	7.67	4.11	-0.900088184	Down	0.512406	0.819475365
73062	2	1	5.02	2.69	-0.900081191	Down	0.678286	0.79459634
97287	2	1	5.02	2.69	-0.900081191	Down	0.678286	0.791698388
229599	6	3	29.28	15.69	-0.900070201	Down	0.404054	0.787753827
74855	2	1	4.74	2.54	-0.900058562	Down	0.678286	0.803876628
66882	4	2	9.48	5.08	-0.900058562	Down	0.512406	0.818217295
319601	10	5	30.81	16.51	-0.900058562	Down	0.265898	0.695547708
26403	4	2	6.83	3.66	-0.90004193	Down	0.512406	0.823590946
213956	2	1	4.46	2.39	-0.900033092	Down	0.678286	0.79014669
245020	4	2	17	9.11	-0.900011787	Down	0.512406	0.820895327
20216	16	8	42.08	22.55	-0.900007271	Down	0.1516874	0.532569981
13590	2	1	8.36	4.48	-0.90000421	Down	0.678286	0.805942854
17938	2	1	12.26	6.57	-0.899993703	Down	0.678286	0.805023222
212937	4	2	7.8	4.18	-0.899971182	Down	0.512406	0.825025218
23790	2	1	3.9	2.09	-0.899971182	Down	0.678286	0.796053287
226849	8	4	17.41	9.33	-0.899967217	Down	0.325592	0.687452685
71207	10	5	20.75	11.12	-0.899954548	Down	0.265898	0.696423437
27979	8	4	18.1	9.7	-0.899933045	Down	0.325592	0.689903497
67150	2	1	14.48	7.76	-0.899933045	Down	0.678286	0.80617309

244723	4	2	17.82	9.55	-0.899924699	Down	0.512406	0.821211542
15193	12	6	35.36	18.95	-0.899920426	Down	0.219154	0.683757193
16670	10	5	40.51	21.71	-0.899918359	Down	0.265898	0.695329119
387524	2	1	5.15	2.76	-0.899904165	Down	0.678286	0.802733296
432940	4	2	18.23	9.77	-0.899884094	Down	0.512406	0.822796281
214951	6	3	26.16	14.02	-0.899876191	Down	0.404054	0.789047954
50926	2	1	4.87	2.61	-0.899871966	Down	0.678286	0.806749258
57330	10	5	12.24	6.56	-0.899835838	Down	0.265898	0.694456139
11740	4	2	20.71	11.1	-0.899767877	Down	0.512406	0.818688619
12040	8	4	38.36	20.56	-0.899762456	Down	0.325592	0.689552312
76954	14	7	31.27	16.76	-0.89975707	Down	0.181852	0.617628772
171429	2	1	4.03	2.16	-0.899748526	Down	0.678286	0.803647701
333307	6	3	16.12	8.64	-0.899748526	Down	0.404054	0.788123144
13824	10	5	17.37	9.31	-0.899744681	Down	0.265898	0.694892355
76832	10	5	41.27	22.12	-0.899742053	Down	0.265898	0.696204298
271457	6	3	17.09	9.16	-0.899732894	Down	0.404054	0.784811703
52670	4	2	19.59	10.5	-0.89972807	Down	0.512406	0.824227784
11482	4	2	7.5	4.02	-0.899695094	Down	0.512406	0.823909242
78779	8	4	22.63	12.13	-0.899657034	Down	0.325592	0.688151138
14450	10	5	20.13	10.79	-0.899652307	Down	0.265898	0.695985297
67049	6	3	20.13	10.79	-0.899652307	Down	0.404054	0.784445482
26572	8	4	34.29	18.38	-0.899651138	Down	0.325592	0.689201484
75612	2	1	3.47	1.86	-0.899633041	Down	0.678286	0.807557277
21414	4	2	10.41	5.58	-0.899633041	Down	0.512406	0.824387147
240505	2	1	2.22	1.19	-0.899598103	Down	0.678286	0.79158735
192120	4	2	15.54	8.33	-0.899598103	Down	0.512406	0.821528001

56772	14	7	37.46	20.08	-0.89959163	Down	0.181852	0.617880969
54216	18	9	21.64	11.6	-0.899575694	Down	0.127053	0.54299333
70717	6	3	14.01	7.51	-0.899572143	Down	0.404054	0.78591242
224432	6	3	9.57	5.13	-0.899560099	Down	0.404054	0.787384856
12469	10	5	28.15	15.09	-0.899542116	Down	0.265898	0.695110669
19826	2	1	8.32	4.46	-0.899539818	Down	0.678286	0.798079441
13017	8	4	17.61	9.44	-0.899536144	Down	0.325592	0.688325974
56484	4	2	9.29	4.98	-0.899532854	Down	0.512406	0.817903381
66975	8	4	15.11	8.1	-0.899509847	Down	0.325592	0.687976392
56334	6	3	19.96	10.7	-0.899500924	Down	0.404054	0.787569298
109242	28	14	33.26	17.83	-0.899481466	Down	0.0546016	0.35192867
233871	12	6	21.34	11.44	-0.899473124	Down	0.219154	0.684270332
75690	2	1	3.88	2.08	-0.899473124	Down	0.678286	0.804334873
234889	2	1	0.97	0.52	-0.899473124	Down	0.678286	0.78937312
236082	22	11	67.06	35.95	-0.899460712	Down	0.0899996	0.452773078
79044	2	1	15.37	8.24	-0.899400923	Down	0.678286	0.795604427
64704	8	4	31.15	16.7	-0.899384061	Down	0.325592	0.688851012
19212	8	4	15.09	8.09	-0.899381198	Down	0.325592	0.68710399
232086	6	3	26.99	14.47	-0.899360055	Down	0.404054	0.783714064
13629	4	2	8.58	4.6	-0.899343787	Down	0.512406	0.819632896
14674	4	2	4.29	2.3	-0.899343787	Down	0.512406	0.819160485
18029	4	2	4.29	2.3	-0.899343787	Down	0.512406	0.817276275
14163	60	30	96.3	51.63	-0.8993262	Down	0.00450136	0.088757859
319758	2	1	1.66	0.89	-0.899306	Down	0.678286	0.793814037
19878	4	2	3.32	1.78	-0.899306	Down	0.512406	0.818374343
80385	4	2	16.32	8.75	-0.899286135	Down	0.512406	0.821369741

108673	6	3	13.41	7.19	-0.899245561	Down	0.404054	0.786280013
75291	2	1	7.05	3.78	-0.899237023	Down	0.678286	0.795268114
69654	2	1	7.05	3.78	-0.899237023	Down	0.678286	0.795044063
108116	2	1	4.7	2.52	-0.899237023	Down	0.678286	0.793367698
56070	4	2	6.08	3.26	-0.899199359	Down	0.512406	0.819317895
269397	2	1	3.04	1.63	-0.899199359	Down	0.678286	0.79214285
193034	2	1	3.73	2	-0.89917563	Down	0.678286	0.797515587
21968	6	3	17.68	9.48	-0.89915931	Down	0.404054	0.788492808
19053	6	3	22.1	11.85	-0.89915931	Down	0.404054	0.784262499
215449	4	2	13.95	7.48	-0.899154947	Down	0.512406	0.822003147
68799	2	1	5.11	2.74	-0.899147398	Down	0.678286	0.803418905
22612	4	2	5.8	3.11	-0.89913832	Down	0.512406	0.822955091
244550	2	1	6.49	3.48	-0.899131172	Down	0.678286	0.793144717
56448	2	1	4.83	2.59	-0.899071091	Down	0.678286	0.803533287
243755	6	3	11.73	6.29	-0.899071091	Down	0.404054	0.786647949
27632	2	1	9.66	5.18	-0.899071091	Down	0.678286	0.790589411
15284	6	3	18.63	9.99	-0.899071091	Down	0.404054	0.786463938
226162	2	1	10.07	5.4	-0.899032371	Down	0.678286	0.788601063
56374	2	1	8.69	4.66	-0.899026222	Down	0.678286	0.792587812
226180	2	1	5.93	3.18	-0.899005339	Down	0.678286	0.790036087
70120	4	2	17.1	9.17	-0.899002686	Down	0.512406	0.82042146
18536	8	4	6.34	3.4	-0.898948094	Down	0.325592	0.690079224
20853	4	2	7.44	3.99	-0.898913875	Down	0.512406	0.819003136
320982	4	2	6.75	3.62	-0.898897805	Down	0.512406	0.82073731
140499	2	1	4.27	2.29	-0.898888472	Down	0.678286	0.797177656
320404	6	3	7.85	4.21	-0.898872421	Down	0.404054	0.783531423

19718	2	1	8.95	4.8	-0.898853277	Down	0.678286	0.790700169
66300	2	1	7.98	4.28	-0.89877795	Down	0.678286	0.802619142
72512	2	1	6.19	3.32	-0.898756168	Down	0.678286	0.790478684
105246	2	1	5.5	2.95	-0.898716664	Down	0.678286	0.795492291
27967	4	2	7.01	3.76	-0.898681782	Down	0.512406	0.817119649
69697	10	5	15.53	8.33	-0.898669429	Down	0.265898	0.695766433
233552	4	2	7.42	3.98	-0.898650756	Down	0.512406	0.818845847
14462	2	1	4.12	2.21	-0.898597968	Down	0.678286	0.793702406
330863	2	1	1.51	0.81	-0.898554736	Down	0.678286	0.789593985
59079	6	3	6.45	3.46	-0.898527123	Down	0.404054	0.784079602
238057	2	1	9.73	5.22	-0.898389998	Down	0.678286	0.798418136
21335	2	1	5.07	2.72	-0.898379096	Down	0.678286	0.797741033
68077	2	1	8.63	4.63	-0.898348366	Down	0.678286	0.792254044
67453	6	3	9.17	4.92	-0.898263418	Down	0.404054	0.78701623
52521	2	1	4.79	2.57	-0.898257297	Down	0.678286	0.796277907
66857	2	1	6.84	3.67	-0.898216262	Down	0.678286	0.794372668
16826	2	1	5.33	2.86	-0.898120386	Down	0.678286	0.805138061
52428	4	2	7.79	4.18	-0.898120386	Down	0.512406	0.822478844
224088	4	2	3.69	1.98	-0.898120386	Down	0.512406	0.820579355
252870	2	1	2.46	1.32	-0.898120386	Down	0.678286	0.788490892
64383	2	1	8.2	4.4	-0.898120386	Down	0.678286	0.803075954
66435	2	1	2.05	1.1	-0.898120386	Down	0.678286	0.792476524
227298	2	1	5.46	2.93	-0.898000286	Down	0.678286	0.791809457
93686	4	2	3.82	2.05	-0.897948729	Down	0.512406	0.821686322
319513	2	1	3.82	2.05	-0.897948729	Down	0.678286	0.793590805
57342	2	1	3	1.61	-0.897901812	Down	0.678286	0.805712749

67120	2	1	1.77	0.95	-0.897749942	Down	0.678286	0.799889145
14268	4	2	3.54	1.9	-0.897749942	Down	0.512406	0.817746514
234344	2	1	4.49	2.41	-0.897682299	Down	0.678286	0.791476344
16010	2	1	6.52	3.5	-0.897517042	Down	0.678286	0.794820139
192157	2	1	1.9	1.02	-0.897430266	Down	0.678286	0.7995492
74191	2	1	5.29	2.84	-0.897376793	Down	0.678286	0.789483537
56351	2	1	6.91	3.71	-0.897266524	Down	0.678286	0.806979955
68318	2	1	2.57	1.38	-0.897100092	Down	0.678286	0.802390931
12267	2	1	3.11	1.67	-0.897066478	Down	0.678286	0.795156073
67581	2	1	3.65	1.96	-0.89704281	Down	0.678286	0.797402911
223701	2	1	3.24	1.74	-0.896906507	Down	0.678286	0.793033273
192191	2	1	4.45	2.39	-0.896794718	Down	0.678286	0.794149121
107239	4	2	6.74	3.62	-0.896758894	Down	0.512406	0.818531451
26920	2	1	1.75	0.94	-0.89662226	Down	0.678286	0.807441746
20440	2	1	2.96	1.59	-0.89657041	Down	0.678286	0.803762148
81877	6	3	4.17	2.24	-0.896548651	Down	0.404054	0.786096173
58172	2	1	2.42	1.3	-0.896495424	Down	0.678286	0.806288258
76719	2	1	2.42	1.3	-0.896495424	Down	0.678286	0.804793641
214987	2	1	4.56	2.45	-0.896252075	Down	0.678286	0.791920556
21961	2	1	1.34	0.72	-0.896164189	Down	0.678286	0.804105685
217882	2	1	2.01	1.08	-0.896164189	Down	0.678286	0.801365584
231861	6	3	3.87	2.08	-0.895750038	Down	0.404054	0.7872005
94109	2	1	0.93	0.5	-0.895302621	Down	0.678286	0.800683474
110611	4	2	4.24	2.28	-0.89503044	Down	0.512406	0.824068482
94223	2	1	3.16	1.7	-0.894389812	Down	0.678286	0.792031688
225638	2	1	1.84	0.99	-0.894205336	Down	0.678286	0.807326249

320080	2	1	2.23	1.2	-0.894009304	Down	0.678286	0.795941025
235041	2	1	2.75	1.48	-0.893834443	Down	0.678286	0.796502654
209773	2	1	3.01	1.62	-0.893769674	Down	0.678286	0.80767284
320706	6	3	3.01	1.62	-0.893769674	Down	0.404054	0.788307933
319807	2	1	1.56	0.84	-0.893084796	Down	0.678286	0.800456362
74249	23.77	11.96	43.51	23.47	-0.890529175	Down	0.0660582	0.406260371
11990	2	1	1.52	0.82	-0.890375509	Down	0.678286	0.798531099
238328	2	1	2.15	1.16	-0.890211854	Down	0.678286	0.805367838
673094	115	58	834.69	451.06	-0.887921133	Down	9.53E-05	0.004106875
71132	12	7	49.08	26.57	-0.885337021	Down	0.336242	0.689810079
93724	6.34	3.2	8.33	4.51	-0.885189062	Down	0.404054	0.785728753
240064	7.28	3.69	8.06	4.37	-0.883146559	Down	0.274784	0.716090718
53871	89	45	247.19	134.2	-0.881235709	Down	0.000633776	0.020051902
97440	78	39	221.11	120.05	-0.881128871	Down	0.001173618	0.032661122
213988	2	1	0.77	0.42	-0.874469118	Down	0.678286	0.796165581
245474	11.69	6	28.02	15.42	-0.861654191	Down	0.302516	0.651795866
210973	4	2.06	6.92	3.82	-0.8571994	Down	0.512406	0.817432961
14017	6	3	17.45	9.65	-0.854626189	Down	0.404054	0.785178266
23955	221.38	114.24	359.06	198.66	-0.853923526	Down	1.50E-07	1.36E-05
17354	25	13	33.79	18.84	-0.842797386	Down	0.0863056	0.455100696
56314	250.89	130.73	257.81	144.04	-0.83983872	Down	3.33E-08	3.34E-06
54610	155	81	232.71	130.39	-0.835699978	Down	1.62E-05	0.000909249
228839	55	29	127.92	71.97	-0.82977428	Down	0.01174354	0.157609672
69256	37.02	19.45	45.02	25.36	-0.828011311	Down	0.0324694	0.282022837
53414	272	143	472.24	266.2	-0.827009676	Down	1.43E-08	1.55E-06
243382	36.19	19.07	43.42	24.53	-0.823812492	Down	0.0427788	0.326571004

276919	55	29	106.86	60.41	-0.822862631	Down	0.01174354	0.157864291
56187	4.98	2.63	13.16	7.45	-0.820847158	Down	0.512406	0.824706059
19291	36	19	28.26	16	-0.820689561	Down	0.0427788	0.326271673
57266	17	9	62.21	35.3	-0.817478323	Down	0.1724526	0.591499623
214579	17	9	20.08	11.4	-0.816725445	Down	0.1724526	0.59174354
18032	17	9	20.55	11.67	-0.816333833	Down	0.1724526	0.591012391
236266	17	9	11.3	6.42	-0.81567757	Down	0.1724526	0.591255906
70472	8.91	4.73	10.46	5.95	-0.813921278	Down	0.325592	0.687627165
22142	14.44	7.67	61.6	35.06	-0.813104355	Down	0.181852	0.618133371
11630	32	17	28.74	16.37	-0.81200574	Down	0.0590464	0.367758304
27049	2	1	2.44	1.39	-0.811796265	Down	0.678286	0.805597746
269587	32	17	42.16	24.02	-0.811638716	Down	0.0590464	0.368033779
329504	39.51	21	77.71	44.28	-0.81144504	Down	0.0406698	0.315096281
20371	77	41	139.69	79.75	-0.808672322	Down	0.0032283	0.067664192
72123	15	8	24.14	13.8	-0.806757409	Down	0.206826	0.659134104
217057	15	8	34.22	19.57	-0.806196004	Down	0.206826	0.658881756
103710	15	8	32.89	18.81	-0.806149159	Down	0.206826	0.6586296
69757	103	55	313.69	179.57	-0.804793194	Down	0.000681194	0.021071432
382074	3.2	1.73	13.04	7.52	-0.794139303	Down	0.420186	0.79716546
18742	13	7	64.2	37.05	-0.793099755	Down	0.24932	0.676423776
75717	13	7	14.59	8.42	-0.793087745	Down	0.24932	0.676865162
105203	13	7	10.36	5.98	-0.792806613	Down	0.24932	0.676644397
56219	13	7	21.05	12.16	-0.791677005	Down	0.24932	0.67708607
242860	24	13	28.79	16.72	-0.783992941	Down	0.1145576	0.50012266
209815	2	1	5.06	2.94	-0.78332123	Down	0.678286	0.802162849
27215	43	18	63.16	36.72	-0.782445112	Down	0.00323806	0.067698234

110265	7	4	35.85	20.88	-0.779851407	Down	0.458738	0.845252192
19346	46	25	95.09	55.41	-0.779147263	Down	0.0281722	0.265181987
109229	23	11	81.16	47.36	-0.777099784	Down	0.0660582	0.40656086
68948	11	6	74.61	43.61	-0.774710018	Down	0.302516	0.6509531
66556	11	6	75.77	44.29	-0.774645751	Down	0.302516	0.651121479
211936	11	6	18.66	10.91	-0.774297885	Down	0.302516	0.651458498
20530	33	18	123.84	72.41	-0.774216521	Down	0.0657216	0.405388757
232164	11	6	22.14	12.95	-0.773703124	Down	0.302516	0.652302575
320635	11	6	27.13	15.87	-0.773586919	Down	0.302516	0.651964682
237711	11	6	9.06	5.3	-0.773518691	Down	0.302516	0.651627138
14388	15	8	20	11.74	-0.768567592	Down	0.206826	0.658377638
231646	859	471	1239.13	728.5	-0.766326674	Down	3.47E-21	1.52E-18
18704	9.12	5	7.55	4.44	-0.765916968	Down	0.370266	0.741869344
67030	40	22	151.83	89.51	-0.762336107	Down	0.0450408	0.337643691
17713	60	33	120.46	71.03	-0.762053774	Down	0.01370058	0.171949512
68968	40	22	41.76	24.63	-0.761705084	Down	0.0450408	0.337948149
57265	20	11	36.91	21.77	-0.76167033	Down	0.1616202	0.560350702
67703	9	5	17.59	10.4	-0.758171954	Down	0.370266	0.741333827
22173	825	455.81	933.45	553	-0.755293266	Down	4.71E-20	1.78E-17
72836	47	26	89.96	53.36	-0.753524995	Down	0.0310886	0.270877739
225888	74.85	41.19	151.46	89.97	-0.751420906	Down	0.0067534	0.112615313
11816	54	31	305.6	181.86	-0.748816285	Down	0.0299996	0.270451432
319965	9	5	18.36	10.93	-0.748272658	Down	0.370266	0.741690753
16828	9	5	36.73	21.87	-0.748005676	Down	0.370266	0.742226785
66596	18	10	93.34	55.58	-0.747929633	Down	0.1928324	0.620478886
63872	9	5	39.95	23.79	-0.747840231	Down	0.370266	0.741512247

104662	45	25	88.81	52.9	-0.747454411	Down	0.0362956	0.307865125
17847	99	55	53.94	32.13	-0.747434541	Down	0.00178228	0.044269707
78749	9	5	18.55	11.05	-0.747372817	Down	0.370266	0.742584571
57435	27	15	31.29	18.64	-0.747299799	Down	0.1074622	0.516874547
268739	9	5	10.96	6.53	-0.747092901	Down	0.370266	0.742763594
225055	9	5	15	8.94	-0.746615764	Down	0.370266	0.740977245
68112	2	1	6.14	3.66	-0.746395007	Down	0.678286	0.800229378
68222	43	24	260.71	155.94	-0.741454881	Down	0.042404	0.324304857
72946	95	53	187.48	112.14	-0.741435725	Down	0.00237938	0.055771327
21399	26.44	14.77	65.22	39.04	-0.740361388	Down	0.096798	0.482598058
14660	44.52	24.89	59.76	35.82	-0.738414811	Down	0.0329164	0.285607262
381318	89.55	50.12	202.13	121.3	-0.736703911	Down	0.00367342	0.075472908
272027	453.95	254.68	815.49	490.54	-0.733296403	Down	3.96E-11	6.86E-09
14964	7.17	4.02	27.96	16.82	-0.733186655	Down	0.458738	0.845439402
321019	16	9	36.5	22.01	-0.729737319	Down	0.230942	0.641625503
110959	16	9	56.1	33.83	-0.729697594	Down	0.230942	0.641197325
11732	16	9	30.52	18.41	-0.729265335	Down	0.230942	0.641411342
382034	32	18	32.67	19.71	-0.729038678	Down	0.0852994	0.450365677
20416	2	1	3.75	2.27	-0.724198298	Down	0.678286	0.804564192
230161	246	139	325.33	197.1	-0.722976089	Down	1.59E-06	0.000116408
217431	58	32.79	129.55	78.51	-0.722560685	Down	0.01593382	0.194406622
76080	53	30	79.97	48.54	-0.720284779	Down	0.0275138	0.261051687
320587	103.71	58.81	200.15	121.68	-0.717989558	Down	0.001839872	0.045429006
16950	81	46	133.8	81.47	-0.715737303	Down	0.00656614	0.109933302
67951	44	25	164.74	100.34	-0.715294051	Down	0.0464668	0.346771518
100502841	6.33	3.6	4.36	2.66	-0.712901889	Down	0.404054	0.786832046

13079	72	41	250.87	153.14	-0.712088796	Down	0.01082264	0.150593959
67040	3	2	4.93	3.01	-0.71182416	Down	0.754082	0.855332105
331188	7	4	11.45	7.01	-0.707861249	Down	0.458738	0.843944041
67115	7	4	51.66	31.63	-0.707754088	Down	0.458738	0.844878021
226970	7	4	17.18	10.52	-0.707595332	Down	0.458738	0.843757493
209707	14	8	16.51	10.11	-0.707557123	Down	0.27786	0.607640752
56784	7	4	5.65	3.46	-0.70747883	Down	0.458738	0.844130672
18120	7	4	27.53	16.86	-0.707400074	Down	0.458738	0.844504181
70430	14	8	50.73	31.07	-0.707316919	Down	0.27786	0.60796031
69802	14	8	41.76	25.58	-0.707105448	Down	0.27786	0.607162043
11931	7	4	18.17	11.13	-0.707104827	Down	0.458738	0.84469106
70503	7	4	19.28	11.81	-0.707096087	Down	0.458738	0.846189071
16834	14	8	30.69	18.8	-0.707035984	Down	0.27786	0.608120216
68304	28.25	16.14	51.91	31.8	-0.706985722	Down	0.1182524	0.511423191
232491	56	32	175.22	107.34	-0.706979664	Down	0.025807	0.247396368
77799	518	296	1389.26	851.07	-0.706966922	Down	8.24E-12	1.56E-09
15516	14	8	37.56	23.01	-0.706936079	Down	0.27786	0.607481098
20382	21	12	74.58	45.69	-0.706910355	Down	0.1786776	0.609834417
238330	28	16	45.54	27.9	-0.706869169	Down	0.1182524	0.511157517
72053	21	12	66.3	40.62	-0.706818631	Down	0.1786776	0.610084657
216440	7	4	12.06	7.39	-0.706583638	Down	0.458738	0.845065065
11431	7	4	15.11	9.26	-0.706419562	Down	0.458738	0.843198343
16468	7	4	8.24	5.05	-0.70636095	Down	0.458738	0.843012124
12799	7	4	20.2	12.38	-0.706343978	Down	0.458738	0.846001529
76943	7	4	18.47	11.32	-0.706309908	Down	0.458738	0.845626694
68926	7	4	10.67	6.54	-0.706197635	Down	0.458738	0.844317385

239985	14	8	8.22	5.04	-0.70571466	Down	0.27786	0.607321529
269400	7	4	10.8	6.64	-0.701776166	Down	0.458738	0.843571027
67674	99	57	649.57	400.76	-0.696746484	Down	0.00333366	0.069348462
574403	33	19	41	25.31	-0.695916402	Down	0.0934482	0.4670165
26570	7162	4127	5164.43	3191.15	-0.694532703	Down	1.62E-138	6.72E-135
67433	76	37.79	87.94	54.39	-0.693178107	Down	0.0009087	0.026073423
69387	19	11	110.94	68.83	-0.688670216	Down	0.212758	0.675194248
56368	19	11	72.16	44.78	-0.68834481	Down	0.212758	0.674936835
12039	19	11	72.53	45.01	-0.688332284	Down	0.212758	0.674679618
75767	11.62	6.73	12.1	7.51	-0.688122235	Down	0.302516	0.652133584
233912	95	55	165.02	102.44	-0.687861728	Down	0.00443182	0.087594238
66610	817	474	1479.74	920.78	-0.684415305	Down	6.72E-17	2.07E-14
68427	7	4	19.5	12.14	-0.683705702	Down	0.458738	0.84581407
13429	2	1	3.5	2.18	-0.683026787	Down	0.678286	0.788821496
17391	285	166	442.63	276.43	-0.679187041	Down	1.00E-06	7.59E-05
107182	11.68	6.81	9.14	5.71	-0.67870342	Down	0.302516	0.651289945
214685	12	7	31.68	19.81	-0.677343455	Down	0.336242	0.689980193
69171	12	7	29.44	18.41	-0.677288045	Down	0.336242	0.69015039
72323	12	7	38.01	23.77	-0.677237122	Down	0.336242	0.690320672
76854	12	7	31.96	19.99	-0.676988936	Down	0.336242	0.690491037
76448	24	14	50.52	31.6	-0.676930081	Down	0.1650076	0.568306391
12355	5	3	25.79	16.18	-0.672600165	Down	0.578922	0.719093889
66480	17	10	61.9	39.03	-0.665355947	Down	0.254172	0.670353474
66491	24.03	14.14	97.46	61.46	-0.665162456	Down	0.1650076	0.568541714
16882	34	20	37.9	23.91	-0.664583718	Down	0.101731	0.505375314
69721	254.57	149.92	368.91	232.94	-0.663310499	Down	5.24E-06	0.000333012

74256	374.59	220.61	328.27	207.3	-0.663162794	Down	3.77E-08	3.73E-06
76568	6.99	4.12	14.04	8.87	-0.662536926	Down	0.630272	0.764057883
68270	56	33	133.3	84.22	-0.662442	Down	0.0352656	0.300353181
15078	6.78	4	42.86	27.08	-0.662404111	Down	0.630272	0.764837875
103677	39	23	44.71	28.28	-0.660815426	Down	0.080565	0.43139084
58239	22	13	107.44	68.05	-0.658864143	Down	0.1955016	0.628581458
12609	22	13	65.59	41.55	-0.658627398	Down	0.1955016	0.628338669
11435	396.23	234.24	613.08	388.6	-0.657789441	Down	2.00E-08	2.08E-06
18583	14.14	8	14.96	9.49	-0.656630183	Down	0.27786	0.607800489
50708	54	32	519.85	329.98	-0.655716815	Down	0.0409192	0.316439278
171168	9.27	5.49	25.68	16.31	-0.65488842	Down	0.370266	0.741155493
80285	27	16	50.73	32.23	-0.654434972	Down	0.1520998	0.533567637
319154	40.09	23.83	569.96	362.8	-0.651686218	Down	0.0634232	0.39295938
26874	3.42	2.04	4.12	2.63	-0.647581538	Down	0.754082	0.854516726
93705	8.5	5.08	12.22	7.84	-0.640318726	Down	0.504726	0.815975334
268449	15	9	189.75	121.91	-0.638283414	Down	0.304848	0.654954869
71679	10	6	123.21	79.16	-0.638275836	Down	0.409916	0.793604243
12450	5	3	9.57	6.15	-0.637932514	Down	0.578922	0.719846079
19896	10	6	83.56	53.71	-0.637621771	Down	0.409916	0.79286635
170644	10	6	10.33	6.64	-0.637585108	Down	0.409916	0.79231383
67512	5	3	21.56	13.86	-0.637429921	Down	0.578922	0.718664771
67382	5	3	6.33	4.07	-0.637176705	Down	0.578922	0.718986561
328977	5	3	6.05	3.89	-0.637164987	Down	0.578922	0.719416064
18005	5	3	10.42	6.7	-0.637122277	Down	0.578922	0.720384322
22771	5	3	10	6.43	-0.637109357	Down	0.578922	0.717594215
18111	20	12	116.67	75.02	-0.637086472	Down	0.232184	0.643571973

15484	5	3	18.04	11.6	-0.637074533	Down	0.578922	0.720168928
67582	5	3	17.48	11.24	-0.637063149	Down	0.578922	0.720492067
18952	5	3	25.1	16.14	-0.637046786	Down	0.578922	0.718129094
57742	15	9	75.19	48.35	-0.637024912	Down	0.304848	0.655124021
232989	10	6	23.28	14.97	-0.637016837	Down	0.409916	0.793235125
67891	10	6	47.85	30.77	-0.636994481	Down	0.409916	0.792497917
78797	5.01	3	7.34	4.72	-0.636993203	Down	0.578922	0.718879266
26444	5	3	21.32	13.71	-0.636978867	Down	0.578922	0.719953663
381062	10	6	42.95	27.62	-0.636944812	Down	0.409916	0.793788931
30944	5	3	6.22	4	-0.63691458	Down	0.578922	0.719201248
54392	5	3	9.05	5.82	-0.636898639	Down	0.578922	0.718557572
70110	5	3	25.05	16.11	-0.636854109	Down	0.578922	0.717060131
12608	20	12	89.5	57.56	-0.63682109	Down	0.232184	0.643357664
67414	5	3	7.37	4.74	-0.63677756	Down	0.578922	0.717166884
67486	5	3	10.96	7.05	-0.636552636	Down	0.578922	0.719738527
11820	5	3	10.26	6.6	-0.636492801	Down	0.578922	0.720599845
14183	5	3	6.84	4.4	-0.636492801	Down	0.578922	0.718343269
18821	15	9	42.92	27.61	-0.636459188	Down	0.304848	0.654785805
27878	10	6	31.23	20.09	-0.636455005	Down	0.409916	0.793050694
13406	5	3	12.17	7.83	-0.636244955	Down	0.578922	0.717380486
216805	5	3	10.6	6.82	-0.63622062	Down	0.578922	0.720276609
114565	5	3	6.17	3.97	-0.636131482	Down	0.578922	0.718450404
218914	10	6	10.49	6.75	-0.636055271	Down	0.409916	0.792129827
11684	5	3	11.22	7.22	-0.636001934	Down	0.578922	0.717915046
320951	5	3	15.68	10.09	-0.635999385	Down	0.578922	0.719631007
16170	10	6	13.38	8.61	-0.635992973	Down	0.409916	0.792682091

67580	5	3	12.99	8.36	-0.635826583	Down	0.578922	0.718022054
11845	5	3	8.95	5.76	-0.635818871	Down	0.578922	0.717487334
71063	5	3	5.92	3.81	-0.635806178	Down	0.578922	0.717808071
207278	5	3	7.66	4.93	-0.635756746	Down	0.578922	0.719523519
14672	5	3	10.27	6.61	-0.635714005	Down	0.578922	0.718236165
72106	413	248	1321.6	850.74	-0.635495399	Down	2.82E-08	2.86E-06
272636	5	3	7.44	4.79	-0.635276965	Down	0.578922	0.717701127
70686	5	3	8.06	5.19	-0.6350453	Down	0.578922	0.717273669
212517	5	3	5.06	3.26	-0.63426542	Down	0.578922	0.72006128
19216	585	352	1895.8	1222.85	-0.632559326	Down	4.61E-11	7.83E-09
16548	2	1	8.53	5.54	-0.622659765	Down	0.678286	0.802847483
26949	28	17	68.85	44.82	-0.619314006	Down	0.164615	0.567659103
231329	28	17	49.15	32	-0.619119511	Down	0.164615	0.56742395
16443	157.32	95.74	74.79	48.81	-0.615668632	Down	0.000769724	0.022874548
76743	10.24	6.24	47.13	30.79	-0.614183814	Down	0.409916	0.793419641
268465	59	36	178.46	116.73	-0.612425359	Down	0.0438562	0.332962993
17527	62.53	38.19	251.91	164.94	-0.610967081	Down	0.040615	0.314964972
19664	43.52	26.6	51.93	34.04	-0.609337188	Down	0.0804858	0.431244264
15273	18	11	12.23	8.02	-0.608750262	Down	0.276514	0.714778811
23831	31	19	129.37	84.99	-0.606138098	Down	0.1510114	0.530644366
574418	628	385	1643.05	1079.88	-0.60550538	Down	5.34E-11	8.72E-09
56424	101	62	409.39	269.37	-0.603886677	Down	0.00898104	0.135874971
70047	37	23	111.3	73.44	-0.599815629	Down	0.1271648	0.543192146
171567	44.03	27	167.3	110.52	-0.598129978	Down	0.0864076	0.455349993
72195	17.34	10.71	43.29	28.67	-0.594491896	Down	0.254172	0.670141068
70294	21	13	86.87	57.64	-0.591787702	Down	0.251092	0.680565646

232943	21	13	78.05	51.8	-0.591446534	Down		0.251092	0.680344035
217125	21	13	42.21	28.02	-0.591127874	Down		0.251092	0.6807874
403178	113	70	227.34	150.99	-0.590398542	Down		0.00670458	0.112025723
54524	45	28	163.86	109.29	-0.584302319	Down		0.092428	0.464428374
208146	2.62	1.63	2.9	1.94	-0.579996248	Down		0.678286	0.795828794
17118	386	241	616.54	412.73	-0.57899619	Down		7.81E-07	6.19E-05
230793	8	5	8.08	5.41	-0.578726699	Down		0.504726	0.814551017
98415	3.85	2.4	4.21	2.82	-0.578125071	Down		0.754082	0.85150174
72692	8	5	17.6	11.79	-0.578011711	Down		0.504726	0.816609964
22350	8	5	17.48	11.71	-0.577964109	Down		0.504726	0.814867102
654812	8	5	26.18	17.54	-0.57781635	Down		0.504726	0.815025237
71448	8	5	30.09	20.16	-0.577788468	Down		0.504726	0.814393067
232984	8	5	28.97	19.41	-0.577759567	Down		0.504726	0.815658389
50798	8	5	9.91	6.64	-0.577701816	Down		0.504726	0.815816831
16675	8	5	34.58	23.17	-0.577679825	Down		0.504726	0.816133899
67054	8	5	22.49	15.07	-0.577604244	Down		0.504726	0.815500009
97484	8	5	25.28	16.94	-0.577562589	Down		0.504726	0.816292526
13200	8	5	25.28	16.94	-0.577562589	Down		0.504726	0.815183433
58233	8	5	17.86	11.97	-0.577308928	Down		0.504726	0.81534169
224648	8	5	6.22	4.17	-0.576867197	Down		0.504726	0.816451214
54711	8	5	9.87	6.62	-0.576218868	Down		0.504726	0.814709029
102626	2	1	4.77	3.21	-0.571415969	Down		0.678286	0.793256192
67158	43	27	102.76	69.18	-0.570851877	Down		0.107237	0.516089692
210146	43	27	46.55	31.34	-0.570775988	Down		0.107237	0.516388355
211914	62	39	74.43	50.2	-0.568196872	Down		0.053204	0.344521777
170753	27	17	12.95	8.75	-0.565597176	Down		0.207848	0.6613779

74203	13	8	23.42	15.84	-0.56416874	Down	0.367388	0.737701628
70802	34.93	22.04	57.04	38.58	-0.564120838	Down	0.1837862	0.618392628
70511	97	61.23	305.68	206.84	-0.563506953	Down	0.01523214	0.187495025
12445	19	12	64.41	43.61	-0.562625701	Down	0.297986	0.644036755
12283	19	12	33.46	22.66	-0.562289584	Down	0.297986	0.644371493
17300	19	12	31.85	21.57	-0.562267196	Down	0.297986	0.644204081
67088	25.83	16.32	31.61	21.41	-0.56209624	Down	0.244232	0.672486589
56809	436.25	276.08	446.07	302.7	-0.559381449	Down	3.61E-07	3.13E-05
107065	30	19	35.27	23.95	-0.55841592	Down	0.1894592	0.61080589
19167	11.02	7	31.31	21.33	-0.553739543	Down	0.445424	0.824554639
414872	25.02	15.89	15.61	10.64	-0.552972386	Down	0.179435	0.611917473
66632	2.96	1.88	7.35	5.01	-0.552933647	Down	0.678286	0.789262733
109910	11	7	13.05	8.9	-0.552172566	Down	0.445424	0.824738118
110006	11	7	30.14	20.56	-0.551839152	Down	0.445424	0.824187926
245469	22	14	40.4	27.56	-0.551779405	Down	0.269472	0.702907997
170483	22	14	44.94	30.66	-0.551642428	Down	0.269472	0.703128414
14680	110	70	133.71	91.23	-0.551527145	Down	0.01188232	0.158704309
67467	140.39	89.33	171.93	117.31	-0.551495302	Down	0.0044036	0.087871356
22390	33	21	64.66	44.12	-0.551440717	Down	0.1728944	0.592282545
19054	147.92	94.82	226.14	154.62	-0.548489258	Down	0.00400102	0.081201189
17948	1.57	1	2.19	1.5	-0.545968369	Down	0.947628	0.96302059
544817	22	15	70.71	48.44	-0.545715394	Down	0.357514	0.719089677
59035	91	57	189.41	130.06	-0.542335172	Down	0.01773716	0.201626924
242202	3.77	2.42	3.74	2.57	-0.541269911	Down	0.754082	0.851039783
27359	9	6	20.42	14.05	-0.539412736	Down	0.544212	0.699797257
654821	56	36	170.01	117.16	-0.53713951	Down	0.0811958	0.434488908

67426	31	20	52.8		36.52	-0.531851164	Down		0.201744	0.646902437
20301	17	11	133.31		92.42	-0.528508011	Down		0.354546	0.713464877
319955	3.01	1.95	2.38		1.65	-0.528495549	Down		0.420186	0.803024278
67201	9.34	6.04	24.9		17.27	-0.527877659	Down		0.544212	0.699472977
11632	17	11	58.31		40.45	-0.52760362	Down		0.354546	0.713637462
15364	34	22	53.79		37.32	-0.527389003	Down		0.1837862	0.618142672
13714	17	11	31		21.51	-0.527260691	Down		0.354546	0.71381013
68955	17	11	15.14		10.51	-0.526602536	Down		0.354546	0.713982881
13871	74	48	139.72		97.17	-0.523955674	Down		0.0494148	0.364845209
51799	41	31	105.25		73.2	-0.52390468	Down		0.383252	0.763658978
11800	37	24	65.88		45.82	-0.523863096	Down		0.1676464	0.577155852
24001	20	13	52.74		36.75	-0.521153321	Down		0.318618	0.681547655
218850	41.47	26.98	37.8		26.37	-0.519488663	Down		0.12444	0.534020237
50877	668.93	435.93	1341.72		937.48	-0.517223813	Down		4.05E-09	4.88E-07
11736	36.26	23.69	30.05		21.05	-0.513544758	Down		0.157944	0.548748235
16151	55.37	36.21	54.77		38.41	-0.511903916	Down		0.0986252	0.490825532
320541	135.8	88.84	142.94		100.27	-0.511519666	Down		0.00854898	0.132223165
74315	4	3	7.17		5.03	-0.511414719	Down		0.804208	0.897146369
102414	29	19	78.22		54.94	-0.509680626	Down		0.235526	0.650884041
74383	5	3	8.45		5.94	-0.50848841	Down		0.578922	0.71930864
223776	32	21	91.29		64.23	-0.507209538	Down		0.213916	0.678093347
18715	38	25	124.86		88.06	-0.503752622	Down		0.1773354	0.605999123
19387	3.03	2	6.89		4.87	-0.500582211	Down		0.754082	0.852079892
171180	40.07	26.42	81.97		57.94	-0.500536313	Down		0.1531032	0.5366351
432779	75.99	50.11	185.91		131.42	-0.500419527	Down		0.0655292	0.404501835
11773	20.8	13.72	47.63		33.69	-0.49955012	Down		0.318618	0.681372495

68852	35.2	23.23	91.61	64.83	-0.498843516	Down	0.194624	0.626001664
104681	53	35	84.03	59.5	-0.498014816	Down	0.1132984	0.494885032
52683	142	94	293.16	208.06	-0.494688639	Down	0.0095599	0.142303986
67605	2	1	8.43	6	-0.49057013	Down	0.678286	0.799209545
58226	3	2	2.44	1.74	-0.487793842	Down	0.754082	0.856733523
64436	233	156	404.43	288.46	-0.48751874	Down	0.001267398	0.034464114
74081	3	2	1.5	1.07	-0.487351704	Down	0.754082	0.857201683
240396	3	2	4.92	3.51	-0.487187285	Down	0.754082	0.848967166
226250	3	2	5.13	3.66	-0.487115177	Down	0.754082	0.850578328
71474	3	2	5.76	4.11	-0.486930418	Down	0.754082	0.856850515
252973	3	2	4.05	2.89	-0.486852415	Down	0.754082	0.849426875
142682	6	4	6.18	4.41	-0.486828182	Down	0.630272	0.763724088
75482	3	2	28.84	20.59	-0.486127335	Down	0.754082	0.851848537
51789	3	2	4.65	3.32	-0.486047475	Down	0.754082	0.851732906
72632	3	2	35.14	25.09	-0.486001721	Down	0.754082	0.851155225
59090	3	2	5.56	3.97	-0.485945876	Down	0.754082	0.85603224
14629	3	2	5.84	4.17	-0.485920985	Down	0.754082	0.850117372
78294	6	4	57.68	41.19	-0.485777038	Down	0.630272	0.764391971
60345	3	2	12.14	8.67	-0.485664523	Down	0.754082	0.852890624
93696	3	2	24.42	17.44	-0.48566316	Down	0.754082	0.851617308
20587	3	2	12.35	8.82	-0.485660481	Down	0.754082	0.853586767
319187	2.99	2	50.14	35.81	-0.485599478	Down	0.93456	0.983119312
12580	3	2	9.8	7	-0.485426827	Down	0.754082	0.853238553
240131	3	2	11.55	8.25	-0.485426827	Down	0.754082	0.853122545
217664	3	2	7.7	5.5	-0.485426827	Down	0.754082	0.856382738
12617	3	2	6.3	4.5	-0.485426827	Down	0.754082	0.854982467

18392	3	2	6.65	4.75	-0.485426827	Down	0.754082	0.85242716
68528	6	4	42.39	30.28	-0.485358761	Down	0.630272	0.764503398
242646	3	2	21.04	15.03	-0.485289695	Down	0.754082	0.855798735
14559	3	2	14.39	10.28	-0.485226328	Down	0.754082	0.856967539
67125	3	2	13.2	9.43	-0.485208254	Down	0.754082	0.853006569
16477	3	2	11.03	7.88	-0.485165256	Down	0.754082	0.854865984
56426	3	2	10.54	7.53	-0.485153097	Down	0.754082	0.853354593
234959	6	4	10.26	7.33	-0.485145627	Down	0.630272	0.76350172
11886	3	2	8.93	6.38	-0.485103751	Down	0.754082	0.849771983
74253	3	2	8.86	6.33	-0.485101199	Down	0.754082	0.854633114
16848	3	2	8.79	6.28	-0.485098606	Down	0.754082	0.856149041
18747	3	2	8.79	6.28	-0.485098606	Down	0.754082	0.850924372
15422	3	2	8.3	5.93	-0.485079232	Down	0.754082	0.854400371
67397	6	4	32.57	23.27	-0.485072509	Down	0.630272	0.764280576
12715	3	2	14.43	10.31	-0.485026967	Down	0.754082	0.855565356
12488	9	6	11.07	7.91	-0.484905622	Down	0.544212	0.700013611
67980	3	2	10.86	7.76	-0.484895546	Down	0.754082	0.849082046
435653	3	2	15.94	11.39	-0.484883882	Down	0.754082	0.850693645
70377	3	2	15.31	10.94	-0.484861545	Down	0.754082	0.856616563
26992	12	8	34.09	24.36	-0.484834466	Down	0.477488	0.777073665
52040	3	2	4.73	3.38	-0.484816937	Down	0.754082	0.852311372
72469	6	4	13.28	9.49	-0.484775154	Down	0.630272	0.764614858
15260	9	6	13.21	9.44	-0.484771702	Down	0.544212	0.699581037
100045778	3	2	4.38	3.13	-0.484768213	Down	0.754082	0.849196958
243510	12	8	25.72	18.38	-0.484753876	Down	0.477488	0.777377744
57874	6	4	14.72	10.52	-0.484642967	Down	0.630272	0.763946586

98733	21	14	40.41	28.88	-0.484641609	Down	0.338448	0.686382113
16673	15	10	61.02	43.61	-0.484623189	Down	0.423096	0.793638822
224836	12	8	14.3	10.22	-0.484619951	Down	0.477488	0.776921715
63959	3	2	10.41	7.44	-0.484595542	Down	0.754082	0.849887081
230099	3	2	10.34	7.39	-0.484589916	Down	0.754082	0.852195616
56504	3	2	10.13	7.24	-0.484572572	Down	0.754082	0.853702901
269854	3	2	12.62	9.02	-0.484512572	Down	0.754082	0.851270699
110078	9	6	15.6	11.15	-0.484502319	Down	0.544212	0.69968913
68479	3	2	12.34	8.82	-0.484491834	Down	0.754082	0.854167754
19222	9	6	18.09	12.93	-0.484470133	Down	0.544212	0.69936495
72014	3	2	8.94	6.39	-0.4844589	Down	0.754082	0.855098981
101706	12	8	11.08	7.92	-0.484385546	Down	0.477488	0.777225674
22377	3	2	16.62	11.88	-0.484385546	Down	0.754082	0.848737498
54723	48	32	90.57	64.74	-0.484375892	Down	0.1422292	0.590858299
65079	3	2	10.73	7.67	-0.484351593	Down	0.754082	0.855682029
13030	21	14	29.49	21.08	-0.484350955	Down	0.338448	0.6860477
14828	3	2	7.96	5.69	-0.484339778	Down	0.754082	0.852658829
114255	3	2	7.75	5.54	-0.484310334	Down	0.754082	0.853935264
69956	3	2	7.75	5.54	-0.484310334	Down	0.754082	0.852542979
20393	3	2	7.75	5.54	-0.484310334	Down	0.754082	0.850347787
18762	51	34	79.08	56.53	-0.484296174	Down	0.1301084	0.553492841
54611	6	4	9.54	6.82	-0.484217527	Down	0.630272	0.763390584
435965	27	18	45.07	32.22	-0.484210956	Down	0.2751	0.711565776
16196	3	2	7.05	5.04	-0.484199524	Down	0.754082	0.848622711
112405	6	4	11.4	8.15	-0.48416186	Down	0.630272	0.76261354
74155	3	2	6.63	4.74	-0.484121811	Down	0.754082	0.851964199

11601	21	14	39.5	28.24	-0.484112565	Down	0.338448	0.686214865
76365	3	2	4.28	3.06	-0.484079144	Down	0.754082	0.856265874
67955	6	4	20.77	14.85	-0.484038285	Down	0.630272	0.763279481
67122	3	2	7.79	5.57	-0.483946001	Down	0.754082	0.857318803
18223	3	2	7.37	5.27	-0.483861657	Down	0.754082	0.84954188
18591	3	2	6.81	4.87	-0.483733026	Down	0.754082	0.854284047
52132	3	2	6.74	4.82	-0.483715445	Down	0.754082	0.855915472
102857	6	4	10.11	7.23	-0.483715445	Down	0.630272	0.763612888
19259	6	4	13.2	9.44	-0.483679165	Down	0.630272	0.762946365
11492	6	4	7.62	5.45	-0.483534768	Down	0.630272	0.76316841
19125	3	2	8.85	6.33	-0.483471956	Down	0.754082	0.848852316
57377	3	2	7.2	5.15	-0.483424474	Down	0.754082	0.853819067
78783	3	2	4.18	2.99	-0.483357458	Down	0.754082	0.854051493
71985	3	2	5.55	3.97	-0.483348764	Down	0.754082	0.850808993
208266	9	6	10.54	7.54	-0.483238438	Down	0.544212	0.699905418
246707	3	2	5.13	3.67	-0.483178763	Down	0.754082	0.856499634
385377	3	2	8.82	6.31	-0.483138651	Down	0.754082	0.857084595
243653	3	2	6.15	4.4	-0.483082887	Down	0.754082	0.853470664
231876	3	2	2.46	1.76	-0.483082887	Down	0.754082	0.849311901
66923	6	4	4.92	3.52	-0.483082887	Down	0.630272	0.762724449
14675	3	2	5.94	4.25	-0.48300009	Down	0.754082	0.857670356
71330	6	4	10.16	7.27	-0.482873133	Down	0.630272	0.763835321
22225	3	2	6.33	4.53	-0.482694449	Down	0.754082	0.849656916
72193	6	4	5.17	3.7	-0.48263901	Down	0.630272	0.764726351
13593	3	2	4.08	2.92	-0.482600783	Down	0.754082	0.85755314
66866	3	2	5.1	3.65	-0.482600783	Down	0.754082	0.852774711

229725	19	16	59.16	42.34	-0.482600783	Down	0.773228	0.867470701
75785	3	2	2.99	2.14	-0.482534688	Down	0.754082	0.857787604
99439	3	2	3.8	2.72	-0.482392767	Down	0.754082	0.851386204
217218	3	2	5.42	3.88	-0.482236199	Down	0.754082	0.857435955
76686	3	2	6.09	4.36	-0.482114093	Down	0.754082	0.855215527
13433	3	2	3.81	2.73	-0.480890047	Down	0.754082	0.854749533
216892	5	3	10.29	7.42	-0.47175189	Down	0.578922	0.718772003
54384	52	35	101.09	72.95	-0.470660407	Down	0.136574	0.57308737
67116	46	31	175.3	126.63	-0.469206761	Down	0.1634948	0.564029946
228852	185.45	125.03	194.06	140.28	-0.468193442	Down	0.00514726	0.097786188
67902	154.08	103.98	480.75	347.78	-0.467111888	Down	0.00866554	0.133529552
56444	373.12	251.9	620.49	449.16	-0.466178506	Down	5.71E-05	0.002699351
100040766	117	79	103.86	75.2	-0.465835564	Down	0.0260466	0.248547888
71131	37	25	68.11	49.34	-0.465108917	Down	0.21609	0.682126286
71957	31	21	88.94	64.59	-0.461521584	Down	0.262364	0.68955491
74096	59	40	149.52	108.68	-0.460252004	Down	0.1199106	0.515646565
11792	28	19	146.11	106.26	-0.459456304	Down	0.29003	0.627656601
22632	28	19	81.14	59.02	-0.459209382	Down	0.29003	0.627819883
215194	56	38	143.4	104.32	-0.459029249	Down	0.1309252	0.555830913
68184	5.89	4	17.75	12.93	-0.457096749	Down	0.841222	0.929342573
382019	6.06	4.12	7.15	5.21	-0.456659869	Down	0.630272	0.763057371
108767	25	17	87.06	63.46	-0.456162499	Down	0.32148	0.686610647
21877	22	15	110.45	80.71	-0.452574079	Down	0.357514	0.719263538
67561	66	45	115.59	84.5	-0.451993345	Down	0.1053632	0.508247645
26987	6.83	6.05	17.52	12.81	-0.451732299	Down	0.897816	0.970098291
69632	180	123	114.19	83.67	-0.448653974	Down	0.00757604	0.121464795

26936	18.7	12.79	14.28	10.47	-0.447734537	Down	0.37746	0.753200158
18025	19	13	50.23	36.84	-0.447276214	Down	0.399218	0.780336617
22184	19	13	45.37	33.28	-0.447083226	Down	0.399218	0.780519967
228880	3050.9	2088.48	7540.32	5534.68	-0.446125844	Down	5.76E-28	4.00E-25
14772	534.15	366.07	1105.18	812.05	-0.444640894	Down	4.94E-06	0.000321077
232087	35	24	83.83	61.63	-0.443833838	Down	0.249644	0.677082048
73242	2	1	6.76	4.98	-0.440877504	Down	0.678286	0.80182097
11938	16	11	24.57	18.11	-0.440111311	Down	0.448144	0.828852239
76366	240	166	322.54	237.77	-0.43991139	Down	0.00293442	0.061815972
319181	2.9	2	44.92	33.13	-0.439219724	Down	0.93456	0.977557984
233011	29	20	59.63	44.09	-0.43558686	Down	0.304624	0.654980957
28081	68	47	173.46	128.53	-0.432497879	Down	0.1145962	0.500028831
67899	39	27	224.55	166.6	-0.430648321	Down	0.237216	0.655119262
234683	13	9	28.5	21.15	-0.430304256	Down	0.506654	0.817187039
56354	26	18	75.74	56.21	-0.430228606	Down	0.337812	0.685259301
215090	13	9	31.74	23.56	-0.429962589	Down	0.506654	0.81734547
235461	39	27	32.13	23.85	-0.429931715	Down	0.237216	0.654901903
19651	26	18	36.13	26.82	-0.429888018	Down	0.337812	0.685426396
229699	8	6	26.31	19.56	-0.427704878	Down	0.706418	0.809544715
219022	60.32	41.89	168.83	125.69	-0.425701412	Down	0.1253514	0.536547839
269966	4.69	3.27	7.99	5.97	-0.420464572	Down	0.804208	0.900042336
74438	195	136	359.86	269.1	-0.419293358	Down	0.00903446	0.135208168
209225	20	14	29.34	21.95	-0.418647931	Down	0.419908	0.810309478
319259	30	21	207.09	155.33	-0.414921395	Down	0.31889	0.681428785
81014	994.93	696.46	2491.89	1870.09	-0.414132682	Down	5.45E-09	6.39E-07
245595	10	7	15.44	11.59	-0.413792186	Down	0.578686	0.720413855

16600	10	7	21.94	16.47	-0.413722971	Down	0.578686	0.720521653
246738	24	16	45.47	34.16	-0.412607029	Down	0.304656	0.654880541
211945	57	40	58.89	44.31	-0.410390347	Down	0.1693188	0.581469969
243371	141	99	350.5	263.84	-0.40975114	Down	0.029998	0.270730323
233046	101	71	146.01	110.06	-0.407776947	Down	0.0677472	0.41541964
64540	16	10	73.18	55.23	-0.405997288	Down	0.330376	0.696669715
76916	1.42	1	4.61	3.48	-0.405679445	Down	0.947628	0.964670001
106582	17	12	76.9	58.18	-0.402460302	Down	0.471594	0.771101135
77626	17	12	25.01	18.93	-0.401830646	Down	0.471594	0.771252688
70103	106	75	642.1	486.86	-0.399291024	Down	0.0669832	0.411037763
52398	14.67	10.02	14.44	10.95	-0.399139872	Down	0.533362	0.842624872
73656	20.83	14.74	76.39	57.93	-0.399073126	Down	0.419908	0.810121602
20532	24	17	70.07	53.21	-0.397099493	Down	0.393514	0.78204681
319996	199.59	141.5	351.03	266.82	-0.395727522	Down	0.01228122	0.157460758
29877	193	137	219.34	166.95	-0.39375284	Down	0.0142714	0.178306786
71799	145	103	301.99	229.99	-0.392929643	Down	0.0342762	0.295556746
21367	12.76	9.06	8.9	6.78	-0.392520063	Down	0.63652	0.769725755
66950	1552.03	1103.52	2468.27	1881.71	-0.391455913	Down	4.00E-12	8.31E-10
68511	188	134.04	891.85	681.5	-0.388087427	Down	0.01711294	0.196138807
270106	14	10	122.53	93.76	-0.386090544	Down	0.533362	0.843265286
66873	7	5	8.69	6.65	-0.386001836	Down	0.67177	0.800486635
68846	14	10	70.61	54.05	-0.385577899	Down	0.533362	0.842944958
71803	7	5	34.88	26.7	-0.385560298	Down	0.67177	0.801060214
215474	7	5	8.23	6.3	-0.385540602	Down	0.67177	0.801404756
54712	7	5	6.61	5.06	-0.385512887	Down	0.67177	0.800945432
15460	7	5	8.49	6.5	-0.385324836	Down	0.67177	0.800601285

21763	14	10	18.9	14.47	-0.385321313	Down	0.533362	0.842784885
242443	14	10	12.16	9.31	-0.385290156	Down	0.533362	0.843425542
110308	7	5	21.55	16.5	-0.385221845	Down	0.67177	0.800372017
11840	7	5	26.33	20.16	-0.385211883	Down	0.67177	0.800715968
68693	28	20	32.62	24.98	-0.384983305	Down	0.36906	0.74052285
216984	7	5	13.28	10.17	-0.384935467	Down	0.67177	0.801289875
12428	42	30	99.76	76.4	-0.384888827	Down	0.26816	0.700144136
68050	21	15	59.32	45.43	-0.384873292	Down	0.439614	0.822768352
19324	14	10	35.41	27.12	-0.384799665	Down	0.533362	0.843105092
27029	63	45	97.39	74.59	-0.384791417	Down	0.1731704	0.592983909
67154	7	5	12.73	9.75	-0.384758295	Down	0.67177	0.801519669
20852	7	5	12.31	9.43	-0.384501086	Down	0.67177	0.801634615
207352	23.14	16.53	33.76	25.87	-0.38403485	Down	0.375856	0.750179366
20017	8.53	6.12	14.2	10.93	-0.377597529	Down	0.706418	0.809656223
14417	56.08	40.27	66.5	51.2	-0.37721053	Down	0.1995656	0.640410859
73649	27.99	20.14	35.88	27.68	-0.374335947	Down	0.442756	0.821077039
109065	43	31	84.41	65.24	-0.371657142	Down	0.27826	0.607876466
13024	313.94	226.99	1602.88	1242.1	-0.367885094	Down	0.00326816	0.068156289
15547	83	62	157.92	122.41	-0.367472474	Down	0.1875078	0.622110209
382083	1521	1100	4003.14	3103.76	-0.367115067	Down	1.08E-10	1.73E-08
140780	76	55	76.92	59.69	-0.365869509	Down	0.1528954	0.536132585
15511	19.33	14	46.2	35.87	-0.365115106	Down	0.51436	0.819607346
105727	4	3	3.55	2.76	-0.363150758	Down	0.804208	0.898109619
11991	11	8	11.1	8.63	-0.363127212	Down	0.6092	0.751208239
72199	91	66	153.53	119.39	-0.362838585	Down	0.120362	0.517053279
270672	223	162	342.8	267.01	-0.360473335	Down	0.01528684	0.187890392

66836	11	8	102.48	79.84	-0.360158755	Down		0.6092	0.750652036
66163	11	8	57.18	44.57	-0.359437659	Down		0.6092	0.750763211
94044	11	8	10.57	8.24	-0.359259134	Down		0.6092	0.750540894
14723	22	16	54.1	42.18	-0.359069499	Down		0.458412	0.845775222
20677	44	32	99.45	77.54	-0.359030636	Down		0.28825	0.628215885
11605	11	8	24.34	18.98	-0.358849176	Down		0.6092	0.750874419
109880	11	8	7.52	5.87	-0.357372159	Down		0.6092	0.751096933
27047	1253.59	915.82	4847.04	3795.68	-0.352745619	Down		1.62E-08	1.73E-06
73902	11.26	8.23	17.72	13.88	-0.352371036	Down		0.6092	0.750985659
67068	1	1	9.96	7.81	-0.350823194	Down		0.947628	0.969413891
14088	45	33	96.93	76.13	-0.348478173	Down		0.298124	0.644167698
12014	61.87	45.32	48.49	38.09	-0.348274966	Down		0.235218	0.650464931
103742	15	11	137.06	107.67	-0.348191263	Down		0.557962	0.715818964
23825	15	11	92.81	72.94	-0.34757006	Down		0.557962	0.715929345
114872	22	16.13	26.98	21.21	-0.347145727	Down		0.458412	0.845962797
170731	15	11	22.41	17.62	-0.346928725	Down		0.557962	0.715488026
74142	15	11	33.89	26.65	-0.346724104	Down		0.557962	0.715708618
230101	15	11	28.28	22.24	-0.346625332	Down		0.557962	0.715598305
23794	30	22	24.64	19.38	-0.346433685	Down		0.398542	0.779381429
56527	158	116	214.34	168.73	-0.345184604	Down		0.0501906	0.365066418
27364	128	94	246.88	194.38	-0.344930181	Down		0.0785276	0.421838709
54631	26.52	19.48	31.25	24.62	-0.344025428	Down		0.426928	0.800285625
108909	19.06	14.02	38.79	30.59	-0.342624669	Down		0.51436	0.819764329
328424	20	15	75.27	59.36	-0.34258388	Down		0.53395	0.843393688
68612	4.08	3	30.22	23.84	-0.342119425	Down		0.804208	0.900163407
212547	38	28	89.02	70.32	-0.34019443	Down		0.349268	0.706088199

18701	61	45	428.67	338.84	-0.339263261	Down	0.235218	0.65024883
108960	6.56	4.84	14.35	11.36	-0.337087902	Down	0.630272	0.762835391
102060	402	297	1569.91	1243.26	-0.336553819	Down	0.0022653	0.053702454
100043597	23	17	14.01	11.11	-0.334598139	Down	0.476374	0.775867695
235907	1296.97	959.6	2190.94	1738.06	-0.334072089	Down	4.97E-08	4.81E-06
654795	77	57	423.12	335.68	-0.333980297	Down	0.18763	0.622267529
12834	27	20	48.46	38.49	-0.332310734	Down	0.442756	0.82089409
101023	19	14	55.89	44.4	-0.332030498	Down	0.51436	0.819450423
211401	8	6	10.83	8.63	-0.327600778	Down	0.706418	0.809321792
74778	82	61	281.86	224.77	-0.326529261	Down	0.1825272	0.619669046
225020	14	12	28.64	22.86	-0.325206089	Down	0.84093	0.929760634
18682	27.7	20.71	77.98	62.5	-0.319247965	Down	0.442756	0.820711222
54127	4	3	90.51	72.61	-0.317908944	Down	0.804208	0.899074939
26557	4	3	2.43	1.95	-0.31748219	Down	0.804208	0.898592019
52898	4	3	45.26	36.35	-0.31628122	Down	0.804208	0.897026108
54651	4	3	8.28	6.65	-0.316276427	Down	0.804208	0.895345835
20115	4	3	28.69	23.05	-0.315781217	Down	0.804208	0.895705363
19385	4	3	32.52	26.13	-0.315620133	Down	0.804208	0.896545387
240873	4	3	13.08	10.51	-0.315599872	Down	0.804208	0.898471371
20618	4	3	36.8	29.57	-0.315571523	Down	0.804208	0.899679318
27176	84	63	673.07	540.84	-0.315554699	Down	0.192496	0.61963606
78376	8	6	57.88	46.51	-0.315523982	Down	0.706418	0.809767761
17755	4	3	2.24	1.8	-0.315501826	Down	0.804208	0.897748158
434437	4	3	20.58	16.54	-0.315283748	Down	0.804208	0.894508056
68477	4	3	4.33	3.48	-0.315279719	Down	0.804208	0.899437469
216965	4	3	2.14	1.72	-0.315202232	Down	0.804208	0.89895416

56637	8	6	6.42	5.16	-0.315202232	Down		0.706418	0.809098992
18203	12	9	69.88	56.17	-0.315079805	Down		0.63652	0.769613909
67027	4	3	12.03	9.67	-0.315048848	Down		0.804208	0.899921297
74187	8	6	15.65	12.58	-0.315030735	Down		0.706418	0.808876315
66887	8	6	33.24	26.72	-0.315000374	Down		0.706418	0.810437637
235584	4	3	8.31	6.68	-0.315000374	Down		0.804208	0.895465645
106025	4	3	15.5	12.46	-0.314964147	Down		0.804208	0.895585488
629873	4	3	24.27	19.51	-0.314960331	Down		0.804208	0.899558377
66622	4	3	8.26	6.64	-0.31495854	Down		0.804208	0.89919575
78787	8	6	8.21	6.6	-0.314916198	Down		0.706418	0.809210377
22027	4	3	9.74	7.83	-0.314909465	Down		0.804208	0.894747261
110796	4	3	4.59	3.69	-0.314873337	Down		0.804208	0.898833414
244646	8	6	8.11	6.52	-0.31482995	Down		0.706418	0.808987638
67014	44	33	137.42	110.48	-0.314806763	Down		0.34861	0.704929237
216964	8	6	39.89	32.07	-0.31480277	Down		0.706418	0.809433239
11937	12	9	23.06	18.54	-0.314751269	Down		0.63652	0.769502095
15220	12	9	28.57	22.97	-0.31475018	Down		0.63652	0.769837634
19052	8	6	28.01	22.52	-0.314735156	Down		0.706418	0.81054939
66354	8	6	23.88	19.2	-0.314696526	Down		0.706418	0.810661175
192199	4	3	14.75	11.86	-0.314610945	Down		0.804208	0.894627643
218865	4	3	4.9	3.94	-0.31458612	Down		0.804208	0.894388501
65102	8	6	24.96	20.07	-0.314577317	Down		0.706418	0.80987933
29857	4	3	14.19	11.41	-0.314575798	Down		0.804208	0.895945209
22782	16	12	20.42	16.42	-0.314528739	Down		0.580742	0.718993332
107358	4	3	4.34	3.49	-0.314468006	Down		0.804208	0.897266662
67225	4	3	12.05	9.69	-0.314464576	Down		0.804208	0.899800292

230603	4	3	11.54	9.28	-0.314446514	Down	0.804208	0.898712701
231630	8	6	16.8	13.51	-0.314433558	Down	0.706418	0.81010256
67041	4	3	7.66	6.16	-0.314414041	Down	0.804208	0.898350754
18023	4	3	7.66	6.16	-0.314414041	Down	0.804208	0.89582527
75764	286.11	214.59	518.75	417.17	-0.314404018	Down	0.014647	0.181096117
20446	8	6	26.56	21.36	-0.3143435	Down	0.706418	0.810325914
67472	4	3	9.45	7.6	-0.314314911	Down	0.804208	0.897507345
56771	4	3	13.23	10.64	-0.314314911	Down	0.804208	0.895106309
320678	4	3	9.4	7.56	-0.314274522	Down	0.804208	0.895226056
208158	8	6	16.3	13.11	-0.314204279	Down	0.706418	0.80999093
17859	4	3	5.52	4.44	-0.31410859	Down	0.804208	0.894149488
26968	4	3	12.78	10.28	-0.314047572	Down	0.804208	0.894268979
14555	4	3	9.56	7.69	-0.31402702	Down	0.804208	0.896185184
244879	4	3	3.63	2.92	-0.314001179	Down	0.804208	0.897386988
229589	4	3	8.49	6.83	-0.313878975	Down	0.804208	0.89823017
17771	4	3	13.25	10.66	-0.313784922	Down	0.804208	0.894030029
67939	8	6	26.51	21.33	-0.313652705	Down	0.706418	0.808765022
20226	4	3	7.37	5.93	-0.313632515	Down	0.804208	0.900284511
229663	4	3	6.81	5.48	-0.313478905	Down	0.804208	0.89690588
22351	4	3	10.09	8.12	-0.313374542	Down	0.804208	0.897868612
210172	4	3	7.94	6.39	-0.313323076	Down	0.804208	0.896425287
226841	4	3	6.61	5.32	-0.313224026	Down	0.804208	0.899316593
328365	4	3	3.54	2.85	-0.312787441	Down	0.804208	0.896785683
230125	22.87	17.17	34.43	27.72	-0.312738923	Down	0.570106	0.730048019
207777	4	3	3.49	2.81	-0.312656906	Down	0.804208	0.896065181
68703	8	6	7.14	5.75	-0.312362118	Down	0.706418	0.810214222

18986	4	3	2.06	1.66	-0.311461096	Down	0.804208	0.897989099
68607	154	116	753.75	608.52	-0.308781417	Down	0.0824056	0.440396273
19089	4	3	13	10.51	-0.306748954	Down	0.804208	0.896305219
80718	3.2	2.41	3.09	2.5	-0.305678743	Down	0.754082	0.850463042
328110	107	81	174.7	141.79	-0.301123821	Down	0.1575518	0.547614256
74335	70	53	189.97	154.2	-0.300968841	Down	0.254738	0.67142062
72185	30	21	132.56	107.75	-0.298957638	Down	0.31889	0.681603825
217303	353.08	268.04	508.11	413.59	-0.296939554	Down	0.01098954	0.151397289
68585	25	19	44.66	36.39	-0.2954412	Down	0.51003	0.822154132
67228	39.88	30.32	90.67	73.91	-0.294855711	Down	0.433614	0.811904162
98758	17	13	46.24	37.7	-0.294576874	Down	0.601932	0.742796407
244954	15.75	12	41.96	34.26	-0.292489526	Down	0.704388	0.809112721
69707	214	163	355.43	290.27	-0.292169983	Down	0.0514002	0.362458529
76485	14.92	11.39	40.39	33.05	-0.289347874	Down	0.683726	0.791938202
23808	25.27	18.6	51.27	41.96	-0.28910022	Down	0.410526	0.792940308
52023	17	13	34.56	28.33	-0.286772616	Down	0.601932	0.742576156
386463	17	13	42.66	34.97	-0.28676617	Down	0.601932	0.742356036
12331	34	26	83.08	68.12	-0.286422781	Down	0.452762	0.836092455
107934	17	13	9.78	8.02	-0.286232228	Down	0.601932	0.742466079
14704	47	36	187.41	153.87	-0.284485959	Down	0.378802	0.755696821
16551	10.59	8.11	14.59	11.98	-0.284351975	Down	0.761922	0.856866193
56248	47	36	113.72	93.38	-0.28430051	Down	0.378802	0.755515686
229211	143.18	109.78	244.81	201.26	-0.282602022	Down	0.1130018	0.493848728
228003	63.39	48.62	167.97	138.13	-0.282176898	Down	0.294394	0.636935121
105352	48.88	37.5	107.23	88.19	-0.282021608	Down	0.38857	0.773514586
239570	17	13.06	30.21	24.88	-0.280039699	Down	0.601932	0.742686265

20042	13	10	172.66	142.2	-0.280012429	Down	0.661216	0.791993427
100465	4	3.07	9.32	7.68	-0.279223644	Down	0.804208	0.894866912
67896	26	20	47.72	39.35	-0.278230407	Down	0.525834	0.834534563
54215	13	10	35.59	29.35	-0.27811143	Down	0.661216	0.792107448
110157	35	27	76.58	63.33	-0.274078581	Down	0.465424	0.855109981
26367	5.22	4.78	10.82	8.96	-0.272129862	Down	0.841222	0.929959913
80290	22	17	36.2	29.99	-0.271508175	Down	0.570106	0.730272787
52504	106	82	163.14	135.31	-0.269842092	Down	0.205094	0.653864818
246196	449	347.6	1231.12	1021.77	-0.268900909	Down	0.00847284	0.131534518
23827	24.13	18.69	74.55	61.92	-0.267802881	Down	0.49356	0.801348831
234076	168.87	131.24	248.91	207.42	-0.263069183	Down	0.1251216	0.535839853
328572	9	7	6.85	5.71	-0.262613242	Down	0.736064	0.839702296
12556	9	7	20.31	16.93	-0.262608266	Down	0.736064	0.839472114
353502	18	14	98.91	82.45	-0.262596894	Down	0.62173	0.765411352
14428	9	7	31.07	25.9	-0.262570144	Down	0.736064	0.839587189
73010	9	7	13.11	10.93	-0.262374285	Down	0.736064	0.839817434
67819	9	7	19.19	16	-0.262282806	Down	0.736064	0.839242059
15437	9	7	29.6	24.68	-0.262254781	Down	0.736064	0.838782326
57376	9	7	20.4	17.01	-0.262186011	Down	0.736064	0.838323097
69934	9	7	30.03	25.04	-0.262169913	Down	0.736064	0.838437857
244885	18	14	43.82	36.54	-0.26211285	Down	0.62173	0.765298126
18263	36	28	95.05	79.26	-0.26209366	Down	0.477762	0.776759985
70785	9	7	23.84	19.88	-0.262066479	Down	0.736064	0.839127078
83669	36	28	57.74	48.15	-0.26203531	Down	0.477762	0.776911785
17135	9	7	21.39	17.84	-0.261820867	Down	0.736064	0.839357071
12908	9	7	13.69	11.42	-0.261559796	Down	0.736064	0.838897212

11491	9	7	13.51	11.27	-0.261540159	Down	0.736064	0.838552648
21833	95	74	256.55	214.24	-0.26001216	Down	0.247518	0.673071006
22608	22.99	17.92	70.75	59.09	-0.259816149	Down	0.570106	0.730160386
20671	1	1	2.32	1.94	-0.258068153	Down	0.947628	0.96243288
77106	41	32	61.85	51.76	-0.256935978	Down	0.456142	0.841960422
381560	10.27	8.02	16.2	13.56	-0.256636635	Down	0.761922	0.856287542
109342	23	18	68.54	57.5	-0.253384236	Down	0.586854	0.72602024
73827	203	159	577.64	485.03	-0.252096665	Down	0.0996384	0.495571504
78323	37	29	82.59	69.4	-0.251031448	Down	0.489786	0.795687096
73061	39.07	30.65	82.9	69.72	-0.249799532	Down	0.433614	0.812086899
15116	14	11	44.82	37.75	-0.247666004	Down	0.683726	0.791827981
100609	70	55	210.39	177.21	-0.247606116	Down	0.344854	0.697503679
74165	28	22	47.6	40.1	-0.247359337	Down	0.555626	0.713041941
22183	28	22	41.65	35.09	-0.247256548	Down	0.555626	0.713151928
217705	11.89	9.36	23.73	20.02	-0.245270126	Down	0.784818	0.879524657
54342	33	26	87.51	73.92	-0.24348313	Down	0.527346	0.836455598
433365	122.58	96.65	326.25	275.78	-0.242460068	Down	0.21444	0.679495522
20872	104	82	244.4	206.6	-0.242404031	Down	0.257542	0.678596258
13680	13.95	11	36.34	30.73	-0.241910653	Down	0.82394	0.913890261
218271	152.27	120.16	463.97	392.49	-0.241375628	Down	0.1733906	0.593493699
94184	23.52	18.58	44.06	37.37	-0.237588931	Down	0.586854	0.725912314
71910	4	3	17.3	14.71	-0.233974791	Down	0.804208	0.894986595
331046	87	69	211.19	179.58	-0.233914839	Down	0.317554	0.679795944
171286	40.38	32.04	88.81	75.55	-0.233290378	Down	0.524112	0.831960311
224530	469	372.92	1590.66	1355.84	-0.230438557	Down	0.0196204	0.217102857
68371	26.11	20.79	90.18	76.97	-0.228511263	Down	0.525834	0.834375422

16475	6.87	5.48	13.13	11.23	-0.225508988	Down	0.870422	0.953499403
57808	10	8	141.88	121.52	-0.22347746	Down	0.761922	0.856056301
20085	10	8	111.82	95.8	-0.223080689	Down	0.761922	0.85640321
22599	5	4	10.2	8.74	-0.222863967	Down	0.841222	0.928479674
18597	5	4	11.69	10.02	-0.222392421	Down	0.841222	0.927987308
74319	5	4	32.55	27.9	-0.222392421	Down	0.841222	0.92626813
77862	5	4	35.29	30.25	-0.222324288	Down	0.841222	0.928972563
225651	5	4	16.82	14.42	-0.222106541	Down	0.841222	0.926513337
94226	5	4	13.39	11.48	-0.222033319	Down	0.841222	0.927126922
225288	5	4	5.75	4.93	-0.221974309	Down	0.841222	0.927495463
12464	5	4	17.18	14.73	-0.221972606	Down	0.841222	0.926390718
75339	5	4	11.36	9.74	-0.221969157	Down	0.841222	0.927864298
16939	10	8	37.61	32.25	-0.221817146	Down	0.761922	0.857097872
107817	5	4	20.07	17.21	-0.221793519	Down	0.841222	0.929095867
67475	5	4	7.86	6.74	-0.221780721	Down	0.841222	0.926881391
380705	10	8	34.81	29.85	-0.221770883	Down	0.761922	0.855247938
66671	10	8	34.18	29.31	-0.221759429	Down	0.761922	0.855825184
106512	5	4	25.06	21.49	-0.221720932	Down	0.841222	0.928849292
66824	10	8	31.59	27.09	-0.221707544	Down	0.761922	0.855478743
18645	5	4	16.71	14.33	-0.221673124	Down	0.841222	0.926758674
14390	5	4	6.67	5.72	-0.221671614	Down	0.841222	0.92774132
16470	15	12	31.6	27.1	-0.221631707	Down	0.704388	0.80922443
214855	5	4	6.04	5.18	-0.221596452	Down	0.841222	0.92700414
12841	5	4	11.8	10.12	-0.22157757	Down	0.841222	0.927249737
216858	10	8	24.52	21.03	-0.221510129	Down	0.761922	0.856518909
216505	10	8	29.72	25.49	-0.221502743	Down	0.761922	0.856982017

69748	5	4	13	11.15	-0.221467913	Down	0.841222	0.9252886
72350	5	4	7.45	6.39	-0.221424494	Down	0.841222	0.929465976
67369	10	8	32.12	27.55	-0.221419574	Down	0.761922	0.8567504
18173	5	4	14.62	12.54	-0.221405963	Down	0.841222	0.929836379
68970	5	4	9.7	8.32	-0.221401219	Down	0.841222	0.927372584
69660	5	4	14.48	12.42	-0.221396429	Down	0.841222	0.929219204
52609	5	4	11.6	9.95	-0.221356375	Down	0.841222	0.929712878
66853	5	4	13.64	11.7	-0.221335115	Down	0.841222	0.925778106
237782	5	4	4.5	3.86	-0.221324154	Down	0.841222	0.928602847
321006	10	8	12.38	10.62	-0.221227548	Down	0.761922	0.856634639
319520	5	4	13.86	11.89	-0.221178542	Down	0.841222	0.928233426
332397	10	8	16.96	14.55	-0.221117017	Down	0.761922	0.855709672
13835	10	8	20.48	17.57	-0.221101524	Down	0.761922	0.855363325
17979	10	8	8.8	7.55	-0.221026879	Down	0.761922	0.856171906
319475	5	4	12.04	10.33	-0.220995138	Down	0.841222	0.928356533
56516	5	4	6.41	5.5	-0.220892738	Down	0.841222	0.929589411
27371	5	4	7.05	6.05	-0.220688115	Down	0.841222	0.925410928
18393	5	4	11.07	9.5	-0.220655804	Down	0.841222	0.925533289
21354	5	4	11.71	10.05	-0.220545574	Down	0.841222	0.928726053
446101	5	4	12.77	10.96	-0.220510727	Down	0.841222	0.926023054
241727	5	4	7.35	6.31	-0.220104245	Down	0.841222	0.927618376
22138	17	12	1.21	1.04	-0.218423519	Down	0.471594	0.7714043
234138	16.28	13.57	35.61	30.63	-0.217337069	Down	0.723472	0.826130165
12870	5	4	7.18	6.18	-0.216377006	Down	0.841222	0.92811035
15267	15.37	12.39	195.91	169.11	-0.212229067	Down	0.704388	0.809001042
18600	32.07	25.83	45.01	38.87	-0.211588457	Down	0.51435	0.819905431

15289	31	25	72.9	63.03	-0.209880152	Down	0.596364	0.736143724
240697	259.64	209.57	683.89	591.8	-0.208654598	Down	0.120078	0.51609971
216345	47	38	45.02	39.03	-0.205982594	Down	0.518372	0.823005803
544678	43.11	34.97	118.35	102.93	-0.201396174	Down	0.477736	0.777021356
94061	5	4.07	21.49	18.73	-0.198314583	Down	0.841222	0.926635989
16205	35.62	29	161.46	140.9	-0.196505185	Down	0.644614	0.779173895
19727	147.53	120.19	270.45	236.23	-0.195169706	Down	0.2809	0.613160782
56410	11.77	9.61	16.64	14.57	-0.191654556	Down	0.784818	0.879643127
215257	22	18	90.56	79.42	-0.189371597	Down	0.689876	0.798394742
71834	22	18	29.39	25.78	-0.189073095	Down	0.689876	0.7985058
17385	11	9	32.16	28.21	-0.189060741	Down	0.784818	0.879406218
237759	11	9	12.95	11.36	-0.188989263	Down	0.784818	0.879287812
22026	44	36	38.37	33.66	-0.188943588	Down	0.566242	0.725434901
103213	11	9	25.76	22.6	-0.188809821	Down	0.784818	0.87976163
117599	2.47	2.03	3.64	3.2	-0.185866545	Down	0.93456	0.987238004
70225	1	1	7.68	6.77	-0.181950477	Down	0.947628	0.959621831
64658	531	437	582.69	514.2	-0.180398942	Down	0.0529808	0.343343642
13052	10	10	21.03	18.56	-0.18025214	Down	0.87128	0.952433116
237336	17	14	39.63	34.99	-0.179650303	Down	0.741194	0.843934767
67713	4	4	19.6	17.33	-0.177582	Down	0.914182	0.980398044
67096	29.53	24.44	102.29	90.76	-0.172536598	Down	0.672932	0.801412219
20845	70	58	116.85	103.81	-0.170712309	Down	0.509642	0.821687867
74183	146	121	253.82	225.54	-0.170422431	Down	0.339174	0.687351888
13169	6	5	17.14	15.29	-0.164778703	Down	0.870422	0.954001773
27028	6	5	8.77	7.83	-0.163564535	Down	0.870422	0.953373893
57314	6	5	17.74	15.84	-0.163433674	Down	0.870422	0.953248416

106326	6	5	8.87	7.92	-0.163433674	Down	0.870422	0.952872183
66244	6	5	10.75	9.6	-0.163230349	Down	0.870422	0.952621526
14962	6	5	14.59	13.03	-0.163142799	Down	0.870422	0.954127449
80707	6	5	17.96	16.04	-0.163113208	Down	0.870422	0.953624946
52348	12	10	12.54	11.2	-0.163038616	Down	0.805346	0.894578036
71176	6	5	18.74	16.74	-0.162821425	Down	0.870422	0.953122972
29856	6	5	12.65	11.3	-0.162814612	Down	0.870422	0.953876131
71382	12	10	18.09	16.16	-0.16276521	Down	0.805346	0.895055972
66711	6	5	26.07	23.29	-0.162679943	Down	0.870422	0.952496247
22594	6	5	18.77	16.77	-0.162545961	Down	0.870422	0.952746838
214292	6	5	13.8	12.33	-0.162495467	Down	0.870422	0.953750522
68810	12	10	31.27	27.94	-0.162447199	Down	0.805346	0.89481694
66223	46.26	38.55	83.81	74.89	-0.162349303	Down	0.58593	0.724984912
280635	6	5	11.66	10.42	-0.162212511	Down	0.870422	0.952997561
217207	6	5	9.23	8.25	-0.161936529	Down	0.870422	0.954253157
100126824	123.51	103.18	730.17	653.68	-0.159647838	Down	0.422964	0.793570111
17083	73	61	333.64	298.82	-0.159015553	Down	0.530996	0.840163095
217030	29.46	24.63	27.59	24.74	-0.157299956	Down	0.672932	0.801641685
226982	49	41	41.84	37.54	-0.156454201	Down	0.613934	0.75626126
71163	65.66	55.03	82.23	73.89	-0.1542857	Down	0.600172	0.740734383
66634	3.57	3	7.05	6.34	-0.153140417	Down	0.923686	0.987155305
94089	25	21	124.66	112.23	-0.151540246	Down	0.73225	0.835809636
51902	75	63	89.44	80.56	-0.150856505	Down	0.54483	0.700051024
12867	19	16	304.67	274.62	-0.149810751	Down	0.773228	0.86735376
14169	44	37	104.63	94.33	-0.149507994	Down	0.648678	0.783858501
213054	41.49	34.94	32.09	28.97	-0.147564105	Down	0.618964	0.762118888

246154	77	65	161.89	146.52	-0.143916266	Down	0.558356	0.715882939
66844	187	158	614.12	556.23	-0.14283903	Down	0.362962	0.728990297
104859	26.02	22	22.23	20.16	-0.14101231	Down	0.7451	0.848034072
67812	10.08	8.53	16.47	14.94	-0.140660407	Down	0.761922	0.855594192
66865	26	22	55.8	50.62	-0.140557615	Down	0.7451	0.847918094
83429	13	11	28.98	26.29	-0.140543453	Down	0.82394	0.913524949
243897	13	11	32.93	29.88	-0.140222364	Down	0.82394	0.913646687
320184	13.58	11.5	10.52	9.55	-0.139562066	Down	0.82394	0.913768458
353211	528	447	280.47	254.63	-0.139444059	Down	0.1328206	0.5630159
208836	72	61	104.09	94.56	-0.138529534	Down	0.585546	0.724617529
77945	337	287	486.66	442.84	-0.136128659	Down	0.259478	0.682185288
211429	14.3	12.18	24.8	22.64	-0.131466162	Down	0.84093	0.92988419
21933	159.46	135.79	334.43	305.33	-0.131334922	Down	0.425124	0.797083552
244219	108	92	209.64	191.47	-0.130795649	Down	0.526682	0.835561663
14683	39	33	147.82	135.05	-0.130347839	Down	0.687306	0.795752501
235504	3.51	3	7.44	6.82	-0.125530882	Down	0.923686	0.983366326
12503	179	164	591.96	543.13	-0.12420214	Down	0.872604	0.953379449
21854	7	6	52.71	48.41	-0.122771598	Down	0.894494	0.970415199
117592	7	6	14.61	13.42	-0.122571507	Down	0.894494	0.971428423
11782	7	6	43.11	39.6	-0.122522132	Down	0.894494	0.969783006
66290	7	6	43.27	39.75	-0.12241226	Down	0.894494	0.971048216
12215	7	6	33.94	31.18	-0.122365637	Down	0.894494	0.970288694
16596	7	6	30.99	28.47	-0.122360262	Down	0.894494	0.96953036
59043	7	6	19.32	17.75	-0.12227607	Down	0.894494	0.970162223
16068	7	6	33.49	30.77	-0.122205932	Down	0.894494	0.969656667
19045	7	6	34.98	32.14	-0.12216036	Down	0.894494	0.971555224

107569	7	6	31.07	28.55	-0.122031496	Down		0.894494	0.970035784
54634	7	6	16.78	15.42	-0.121939951	Down		0.894494	0.969909379
228836	7	6	16.04	14.74	-0.121937617	Down		0.894494	0.970668306
21981	7	6	10.74	9.87	-0.121872004	Down		0.894494	0.971682059
18550	14	12	22.44	20.63	-0.121328855	Down		0.84093	0.930007779
214597	7	6	11.04	10.15	-0.121260445	Down		0.894494	0.970921546
68988	7	6	15.02	13.81	-0.121171493	Down		0.894494	0.971301654
270110	7	6	10.32	9.49	-0.120962978	Down		0.894494	0.971174918
76306	10.28	8.82	24.33	22.38	-0.120526284	Down		0.761922	0.855940727
13446	134	115	352.4	324.23	-0.120196339	Down		0.51619	0.82016746
18128	7	6	4.9	4.51	-0.119654316	Down		0.894494	0.97079491
18148	5	4	24.29	22.37	-0.118797234	Down		0.841222	0.925655681
231583	67	57	122.94	113.41	-0.116406534	Down		0.614838	0.757262729
22145	15	12	48.28	44.54	-0.116324118	Down		0.704388	0.809336171
64707	29	25	45.27	41.85	-0.113327684	Down		0.780532	0.875075016
226139	44	38	61.24	56.71	-0.110871127	Down		0.734514	0.838278836
328580	140	121	134.07	124.25	-0.109740599	Down		0.543028	0.698598638
170776	89	77	385	357.02	-0.108853551	Down		0.63364	0.766465829
235441	21.62	18.7	26.78	24.84	-0.108490787	Down		0.801562	0.897563908
14764	15	13	38.13	35.43	-0.105955066	Down		0.85657	0.940802398
16658	15	13	29.66	27.56	-0.10594271	Down		0.85657	0.940926597
71722	9	9	9.79	9.1	-0.105442315	Down		0.87736	0.95731872
77579	15	13	12.83	11.93	-0.104927127	Down		0.85657	0.941050828
140792	666.23	577.98	1344.04	1250.13	-0.104497947	Down		0.1961962	0.630327637
72585	12.59	10.93	13.63	12.68	-0.104230817	Down		0.805346	0.89493644
225392	53	46	201.17	187.16	-0.104143042	Down		0.726614	0.829490341

12633	61.2	53.19	63.32	59.01	-0.101701797	Down	0.711024	0.812361761
53617	23	20	89.4	83.33	-0.101438851	Down	0.826956	0.916258439
19720	45.02	39.18	71.59	66.81	-0.099694021	Down	0.743264	0.846175912
57321	28.27	24.63	52.6	49.14	-0.098164942	Down	0.7692	0.863184518
170765	12.37	10.8	54.06	50.57	-0.096279733	Down	0.805346	0.894697472
433904	6.89	6.01	19.38	18.14	-0.095394115	Down	0.897816	0.972371071
19025	1265	1096	2521.65	2364.04	-0.0931136	Down	0.0748296	0.406699609
100043688	2.67	2.33	4.8	4.5	-0.093109404	Down	0.93456	0.986862152
100043686	2.67	2.33	4.8	4.5	-0.093109404	Down	0.93456	0.9839901
100043684	2.67	2.33	4.8	4.5	-0.093109404	Down	0.93456	0.977312273
12577	8	7	28.25	26.49	-0.092803024	Down	0.914966	0.980228156
22190	8	7	22.21	20.83	-0.092546554	Down	0.914966	0.980101968
73712	24	21	81.99	76.9	-0.092464362	Down	0.83873	0.928315021
234686	8	7	13.55	12.71	-0.092328821	Down	0.914966	0.979975812
59013	8	7	24.04	22.55	-0.092309463	Down	0.914966	0.979345522
360198	2.28	2	32.45	30.44	-0.092250119	Down	0.93456	0.982374149
97884	16	14	29.1	27.3	-0.092118202	Down	0.871058	0.953066879
233744	16	14	17.3	16.23	-0.092109038	Down	0.871058	0.952691064
140488	32	28	48.99	45.96	-0.092108494	Down	0.812072	0.901928872
99712	16	14	38.18	35.82	-0.092051765	Down	0.871058	0.952816303
21387	8	7	18.13	17.01	-0.091995784	Down	0.914966	0.979093632
98732	16	14	15.22	14.28	-0.091972379	Down	0.871058	0.952941575
217109	8	7	20.58	19.31	-0.091894818	Down	0.914966	0.979219561
215445	8	7	16.39	15.38	-0.091760351	Down	0.914966	0.979849689
208869	8	7	5.87	5.51	-0.091308185	Down	0.914966	0.979597541

22035	104.73	91.71	141.44	132.8	-0.090935033	Down	0.660658	0.791552947
229228	25	22	158.84	149.36	-0.088780433	Down	0.849962	0.934161115
278304	559.33	492.25	914.56	862.99	-0.083733979	Down	0.350666	0.708742236
78581	379.24	334	872.65	823.98	-0.082793818	Down	0.452666	0.836100729
30049	4.55	4.01	8.79	8.3	-0.082751829	Down	0.914182	0.981030232
11744	17	15	47.42	44.85	-0.080387678	Down	0.884554	0.963904378
67168	17	15	46.34	43.84	-0.080010246	Down	0.884554	0.963778163
17827	26	23	116.27	110.02	-0.079713092	Down	0.860702	0.944468066
20702	6.81	6.02	32.54	30.81	-0.078815569	Down	0.897816	0.969972337
574428	43	38	103.1	97.68	-0.077909227	Down	0.814558	0.904448508
72043	7	6	11.99	11.36	-0.077868824	Down	0.894494	0.970541736
229603	10.96	9.69	9.07	8.6	-0.076765891	Down	0.94858	0.957323733
192976	26	23	85.72	81.29	-0.076553962	Down	0.860702	0.944343531
16478	104	92	241.24	228.79	-0.076445144	Down	0.715802	0.817708463
14362	26	23	39.54	37.5	-0.076422275	Down	0.860702	0.94421903
106389	35	31	155.37	147.49	-0.075090823	Down	0.840486	0.929640304
68916	157.13	139.48	433.87	412.9	-0.071470416	Down	0.658394	0.788953985
240442	9	8	14.37	13.69	-0.069937615	Down	0.932788	0.988881252
619301	9	8	50.12	47.75	-0.069885682	Down	0.932788	0.988377556
80880	9	8	22.97	21.89	-0.069478902	Down	0.932788	0.988503432
97761	9	8	12.55	11.96	-0.069469975	Down	0.932788	0.988251712
73072	18	16	25.82	24.61	-0.069244343	Down	0.897186	0.972068321
66493	7.87	7	35.27	33.63	-0.068692797	Down	0.890562	0.968295623
103806	9	8	10.77	10.27	-0.068582068	Down	0.932788	0.98875528
74244	311	277	551.51	526.68	-0.066460369	Down	0.580136	0.718349949
71151	28	25	52.54	50.29	-0.063144647	Down	0.88087	0.960896601

21916	635.23	567.35	1477.09	1414.4	-0.062567553	Down	0.453656	0.837557483
20850	10.87	9	18.59	17.81	-0.061839254	Down	0.94858	0.957556009
16582	2	2	4.56	4.37	-0.061400545	Down	0.93456	0.984737718
237073	2.23	2	2.69	2.58	-0.060235107	Down	0.93456	0.988241677
78784	9	7	21.24	20.38	-0.059629715	Down	0.736064	0.838667471
330660	26.81	24	51.93	49.83	-0.059553651	Down	0.976916	0.979978063
15015	638.19	571.6	2408.57	2312.41	-0.058779637	Down	0.47687	0.776523536
71807	4	3	8.84	8.49	-0.058281816	Down	0.804208	0.896665519
56284	62.87	56.46	83.68	80.57	-0.054640096	Down	0.866638	0.950229911
72789	6.78	6.09	7	6.75	-0.05246742	Down	0.897816	0.970728552
12298	20	18	17.26	16.65	-0.051910287	Down	0.92027	0.984516157
56335	10	9	33.17	32	-0.051807107	Down	0.94858	0.957788397
277773	10	9	40.93	39.49	-0.051671299	Down	0.94858	0.957904633
215001	10	9	26.52	25.59	-0.051500628	Down	0.94858	0.958369862
68597	286.42	281.21	528.52	510.08	-0.051234532	Down	0.533096	0.843165143
75705	10	9	17.28	16.68	-0.050983929	Down	0.94858	0.957672189
72121	10	9	16.51	15.94	-0.050688491	Down	0.94858	0.958020898
17193	66.25	59.69	123.77	119.58	-0.049685553	Down	0.817674	0.907787239
16847	41.39	37.32	49.56	47.92	-0.048548279	Down	0.890064	0.969400857
217166	92	83	222.11	214.83	-0.048078875	Down	0.830386	0.919936347
21940	3	3	16.2	15.68	-0.047068254	Down	0.923686	0.980856458
13709	102.33	85.78	161.66	156.55	-0.046339244	Down	0.446724	0.826409605
12986	28.64	26.01	53.52	52.12	-0.038241032	Down	0.992784	0.993978542
20353	11	10	19.12	18.62	-0.03822945	Down	0.962774	0.97082434
13831	11	10	91.45	89.07	-0.038043576	Down	0.962774	0.97011897
50905	11	10	36.02	35.1	-0.037327151	Down	0.962774	0.970706707

74026	11	10	16.87	16.44	-0.037249675	Down	0.962774	0.97023646
17886	11	10	9.85	9.6	-0.037089319	Down	0.962774	0.970471527
12611	22	20	31.15	30.36	-0.037060372	Down	0.940962	0.982895406
18105	11	10	19.35	18.86	-0.03700389	Down	0.962774	0.970001508
216543	33	30	45.82	44.66	-0.036994207	Down	0.925074	0.98120341
66643	43.94	39.96	94.33	91.98	-0.03639647	Down	0.904862	0.977203985
114304	13.47	12.26	19.71	19.23	-0.035569014	Down	0.987522	0.989543661
217430	12	10	42.06	41.07	-0.034363903	Down	0.805346	0.895175536
108654	47	43	32.34	31.72	-0.027926908	Down	0.932368	0.988561943
50789	53.91	49.35	84.5	82.94	-0.026883294	Down	0.969306	0.973988072
26558	103	94	283.47	278.38	-0.026140495	Down	0.882878	0.962960786
93671	131	120	198.57	195.03	-0.025951617	Down	0.890422	0.969663848
18640	12	11	21.78	21.4	-0.025393157	Down	0.97568	0.97944665
232791	12	11	27.56	27.08	-0.025348149	Down	0.97568	0.979092291
72057	12	11	48.85	48	-0.025324156	Down	0.97568	0.978856195
68310	12	11	20.96	20.6	-0.024994379	Down	0.97568	0.978974229
54139	12	11	19.38	19.05	-0.024777573	Down	0.97568	0.979210382
15502	12	11	24.25	23.84	-0.024600511	Down	0.97568	0.979328502
74498	159	146	305.78	301.04	-0.022538854	Down	0.89535	0.970331773
217201	13	12	26.98	26.7	-0.015050606	Down	0.987522	0.989782048
74255	13	12	26.62	26.35	-0.014707609	Down	0.987522	0.98966284
20933	13	12	25.12	24.87	-0.014429957	Down	0.987522	0.989424511
20908	64	59	140.92	139.74	-0.012131335	Down	0.952946	0.961613348
675812	49.57	45.88	105.26	104.45	-0.011144808	Down	0.945206	0.987080714
245368	65.18	60.38	93.48	92.85	-0.009755824	Down	0.958278	0.966524999
12984	1	1	1.51	1.5	-0.009586049	Down	0.947628	0.95973863

58799	109	101	179.36	178.2	-0.009360846	Down	0.966042	0.970825541
74157	69	64	71.91	71.52	-0.007845673	Down	0.978786	0.981735572
98828	88.9	82.49	371.47	369.47	-0.007788477	Down	0.999058	0.999058
268564	14	13	23.41	23.3	-0.00679498	Down	0.998478	0.998598009
78887	28	26	46.53	46.32	-0.006525934	Down	0.992784	0.993858959
66105	42	39	107.8	107.32	-0.006438218	Down	0.989072	0.990858188
230649	14	13	40.46	40.28	-0.006432637	Down	0.998478	0.998718048
21770	14	13	32.11	31.97	-0.006303921	Down	0.998478	0.998838115
18132	14	13	14.13	14.07	-0.006139137	Down	0.998478	0.999078335
27057	14	13	23.69	23.59	-0.00610278	Down	0.998478	0.999198489
57247	14	13	19.17	19.09	-0.006033234	Down	0.998478	0.998958211
231151	14	13	24.71	24.61	-0.005850353	Down	0.998478	0.999318672
67266	127	118	313.23	312.01	-0.005630127	Down	0.979864	0.98257994
235542	43	40	60.11	59.95	-0.003845266	Down	0.99528	0.996237806

Supplementary Table 2 Differentially expressed unknown genes in CDK5-knockdown versus wild-type mice skin

geneID	Wild-type-Expression	CDK5-knockdown-Expresion	Wild-type-FPKM	CDK5-knockdown-FPKM	log2 Ratio(CDK5-knockdown/Wild-type)	Up-Down-Regulation(CDK5-knockdown/Wild-type)	P-value	FDR
225884	0	4	0.001	43.92	15.42259043	Up	0.052321	0.355688759
633640	0	8	0.001	31.71	14.95265026	Up	0.00283656	0.060520553
100328588	0	9.77	0.001	30.41	14.8922582	Up	0.001368742	0.036739684
627302	0	7	0.001	27.98	14.77210834	Up	0.00587846	0.102331937
66206	0	5	0.001	25.53	14.63990592	Up	0.0252468	0.249499552
75328	0	7	0.001	18.82	14.19997901	Up	0.00587846	0.102546469
