# Supplementary material for: Gene expression profiles of skin from cyclin dependent kinases 5-knockdown mice
Source: Anim Biosci. 2023 Nov 2;37(4):567–75. doi: 10.5713/ab.23.0244 (PMC10915219; doi:10.5713/ab.23.0244)
Supplement: Supplementary file 2 [file ab-23-0244-Supplementary-Table-S2.pdf]

58799	109	101	179.36	178.2	-0.009360846	Down	0.966042	0.970825541
74157	69	64	71.91	71.52	-0.007845673	Down	0.978786	0.981735572
98828	88.9	82.49	371.47	369.47	-0.007788477	Down	0.999058	0.999058
268564	14	13	23.41	23.3	-0.00679498	Down	0.998478	0.998598009
78887	28	26	46.53	46.32	-0.006525934	Down	0.992784	0.993858959
66105	42	39	107.8	107.32	-0.006438218	Down	0.989072	0.990858188
230649	14	13	40.46	40.28	-0.006432637	Down	0.998478	0.998718048
21770	14	13	32.11	31.97	-0.006303921	Down	0.998478	0.998838115
18132	14	13	14.13	14.07	-0.006139137	Down	0.998478	0.999078335
27057	14	13	23.69	23.59	-0.00610278	Down	0.998478	0.999198489
57247	14	13	19.17	19.09	-0.006033234	Down	0.998478	0.998958211
231151	14	13	24.71	24.61	-0.005850353	Down	0.998478	0.999318672
67266	127	118	313.23	312.01	-0.005630127	Down	0.979864	0.98257994
235542	43	40	60.11	59.95	-0.003845266	Down	0.99528	0.996237806

Supplementary Table 2 Differentially expressed unknown genes in CDK5-knockdown versus wild-type mice skin

geneID	Wild-type-Expression	CDK5-knockdown-Expression	Wild-type-FPKM	CDK5-knockdown-FPKM	log2 Ratio(CDK5-knockdown/Wild-type)	Up-Down-Regulation(CDK5-knockdown/Wild-type)	P-value	FDR
225884	0	4	0.001	43.92	15.42259043	Up	0.052321	0.355688759
633640	0	8	0.001	31.71	14.95265026	Up	0.00283656	0.060520553
100328588	0	9.77	0.001	30.41	14.8922582	Up	0.001368742	0.036739684
627302	0	7	0.001	27.98	14.77210834	Up	0.00587846	0.102331937
66206	0	5	0.001	25.53	14.63990592	Up	0.0252468	0.249499552
75328	0	7	0.001	18.82	14.19997901	Up	0.00587846	0.102546469

69987	0	2.28	0.001	18.74	14.19383333	Up	0.224708	0.689198403
78896	0	2	0.001	16.69	14.02669633	Up	0.224708	0.653317704
68026	0	5	0.001	15.12	13.88417052	Up	0.0252468	0.254949785
77798	0	2	0.001	14.64	13.83762793	Up	0.224708	0.675016342
546347	0	1.39	0.001	13.93	13.76590764	Up	0.46568	0.785829098
66714	0	3	0.001	13.68	13.73978061	Up	0.1084294	0.506592385
100554	0	2	0.001	12.09	13.56152662	Up	0.224708	0.694574765
271278	0	4	0.001	10.85	13.40540742	Up	0.052321	0.345526223
100040299	0	1	0.001	10.36	13.33873638	Up	0.46568	0.828152015
230991	0	3	0.001	10	13.28771238	Up	0.1084294	0.503482722
74054	0	5.06	0.001	9.15	13.15955603	Up	0.0252468	0.251892833
68281	0	3	0.001	8.95	13.12767197	Up	0.1084294	0.502081824
74426	0	2	0.001	8.27	13.01367161	Up	0.224708	0.634043835
240755	0	2	0.001	8.2	13.00140819	Up	0.224708	0.638809453
97187	0	2	0.001	7.94	12.95492329	Up	0.224708	0.646987982
243780	0	2	0.001	7.42	12.85720347	Up	0.224708	0.660005389
100041433	0	3	0.001	7.31	12.83565569	Up	0.1084294	0.486908277
57355	0	2	0.001	7.13	12.79968636	Up	0.224708	0.696645033
70920	0	1	0.001	7.04	12.78135971	Up	0.46568	0.850136744
67750	0	1	0.001	6.9	12.75238065	Up	0.46568	0.768529012
77727	0	1	0.001	6.82	12.73555602	Up	0.46568	0.80845468
101835	0	3	0.001	6.32	12.62570884	Up	0.1084294	0.491416687
233103	0	5	0.001	6.16	12.58871464	Up	0.0252468	0.2434283
667803	0	1	0.001	5.67	12.46913302	Up	0.46568	0.781707339
80748	0	2	0.001	5.13	12.32474311	Up	0.224708	0.650137437
230757	0	2	0.001	5.05	12.30206767	Up	0.224708	0.683904634

212439	0	2	0.001	4.89	12.25561875	Up	0.224708	0.634905015
622019	0	1	0.001	4.31	12.07347215	Up	0.46568	0.76670425
72190	0	3	0.001	4.16	12.02236781	Up	0.1084294	0.49222097
75799	0	1	0.001	3.93	11.9403136	Up	0.46568	0.852943711
243219	0	3	0.001	3.68	11.84549005	Up	0.1084294	0.493027889
69053	0	2	0.001	3.52	11.78135971	Up	0.224708	0.683155012
66839	0	1	0.001	3	11.55074679	Up	0.46568	0.824627214
232217	0	1	0.001	2.75	11.4252159	Up	0.46568	0.777160706
434223	0	3	0.001	2.47	11.27029533	Up	0.1084294	0.517043574
328829	0	1	0.001	2	10.96578428	Up	0.46568	0.792093884
223593	0	1	0.001	1.78	10.79766153	Up	0.46568	0.769597474
214239	0	1	0.001	1.52	10.56985561	Up	0.46568	0.810483849
237029	1	15.88	1.84	31.36	4.091147888	Up	0.000160331	0.006293
224833	1	8	1.33	11.38	3.097002407	Up	0.01604696	0.18859711
100041840	33	253.93	142.38	1174.32	3.044007182	Up	7.44E-47	7.74E-44
100039863	0.25	1	2.45	16.29	2.73313295	Up	0.46568	0.804593704
100039939	0.25	1	2.45	16.29	2.73313295	Up	0.46568	0.776537731
67326	4	18	22.02	106.18	2.269625673	Up	0.001495712	0.038294829
319266	1.02	4.43	1.88	8.77	2.221844181	Up	0.1876922	0.61031137
215928	2	9	5.68	25.5	2.166534412	Up	0.0286094	0.262179314
668661	1	4	3.37	14.47	2.102244425	Up	0.1876922	0.607699142
75304	2	7	4.05	15.18	1.906177978	Up	0.0868802	0.453532085
626802	16.52	54.42	27.28	96.35	1.820441022	Up	8.97E-07	6.98E-05
100317	3	9	5.15	16.86	1.710960199	Up	0.0703334	0.427497605
101148	1	3	1.61	5.19	1.68867385	Up	0.332862	0.70120119
68283	1	3	3.1	9.98	1.6867716	Up	0.332862	0.698724698

74359	1	3	2.6	8.37	1.686716	Up		0.332862	0.701556409
320095	1	3	4.27	13.74	1.686074029	Up		0.332862	0.69679112
109299	1	3	2.91	9.36	1.685489377	Up		0.332862	0.695740945
117171	1	3	6.14	19.74	1.684811429	Up		0.332862	0.685071655
75480	1	3	9.2	29.57	1.684428477	Up		0.332862	0.684902251
225995	1	3	4.16	13.37	1.684344032	Up		0.332862	0.69784447
223739	1	3	1.33	4.27	1.682809824	Up		0.332862	0.69209013
207819	19.83	52.96	28.84	82.58	1.517721254	Up		2.13E-05	0.001145109
100233208	9.96	26.13	14.02	39.42	1.491441427	Up		0.001996508	0.048575857
78703	25	62	195.82	520.31	1.409843314	Up		1.52E-05	0.000857781
76964	7	17	26.88	69.97	1.380203354	Up		0.0281488	0.265261795
225594	53	124	125.4	314.54	1.326706148	Up		4.77E-09	5.67E-07
434234	2	5	7.42	18.02	1.280107919	Up		0.245598	0.670699363
628308	85.48	192.23	122.43	295.21	1.269784476	Up		1.49E-12	3.35E-10
319772	7	15	23.56	54.12	1.1998223	Up		0.0647598	0.400346431
545652	14.69	31.44	64.08	147.02	1.198066374	Up		0.00565008	0.101106055
100043915	3.59	7.44	5.28	11.74	1.152822574	Up		0.1846234	0.618209783
627914	3.59	7.44	5.28	11.74	1.152822574	Up		0.1846234	0.617961107
100043381	3.59	7.44	5.28	11.74	1.152822574	Up		0.1846234	0.616968398
668039	3.59	7.44	5.28	11.74	1.152822574	Up		0.1846234	0.614992519
68045	75	153	337.43	737.81	1.128661097	Up		1.01E-08	1.14E-06
69315	1	2	3.74	8.02	1.100563967	Up		0.573542	0.725846841
78625	2	4	5.48	11.75	1.100412958	Up		0.397842	0.785955195
70691	1	2	6.74	14.45	1.100248996	Up		0.573542	0.722550035
69770	1	2	10.78	23.1	1.099535674	Up		0.573542	0.729954571
381272	2	4	21.67	46.42	1.099047369	Up		0.397842	0.782610705

68544	2	4	14.78	31.66	1.099014986	Up	0.397842	0.785395796
408059	1	2	4.03	8.63	1.098580721	Up	0.573542	0.731745321
667962	1.93	1.91	3.6	7.65	1.087462841	Up	0.947628	0.98874139
100303732	1.93	1.91	3.6	7.65	1.087462841	Up	0.947628	0.960557021
76421	4	7	8.67	16.26	0.907223359	Up	0.323714	0.688554242
668208	14.67	25.3	34.55	63.9	0.887130221	Up	0.049088	0.363076665
100101807	38.73	66.06	67.09	122.69	0.870848016	Up	0.001909832	0.0470169
319493	67	113.96	151.39	276.06	0.86671195	Up	9.43E-05	0.004085585
665211	3	5	22.88	40.85	0.836249026	Up	0.4415	0.822045536
100038657	3	5	26.26	46.88	0.836105654	Up	0.4415	0.825926596
243308	20	31	42.48	70.6	0.732884417	Up	0.0749048	0.406842585
102631730	24.19	37	231.57	379.34	0.712043143	Up	0.0535778	0.346134219
100041574	48.22	72.9	153.41	248.63	0.696607857	Up	0.01012224	0.149074618
74098	2	3	8.75	14.08	0.686292412	Up	0.623204	0.759250437
72244	2	3	3.99	6.42	0.686184551	Up	0.623204	0.759695354
233913	2	3	5.16	8.3	0.685740271	Up	0.623204	0.760698325
73667	2	3	7.7	12.38	0.685080964	Up	0.623204	0.763723194
67388	2	3	12.89	20.72	0.684771739	Up	0.623204	0.762824431
319482	2	3	1.68	2.7	0.684498174	Up	0.623204	0.766772214
100041261	2	3	20.19	32.44	0.684132909	Up	0.623204	0.764398656
215467	2	3	13.49	21.65	0.682476677	Up	0.623204	0.764285996
269700	2	3	0.86	1.38	0.682259702	Up	0.623204	0.764624076
100504263	28.52	42.09	49.77	78.75	0.662003536	Up	0.0500422	0.364624471
102639598	28.52	42.09	49.77	78.75	0.662003536	Up	0.0500422	0.364305465
100043133	52	75	97.24	150.37	0.628894968	Up	0.01500216	0.185212127
231807	5	7	13.15	19.74	0.58605919	Up	0.496454	0.805576001

67593	20.56	27.24	67.94	96.51	0.506417218	Up	0.210074	0.668205563
77877	24.19	31.7	40.73	57.22	0.490427683	Up	0.231074	0.640708682
240613	7	9	5.81	8.01	0.463264079	Up	0.529262	0.838535625
100529082	76.75	95.99	389.15	521.62	0.422672829	Up	0.0563802	0.354335079
330173	19.24	24.01	140.43	187.72	0.418731195	Up	0.32412	0.6871344
58248	21	26	75.15	99.73	0.408254446	Up	0.334926	0.687958343
67392	22	27	56.08	73.78	0.39574344	Up	0.339754	0.688192073
228602	13.04	14.96	12.88	16.1	0.321928095	Up	0.707696	0.808670477
240327	7	8.01	17.77	21.8	0.294884454	Up	0.696088	0.803349272
623781	14	16	30.62	37.52	0.293185545	Up	0.5793	0.717421536
100190996	3.24	3.64	5.38	6.47	0.266159539	Up	0.923686	0.982612018
74356	58.42	64.06	123.41	145.1	0.233588218	Up	0.352886	0.710812008
73344	11	12	36.2	42.33	0.225690791	Up	0.706648	0.808805778
381994	23	24	155.85	174.26	0.161083293	Up	0.698442	0.805507399
100042856	0.75	0.76	0.56	0.61	0.123382416	Up	0.96507	0.971021459
100043665	0.98	0.98	3.83	4.13	0.108797389	Up	0.96507	0.971138889
620078	1	1	3.58	3.85	0.104898858	Up	0.947628	0.966206664
319277	182.41	182.88	207.49	223.06	0.10439002	Up	0.504144	0.815826959
76982	3	3	6.31	6.77	0.101515829	Up	0.923686	0.986521782
78809	3	3	5.24	5.62	0.101003319	Up	0.923686	0.980481083
66523	2	2	9.85	10.56	0.100414205	Up	0.93456	0.979158116
103712	2	2	8.34	8.94	0.100227448	Up	0.93456	0.986361461
381306	9	9	20.62	22.1	0.100002037	Up	0.87736	0.958072514
68067	2	2	11.72	12.56	0.099863894	Up	0.93456	0.989247393
69066	4	4	24.94	26.72	0.099458543	Up	0.914182	0.982423921
73737	1	1	10.39	11.13	0.099257938	Up	0.947628	0.983806935

68920	1	1	8.86	9.49	0.099101388	Up	0.947628	0.960206112
73363	3	3	21.38	22.9	0.099085745	Up	0.923686	0.979981028
73103	1	1	6.67	7.14	0.098237313	Up	0.947628	0.981602463
240038	45.14	44.8	61.77	65.73	0.089645654	Up	0.820372	0.910661074
319887	25.36	24.84	82.22	86.33	0.070372617	Up	0.914714	0.980084367
639910	23	22.24	109.64	113.65	0.051823451	Up	0.927438	0.983460125
228356	761	723	1091.73	1118.42	0.034845963	Up	0.718556	0.820741864
68115	0	5	0.001	27.57	14.75081165	Up	0.0252468	0.244277468
625424	5	7	14.9	22.36	0.585607857	Up	0.496454	0.804634541
100038993	113.52	125	284.64	336.01	0.239365754	Up	0.1879076	0.606273416
114585	43.27	43.64	137.26	148.41	0.112677046	Up	0.743792	0.846661181
545651	13.78	0	64.09	0.001	-15.96781165	Down	0.000197405	0.007640048
67101	6	0	52.03	0.001	-15.66705609	Down	0.01986984	0.208758761
73410	3	0	41.16	0.001	-15.32895536	Down	0.1434012	0.582922025
546049	9.11	0	38.1	0.001	-15.21750338	Down	0.0027532	0.061750343
225058	2	0	29.51	0.001	-14.8489163	Down	0.277122	0.635768448
73453	4	0	25.32	0.001	-14.62798978	Down	0.074205	0.428493966
74892	2	0	24.06	0.001	-14.55434902	Down	0.277122	0.693096532
69349	3	0	22.39	0.001	-14.45056691	Down	0.1434012	0.54312307
628416	6	0	20.07	0.001	-14.292753	Down	0.01986984	0.212515345
246735	2	0	19.65	0.001	-14.26224169	Down	0.277122	0.63524302
100502831	2	0	17.34	0.001	-14.08181628	Down	0.277122	0.631070652
545655	1.23	0	15.94	0.001	-13.96036401	Down	0.53554	0.701768243
100233175	2.54	0	14.94	0.001	-13.86689253	Down	0.277122	0.657522715
666926	3	0	14.87	0.001	-13.86011703	Down	0.1434012	0.551405446
100041062	1.13	0	14.59	0.001	-13.83269226	Down	0.53554	0.842865205

66323	2	0	14.45	0.001	-13.81878187	Down	0.277122	0.666069371
75368	2	0	14.05	0.001	-13.77828251	Down	0.277122	0.609390106
621080	4	0	13.69	0.001	-13.74083483	Down	0.074205	0.419469976
72275	1	0	13.64	0.001	-13.73555602	Down	0.53554	0.773113869
330097	1	0	13.07	0.001	-13.67397152	Down	0.53554	0.755677182
545649	2.8	0	13.03	0.001	-13.66954946	Down	0.277122	0.675434142
433215	1	0	12.44	0.001	-13.60269887	Down	0.53554	0.746062002
77358	1	0	12.37	0.001	-13.59455788	Down	0.53554	0.797749434
69662	2	0	10.91	0.001	-13.41336348	Down	0.277122	0.67071907
626832	6	0	10.61	0.001	-13.37313704	Down	0.01986984	0.216409606
75758	2	0	10.25	0.001	-13.32333629	Down	0.277122	0.68690264
69068	2	0	10.24	0.001	-13.32192809	Down	0.277122	0.665108786
58251	3	0	10.16	0.001	-13.31061278	Down	0.1434012	0.568480889
240216	3	0	10.05	0.001	-13.29490788	Down	0.1434012	0.542629097
545653	1.39	0	9.87	0.001	-13.26883437	Down	0.53554	0.722709753
545648	1.14	0	9.78	0.001	-13.25561875	Down	0.53554	0.822182351
100041488	0.53	0	9.67	0.001	-13.23930017	Down	0.96507	0.971961688
381196	3	0	8.94	0.001	-13.12605912	Down	0.1434012	0.559419309
71952	3	0	8.61	0.001	-13.07179752	Down	0.1434012	0.566322442
622554	2	0	8.41	0.001	-13.03789009	Down	0.277122	0.61639459
67290	1	0	8.08	0.001	-12.98013958	Down	0.53554	0.786069561
239673	3	0	7.96	0.001	-12.95855272	Down	0.1434012	0.545357123
67851	1	0	7.7	0.001	-12.91064273	Down	0.53554	0.80904654
100126226	2	0	7.63	0.001	-12.89746734	Down	0.277122	0.676821885
670550	1	0	7.5	0.001	-12.87267488	Down	0.53554	0.723178893
381260	4	0	7.41	0.001	-12.85525783	Down	0.074205	0.416078036

72056	2	0	7.18	0.001	-12.80976813	Down	0.277122	0.672479487
70153	3	0	7.14	0.001	-12.80170836	Down	0.1434012	0.537980787
226777	3	0	7.05	0.001	-12.78340754	Down	0.1434012	0.537738344
97159	1	0	6.92	0.001	-12.75655632	Down	0.53554	0.703540944
237880	1	0	6.62	0.001	-12.6926155	Down	0.53554	0.740729445
75555	1	0	6.31	0.001	-12.62342429	Down	0.53554	0.766201572
217830	3	0	6.23	0.001	-12.60501645	Down	0.1434012	0.574779087
69861	1	0	5.75	0.001	-12.48934624	Down	0.53554	0.795612987
280621	1	0	5.58	0.001	-12.44604941	Down	0.53554	0.803648032
100039060	3.72	0	5.47	0.001	-12.41732512	Down	0.1434012	0.553194893
100039123	1	0	5.15	0.001	-12.33035672	Down	0.53554	0.76070815
70984	2	0	4.87	0.001	-12.24970606	Down	0.277122	0.689779289
320333	1	0	4.78	0.001	-12.2227949	Down	0.53554	0.77244381
73598	1	0	4.72	0.001	-12.20457114	Down	0.53554	0.781794446
73212	1	0	4.59	0.001	-12.16427844	Down	0.53554	0.726480003
667693	2	0	4.31	0.001	-12.07347215	Down	0.277122	0.642678975
226499	2	0	4.25	0.001	-12.05324713	Down	0.277122	0.674643699
69773	2	0	4.23	0.001	-12.04644195	Down	0.277122	0.626101592
70617	1	0	4.23	0.001	-12.04644195	Down	0.53554	0.693684362
100043456	1	0	4.12	0.001	-12.00842862	Down	0.53554	0.79689348
328830	1	0	3.95	0.001	-11.94763694	Down	0.53554	0.714941174
216393	2	0	3.91	0.001	-11.93295289	Down	0.277122	0.659780304
114671	1	0	3.82	0.001	-11.89935692	Down	0.53554	0.690673952
100041085	1	0	3.8	0.001	-11.8917837	Down	0.53554	0.701436855
11615	1	0	3.22	0.001	-11.65284497	Down	0.53554	0.841432844
100861908	0.86	0	3.14	0.001	-11.61654884	Down	0.96507	0.971608889

66808	1	0	2.97	0.001	-11.53624722	Down	0.53554	0.798607229
665186	1	0	2.88	0.001	-11.4918531	Down	0.53554	0.840480637
236366	1	0	2.72	0.001	-11.40939094	Down	0.53554	0.809340418
106064	2	0	2.62	0.001	-11.3553511	Down	0.277122	0.677617444
67430	1	0	2.57	0.001	-11.32755264	Down	0.53554	0.692283415
271221	1	0	2.54	0.001	-11.31061278	Down	0.53554	0.823702096
231201	1	0	2.33	0.001	-11.18611424	Down	0.53554	0.7542702
381622	1	0	2.28	0.001	-11.15481811	Down	0.53554	0.70834976
74200	1	0	2.24	0.001	-11.12928302	Down	0.53554	0.727072661
213438	1	0	2.24	0.001	-11.12928302	Down	0.53554	0.692390979
67313	1	0	2.23	0.001	-11.12282799	Down	0.53554	0.738642191
214489	1	0	2.2	0.001	-11.10328781	Down	0.53554	0.818708128
242594	1	0	2.19	0.001	-11.09671515	Down	0.53554	0.809928815
68861	1	0	1.69	0.001	-10.72280753	Down	0.53554	0.73088869
442834	2	0	1.6	0.001	-10.64385619	Down	0.277122	0.636294747
208111	1	0	1.33	0.001	-10.37721053	Down	0.53554	0.728022928
212728	1	0	1.32	0.001	-10.36632221	Down	0.53554	0.746937368
210108	1	0	1.3	0.001	-10.34429591	Down	0.53554	0.71967512
233865	1	0	1.08	0.001	-10.0768156	Down	0.53554	0.822030684
320492	0.99	0	1.07	0.001	-10.06339508	Down	0.96507	0.970904059
72503	1	0	0.88	0.001	-9.781359714	Down	0.53554	0.719326609
69155	9.01	1	22.6	2.69	-3.070644695	Down	0.01603832	0.192021382
76482	9	1	28.44	3.39	-3.068564286	Down	0.01603832	0.193132939
67884	7	1	27.96	4.28	-2.707681659	Down	0.0499732	0.368640955
630836	6.76	1.07	17.31	2.93	-2.562633155	Down	0.0869852	0.449847016
432582	6	1	10.03	1.79	-2.486290113	Down	0.0869852	0.452377406

241944	8.6	1.74	8.65	1.88	-2.201967471	Down		0.0284266	0.265474454
73545	4.89	1.03	10.58	2.4	-2.140233317	Down		0.253238	0.684598245
98404	9	2	17.34	4.13	-2.069890212	Down		0.0512964	0.364195686
414069	9.1	2.04	25.03	6.02	-2.055822899	Down		0.0512964	0.365756079
432479	59	14	593.79	150.92	-1.976168792	Down		3.03E-07	2.65E-05
667250	12.22	3	86.03	22.64	-1.92596588	Down		0.0303596	0.268747055
70591	2.64	0.73	3.88	1.16	-1.741931847	Down		0.277122	0.706257936
69399	32	9	21.74	6.56	-1.72858422	Down		0.000626732	0.019904721
667214	6.55	2	11.34	3.71	-1.611929548	Down		0.216528	0.678106695
67862	13	4	71.44	23.56	-1.600392541	Down		0.044249	0.335333269
100503879	19	6	130.48	44.15	-1.563343344	Down		0.01538816	0.188578615
74387	3	1	8.29	2.96	-1.485774926	Down		0.420186	0.807102425
70730	3	1	18.14	6.48	-1.485108738	Down		0.420186	0.792108678
100503353	6	2	11.89	4.25	-1.484213969	Down		0.216528	0.682473291
434179	3	1	6.35	2.27	-1.484064294	Down		0.420186	0.808782722
76261	3	1	10.85	3.88	-1.483566485	Down		0.420186	0.795714089
217370	3	1	8.75	3.13	-1.48312036	Down		0.420186	0.802471358
75939	65.32	22.58	220.06	81.57	-1.431786375	Down		1.31E-05	0.000763758
381218	23	8	72.07	26.87	-1.423402524	Down		0.01235926	0.158217542
105734734	17	6	177.82	67.22	-1.40345515	Down		0.0348934	0.297792801
270156	24	9	48.23	19.39	-1.314618007	Down		0.01599712	0.194608239
100141474	39	15	186.21	76.76	-1.278503935	Down		0.00244186	0.057075048
381287	20.31	7.96	78.13	32.83	-1.250861864	Down		0.0206816	0.216195469
66761	7	3	46.02	21.13	-1.122968216	Down		0.274784	0.715418543
67317	7	3	12.58	5.78	-1.121990524	Down		0.274784	0.714077971
68364	9	4	12.93	6.16	-1.069720019	Down		0.226072	0.631468651

73133	161	74	601.12	296.16	-1.021276221	Down	2.01E-07	1.80E-05
101056408	65	30	259.5	128.38	-1.015314072	Down	0.00106952	0.030167715
432870	2	1	6.26	3.35	-0.902001562	Down	0.678286	0.803190239
381353	2	1	2.84	1.52	-0.901819606	Down	0.678286	0.794484488
217887	2	1	7.06	3.78	-0.901281949	Down	0.678286	0.789152378
97820	2	1	6.16	3.3	-0.900464326	Down	0.678286	0.796840012
76925	2	1	18.74	10.04	-0.900361684	Down	0.678286	0.796952528
73449	4	2	10.75	5.76	-0.900195943	Down	0.512406	0.816963082
74666	4	2	11.27	6.04	-0.899867061	Down	0.512406	0.824546572
381933	4	2	23.58	12.64	-0.899567255	Down	0.512406	0.81994814
71837	8	4	26.34	14.12	-0.899515257	Down	0.325592	0.687801734
229588	2	1	9.25	4.96	-0.899113245	Down	0.678286	0.793479236
268759	2	1	6.75	3.62	-0.898897805	Down	0.678286	0.800797078
72139	2	1	7.62	4.09	-0.897690155	Down	0.678286	0.792365268
70900	376	192	802.51	439.35	-0.869148676	Down	3.48E-12	7.42E-10
66132	9	5	19.36	11.53	-0.74768644	Down	0.370266	0.742048022
67609	16	9	81.94	49.4	-0.730056851	Down	0.230942	0.64098345
330503	615.44	367.76	1397.73	895.45	-0.642400919	Down	6.73E-12	1.30E-09
381438	9085.89	5465	51114.5	32948.4	-0.6335242	Down	8.71E-149	7.25E-145
108899	11	7	14.64	9.99	-0.55135897	Down	0.445424	0.824371242
110956	31.96	20.54	64.4	44.38	-0.537151021	Down	0.201744	0.646653245
67038	3.07	2	11.02	7.68	-0.520946008	Down	0.754082	0.850002211
100041621	33.78	22.1	110.61	77.58	-0.511725141	Down	0.225964	0.631591012
73261	6	4	37.44	26.75	-0.485041543	Down	0.630272	0.764169213
73430	15	10	19.41	13.87	-0.48483233	Down	0.423096	0.793459954
78412	681	461	1341.97	973.99	-0.462373555	Down	8.80E-08	8.22E-06

234678	83	59	193.95	147.81	-0.391940898	Down	0.1111176	0.486124895
72061	9	7	8.85	7.38	-0.262056639	Down	0.736064	0.839012129
27660	126	98	724.21	606.37	-0.25620972	Down	0.1787614	0.609870279
104457	7	5	60.86	51.56	-0.23924207	Down	0.67177	0.801175028
67912	30	24	80.59	69.12	-0.221497616	Down	0.583266	0.721903374
320827	5	4	4.87	4.18	-0.22041883	Down	0.841222	0.926145576
633057	142.01	117.31	593.01	525.07	-0.175546664	Down	0.323254	0.689868308
75462	29	24	121.25	107.56	-0.172843087	Down	0.672932	0.801526936
545929	0.02	0.02	0.09	0.08	-0.169925001	Down	0.96507	0.970317481
112415	27.31	22.74	39.29	35.2	-0.158586739	Down	0.646622	0.781487533
66330	55	46	407.68	365.33	-0.158236954	Down	0.590888	0.730467843
72650	18.32	15.57	73.26	66.76	-0.134041746	Down	0.757732	0.852615006
240328	103.39	88.04	210.18	191.88	-0.131421049	Down	0.54971	0.706212276
213393	8	7	41.52	38.93	-0.092924099	Down	0.914966	0.979471515
319278	8	7	32.21	30.21	-0.092482477	Down	0.914966	0.979723599
100041586	4.5	4	39.75	37.85	-0.07066156	Down	0.914182	0.982550817
100041639	4.5	4	39.75	37.85	-0.07066156	Down	0.914182	0.980524416
382523	1	0	12.58	0.001	-13.6188443	Down	0.53554	0.738397405
545650	2.21	0	10.27	0.001	-13.32614856	Down	0.277122	0.689366865
225289	2	0	2.48	0.001	-11.27612441	Down	0.277122	0.630208298
210998	1	0	1.19	0.001	-10.21674586	Down	0.53554	0.771908599
67246	1	0	1.1	0.001	-10.10328781	Down	0.53554	0.826911921
227695	96	43	353.26	169.61	-1.058509169	Down	3.72E-05	0.001862942
100861753	121	63	470.04	262.33	-0.841400731	Down	0.000129691	0.005238651
620913	82.08	52.51	103.61	71.07	-0.543850648	Down	0.0287854	0.261488333
227622	11	10	75.93	73.97	-0.037729732	Down	0.962774	0.970353979
