# Supplementary material for: Gene expression profiles of skin from cyclin dependent kinases 5-knockdown mice
Source: Anim Biosci. 2023 Nov 2;37(4):567–75. doi: 10.5713/ab.23.0244 (PMC10915219; doi:10.5713/ab.23.0244)
Supplement: Supplementary file 3 [file ab-23-0244-Supplementary-Table-S3.pdf]

Supplementary Table 3 Statistic of Differentially Expressed Genes in CDK5-knockdown versus wild-type mice skin

| geneID | Wide_type-Expression | CDK5_knockdown-Expression | Wide_type-FPKM | CDK5_knockdown-FPKM | log2 Ratio(CDK5_knockdown/Wide_type) | Up-Down-Regulation(CDK5_knockdown/Wide_type) | P-value | FDR |
|---|---|---|---|---|---|---|---|---|
| 94245 | 0 | 2 | 0.001 | 11.15 | 13.44475609 | Up | 0.224708 | 0.665170853 |
| 15201 | 0 | 2 | 0.001 | 4.73 | 12.20762447 | Up | 0.224708 | 0.649009118 |
| 19014 | 0 | 4 | 0.001 | 4.48 | 12.12928302 | Up | 0.052321 | 0.351099227 |
| 100169 | 0 | 2 | 0.001 | 3.18 | 11.63481105 | Up | 0.224708 | 0.679678396 |
| 13798 | 0 | 1 | 0.001 | 2.82 | 11.46147945 | Up | 0.46568 | 0.79599903 |
| 18975 | 2 | 11 | 2.91 | 17.14 | 2.558276051 | Up | 0.00895402 | 0.135960585 |
| 12807 | 1 | 5 | 1.85 | 9.93 | 2.424268447 | Up | 0.1036324 | 0.508146848 |
| 240888 | 2 | 10 | 1.96 | 10.48 | 2.418713157 | Up | 0.0160922 | 0.18753949 |
| 14451 | 3 | 7 | 6.39 | 15.98 | 1.322379572 | Up | 0.1846234 | 0.617216276 |
| 50518 | 1 | 2 | 10.11 | 21.66 | 1.099250246 | Up | 0.573542 | 0.728061477 |
| 16413 | 17 | 26 | 61.33 | 100.55 | 0.713248226 | Up | 0.1115342 | 0.487691055 |
| 13626 | 2 | 3 | 6.55 | 10.54 | 0.686308055 | Up | 0.623204 | 0.763161219 |
| 67427 | 2 | 3 | 3.89 | 6.25 | 0.684086035 | Up | 0.623204 | 0.756591842 |
| 11776 | 4 | 5 | 5.65 | 7.57 | 0.422042433 | Up | 0.669584 | 0.800173555 |
| 13607 | 5 | 6 | 6.79 | 8.74 | 0.364221705 | Up | 0.681698 | 0.791683051 |
| 13518 | 66 | 77 | 49.98 | 62.53 | 0.323197616 | Up | 0.1813946 | 0.616830595 |
| 83603 | 1 | 1 | 2.18 | 2.34 | 0.102180395 | Up | 0.947628 | 0.968580345 |
| 20529 | 6 | 6 | 10.67 | 11.44 | 0.100526876 | Up | 0.897816 | 0.971359633 |
| 23937 | 8 | 8 | 19.89 | 21.32 | 0.100164162 | Up | 0.883768 | 0.963805181 |
| 22178 | 0 | 5 | 13.17 | 0.001 | -13.68496773 | Down | 0.0252468 | 0.252195225 |
| 232946 | 3.46 | 0 | 12.65 | 0.001 | -13.62684976 | Down | 0.1434012 | 0.577840865 |

| | | | | | | | | |
|---|---|---|---|---|---|---|---|---|
| 19659 | 5 | 0 | 12.79 | 0.001 | -13.64272864 | Down | 0.0383984 | 0.301997246 |
| 19668 | 4 | 0 | 10.96 | 0.001 | -13.41996018 | Down | 0.074205 | 0.430285578 |
| 20665 | 4 | 0 | 9.91 | 0.001 | -13.27466934 | Down | 0.074205 | 0.415238605 |
| 110312 | 1 | 0 | 9.32 | 0.001 | -13.18611424 | Down | 0.53554 | 0.747062588 |
| 18505 | 0 | 4 | 5.81 | 0.001 | -13.05494342 | Down | 0.052321 | 0.353379092 |
| 79456 | 4 | 0 | 6.82 | 0.001 | -12.73555602 | Down | 0.074205 | 0.444855767 |
| 14712 | 3 | 0 | 6.75 | 0.001 | -12.72067179 | Down | 0.1434012 | 0.580935436 |
| 17237 | 3 | 0 | 6.11 | 0.001 | -12.57695666 | Down | 0.1434012 | 0.589255005 |
| 26395 | 2 | 0 | 5.64 | 0.001 | -12.46147945 | Down | 0.277122 | 0.670914216 |
| 16678 | 2 | 0 | 5.47 | 0.001 | -12.41732512 | Down | 0.277122 | 0.616065232 |
| 72433 | 1 | 0 | 4.3 | 0.001 | -12.07012094 | Down | 0.53554 | 0.69748448 |
| 103199 | 2 | 0 | 4.09 | 0.001 | -11.99788513 | Down | 0.277122 | 0.65959158 |
| 12686 | 1 | 0 | 3.6 | 0.001 | -11.81378119 | Down | 0.53554 | 0.810370675 |
| 14682 | 3 | 0 | 3.55 | 0.001 | -11.79360331 | Down | 0.1434012 | 0.578401059 |
| 116905 | 1 | 0 | 3.21 | 0.001 | -11.64835758 | Down | 0.53554 | 0.801768323 |
| 13190 | 0 | 1 | 3.17 | 0.001 | -11.63026713 | Down | 0.46568 | 0.842924359 |
| 14634 | 4 | 0 | 3.16 | 0.001 | -11.62570884 | Down | 0.074205 | 0.414124618 |
| 23908 | 2 | 0 | 2.78 | 0.001 | -11.44086917 | Down | 0.277122 | 0.628490641 |
| 13649 | 2 | 0 | 2.51 | 0.001 | -11.29347165 | Down | 0.277122 | 0.632628851 |
| 213827 | 1 | 0 | 1.93 | 0.001 | -10.91438513 | Down | 0.53554 | 0.775131038 |
| 21384 | 1 | 0 | 1.87 | 0.001 | -10.86882255 | Down | 0.53554 | 0.708575026 |
| 13608 | 1 | 0 | 1.81 | 0.001 | -10.82177398 | Down | 0.53554 | 0.723296273 |
| 56453 | 1 | 0 | 1.58 | 0.001 | -10.62570884 | Down | 0.53554 | 0.774188384 |
| 19645 | 1 | 0 | 1.44 | 0.001 | -10.4918531 | Down | 0.53554 | 0.757604274 |
| 21859 | 1 | 0 | 1.43 | 0.001 | -10.48179943 | Down | 0.53554 | 0.733052861 |
| 11789 | 0 | 2 | 0.001 | 1.14 | -10.15481811 | Down | 0.224708 | 0.690470926 |

| | | | | | | | | |
|---|---|---|---|---|---|---|---|---|
| 18607 | 1 | 0 | 0.94 | 0.001 | -9.876516947 | Down | 0.53554 | 0.765149097 |
| 18129 | 1 | 0 | 0.63 | 0.001 | -9.299208018 | Down | 0.53554 | 0.758248824 |
| 17101 | 0 | 1 | 0.6 | 0.001 | -9.22881869 | Down | 0.46568 | 0.801929487 |
| 20431 | 2 | 12 | 40.73 | 6.33 | -2.68581441 | Down | 0.00493554 | 0.095286841 |
| 17918 | 9 | 2 | 5.12 | 1.22 | -2.069262662 | Down | 0.0512964 | 0.365442932 |
| 11774 | 4 | 1 | 6.66 | 1.78 | -1.903644936 | Down | 0.253238 | 0.668101902 |
| 93762 | 4 | 1 | 5.74 | 1.54 | -1.898120386 | Down | 0.253238 | 0.679520606 |
| 74355 | 8 | 2 | 7.56 | 2.03 | -1.896906507 | Down | 0.0841744 | 0.445840345 |
| 67299 | 3 | 1 | 2.84 | 1.01 | -1.491535637 | Down | 0.420186 | 0.801919199 |
| 22642 | 3 | 1 | 5.89 | 2.1 | -1.487878306 | Down | 0.420186 | 0.796257733 |
| 54635 | 3 | 1 | 5.72 | 2.04 | -1.487445995 | Down | 0.420186 | 0.803393315 |
| 17869 | 6 | 2 | 16.86 | 6.02 | -1.485769144 | Down | 0.216528 | 0.680668488 |
| 109052 | 3 | 1 | 6.15 | 2.2 | -1.483082887 | Down | 0.420186 | 0.804317393 |
| 16667 | 3 | 1 | 12.88 | 4.61 | -1.482293938 | Down | 0.420186 | 0.794990383 |
| 230857 | 2 | 1 | 2.75 | 1.47 | -0.903615464 | Down | 0.678286 | 0.794037395 |
| 171211 | 4 | 2 | 3.29 | 1.76 | -0.902512155 | Down | 0.512406 | 0.824865608 |
| 14462 | 2 | 1 | 4.12 | 2.21 | -0.898597968 | Down | 0.678286 | 0.793702406 |
| 11990 | 2 | 1 | 1.52 | 0.82 | -0.890375509 | Down | 0.678286 | 0.798531099 |
| 56187 | 4.98 | 2.63 | 13.16 | 7.45 | -0.820847158 | Down | 0.512406 | 0.824706059 |
| 18742 | 13 | 7 | 64.2 | 37.05 | -0.793099755 | Down | 0.24932 | 0.676423776 |
| 22173 | 825 | 455.81 | 933.45 | 553 | -0.755293266 | Down | 4.71E-20 | 1.78E-17 |
| 26570 | 7162 | 4127 | 5164.43 | 3191.15 | -0.694532703 | Down | 1.62E-138 | 6.72E-135 |
| 14183 | 5 | 3 | 6.84 | 4.4 | -0.636492801 | Down | 0.578922 | 0.718343269 |
| 14672 | 5 | 3 | 10.27 | 6.61 | -0.635714005 | Down | 0.578922 | 0.718236165 |
| 13871 | 74 | 48 | 139.72 | 97.17 | -0.523955674 | Down | 0.0494148 | 0.364845209 |
| 18591 | 3 | 2 | 6.81 | 4.87 | -0.483733026 | Down | 0.754082 | 0.854284047 |

| | | | | | | | | |
|---|---|---|---|---|---|---|---|---|
| 11491 | 9 | 7 | 13.51 | 11.27 | -0.261540159 | Down | 0.736064 | 0.838552648 |
| 20085 | 10 | 8 | 111.82 | 95.8 | -0.223080689 | Down | 0.761922 | 0.85640321 |
| 107817 | 5 | 4 | 20.07 | 17.21 | -0.221793519 | Down | 0.841222 | 0.929095867 |
| 18508 | 32 | 33 | 68 | 61.5 | -0.144948336 | Down | 0.683368 | 0.791633736 |
| 17199 | 1 | 1.02 | 2 | 1.82 | -0.13606155 | Down | 0.947628 | 0.97264248 |
| 18128 | 7 | 6 | 4.9 | 4.51 | -0.119654316 | Down | 0.894494 | 0.97079491 |
| 171531 | 2 | 2 | 3.2 | 2.98 | -0.102759574 | Down | 0.93456 | 0.97694394 |

Supplementary Table 4. Differentially expressed known hair color genes in CDK5-knockdown vs wild-type mice skin

| Symbol | Gene Name | Differential expression log2 Ratio(CDK5-knockdown/Wild-type) | Function |
|---|---|---|---|
| **(a) Melanocyte Development** | | | |
| Adam17 | a disintegrin and metallopeptidase domain 17 | Down-regulated | Protease, processing various surface proteins |
| Apc | adenomatosis polyposis coli | Down-regulated | Wnt pathway mediator; transcription factor |
| Arcn1 | archain 1 | Down-regulated | coatomer protein delta-COP, conserved across diverse eukaryotes |
| Dph1 | diphthamide biosynthesis 1 | Down-regulated | Delayed embryonic eye pigmentation |
| Dock7 | dedicator of cytokinesis 7 | Down-regulated | generalized hypopigmentation and localized white-spotting in mice, with a lack of pigment on the belly, tail tip, and paws; but |
