# Supplementary material for: Gene expression profiles of skin from cyclin dependent kinases 5-knockdown mice
Source: Anim Biosci. 2023 Nov 2;37(4):567–75. doi: 10.5713/ab.23.0244 (PMC10915219; doi:10.5713/ab.23.0244)
Supplement: Supplementary file 4 [file ab-23-0244-Supplementary-Table-S4.pdf]

11491	9	7	13.51	11.27	-0.261540159	Down	0.736064	0.838552648
20085	10	8	111.82	95.8	-0.223080689	Down	0.761922	0.85640321
107817	5	4	20.07	17.21	-0.221793519	Down	0.841222	0.929095867
18508	32	33	68	61.5	-0.144948336	Down	0.683368	0.791633736
17199	1	1.02	2	1.82	-0.13606155	Down	0.947628	0.97264248
18128	7	6	4.9	4.51	-0.119654316	Down	0.894494	0.97079491
171531	2	2	3.2	2.98	-0.102759574	Down	0.93456	0.97694394

Supplementary Table 4. Differentially expressed known hair color genes in CDK5-knockdown vs wild-type mice skin

Symbol	Gene Name	Differential expression log2 Ratio(CDK5-knockdown/Wild-type)	Function
**(a) Melanocyte Development**			
Adam17	a disintegrin and metallopeptidase domain 17	Down-regulated	Protease, processing various surface proteins
Apc	adenomatosis polyposis coli	Down-regulated	Wnt pathway mediator; transcription factor
Arcn1	archain 1	Down-regulated	coatomer protein delta-COP, conserved across diverse eukaryotes
Dph1	diphthamide biosynthesis 1	Down-regulated	Delayed embryonic eye pigmentation
Dock7	dedicator of cytokinesis 7	Down-regulated	generalized hypopigmentation and localized white-spotting in mice, with a lack of pigment on the belly, tail tip, and paws; but

			melanocytes in vitro hyperpigmented
Ece1	endothelin converting enzyme 1	Down-regulated	No melanocytes in uvea, dorsal skin at birth (perinatal lethal)
Eed	embryonic ectoderm development	Up-regulated	Diluted coat (dwarfism etc)
Egfr	epidermal growth factor receptor	Down-regulated	epidermal growth factor receptor
En1	engrailed 1	Up-regulated	transcription factor; Hyperpigmentation of digits
Fgfr2	fibroblast growth factor receptor 2	Down-regulated	Lighter skin (many other defects)
Gas1	growth arrest specific 1	Up-regulated	Can enhance hedgehog signaling, inhibit growth; RPE transdifferentiates to neural retina
Gata3	GATA binding protein 3	Down-regulated	transcription factor; Extra stem-like cells in hair follicles; abnormal hair, irregular pigment deposition
Gli3	GLI-Kruppel family member GLI3	Down-regulated	Signaling in Hedgehog pathway. Modifies SOX10 expression
Gnaq	guanine nucleotide binding protein, alpha q polypeptide	Down-regulated	GPCR signalling: limits melanocyte proliferation / Dark skin
Gna11	guanine nucleotide binding protein, alpha 11	Down-regulated	GPCR signalling: limits melanocyte proliferation / Dark skin
Gnpat	glyceronephosphate O-acyltransferase	Down-regulated	Abnormal RPE morphology, microphthalmia
Gpr161	G protein-coupled receptor 161	Up-regulated	Signal transduction;Vacuolated lens, occasional belly spot, spine development
Hells	helicase, lymphoid specific	Up-regulated	Early ageing includes graying by 15d old; p16 overexpression
Itgb1	integrin beta 1 (fibronectin receptor beta)	Up-regulated	transient patchy hypopigmentation, crest migration defect
Jmjd6	jumonji domain containing 6	Down-regulated	Transcriptional regulator; Lack of one/both eyes, ectopic RPE in nose
Krt1	keratin 1	Down-regulated	cytoskeleton; dark skin, Primary action in keratinocytes. Limits melanization

Krt17	keratin 17	Down-regulated	cytoskeleton;dark skin, abnormal hairs with clustered melanin granules
Krt75	keratin 75	Down-regulated	cytoskeleton;hair defects with variable pigment clumping
Lef1	lymphoid enhancer binding factor 1	Down-regulated	transcription factor, Wnt/b-catenin mediator,<br>Mutations result in impaired binding to beta-catenin
Mab21l2	mab-21-like 2 (C. elegans)	Up-regulated	Cell fate determination, TGFβ signaling
Map2k1	mitogen-activated protein kinase kinase 1	Down-regulated	activated Map2k1 induces transdifferentiation of RPE cells to neural retina by inhibiting Mitf
Mbtps1	membrane-bound transcription factor peptidase, site 1	Down-regulated	Diluted hair with white base (melanocyte death)
Med1	mediator complex subunit 1	Up-regulated	Low retinal pigmentation (before embryonic lethality)
Myc	myelocytomatosis oncogene	Down-regulated	Transcription factor, regulator of cell proliferation;<br>Pigmentary spotting, not head
Notch1	Notch gene homolog 1 (Drosophila)	Down-regulated	Scattered grey hairs, when KO targetted to melanocytes (Tyr-Cre)
Notch2	Notch gene homolog 2 (Drosophila)	Down-regulated	Scattered grey hairs, when KO targetted to melanocytes (Tyr-Cre).<br>All grey with Notch1 KO, eventually white
Pax3	paired box 3	Down-regulated	transcription factor； neural tube development
Pax6	paired box 6	Down-regulated	transcription factor;<br>Eye abnormalities can include reduced RPE, also distal/ventral white spotting
Pdgfb	platelet derived growth factor, B polypeptide	Down-regulated	Cardiovascular and eye defects include abnormal RPE, microphthalmia

Pdgfc	platelet-derived growth factor, C polypeptide	Down-regulated	Depigmented spots in the retina
Phactr4	phosphatase and actin regulator 4	Up-regulated	Neuroblast overgrowth; outgrowths in RPE
Pitx3	paired-like homeodomain transcription factor 3	Down-regulated	Transcription factor. CNS neuronal differentiation; Eye abnormalities including hyperpigmentation around embryonic pupil
Rb1	retinoblastoma 1	Down-regulated	Growth-inhibitor, melanocyte over-proliferation in culture
Recql4	RecQ protein-like 4	Down-regulated	Mutant mice show growth retardation and skin abnormlaities, including patches of colorless hair; premature graying of fur
Sox10	SRY (sex determining region Y)-box 10	Down-regulated	Transcription factor; White spotting, megacolon and other neural crest defects
Tbx15	T-box 15	Down-regulated	transcription factor;Ear shape; skeletal, altered dorsoventral color pattern with At, ae
Timp3	tissue inhibitor of metalloproteinase 3	Down-regulated	Protease inhibitor and can block VEGF binding to receptor; Abnormal RPE morphology
Zbtb17	zinc finger and BTB domain containing 17	Down-regulated	Transcription factor;Darkened coat (mixed strain background); dark skin, dark dermis around hairs, Abnormal follicles

(b) Components of melanosomes and their precursors

Dct	dopachrome tautomerase	Down-regulated	melanosomal enzyme;Dilution of eumelanin color
Pmel	premelanosome protein	Down-regulated	premelanosome protein, melanosomal matrix protein, trapping of melanin intermediates
Tyr	tyrosinase	Down-regulated	melanogenic enzyme
Tyrp1	tyrosinase-related protein 1	Down-regulated	melanosomal enzyme/stabilizing factor

(c) Melanosome construction / protein routing (HPS-related)

Ap3b1	adaptor-related protein complex 3, beta 1 subunit	Down-regulated	Organellar protein routing
Ap3d1	adaptor-related protein complex 3, delta 1 subunit	Up-regulated	Organellar protein routing
Bloc1s3	biogenesis of lysosomal organelles complex-1, subunit 3	Down-regulated	a component of the BLOC1 protein transport complex
Dtnbp1	dystrobrevin binding protein 1	Up-regulated	lysosome-related organelles complex 1
Fig4	FIG4 phosphoinositide 5-phosphatase	Down-regulated	late endosome-lysosome axis, Pale skin neonatally, few hair follicles, clumped melanosomes (and immune effects etc)
Hps3	Hermansky-Pudlak syndrome 3	Up-regulated	Organelle biogenesis
Lyst	lysosomal trafficking regulator	Down-regulated	Organelle biogenesis and size
Rab38	RAB38, member RAS oncogene family	Down-regulated	Targeting of Tyrp1 protein to the melanosome
Rabggta	Rab geranylgeranyl transferase, a subunit	Down-regulated	Organelle biogenesis

(d) Melanosome transport

Mlph	melanophilin	Down-regulated	melanosome transport
Myo5a	myosin VA	Down-regulated	melanosome transport

(e) Eumelanin and Pheomelanin

a	nonagouti	Up-regulated	Eumelanin / pheomelanin switch
Atrn	attractin	Down-regulated	Eumelanin / pheomelanin switch (among others)

Eda	ectodysplasin-A	Up-regulated	Sweat gland, tooth and hair morphogenesis
Edar	ectodysplasin-A receptor	Down-regulated	hyperpigmentation, hair morphogenesis
Edaradd	EDAR (ectodysplasin-A receptor)-associated death domain	Down-regulated	Ectodysplasin A receptor-associated death domain
Mc1r	melanocortin 1 receptor	Down-regulated	Eumelanin / pheomelanin switch
Mgrn1	mahogunin, ring finger 1	Down-regulated	Melanin color Spongiform Degeneration
Pmch	pro-melanin-concentrating hormone	Down-regulated	Precursor of the melanin-concentrating hormone
Slc7a11	solute carrier family 7 (cationic amino acid transporter, y+ system), member 11	Down-regulated	Cystine transporter needed for pheomelanin synthesis
Smarca5	SWI/SNF related, matrix associated, actin dependent regulator of chromatin, subfamily a, member 5	Down-regulated	Dominant mottled coat with A^{y}/-
Smchd1	SMC hinge domain containing 1	Down-regulated	

(f) Systemic effects

Dst	dystonin	Up-regulated	Pale skin
Elovl3	elongation of very long chain fatty acids (FEN1/Elo2, SUR4/Elo3, yeast)-like 3	Down-regulated	Abnormal hairs with scattered hyperpigmentation
Elovl4	elongation of very long chain fatty acids (FEN1/Elo2, SUR4/Elo3, yeast)-like 4	Up-regulated	Abnormal retinae including RPE, macular distrophy with flecks
Ercc2	excision repair cross-complementing rodent repair deficiency, complementation group 2	Down-regulated	Nucleotide excision repair (NER)

Hs2st1	heparan sulfate 2-O-sulfotransferase 1	Down-regulated	abnormal RPE differentiation
Pdpk1	3-phosphoinositide dependent protein kinase 1	Down-regulated	Abnormal eye pigmentation
Polg	polymerase (DNA directed), gamma	Up-regulated	General premature ageing including coat graying
Rbp1	retinol binding protein 1, cellular	Down-regulated	abnormal RPE morphology
Rbpj	recombination signal binding protein for immunoglobulin kappa J region-like	Down-regulated	Hair depigmentation, other melanocytes not affected
Rps19	ribosomal protein S19	Down-regulated	increased number of melanocytes in the epidermis
Rps20	ribosomal protein S20	Up-regulated	increased number of melanocytes in the epidermis
Slc31a1	solute carrier family 31, member 1	Up-regulated	Copper deficiency, hypopigmentation
